# Comparative effectiveness of different therapies for *Clostridioides difficile* infection in adults: a systematic review and network meta-analysis of randomized controlled trials

**DOI:** 10.1016/j.lanepe.2024.101151

**Published:** 2025-01-05

**Authors:** Dániel Steve Bednárik, Kincső Csepke Földvári-Nagy, Viktor Simon, Anett Rancz, Noémi Gede, Dániel Sándor Veres, Panagiotis Paraskevopoulos, Tamás Schnabel, Bálint Erőss, Péter Hegyi, Katalin Lenti, László Földvári-Nagy

**Affiliations:** a Centre for Translational Medicine, Semmelweis University, Budapest, Hungary; b Heim Pál National Pediatric Institute, Budapest, Hungary; c School of Life Sciences, University of Warwick, Coventry, United Kingdom; d Faculty of Health Sciences, Semmelweis University, Budapest, Hungary; e Department of Biophysics and Radiation Biology, Semmelweis University, Budapest, Hungary; f Department of Gastroenterology, Skien Hospital, Telemark Hospital Trust, Skien, Norway; g Institute of Pancreatic Diseases, Semmelweis University, Budapest, Hungary; h Institute for Translational Medicine, University of Pécs, Medical School, Pécs, Hungary; i Department of Morphology and Physiology, Faculty of Health Sciences, Semmelweis University, Budapest, Hungary

**Keywords:** Clostridioides difficile infection, CDI, Clostridium difficile, Network meta-analysis, Fecal microbiota transplantation, FMT, Treatment

## Abstract

**Background:**

*Clostridioides difficile* infection (CDI) is a leading cause of healthcare-associated diarrhea, with substantial morbidity and mortality. CDI is a severe and growing problem with numerous treatment options. We evaluated the effectiveness of all therapies in recurrent and non-recurrent infections and their prevention.

**Methods:**

This network meta-analysis and systematic review of randomized controlled trials (RCTs) compared all CDI therapies and preventions. We included RCTs published until 19 August 2024 and focused on adult population. We performed a systematic search in MEDLINE, EMBASE, and Cochrane Central Register of Controlled Trials. Inclusion criteria were patients: adults (>16) treated against CDI; study type: randomized controlled trial; outcome: cure rate, recurrence or effectiveness of prevention. Any publication not meeting all criteria was considered to be ineligible and excluded. We applied random-effects meta-analysis using frequentist methods. We reported our main results as odds ratios (as a symmetric effect size measure, OR) with 95% confidence interval (95% CI). We used the Cochrane risk-of-bias tool to assess the risk of bias. Our study protocol was preregistered in PROSPERO (CRD42022371210).

**Findings:**

We assessed 73 RCTs with 28 interventions, involving 27,959 patients (49.2% female) in five networks. Fecal microbiota transplantation (FMT) was the most effective treatment in terms of the cure rate overall (P-score: 0.9952) and in recurrent cases (P-score: 0.9836). In recurrent cases, fidaxomicin (P-score: 0.6734) showed significantly greater effectiveness than vancomycin (P-score: 0.3677) and tolevamer (P-score: 0.0365). For non-recurrent CDI treatments ridinilazole, fidaxomicin, FMT and nitazoxanide were equally effective. Ridinilazole (P-score: 0.7671) and fidaxomicin (P-score: 0.7627) emerged as the most effective in preventing recurrence. Probiotics were not effective in preventing CDI, since network meta-analyses did not show significant differences between probiotics and placebo. In probiotics’ subgroups pairwise meta-analyses *Lactobacillaceae* proved to be significantly more effective in prevention than placebo. Oral and colonoscopic FMT administration methods were equally effective. The study-level aggregated risk of bias of the publications included ranged from low to high. We observed relevant heterogeneity among studies in therapeutic doses, treatment durations, and follow-up times.

**Interpretation:**

The superiority of FMT in the treatment of CDI highlights the potential for increased use of FMT in clinical settings. Further research on optimizing FMT protocols and exploring its long-term safety and efficacy in larger samples is needed. Our findings suggest that the preventive use of probiotics might be questioned.

**Funding:**

None.

**Research in context**

**Evidence before this study**

The systematic search was performed in MEDLINE, EMBASE, and Cochrane Central Register of Controlled Trials databases on 11 November 2022 and updated on 19 August 2024 using the following search key: (c.diff OR ((clostr∗ OR c.) AND difficile)) AND (random∗ OR blind∗ OR placebo OR rct). Five network meta-analyses have studied different aspects of CDI treatment options. The network meta-analysis of Beinortas et al. (2018) analyzing non-recurrent cases showed the superiority of fidaxomicin and teicoplanin. Two other network meta-analysis by Rokkas et al. (2019) and Dembrovszky et al. (2021) analyzed recurrent cases and concluded that FMT is the most effective therapy against CDI. Sridharan et al. (2019) investigated the effectiveness of various antimicrobial therapies highlighted the effectiveness of fidaxomicin and teicoplanin. Alhifany et al. (2019) studied the effectiveness of FMT and bezlotoxumab in recurrent CDI.

**Added value of this study**

This network meta-analysis and systematic review, the largest to date, provides the most up-to-date therapeutic options for CDI and its prevention. Our analysis had five significant endpoints (overall cure rate, cure rate of non-recurrent and recurrent cases, rate of recurrence and prevention), providing the most detailed review of currently published randomized controlled trials. For the cure rate endpoints, we compared 16 interventions using direct and indirect comparison methods involving 5654 patients and for the prevention of recurrence, we compared 15 interventions involving 4592 patients. For prevention, we compared 10 types of interventions involving 17 713 patients. We also performed a pairwise comparison of different types of probiotics. Thus, this study provides the most comprehensive insight into the relative effectiveness of CDI therapies for recurrent and non-recurrent cases, and infection prevention. It offers the most detailed and up-to-date comparisons and analyses of treatment outcomes, including the efficiency of probiotics in preventing CDI and the administration methods of FMTs.

**Implications of all the available evidence**

This study is significant for clinical practice, future research, and policy making. Future research should also explore the optimization of FMT, including administration methods and long-term outcomes, to better integrate this treatment into standard practice. Our findings could inform CDI treatment guidelines, prioritizing FMT, especially in recurrent cases, and fidaxomicin in non-recurrent cases. Our data may urge the reconsideration of probiotics administration with preventative aims.

## Introduction

The infection caused by *Clostridioides difficile* (CD), formerly *Clostridium difficile*, is the most common cause of healthcare-associated diarrhea worldwide. CD is a Gram-positive, obligate anaerobe, spore-forming bacterium.^1^ The latest data indicate that nearly half a million people in the United States are infected with CD each year,^2^ with a death toll ranging from 15,000 to 30,000 in the USA annually.^3^

The diagnosis of CD infection (CDI) is based on clinical symptoms, such as diarrhea more than three times a day, and the detection of CD toxin.^4^ Not all CD carriers exhibit symptoms; approximately 5% of the adult population and 15–70% of infants are asymptomatic carriers.^4^ Asymptomatic carriers can spread the infection, and CD spores can survive in the environment for months. The spores, which are transmitted via fecal-oral routes, are widely distributed in the environment. The spores are highly resistant to alcohol-based hand sanitizers, heat, and various antibiotics, and are prevalent in hospital and nursing home settings. Factors, such as older age, inflammatory bowel disease, immunosuppressives, gastrointestinal surgery, and chronic kidney failure increase the risk of CDI.^5^ As a nosocomial infection, it is usually transmitted through the hands of medical staff or equipment.^4^ Handwashing with soap and water, and using rubber gloves are crucial measures among hospital staff to prevent the transfer of infection between patients.^6^ The use of various antibiotics, including ampicillin, amoxicillin, clindamycin, cephalosporins, and fluoroquinolones, is an important risk factor for the development of CDI, as they destroy the normal intestinal flora. Therefore, antibiotics play a dual role in the treatment of CDI and are partly responsible for it.^5^

Until the mid-2010s, metronidazole and vancomycin were the first-line treatment for CDI.^5^ The Clinical Practice Guideline by the Infectious Diseases Society of America^7^ recommends fidaxomicin over vancomycin both for the first time and for the first recurrent infection, with the addition of bezlotoxumab as an adjunct in subsequent recurrences. The European Society of Clinical Microbiology and Infectious Diseases guideline recommends fecal microbiota transplantation (FMT) after the second infection recurrence.^8^ In primary cases, fidaxomicin is recommended if available, otherwise vancomycin should be used. In the first recurrent cases, therapy should be supplemented with bezlotoxumab, and if there is still a recurrent case, then FMT is recommended.

We performed a network meta-analysis for non-recurrent cases in which the role of FMT had not yet been investigated. The previous analysis showed the superiority of fidaxomicin treatment.^9^ Two network meta-analyses examined recurrent cases, which found FMT to be the best therapy.^10^^,^^11^ Another meta-analysis investigated the effectiveness of various antimicrobial therapies treatments., which highlighted the importance of fidaxomicin and teicoplanin.^12^ A meta-analysis studied the recurrence prevention potential of FMT and bezlotoxumab.^13^

Given the global challenge of CDI, this research aims to identify the most effective treatment. We completed a network meta-analysis focusing on cure rates of recurrent and non-recurrent cases, recurrence rate, and prevention.

## Methods

### Study design and selection criteria

To comprehensively identify the most effective therapy against CDI, we conducted a network meta-analysis and systematic review. The research was completed following the PRISMA 2020 statement^14^ (PRISMA-NMA extension checklist,^15^ Supplementary Table S1) and the Cochrane Handbook.^16^ The PROSPERO registration number of this research is CRD42022371210 (Supplementary Methods S1).

Articles on adult patients (>16 years of age) (P) with any CDI treatment were included (I/C). Our outcomes (O) were cure rate, recurrence rate, and effectiveness of prevention. We only included randomized controlled trials (RCTs) to achieve the highest level of evidence. Publications not meeting all criteria were considered to be ineligible and excluded.

European Society of Clinical Microbiology and Infectious Diseases (ESCMID) define cure as treatment response without recurrence of CDI during follow-up.^8^ The most recent Infectious Diseases Society of America (IDSA) guidelines does not specifically define cure.^17^ The updated guideline defines cure as the passing of symptoms.^7^ Trials generally define cure rate as the number/ratio of patients cured by the end of the follow-up period. The trials consider it a cure when diarrhea and other symptoms (e.g., abdominal pain, fever) cease, no recurrence during the follow-up period and recovery is microbiologically confirmed. Since the combinations of criteria used differed between trials, in our analysis we considered a patient cured if it was defined cured by the publication.

Recurrent CDI is defined as a forthcoming episode occurring within eight weeks after the onset of the previous infection.^8^^,^^17^^,^^18^ This definition was followed by a minority of the clinical trials. The majority of the studies used an approximately 1-month cut-off, while three used a longer time. The different lengths of time were mixed for the different treatments, so it was not possible to perform a subgroup analysis on this basis because the network would have fallen apart. For these reasons, in the case of recurrence, we accepted the definition used by the given publication.

For the cure rate, we also performed a subgroup analysis: recurrent and non-recurrent cases. We considered a study to be recurrent if this was clearly indicated by the authors or clearly stated in the Methods section. We considered those trials as non-recurrent, similarly to other meta-analyses,^9^ which were not defined as recurrent. In terms of prevention, we investigated which therapy is the most effective in preventing the development of CDI.

### Search strategy

We systematically searched three databases: MEDLINE (via PubMed), EMBASE (via Embase.com), and the Cochrane Central Register of Controlled Trials (CENTRAL). The search key is available in the Appendix (Supplementary Methods S2). The databases were originally downloaded on 11 November 2022. Due to delays in the process of publication selection, data analysis, and manuscript writing the first systematic search was complemented on 19 August 2024, using the same search key and databases, to identify and include the latest publications as well. We included articles published until 19 August 2024, without language restrictions. To achieve the highest level of evidence, we exclusively included RCTs published in peer-reviewed journals and excluded all non-peer-reviewed datasets (conference abstracts, unpublished data, etc.). After duplicate removal in Endnote, four authors (DSB, KCSFN, VS, and TS) independently completed the selection process, scrutinizing all articles using the Rayyan system.^19^ The inclusion of questionable articles were decided by a senior researcher (LFN). At each selection step, Cohen's kappa coefficient was calculated to measure agreement between assessors.^20^

### Study selection and data extraction

Two authors (DSB and KCSFN) independently collected relevant data from the corresponding articles, listed in the baseline characteristic tables (Supplementary Tables S2–S4).

The risk of bias assessment was separately performed by two authors (DSB and KCSFN) using Version 2 of the Cochrane risk of bias tool (RoB 2). Any discrepancies in the results were resolved by a third co-author (LFN). We used the Grading of Recommendations Assessment, Development and Evaluation Working Group modality (GRADE) approach to assess certainty of evidence for both outcomes for all pairwise comparisons.^21^ The process was separately performed by two authors (DSB and KCSFN) in the CINeMA system, which was explicitly designed for network meta-analyses.^22^ Any discrepancies were resolved by a third co-author (LFN).

### Data analysis

A frequentist method was used to perform the meta-analyses. As we assumed considerable between-study heterogeneity due to the nature of medical treatments and conditions, a random-effects model was used to pool effect sizes with a 95% confidence interval (95% CI).

Comparisons of several CDI treatments were assessed by network meta-analyses (NMAs) using odds ratio (OR) as a symmetric effect size measure. P-scores^23^ were calculated to indicate which treatments were more or less likely to have the most significant benefits or negligible adverse effects; the score ranged from 0 to 1, representing the theoretically worst and best treatments, respectively, compared to the other treatments. Net heat plots and forest plots were used to assess consistency.^24^ A direct evidence plot was generated to examine the reliability of effect size estimates within the NMA model. Small study publication bias was assessed by visual inspection of funnel-plots and calculating modified Egger's test P-value.^25^ However, we kept in mind that the test had limited diagnostic assessment below approximately 10 studies or approximately 25 study records.

The NMA analysis was supplemented with pairwise meta-analyses to directly compare the proportion of cured patients among *Saccharomyces* vs. placebo and *Lactobacillaceae* vs. placebo. The proportion of cured patients or risk ratio (RR) and additionally risk difference (RD, as experimental–control group risks) and the number needed to treat (NNT) were also determined as effect size measures for better interpretability.

For FMT treatment, we indirectly compared the proportion of cured patients via colonoscopy (or enema) vs. oral (or nasogastric tube) methods. To be able to include both single-arm and double-arm studies (where more results were available in the same study with different treatments (subgroups)), we used a 3-level model to pool proportions (meta-analysis for proportion).

We summarized the findings from both pairwise and 3-level meta-analyses in forest plots. Between-study heterogeneity was described by the between-study variance (τ^2^) and the I^2^ statistics. We reported the prediction interval when it was meaningful (i.e., when there were enough studies and sample sizes).

Analyses were performed using the *R software* (version 4.3.2),^26^ using *Netmeta* (version 2.7–0),^27^ *Pairwise* (version 0.6.0-0), and *BUGSnet* (version 1.1.0)^28^ packages for NMA; *meta* (v6.2.1)^29^ package for basic meta-analysis calculations and plots; *metafor* (v4.0.0)^30^ for 3-level models, and *dmetar* (v0.0.9000)^31^ package for additional influential analysis calculations and plots.

More details on the statistical methods are available in Supplementary Methods S3.

### Role of the funding source

There was no funding source for this study.

## Results

**Fig. 1:**
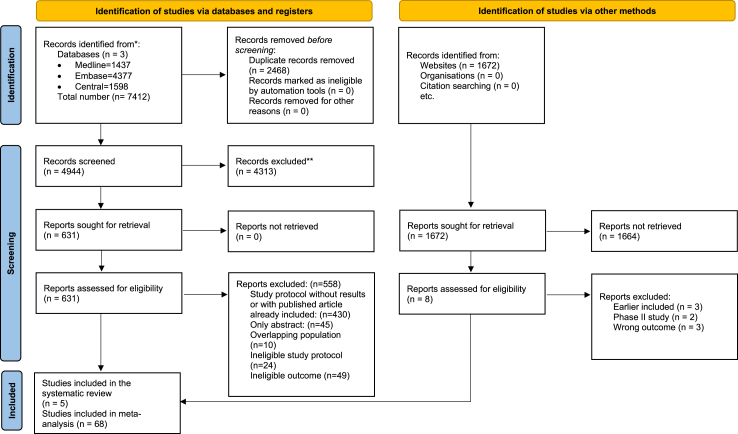
**PRISMA flowchart of the selection process**. The structure of the flowchart is based on The PRISMA 2020 statement.^32^

Following our thorough selection protocol, we found 73 articles eligible for our network meta-analysis and systematic review. We performed our selection by title and abstract with a Cohen's kappa index of 0.91, and the full-text selection with a Cohen's kappa index of 0.93. After this, 73 articles were eligible for data extraction. Of these, 68 articles were included in the analysis, and 5 articles were used for the systematic review. We excluded overlapping populations and included adult population only (>16 years of age). The flowchart of our selection is shown in Fig. 1.

The included studies were published between 1983 and 2024. Baseline characteristics are included in the Baseline characteristics tables (Supplementary Tables S2–S4).

Our main endpoints are shown in appendix (Supplementary Figure S1).

In Supplementary Tables S5–S9, the included treatments and pairwise comparisons can be tracked for each analysis.

### Network meta-analysis of cure rate results

#### Overall cure rate

**Fig. 2:**
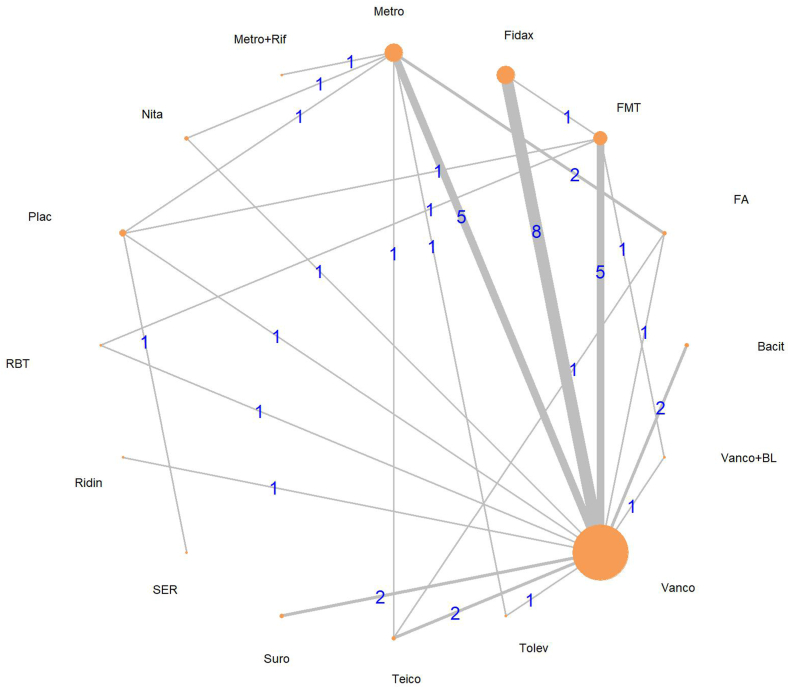
**Network plot of possible treatments for cure rate**. Every knot represents a different therapy for CDI. The larger the knot, the more studies included the treatment. Every edge compares different therapies. The width and the number above indicate how many studies investigated this comparison. Bacit, Bacitracin; FA, Fusidic acid; Fidax, Fidaxomicin; FMT, Fecal microbiota transplantation; Metro, Metronidazole; Metro + rif, Metronidazole + Rifampin; Nita, Nitazoxanide; Plac, Placebo; RBT, Rectal bacteriotherapy; Ridin=Ridinazole; SER, SER 109; Suro, Surotomycin; Teico, Teicoplanin; Tolev, Tolevamer; Vanco, Vancomycin; Vanco + BL, Vancomycin + bowel lavage.

Our main outcome was cure rate. We identified 16 interventions from 29 articles.^33^, ^34^, ^35^, ^36^, ^37^, ^38^, ^39^, ^40^, ^41^, ^42^, ^43^, ^44^, ^45^, ^46^, ^47^, ^48^, ^49^, ^50^, ^51^, ^52^, ^53^, ^54^, ^55^, ^56^, ^57^, ^58^, ^59^, ^60^, ^61^ The analysis included 5654 patients, 120 possible pairwise comparisons, and 25 pairwise comparisons with direct data (Supplementary Table S10). The network plot is shown in Fig. 2.

FMT significantly outperformed other treatments supported by the P-score (Table 1, Supplementary Table S11).

**Table 1:** League table of possible treatments for cure rates.

Possible treatments are arranged in descending order of the P score in blue bars. P scores are given in brackets after treatment names. Direct comparisons are shown in the area above these blod, whereas below, the direct and indirect (estimated) ones are pooled together as network estimates. Odds ratios are given in the cells. We compare one treatment on the left side with another one on the right side, indicating a greater odds ratio of recovery. The 95% confidence interval is shown in brackets. Significant results are marked in green.

Bacit, Bacitracin; FA, Fusidic acid; Fidax, Fidaxomicin; FMT, Fecal microbiota transplantation; Metro, Metronidazole; Metro + rif, Metronidazole + Rifampin; Nita, Nitazoxanide; Plac, Placebo; RBT, Rectal bacteriotherapy; Ridin, Ridinilazole; SER=SER 109; Suro, Surotomycin; Teico, Teicoplanin; Tolev, Tolevamer; Vanco, Vancomycin; Vanco + BL, Vancomycin + bowel lavage.

It must be noted that in all cases, antibiotic treatment, typically vancomycin, was administered as pre-treatment before FMT therapy. After FMT {P-score: 0.9952}, fidaxomicin {0.7616} and nitazoxanide {0.7152} showed similar P-scores. As Table 1 shows, FMT exhibited statistically significant and relevant superiority over most of the other therapies. Fidaxomicin {0.7616} was significantly superior to vancomycin {0.5321}, placebo {0.1812} and tolevamer {0.0475}. SER 109 {0.6536} was also significantly superior to placebo {0.1812} and tolevamer {0.0475}. The evidence plot, forest plot of consistency analysis, net heat plot, and funnel plot for the analysis are in the appendix (Supplementary Figures S3–S6).

Due to its clinical significance, the cure rate results were also analyzed by subgroups of recurrent and non-recurrent cases.

#### Cure rate—recurrent cases subgroup analysis

To analyze exclusively recurrent cases, we explored 11 interventions from 10 studies^33^^,^^36^^,^^37^^,^^45^, ^46^, ^47^, ^48^^,^^50^^,^^56^^,^^60^ involving 2283 patients. There were 55 total possible pairwise comparisons and 17 pairwise comparisons with direct data (Supplementary Table S12). The network plot is shown in Supplementary Figure S7.

In recurrent cases, FMT showed statistically significant superiority over alternative therapies, as evidenced in Table 2 and Supplementary Table S13. Fidaxomicin was significantly superior to vancomycin and tolevamer. It must be noted that recipients of FMT therapy also received antibiotic pretreatment.

**Table 2:** League table of possible treatments for cure rates in recurrent cases.

Possible treatments are arranged in descending order of the P score in blue bars. P scores are given in brackets after treatment names. Direct comparisons are shown above these blod, whereas below, direct and indirect (estimated) ones are pooled together as network estimates. Odds ratios are given in the cells. We compare one treatment on the left side with another one on the right side, indicating a greater odds ratio of recovery. The 95% confidence interval is shown in brackets. Significant results are marked in green.

FA, Fusidic acid; Fidax, Fidaxomicin; FMT, Fecal microbiota transplantation; Metro, Metronidazole; Nita, Nitazoxanide; RBT, Rectal bacteriotherapy; Suro, Surotomycin; Teico, Teicoplanin; Tolev, Tolevamer; Vanco, Vancomycin; Vanco + BL, Vancomycin + bowel lavage.

The evidence plot, forest plot of consistency analysis, net heat plot, and funnel plot for the analysis can be viewed in the appendix (Supplementary Figures S8–S11).

#### Cure rate—non-recurrent cases subgroup analysis

To analyze non-recurrent cases, we used 12 interventions from 18 studies^34^^,^^38^, ^39^, ^40^, ^41^, ^42^, ^43^, ^44^^,^^47^^,^^49^^,^^51^^,^^53^, ^54^, ^55^^,^^57^, ^58^, ^59^^,^^61^ involving 3189 patients. We performed 66 total possible pairwise comparisons and 13 pairwise comparisons using direct data (Supplementary Table S14). The network plot is presented in Supplementary Figure S12.

Table 3 and Supplementary Table S15 show that fidaxomicin, surotomycin, ridinilazole, vancomycin, and FMT were all significantly better than placebo, but there was no statistically significant difference between the five therapies.

**Table 3:** League table of possible treatments for cure rates in non-recurrent cases.

Possible treatments are arranged in descending order of the P score in blue bars. P scores are given in brackets after treatment names. Direct comparisons are shown above these blod, whereas below, the direct and indirect (estimated) ones are pooled together as network estimates. Odds ratios are given in the cells. We compare one treatment on the left side with another one on the right side, indicating a greater odds ratio of recovery. The 95% confidence interval is shown in brackets. Significant results are marked in green.

Bacit, Bacitracin; FA, Fusidic acid; Fidax, Fidaxomicin; FMT, Fecal microbiota transplantation; Metro, Metronidazole; Metro + rif, Metronidazole + Rifampin; Nita, Nitazoxanide; Plac, Placebo; Ridin, Ridinilazole; Suro, Surotomycin; Teico, Teicoplanin; Vanco, Vancomycin.

The evidence plot, forest plot of consistency analysis, net heat plot, and funnel plot for the analysis are in the appendix (Supplementary Figures S13–S16).

We also performed sub-group analyses by dose of vancomycin as the most commonly used treatment for CDI. The subgroup analyses of cure rate results are in the Appendix (Supplementary Results S1—Subgroup analyses of cure rate results by dose of vancomycin treatments).

### Network meta-analysis of recurrence result

We also examined recurrence outcomes, i.e., which therapy was the most effective in preventing recurrent CDI. An examination of 15 interventions from 22 studies^33^^,^^36^^,^^38^^,^^40^^,^^41^^,^^43^, ^44^, ^45^, ^46^, ^47^^,^^49^, ^50^, ^51^^,^^53^^,^^54^^,^^57^^,^^58^^,^^60^, ^61^, ^62^, ^63^, ^64^ involving 4592 individuals was conducted. There were 105 total possible pairwise comparisons and 19 pairwise comparisons with direct data (Supplementary Table S16). The network plot is presented in Supplementary Figure S17.

Considering Table 4 and Supplementary Table S17, tolevamer proved to be the best therapy for preventing CDI recurrence, and teicoplanin and fidaxomicin showed similarities in effectiveness. Tolevamer was significantly better than surotomycin, vancomycin, metronidazole, metronidazole-rifampicin, the combination of vancomycin and bowel lavage, fusidic acid, and bacitracin. Teicoplanin proved to be significantly better than fusidic acid. Ridinilazole was significantly better than vancomycin, metronidazole, fusidic acid and bacitracin. Fidaxomicin proved to be significantly better than surotomycin, vancomycin, metronidazole, fusidic acid, and bacitracin.

**Table 4:** League table of possible treatments in recurrence.

Possible treatments are arranged in descending order of the P score in blue bars. P scores are given in brackets after treatment names. Direct comparisons are shown above these blod, whereas below, the direct and indirect (estimated) ones are pooled together as network estimates. Odds ratios are given in the cells. We compare one treatment on the left side with another one on the right side, indicating a greater odds ratio of recovery. The 95% confidence interval is shown in brackets. Significant results are marked in green.

Bacit, Bacitracin; FA, Fusidic acid; Fidax, Fidaxomicin; FMT, Fecal microbiota transplantation; FMT-Bez, Fecal microbiota transplantation + Bezlotoxumab; FMT-L, Fecal microbiota transplantation + Lactobacillus; Metro, Metronidazole; Metro + rif, Metronidazole + Rifampin; Nita, Nitazoxanide; Ridin, Ridinilazole; Suro, Surotomycin; Teico, Teicoplanin; Tolev, Tolevamer; Vanco, Vancomycin; Vanco + BL, Vancomycin + bowel lavage.

The evidence plot, forest plot of consistency analysis, net heat plot, and funnel plot for the analysis can be viewed in the appendix (Supplementary Figures S18–S21).

We also performed a sub-group analysis by dose of vancomycin as the most commonly used treatment for CDI. The subgroup analysis of recurrence result is in the Appendix (Supplementary Results S2—Subgroup analysis of recurrence result by dose of vancomycin treatments).

### Network meta-analysis of prevention effectiveness results

We analyzed which therapy prevented the development of CDI. We examined 10 interventions from 29 studies^65^, ^66^, ^67^, ^68^, ^69^, ^70^, ^71^, ^72^, ^73^, ^74^, ^75^, ^76^, ^77^, ^78^, ^79^, ^80^, ^81^, ^82^, ^83^, ^84^, ^85^, ^86^, ^87^, ^88^, ^89^, ^90^, ^91^, ^92^, ^93^ involving 17,713 patients. There were 45 total possible pairwise comparisons and 13 pairwise comparisons with direct data (Supplementary Table S18). The network plot is shown in Supplementary Figure S22.

Table 5 and Supplementary Table S19 show that oligofructose was the most effective treatment, followed by a combination of actoxumab and bezlotoxumab. The P-score difference between the two treatments was not significant. The results in Table 5 proved oligofructose to be significantly better than probiotics, bezlotoxumab, the vaccine, and placebo.

**Table 5:** League table of possible treatments for prevention.

Possible treatments are arranged in descending order of the P score in blue bars. P scores are given in brackets after treatment names. Direct comparisons are shown above these blod, whereas below, the direct and indirect (estimated) ones are pooled together as network estimates. Odds ratios are given in the cells. We compare one treatment on the left side with another one on the right side, indicating a greater odds ratio of recovery. The 95% confidence interval is shown in brackets. Significant results are marked in green.

AB, Antibiotic; ACT, Actoxumab; ACT + BEZ,Actoxumab + Bezlotoxumab; BEZ, Bezlotoxumab; LF, Lactoferrin; Oligo, Oligofructose; Plac, Placebo; Probi, Probiotics; RBX, RBX2660; Vac, Vaccine.

The evidence plot, forest plot of consistency analysis, net heat plot, and funnel plot for the analysis can be viewed in the appendix (Supplementary Figures S23–S26).

More detailed analysis of prevention methods for CDI development is in the Appendix (Supplementary Results S3—Additional analysis of prevention therapies against the development of CDI).

### Additional analyses

On the basis of available articles, we separately examined the cure rates achieved with colonoscopy or oral FMT therapy.^63^^,^^94^, ^95^, ^96^, ^97^, ^98^, ^99^

We found a cure rate of 85% (CI: 0.70–0.94) for colonoscopy, and 86% (CI: 0.66–0.95) for the oral method (Supplementary Figure S52). We found no statistically significant difference between the two methods, which is not clinically relevant.

Further analysis of two-arm studies can be found in Supplementary Results S4.

We were able to perform consistency analysis for all the five endpoints. Forest plots show the results of the consistency tests (Supplementary Figures S4, S9, S14, S19, S24). The direct, indirect and network estimates overlap, so the network is considered consistent.

The net heat plots (Supplementary Figures S5, S10, S15, S20, S25) graphically identify the inconsistencies within the networks. The net heat plot of cure rate (Supplementary Figure S5) shows some inconsistencies in two cases. In the case of Johnson et al. (1992)^59^ (metronidazole, vancomycin, placebo), the reason may be that asymptomatic patients were included in the study. In the Teasley et al. (1983)^42^ (metronidazole, vancomycin) trial, vancomycin was administered to patients at a dose four times higher than in other trials.

We performed tests for small study effects for all five endpoints, which were visualized by Funnel plots. We also performed Egger's test, which was greater than 0.05 in all cases, so no small study effect could be detected (Supplementary Figures S6, S11, S16, S21, S26).

The aggregated risk of bias of the publications analyzed ranged from low to high (Supplementary Table S35).

The level of bias in randomization was generally low. There were some concerns about the measurement of the results.

On the basis of the CINeMA (grading method) assessment,^22^ the quality of evidence for the meta-analyses were low (Supplementary Tables S36–S38).

In some articles, the primary outcome differed from our main endpoints, raising concerns about the selection of reported results. We found a low risk of bias in the randomization process and outcome measurements.

## Discussion

To the best of our knowledge, this analysis provides the most up-to-date and comprehensive analysis of therapeutic options for CDI and its prevention, using a network meta-analysis of RCTs to compare all possible treatments.

In terms of cure rate, FMT proved to be the most effective therapy in the all-case comparison compared to other therapies included in our study (Table 1, Supplementary Table S11). One trial had to be terminated prematurely, as FMT was much more effective than placebo.^34^

We found no statistical or clinical differences in the cure rates of the other therapies included. Among the antibiotic therapies, fidaxomicin proved significantly better than vancomycin, placebo or tolevamer, making it the most effective antibiotic therapy against CDI. Fidaxomicin, a narrow-spectrum antibiotic, is effective against CDI, *Staphylococci*, and *Enterococci*, but its effectiveness against Gram-negative organisms and fungi is negligible.^100^ Moreover, fidaxomicin is poorly absorbed from the intestines, and has a similarly low spectrum of systemic side effects as vancomycin.^100^, ^101^, ^102^ Furthermore, fidaxomicin has a prolonged post-antibiotic effect against CDI, which is not observed with vancomycin. Although vancomycin treatment results in the suppression of organisms belonging to the *Bacteroides* group in the stool flora, which are markers of normal anaerobic microflora,^103^ fidaxomicin preserves these bacteria in the flora of patients, essential to avoid recurrence. The spread of Vancomycin-Resistant Enterococci is a concern associated with the use of vancomycin and metronidazole.^104^^,^^105^ Fidaxomicin has a low effect on commensal gut flora.^106^ In addition to the fact that fidaxomicin treatment proved to be more successful than vancomycin, Guery et al. confirmed that fidaxomicin was significantly better at supporting microbiota recovery in patients.^36^

Focusing solely on recurrent cases, we identified FMT as the best therapy. FMT is followed by fidaxomicin. Both therapies showed significantly better results than vancomycin and fidaxomicin proved to be significantly better than metronidazole. Table 2 and P-scores (Supplementary Table S13) showed no decisive difference between the remaining therapies. This conclusion from our network meta-analysis is consistent with the findings of several randomized controlled trials,^33^^,^^48^^,^^56^ and the FMT is already being carried out safely in more and more centers across Europe.^107^

In non-recurrent cases, fidaxomicin emerged as the best treatment option for CDI; however, this was significant only when compared to metronidazole, fusidic acid or placebo. As per current recommendations, antibiotics is the most effective first-line treatment in non-recurrent cases. Of the antibiotics, fidaxomicin showed the most favorable outcomes, whereas vancomycin and metronidazole exhibited lower efficacy. FMT was positioned within the mid-range according to both Table 3 and P-scores (Supplementary Table S15). Nonetheless, FMT's ranking may be attributed to the limited number of studies investigating FMT within the non-recurrent subgroup. Only two trials explored FMT in non-recurrent cases, and in one of these trials, FMT showed so much better results compared to placebo, so the trial had to be terminated early. Both subgroups of this trial received vancomycin pretreatment.^34^^,^^55^

We consider it important to emphasize that the different results of the subgroup analyses are not only and not necessarily caused by differences in the effectiveness of individual treatments in different types of cases, but also by habits and practical considerations. For example, FMT is highly effective in recurrent cases, while in non-recurrent cases it is in the “middle range”. We are convinced that the reason for this is not the actual lower effectiveness of FMT in non-recurrent cases, but the fact that its use in non-recurrent cases has not yet been widely adopted, mainly due to safety, technical and financial reasons. We think that this situation will change and that even in the near future FMT will become more prominent in the treatment of first cases, at least in vulnerable groups (e.g., elderly or immunosuppressed patients). The ongoing FMT trials addressing the first CDI cases will hopefully answer this question.

When assessing the results for surotomycin, it is important to note that its development has been suspended. After a promising phase II trial,^108^ surotomycin was found to be less effective than vancomycin in two phase III trials,^45^^,^^57^ and the development has been discontinued.^8^

We performed a subgroup analysis of the cure rate data by CDI severity (severe, mild/moderate). Some publications reported the percentage of patients with severe or mild/moderate CDI, but the cure rate data were only given in a pooled manner. Only nine of the publications provided sufficiently detailed data. These publications either stated that only severe cases were analyzed or gave the ratio of severe and mild/moderate cases and the cure rates separately. One article gave OR rates in such a way that they could not be analyzed together with the other data sets and was therefore excluded. Three publications compared different methods of FMT treatment, so they fell out of the network. The remaining five publications were included in both subgroups. This is only a very small subset of the total set of 28 publications. In addition, recurrent and non-recurrent cases are also mixed in this subgroup analysis. For all these reasons, no clinically meaningful conclusion could be drawn from the analysis, so these results are not included in the manuscript.

Tolevamer emerged as the most effective in preventing CDI recurrence, followed by teicoplanin and ridinilazole (Table 4, Supplementary Table S17). However, only a few comparisons gave significant results, which are of no clinical relevance, as tolevamer and teicoplanin analyses are from only one study^60^ and two small RCT studies,^50^^,^^58^ respectively. Moreover, the dropout rate was nearly twice as high in the tolevamer group compared to the vancomycin and metronidazole group, which might have significantly influenced the final result.^60^ Additionally in two Phase III trials, tolevamer was shown to be less effective than both vancomycin and metronidazole.^109^^,^^110^ Moreover, tolevamer had a high rate of adverse effects.^60^ The inferiority to antibiotics and the higher rate of side effects lead to the discontinuation of its development.^8^ However, despite the drug's initial clinical failure, its lower recurrence rate suggests potential for use as a supplementary adjunctive therapy or for patients prone to frequent relapses.^111^ A case series^112^ and a prospective observational study^113^ were also conducted in the mid-2010s, which highlighted the benefits of using teicoplanin. However, the one introduced later had more benefits, so current guidelines no longer recommend the use of teicoplanin.^7^^,^^8^ Ridinilazole is a promising new molecule with a favorable recurrence rate.

Given that probiotics are nowadays a favored method of prevention of CDI, we analyzed the effectiveness of *Lactobacillaceae*, *Saccharomyces*, and mixed-group probiotics (containing both *Lactobacillaceae* and *Saccharomyces*). The use of probiotics was not found to have statistically significant benefits, but our subgroup analysis revealed some differences between distinct probiotic types (Table 5, Supplementary Table S19, Supplementary Results S3). We conducted a pairwise meta-analysis, comparing probiotic subgroups (*Lactobacillaceae* and *Saccharomyces*) with placebo. We found no statistically significant differences in case of *Saccharomyces*. *Lactobacillaceae* showed significant difference compared to placebo. In pairwise meta-analysis neither *Lactobacillaceae* nor *Saccharomyces* proved to be significantly better compared to placebo. For all these contradictory results the clinical relevance of probiotics can be judged very carefully, and further studies are needed (Supplementary Results S1). In the light of our findings, we recommend further testing and re-evaluating the assumed benefits of using probiotics in clinical practice.

Moreover, we compared the two FMT administration methods. We could not identify significant differences in effectiveness between colonoscopy and oral FMT methods (Supplementary Figure S52 and Supplementary Results S4).

With our search key, we retrieved articles unsuitable for meta-analysis. Some articles were ineligible for the network and lacked sufficient data. The articles excluded from the meta-analysis raised several questions, such as which antibiotics are most likely to promote the development of CDI and how PPIs increase the risk of developing CDI.

A systematic review of articles that were excluded from the network analysis can be found in the Supplementary Discussion ‒ Systematic review.

This collection of studies excluded from the network, examining antibiotic and probiotic treatments and prevention methods for CDI in different contexts, including surgical and prophylactic conditions, highlights the need for further large-scale research to better understand the role of different methods in managing and preventing CDI, given the preliminary and inconclusive results obtained from their smaller samples.

We conducted our analysis rigorously according to the Prisma Statement 2020^14^ and the Cochrane Guideline.^16^ We preregistered our analysis in PROSPERO(CRD42022371210). The main strength of our analysis is that we included only RCTs, encompassing 73 studies and 27,959 patients, making this network meta-analysis and systematic review the largest in the field to our knowledge. We examined CDI both collectively and as subgroups, achieving multiple endpoints. A limitation of this research is the significant heterogeneity between the populations in the articles. Variations in therapeutic doses, treatment durations, and follow-up times were notable. Individual clinical trials define and apply cure and recurrence in rather different ways. The presence of moderate and high risk of bias in some of the domains is another limitation with our grading, ranging from very low to high (Supplementary Tables S36–S38).

Our recommendations include conducting further investigations on the experiences and effects of FMT. It may also be worth conducting further studies on how FMT therapy can be used in non-recurrent CDI. Moreover, studies do not always provide comprehensive population data on all aspects (ITT, mITT, PP); thus, it would be beneficial to standardize this process or, specify all aspects in RCT studies. Finally, further studies involving many cases are crucial to determine which methods are the most effective in preventing CDI.

Summarizing the available literature helps to integrate scientific results into daily practice, which is essential.^114^^,^^115^ In conclusion, this network meta-analysis and systematic review of 73 studies and 27,959 patients represents the most comprehensive and up-to-date assessment of therapeutic options for CDI in recurrent and non-recurrent cases as well as its recurrence rate and prevention. Our findings indicate that FMT with antibiotic pretreatment is the most effective treatment. For recurrent cases, the most effective treatment is also FMT, whereas in non-recurrent cases, antibiotic treatment showed the highest efficacy, of which fidaxomicin may be the best option. However, due to financial considerations, vancomycin cannot be neglected either. In terms of recurrence and prevention, no significant results were observed between the different treatments. The results of this study emphasize the need for further RCT studies involving many patients, especially those focusing on the prevention of CDI.

No race or ethnicity-based analyses were carried out. Although we consider such analyses to be very important, as studies have shown that there can be differences in the response of races and ethnicities to a given treatment, at the same time, the available publications only provide data in this breakdown in a very limited number of cases, which makes it impossible to carry out a comprehensive analysis.

Research analyzing the safety, technical needs, accessibility and affordability of treatments would also be an important and urgently needed complement to the efficacy analyses detailed in this paper. In order to develop the most effective and feasible treatment protocol, it is necessary to consider all aspects together.

## Contributors

DSB, KL, and LFN, conceptualized the study with input from AR, TS, BE, and PH. DSB, KCSFN, TS, and VS carried out literature search, data collection and curation. NG and DSV completed the statistical analysis. DSB, TS, KL, and LFN led data interpretation with input from KCSFN, AR, NG, DSV, PP, BE, and PH. KL and LFN supervised the project with the cooperation of AR, BE, and PH. DSB wrote the first draft of the manuscript. KL and LFN equally contributed as the last authors. All authors edited the manuscript and approved the final version. All authors had full access to all data in the study and had final responsibility for the decision to submit it for publication. All authors certify that they have participated sufficiently in the work to assume public responsibility for the content, including participation in the concept, design, analysis, writing, and revision of the manuscript.

## Data sharing statement

All datasets used in this study can be found in the full-text publications included in the systematic review and meta-analysis. The detailed study protocol including the search key can be found in the PROSPERO registration (CRD42022371210) and the supplementary material of the publication.

## Declaration of interests

We declare no competing interests.
