# Supplementary material for: Comparative effectiveness of different therapies for Clostridioides difficile infection in adults: a systematic review and network meta-analysis of randomized controlled trials
Source: Lancet Reg Health Eur. 2025 Jan 5;49:101151. doi: 10.1016/j.lanepe.2024.101151 (PMC11846439; doi:10.1016/j.lanepe.2024.101151)
Supplement: Supplementary Appendix [file mmc1.docx]

THE LANCET REGIONAL HEALTH – EUROPE

**Supplementary appendix**

This appendix formed part of the original submission and has been peer reviewed.

We post it as supplied by the authors.

Supplement to: Bednárik SD, Földvári-Nagy KCS, Simon V, et al. Comparative effectiveness of different therapies in Clostridium difficile infection in adults: a systematic review and network meta-analysis of randomized controlled trials. *Lancet Regional Health – Europe* 2024; published online <https://doi.org/10.1016.j.lanepe.2024.101151>

Contents

[Supplementary Methods S1 ‒ Prospero registration 7](#_Toc180611219)

[Supplementary Methods S2 – Details of the systematic search 8](#_Toc180611220)

[Supplementary Methods S3 – Statistics 9](#_Toc180611221)

[*Table S1:* PRISMA-NMA extension checklist 10](#_Toc180611222)

[*Table S2:* Baseline Characteristics table – Cure rate, recurrency 16](#_Toc180611223)

[*Table S3:* Baseline Characteristics table – Prevention 22](#_Toc180611224)

[*Table S4:* Baseline Characteristics table – Systematic review 27](#_Toc180611225)

[*Table S5:* Treatments – Cure rate - overall 29](#_Toc180611226)

[*Table S6:* Treatments – Cure rate, recurrent cases 31](#_Toc180611227)

[*Table S7:* Treatments – Cure rate, non-recurrent cases 32](#_Toc180611228)

[*Table S8:* Treatments – Recurrence 34](#_Toc180611229)

[*Table S9:* Treatments – Prevention 36](#_Toc180611230)

[*Figure S1:* Analysis endpoints 38](#_Toc180611231)

[*Table S10:* Network summary table of the treatments for cure rates 39](#_Toc180611232)

[*Figure S2:* Network plot of possible treatments for cure rate 40](#_Toc180611233)

[*Table S11:* P-score table (SUCRA) of the treatments in case of cure rate 41](#_Toc180611234)

[*Figure S3:* Evidence plot of the treatments in case of cure rate 43](#_Toc180611235)

[*Figure S4:* Forest plot for results of consistency analysis for the treatments in case of cure rate 45](#_Toc180611236)

[*Figure S5:* Net heat plot of the treatments in case of cure rate 46](#_Toc180611237)

[*Figure S6:* Funnel plot of the treatments in case of cure rate 47](#_Toc180611238)

[*Table S12:* Network summary table of the treatments for cure rates in recurrent cases 48](#_Toc180611239)

[*Figure S7:* Network plot of possible treatments for cure rates in recurrent cases 49](#_Toc180611240)

[*Table S13:* P-score table (SUCRA) of the treatments in case of cure rate in the recurrent cases 50](#_Toc180611241)

[*Figure S8:* Evidence plot of the treatments in case of cure rate in the recurrent cases 52](#_Toc180611242)

[*Figure S9:* Forest plot for results of consistency analysis for the treatments in case of cure rate in the recurrent cases 54](#_Toc180611243)

[*Figure S10:* Net heat plot of the treatments in case of cure rate in the recurrent cases 55](#_Toc180611244)

[*Figure S11:* Funnel plot of the treatments in case of cure rate in the recurrent cases 56](#_Toc180611245)

[*Table S14:* Network summary table of the treatments for cure rates in non-recurrent cases 57](#_Toc180611246)

[*Figure S12:* Network plot of possible treatments for cure rates in non-recurrent cases 58](#_Toc180611247)

[*Table S15:* P-score table (SUCRA) of the treatments in case of cure rate in the non-recurrent cases 59](#_Toc180611248)

[*Figure S13:* Evidence plot of the treatments in case of cure rate in the non-recurrent cases 61](#_Toc180611249)

[*Figure S14:* Forest plot for results of consistency analysis for the treatments in case of cure rate in the non-recurrent cases 62](#_Toc180611250)

[*Figure S15:* Net heat plot of the treatments in case of cure rate in the non-recurrent cases 63](#_Toc180611251)

[*Figure S16:* Funnel plot of the treatments in case of cure rate in the non-recurrent cases 64](#_Toc180611252)

[*Table S16:* Network summary table of the treatments in recurrence 65](#_Toc180611253)

[*Figure S17:* Network plot of possible treatments in recurrence 66](#_Toc180611254)

[*Table S17:* P-score table (SUCRA) of the treatments in case of recurrence 67](#_Toc180611255)

[*Figure S18:* Evidence plot of the treatments in case of recurrence 68](#_Toc180611256)

[*Figure S19:* Forest plot for results of consistency analysis for the treatments in case of recurrence 69](#_Toc180611257)

[*Figure S20:* Net heat plot of the treatments in case of recurrence 70](#_Toc180611258)

[*Figure S21:* Funnel plot of the treatments in case of recurrence 71](#_Toc180611259)

[*Table S18:* Network summary table of the treatments for prevention 72](#_Toc180611260)

[*Figure S22:* Network plot of possible treatments for prevention 73](#_Toc180611261)

[*Table S19:* P-score table (SUCRA) of the treatments in case of prevention 74](#_Toc180611262)

[*Figure S23:* Evidence plot of the treatments in case of prevention 75](#_Toc180611263)

[*Figure S24:* Forest plot for results of consistency analysis for the treatments in case of prevention 76](#_Toc180611264)

[*Figure S25:* Net heat plot of the treatments in case of prevention 77](#_Toc180611265)

[*Figure S26:* Funnel plot of the treatments in case of prevention 78](#_Toc180611266)

[Supplementary Results S1 – Subgroup analyses of cure rate results by dose of vancomycin treatments 79](#_Toc180611267)

[*Table S20:* Network summary table of the treatments for cure rates, vancomycin dosages subgroup analysis 80](#_Toc180611268)

[*Figure S27:* Network plot of possible treatments for cure rate, vancomycin dosages subgroup analysis 81](#_Toc180611269)

[*Table S21:* P-score table (SUCRA) of the treatments in case of cure rate, vancomycin dosages subgroup analysis 82](#_Toc180611270)

[*Table S22:* League table of possible treatments in cure rate, vancomycin dosages subgroup analysis 83](#_Toc180611271)

[*Figure S28:* Evidence plot of the treatments in case of cure rate, vancomycin dosages subgroup analysis 86](#_Toc180611272)

[*Figure S29:* Forest plot for results of consistency analysis for the treatments in case of cure rate 88](#_Toc180611273)

[*Figure S30:* Net heat plot of the treatments in case of cure rate, vancomycin dosages subgroup analysis 89](#_Toc180611274)

[*Figure S31:* Funnel plot of the treatments in case of cure rate, vancomycin dosages subgroup analysis 90](#_Toc180611275)

[*Table S23:* Network summary table of the treatments for cure rates in recurrent cases, vancomycin dosages subgroup analysis 91](#_Toc180611276)

[*Figure S32:* Network plot of possible treatments for cure rates in recurrent cases, vancomycin dosages subgroup analysis 92](#_Toc180611277)

[*Table S24:* P-score table (SUCRA) of the treatments in case of cure rate in the recurrent cases, vancomycin dosages subgroup analysis 93](#_Toc180611278)

[*Table S25:* League table of possible treatments for cure rates in recurrent cases, vancomycin dosages subgroup analysis 94](#_Toc180611279)

[*Figure S33:* Evidence plot of the treatments in case of cure rate in the recurrent cases, vancomycin dosages subgroup analysis 96](#_Toc180611280)

[*Figure S34:* Forest plot for results of consistency analysis for the treatments in case of cure rate in the recurrent cases, vancomycin dosages subgroup analysis 98](#_Toc180611281)

[*Figure S35:* Net heat plot of the treatments in case of cure rate in the recurrent cases, vancomycin dosages subgroup analysis 99](#_Toc180611282)

[*Figure S36:* Funnel plot of the treatments in case of cure rate in the recurrent cases, vancomycin dosages subgroup analysis 100](#_Toc180611283)

[*Table S26:* Network summary table of the treatments for cure rates in non-recurrent cases, vancomycin dosages subgroup analysis 101](#_Toc180611284)

[*Figure S37:* Network plot of possible treatments for cure rates in non-recurrent cases, vancomycin dosages subgroup analysis 102](#_Toc180611285)

[*Table S27:* P-score table (SUCRA) of the treatments in case of cure rate in the non-recurrent cases, vancomycin dosages subgroup analysis 103](#_Toc180611286)

[*Table S28:* League table of possible treatments for cure rates in non-recurrent cases, vancomycin dosages subgroup analysis 104](#_Toc180611287)

[*Figure S38:* Evidence plot of the treatments in case of cure rate in the non-recurrent cases, vancomycin dosages subgroup analysis 105](#_Toc180611288)

[*Figure S39:* Forest plot for results of consistency analysis for the treatments in case of cure rate in the non-recurrent cases, vancomycin dosages subgroup analysis 106](#_Toc180611289)

[*Figure S40:* Funnel plot of the treatments in case of cure rate in the non-recurrent cases, vancomycin dosages subgroup analysis 107](#_Toc180611290)

[Supplementary Results S2 – Subgroup analysis of recurrence result by dose of vancomycin treatments 108](#_Toc180611291)

[*Table S29:* Network summary table of the treatments in recurrence, vancomycin dosages subgroup analysis 109](#_Toc180611292)

[*Figure S41:* Network plot of possible treatments in recurrence, vancomycin dosages subgroup analysis 110](#_Toc180611293)

[*Table S30:* P-score table (SUCRA) of the treatments in case of recurrence, vancomycin dosages subgroup analysis 111](#_Toc180611294)

[*Table S31:* League table of possible treatments in recurrence, vancomycin dosages subgroup analysis 112](#_Toc180611295)

[*Figure S42:* Evidence plot of the treatments in case of recurrence, vancomycin dosages subgroup analysis 115](#_Toc180611296)

[*Figure S43:* Forest plot for results of consistency analysis for the treatments in case of recurrence, vancomycin dosages subgroup analysis 117](#_Toc180611297)

[*Figure S44:* Net heat plot of the treatments in case of recurrence, vancomycin dosages subgroup analysis 118](#_Toc180611298)

[*Figure S45:* Funnel plot of the treatments in case of recurrence, vancomycin dosages subgroup analysis 119](#_Toc180611299)

[Supplementary Results S3 – Additional analysis of prevention therapies against the development of CDI 120](#_Toc180611300)

[*Figure S52:* Forest plots on FMT cure rates via colonoscopy or oral administration 127](#_Toc180611301)

[Supplementary Results S4 – Additional analysis of two armed FMT studies 128](#_Toc180611302)

[*Table S35:* Risk of Bias assessment 129](#_Toc180611303)

[*Table S36:* Grade: cure rate 132](#_Toc180611304)

[*Table S37:* Grade: recurrency 137](#_Toc180611305)

[*Table S38:* Grade: prevention 141](#_Toc180611306)

[Supplementary Discussion – Systematic review 143](#_Toc180611307)

[Supplementary References 144](#_Toc180611308)

# **Supplementary Methods S1 ‒ Prospero registration**

We previously submitted our study protocol to the **International prospective register of systematic reviews (PROSPERO) (CRD42022371210).**

The study protocol can be accessed via the following link:

<https://www.crd.york.ac.uk/prospero/display_record.php?RecordID=371210>

# **Supplementary Methods S2 – Details of the systematic search**

| **Date of search: 11 November, 2022 and 19 August 2024** | |
| --- | --- |
| **Databases** | |
|  | MEDLINE (via PubMed) (1294),  Embase (3840),  Cochrane Central Register of Controlled Trials (CENTRAL) (1412) |
| **Search key** | |
|  | (c.diff OR ((clostr* OR c.) AND difficile)) AND (random* OR blind* OR placebo OR rct) |
| **Search key expansions** | |
| **MEDLINE via PubMed** | |
|  | ("c.diff"[All Fields] OR (("clostr*"[All Fields] OR "c"[All Fields]) AND "difficile"[All Fields])) AND ("random*"[All Fields] OR "blind*"[All Fields] OR ("placeboes"[All Fields] OR "placebos"[MeSH Terms] OR "placebos"[All Fields] OR "placebo"[All Fields]) OR "rct"[All Fields]) |
| **Embase** | |
|  | (c.diff OR ((clostr* OR c.) AND difficile)) AND (random* OR blind* OR 'placebo'/exp OR placebo OR rct) |
| **Cochrane Central Register of Controlled Trials (CENTRAL)** | |
|  | (c.diff OR ((clostr* OR c.) AND difficile)) AND (random* OR blind* OR placebo OR rct |

# **Supplementary Methods S3 – Statistics**

For network meta-analysis inverse variance weighting method with DerSimonian-Laird estimator for τ^2^ was used.

For pooling the effect size in 2 level models, pooled RR, RD was calculated by the Mantel-Haenszel method.^1,2^ Exact Mantel-Haenszel method (without continuity correction) was used to handle zero cell counts (as recommended by Cooper and Sweeting).^3,4^ To estimate the heterogeneity variance measure (τ^2^), the Paule-Mandel method^5^ was used with the Q profile method for confidence interval (recommended in Veroniki et al.).^6^ We used a Hartung-Knapp adjustment.^7^

For pooling proportions (3 level meta-analysis), the logit transformation of the observed proportions was used. For the 0 events, as continuity correction we added 0.5 to 0 and the corresponding total case value. We used inverse variance weighting with restricted maximum likelihood method (estimating τ^2^) for pooling the proportions. For confidence interval calculation and prediction intervals we used a t-distribution method.

# ***Table S1:* PRISMA-NMA extension checklist**

| **Section/Topic** | **Item #** | **Checklist Item** | **Location where item is reported** |
| --- | --- | --- | --- |
| **TITLE** |  |  |  |
| Title | 1 | Identify the report as a systematic review *incorporating a network meta-analysis (or related form of meta-analysis).* | Title page |
|  |  |  |  |
| **ABSTRACT** |  |  |  |
| Structured summary | 2 | Provide a structured summary including, as applicable:  **Background:** main objectives  **Methods:** data sources; study eligibility criteria, participants, and interventions; study appraisal; and *synthesis methods, such as network meta-analysis.*  **Results:** number of studies and participants identified; summary estimates with corresponding confidence/credible intervals; *treatment rankings may also be discussed. Authors may choose to summarize pairwise comparisons against a chosen treatment included in their analyses for brevity.*  **Discussion/Conclusions:** limitations; conclusions and implications of findings.  **Other:** primary source of funding; systematic review registration number with registry name. | Abstract |
|  |  |  |  |
| **INTRODUCTION** |  |  |  |
| Rationale | 3 | Describe the rationale for the review in the context of what is already known*, including mention of why a network meta-analysis has been conducted.* | Introduction |
| Objectives | 4 | Provide an explicit statement of questions being addressed, with reference to participants, interventions, comparisons, outcomes, and study design (PICOS). | Introduction |
|  |  |  |  |
| **METHODS** |  |  |  |
| Protocol and registration | 5 | Indicate whether a review protocol exists and if and where it can be accessed (e.g., Web address); and, if available, provide registration information, including registration number. | Methods  Study design and selection criteria  Supplementary Methods S1 |
| Eligibility criteria | 6 | Specify study characteristics (e.g., PICOS, length of follow-up) and report characteristics (e.g., years considered, language, publication status) used as criteria for eligibility, giving rationale. *Clearly describe eligible treatments included in the treatment network, and note whether any have been clustered or merged into the same node (with justification).* | Methods  Study design and selection criteria |
| Information sources | 7 | Describe all information sources (e.g., databases with dates of coverage, contact with study authors to identify additional studies) in the search and date last searched. | Methods  Search strategy  Supplementary Methods S2 – Details of the systematic search |
| Search | 8 | Present full electronic search strategy for at least one database, including any limits used, such that it could be repeated. | Methods  Search strategy  Supplementary Methods S2 – Details of the systematic search |
| Study selection | 9 | State the process for selecting studies (i.e., screening, eligibility, included in systematic review, and, if applicable, included in the meta-analysis). | Methods  Study selection and data extraction |
| Data collection process | 10 | Describe method of data extraction from reports (e.g., piloted forms, independently, in duplicate) and any processes for obtaining and confirming data from investigators. | Methods  Study selection and data extraction |
| Data items | 11 | List and define all variables for which data were sought (e.g., PICOS, funding sources) and any assumptions and simplifications made. | Methods |
| **Geometry of the network** | **S1** | Describe methods used to explore the geometry of the treatment network under study and potential biases related to it. This should include how the evidence base has been graphically summarized for presentation, and what characteristics were compiled and used to describe the evidence base to readers. | Methods  Data analysis |
| Risk of bias within individual studies | 12 | Describe methods used for assessing risk of bias of individual studies (including specification of whether this was done at the study or outcome level), and how this information is to be used in any data synthesis. | Methods  Study selection and data extraction  Supplementary [Table S28: Risk of Bias assessment](#_Toc169361244) |
| Summary measures | 13 | State the principal summary measures (e.g., risk ratio, difference in means). *Also describe the use of additional summary measures assessed, such as treatment rankings and surface under the cumulative ranking curve (SUCRA) values, as well as modified approaches used to present summary findings from meta-analyses.* | Methods  Data analysis |
| Planned methods of analysis | 14 | Describe the methods of handling data and combining results of studies for each network meta-analysis. This should include, but not be limited to:   - *Handling of multi-arm trials;* - *Selection of variance structure;* - *Selection of prior distributions in Bayesian analyses; and* - *Assessment of model fit.* | Methods  Data analysis |
| **Assessment of Inconsistency** | **S2** | Describe the statistical methods used to evaluate the agreement of direct and indirect evidence in the treatment network(s) studied. Describe efforts taken to address its presence when found. | Methods |
| Risk of bias across studies | 15 | Specify any assessment of risk of bias that may affect the cumulative evidence (e.g., publication bias, selective reporting within studies). | Methods  Study selection and data extraction  Supplementary [Table S28: Risk of Bias assessment](#_Toc169361244) |
| Additional analyses | 16 | Describe methods of additional analyses if done, indicating which were pre-specified. This may include, but not be limited to, the following:   - Sensitivity or subgroup analyses; - Meta-regression analyses; - *Alternative formulations of the treatment network; and* - *Use of alternative prior distributions for Bayesian analyses (if applicable).* | Methods  Data analysis |
|  |  |  |  |
| **RESULTS** |  |  |  |
| Study selection | 17 | Give numbers of studies screened, assessed for eligibility, and included in the review, with reasons for exclusions at each stage, ideally with a flow diagram. | Results |
| **Presentation of network structure** | **S3** | Provide a network graph of the included studies to enable visualization of the geometry of the treatment network. | Results  Supplement |
| **Summary of network geometry** | **S4** | Provide a brief overview of characteristics of the treatment network. This may include commentary on the abundance of trials and randomized patients for the different interventions and pairwise comparisons in the network, gaps of evidence in the treatment network, and potential biases reflected by the network structure. | Results  Supplement |
| Study characteristics | 18 | For each study, present characteristics for which data were extracted (e.g., study size, PICOS, follow-up period) and provide the citations. | Table S2: Baseline Characteristics table Cure rate, recurrency  [Table S3: Baseline Characteristics table – Prevention](#_Toc169361210)  [Table S4: Baseline Characteristics table – Systematic review](#_Toc169361211) |
| Risk of bias within studies | 19 | Present data on risk of bias of each study and, if available, any outcome level assessment. | Methods  Study selection and data extraction  Supplementary [Table S28: Risk of Bias assessment](#_Toc169361244) |
| Results of individual studies | 20 | For all outcomes considered (benefits or harms), present, for each study: 1) simple summary data for each intervention group, and 2) effect estimates and confidence intervals. *Modified approaches may be needed to deal with information from larger networks.* | Results |
| Synthesis of results | 21 | Present results of each meta-analysis done, including confidence/credible intervals. *In larger networks, authors may focus on comparisons versus a particular comparator (e.g. placebo or standard care), with full findings presented in an appendix. League tables and forest plots may be considered to summarize pairwise comparisons.* If additional summary measures were explored (such as treatment rankings), these should also be presented. | Results |
| **Exploration for inconsistency** | **S5** | Describe results from investigations of inconsistency. This may include such information as measures of model fit to compare consistency and inconsistency models, *P* values from statistical tests, or summary of inconsistency estimates from different parts of the treatment network. | Results  Supplement |
| Risk of bias across studies | 22 | Present results of any assessment of risk of bias across studies for the evidence base being studied. | Methods  Study selection and data extraction  Supplementary [Table S28: Risk of Bias assessment](#_Toc169361244) |
| Results of additional analyses | 23 | Give results of additional analyses, if done (e.g., sensitivity or subgroup analyses, meta-regression analyses*, alternative network geometries studied, alternative choice of prior distributions for Bayesian analyses,* and so forth). | Results |
|  |  |  |  |
| **DISCUSSION** |  |  |  |
| Summary of evidence | 24 | Summarize the main findings, including the strength of evidence for each main outcome; consider their relevance to key groups (e.g., healthcare providers, users, and policy-makers). | Discussion |
| Limitations | 25 | Discuss limitations at study and outcome level (e.g., risk of bias), and at review level (e.g., incomplete retrieval of identified research, reporting bias). *Comment on the validity of the assumptions, such as transitivity and consistency. Comment on any concerns regarding network geometry (e.g., avoidance of certain comparisons).* | Discussion |
| Conclusions | 26 | Provide a general interpretation of the results in the context of other evidence, and implications for future research. | Discussion |
|  |  |  |  |
| **FUNDING** |  |  |  |
| Funding | 27 | Describe sources of funding for the systematic review and other support (e.g., supply of data); role of funders for the systematic review. This should also include information regarding whether funding has been received from manufacturers of treatments in the network and/or whether some of the authors are content experts with professional conflicts of interest that could affect use of treatments in the network. | Declaration of interests |

PICOS = population, intervention, comparators, outcomes, study design.

* Text in italics indicateS wording specific to reporting of network meta-analyses that has been added to guidance from the PRISMA statement.

# ***Table S2:* Baseline Characteristics table – Cure rate, recurrency**

| **Article (First author, year)** | **Study design**  **Centers,**  **Location** | **Patients number** | **Treatments** | **Duration** | **Follow up** | **Male**  **%** | **Mean age, y** | **Severity** | **Proportion of patients with severe Clostridioides difficile infection (%)** |
| --- | --- | --- | --- | --- | --- | --- | --- | --- | --- |
| Allegreti et al, 2024 | A multicenter randomized placebo-controlled trial.  USA | 30  31 | Single colonoscopic FMT+single bezlotoxumab infusion  Placebo infusion before or during the FMT | Once | 8 weeks | 53  55 | 38·5  34 | NA | NA |
| Baunwall et al., 2022 | Randomised, double-blind, placebo-controlled trial  Single centre  Denmark | 21  21 | Encapsulated FMT (vancomycin pretreatment 125 mg four times daily for 10 days)  Placebo (vancomycin pretreatment 125 mg four times daily for 10 days) | 10 days | 8 weeks | 29  24 | 58  60 | Mild to moderate, severe too | 76 |
| Boix et al., 2017 | Randomized, DoubleBlind, Active-Controlled Trial, Phase 3 Multicentre  USA, Europe, Middle East | 290  280 | Surotomycin 250 mg twice daily  Oral vancomycin 125 mg four times daily | 10 days | 40 to 50 days | 59·7  59·3 | 64·0  64·5 | Mild to moderate, severe too | 34 |
| Camacho-Ortiz et al., 2017 | Open, per-protocol, two-arm pilot trial  Single centre  Mexico | 9  10 | Fecal donor-unrelated donor mix (FMTFURM) transplantation, Feces were diluted with 500 mL of sterile saline (0.9%)  Oral Vancomycin 250 mg 4 times | 10-14 days | 30 days, 1 year | 57·1  66·7 | 39·7  46·7 | NA | NA |
| Cammarota et al., 2015 | Randomised clinical trial, open-label  Single centre  Italy | 20  19 | Vancomycin+FMT. short regimen of vancomycin, (125 mg by mouth four times a day for 3 days, Feces were diluted with 500 mL of sterile saline (0.9%)  Vancomycin 125 mg 4 times daily | 10 days | 10 weeks | 40  42 | 71  75 | Mild, moderate and severe too | NA |
| Cornely et al., 2012 | Randomised, double-blind, non-inferiority trial  Multicentre  International | 270  265 | Fidaxomicin 200 mg every 12 hours  Vancomycin 125 mg every 6 hours | 10 days | 28 days | 41·3  37 | 64·3  62·5 | severe/not severe | 24 |
| Daley et al., 2017 | Randomized, double-blind, active-controlled investigation, consisting of two treatment arms  Multicentre  North and Sout America, Asia-Pacifiv | 285  292 | Oral surotomycin 250 mg twice daily  Oral vancomycin 125 mg 4 times | 10 days | 30-40 days | 39·3  33·6 | 57·6  56·5 | Mild, moderate and severe too | 67·4 |
| De Lalla et al., 1992 | Randomized, prospective study  Single centre  Italy | 27  20 | Teicoplanin 100 mg twice daily  Oral vancomycin 500 mg four time daily | 10 days | 1 month | 30·8  30 | 48  47 | NA | NA |
| Dudley et al., 1986 | A Randomized Double-blind Trial  Single centre  USA | 15  15 | Bacitracin 25 000 units 4 daily  Vancomycin 500 mg 4 daily | 10 days | 60 days | 26·7  53·3 | 71  66 | Moderate to severe symptoms | NA |
| Feuerstadt et al., 2022 | Randomized, double-blind, placebo-controlled trial, phase 3.  Multicentre  USA,Canada | 89  93 | SER 109 Approximately 3×10^7^ spore colony-forming units.  Placebo | 3 consecutive days | 8 weeks | 33  47 | 65·6±16·5  65·5±16·7 | NA | NA |
| Garza-Gonzalez et al., 2019 | Randomized, double blinded, comparative two-arm pilot study Multicentre (2)  Mexico | 13  8 | FMT Per os every 12 hours for a total of 4 doses (a total of 60 capsules)  FMTenriched with 3 species of *Lactobacillus* (FMT-L) | 0,3,7,28th day | 90 days | 38,5  50 | 56·8  62·4 | NA | NA |
| Guery et al., 2017 | Randomised, controlled, parallel, superiority, open-label trial, phase 3b/4  Multicentre  Europe | 177  179 | Extended-pulsed fidaxomicin 200 mg oral tablets, twice daily on days 1–5, then once daily on alternate days on days 7–25  Vancomycin 125 mg four times daily for 10 days | 25 days | 90 days | 40  44 | 75·1  61·6 | Severe/non severe | 27 |
| Hota et al., 2017 | RCT single-site, open-label, a phase 2/3  Single centre  Canada | 12  16 | Vancomycin taper followed by a taper over 4 weeks: vancomycin 125 mg orally every 12 hours for 1 week; then, vancomycin 125 mg orally every 24 hours for 1 week; then, vancomycin 125 mg orally every second day for 1 week; then, vancomycin 125 mg orally every third day for 1 week  14 days of oral vancomycin 125 mg every 6 hours pretreatment + FMT (enema) | 4 weeks | 120 days | 33,3  31,2 | 50  50 | NA | NA |
| Housman et al., 2016 | Randomized prospective, open-label, trial  Single centre  USA | 16  18 | Vancomycin orally 125 mg 4 times daily for 10 days  Fidaxomicin 200 mg twice a day | 10 days | On 38th day (10+28) end of treatment (day 38) | 43·8  39 | 66±15  69±15 | NA | NA |
| Hvas et al., 2019 | Randomized, active-comparator, open-label clinical trial  Single centre  Denmark | 24  24  16 | FMT preceded by 4–10 days of vancomycin 125 mg 4 times daily  Fidaxomicin 200 mg twice a day for ten days  Oral vancomycin 125 mg 4 times daily | 4-10 days | 8 weeks | 17  46  31 | 68  64  72 | NA | NA |
| Ianiro et al., 2018 | Randomised open-label, clinical trial  Single centre  Italy | 28  28 | FMT-S + 3-day pre-treatment with vancomycin (250 mg by mouth four times a day)  FMT-M + 3-day pre-treatment with vancomycin (250 mg by mouth four times a day) | 14-day vancomycin course; including single/multiple (at least two) faecal infusions | 8 weeks | 36  25 | 75  74 | Severe | 100 |
| Jiang et al., 2017 | Randomised clinical trial  Single centre  USA | 25  24  23 | Fresh microbiota stool samples ≥50 g, diluted in 0.85% NaCl (1:10) with a total volume of 1500 mL  Frozen microbiota  Lyophilised microbiota Lyophilised | Once | 5 months | 16  25  43·5 | 75  62·5  63 | NA | NA |
| Jiang et al., 2018 | Randomized clinical trial  Single centre  USA | 34  31 | FMT frozen enema product Frozen FMT from 100 g of donor feces by enema  Lyophilized FMT-oral product  Receive encapsulated lyophilized fecal microbiota from 100–200 g of donor feces | Once | 90 days | 26  32 | 63  67 | NA | NA |
| Johnson et al., 1992 | Randomized, nonblinded placebo-controlled trial  Single centre  USA | 10  10  10 | Oral vancomycin 125 mg four times daily for 10 days  Oral metronidazole 500 mg twice daily  Placebo 3 times daily | 10 days | 70 days | NA | 65  69  72 | NA | NA |
| Johnson et al., 2014 | Randomized, Controlled trials  Multicentre  International | 563  266  289 | Tolevamer 9 g (loading dose) followed by 3 g every 8 hours for 14 days  Vancomycin 125 mg every 6 hours for 10 days  Metronidazole 375 mgevery 6 hours for 10 days | For 14 days | 4 weeks | 37·3  44·2  52 | 65·5  64·5  64·5 | Mild/moderate/severe | 29 |
| Kao et al., 2017 | Randomized, noninferiority trial  Multicentre  Canada | 57  59 | FMT by oral capsule Swallowed 40 capsules under direct observation (vancomycin pretreatment 125 mg four times daily for 10 days)  FMT by Colonoscopy Received 360mL of fecal slurry in the cecum (vancomycin pretreatment 125 mg four times daily for 10 days) | Once | 12 weeks | 24·6  39 | 58·7  57·4 | Mild | 0 |
| Lagrotteria et al., 2006 | Randomized, single-blinded, prospective study  Multicentre  Canada | 20  19 | Metronidazole 500 mg 3 times daily orally for 10 days  Metronidazole+rifampin 500 mg of metronidazole orally 3 times per day plus 300 mg of rifampin orally 2 times per day for 10 days | 10 days | 30 days | 45  36·8 | 67·5  70·5 | NA | NA |
| Lee et al., 2016 | Randomized, double-blind, noninferiority trial  Multicentre  Canada | 114  118 | Frozen FMT group Patients received 50 mL of frozen FMT by enema  Fresh FMT group Patients received 50 mL of fresh FMT by enema | Once, or twice who needed | 13 weeks | 50  23·1 | 72·2  72·9 | Mild/moderate/severe | 6 |
| Louie et al., 2011 | Phase 3 randomized clinical trial  Multicentre  International | 302  327 | Fidaxomicin 200 mg oral capsule twice daily for 10 days  Vancomycin 125 mg 4 times daily for 10 days | For 10 days | 28 days | 42·9  45·3 | 60·3  62·9 | Mild/moderate/severe | 39 |
| McFarland et al., 1994 | Randomized, double-blind, placebo-controlled, parallel-group intervention study  Multicentre  USA | 57  67 | *Saccharomyces boulardii* with an antibiotics (vancomycin hydrochloride or metronidazole). *S. boulardii* i (1 g/d for 4 weeks)  Placebo+antibiotics | For 4 weeks | 4 weeks | 26·3  20·9 | 56·8  67 | severe |  |
| Mikamo et al., 2018 | Double-blind, parallel-group study  Multicentre  Japan | 104  108 | Fidaxomicin 200 mg twice daily orally Vancomycin 125 mg four-times daily, orally | 10 days | 28 days | 46·2  50 | 74  75 | Mild/moderate/severe | 22·4 |
| Musher et al., 2006 | Randomized, double-blind, prospective study  Single centre  USA | 44  49  49 | Metronidazole 250 mg every 6 h  Nitazoxanide 500 mg every 12 hours  Nitazoxanide 500 mg every 12 hours | 10 days  7 days  10 days | 31 days | 73  76  80 | 66·8  67·3  69·2 | Mild/moderate/severe | NA |
| Musher et al., 2009 | Randomized, Double-Blind Study  Single centre  USA | 27  23 | Vancomycin 125 mg every 6 h for 10 days  Nitazoxanide 500 mg twice daily | 10 days | 31 days | 67  64 | 65·7  59·6 | Mild/moderate/severe | 41 |
| Okhuysen et al, 2024 | A Randomized, Double-Blind, Phase 3 Safety and Efficacy Study  International | 370  375 | Ridinilazole 200 mg twice daily  Vancomycin 125 mg 4 times daily | 10 days | 30 days | 43·5  39·5 | 61  63 | Mild/moderate/severe | 24·6 |
| Rao et al, 2024 | Randomized, controlled, open-label trial  USA | 64  54 | Fidaxomicin 200 mg twice daily  Vancomycin 125 mg orally 4 times daily | 10 days | 30 days | 45·9  50 | NA | Mild/moderate/severe | NA |
| Rode et al., 2021 | Randomised controlled trial, open-label 3-arm  Multicentre  Denmark | 34  33  31 | Fecal microbiota transplantation 1-3 infusions within 14 days based on predefined clinical criteria (vancomycin pretreatment 125 mg four times daily for 10 days)  Rectal bacteriotherapy Three infusions on 3 consecutive days (vancomycin pretreatment 125 mg four times daily for 10 days)  Oral vancomycin 125 mg g four times daily | 14 days | 180 days | 41  42  55 | 75  67  76 | Mild/moderate/severe | NA |
| Teasley et al., 1983 | Prospective randomized trial  Single centre  USA | 52  42 | Vancomycin 500 mg 4 times per day for 10 days  Metronidazole 250 mg 4 times daily for 10 days | For 10 days | 21 days | NA | 65·5  63·6 | NA | NA |
| Thabit et al., 2016 | Open-label, randomized controlled study  Single centre  USA | 7  5 | Fidaxomicin 200 mg every 12 h orally for 10 days  Vancomycin 125 mg every 6 hour for 10 days | For 10 days | 38 days | 57  40 | 68  73 | NA | NA |
| Young et al., 1985 | Randomized double-blind study  Multicentre  Australia | 21  21 | Bacitracin 80 000 U/day  Vancomycin 500 mg/day | 7 days | 1 month | NA | 63,9  60·7 | NA | NA |
| Youngster et al., 2014 | A Randomized, Open-Label, Controlled Pilot Study  Single centre  USA | 10  10 | FMT by colonoscopic This amount of fecal material was further diluted to 250 cc  FMT by nasogastric tube 90 cc of inoculum was administered | Once | 8 weeks | 40  50 | 50·4  58·6 | Mild/severe | NA |
| van Nood et al., 2013 | Randomized, open-label, controlled trial  Single centre  Netherlands | 16  13  13 | Donor-feces infusion an initial vancomycin regimen (500 mg orally four times per day for 4 days), followed by bowel lavage and subsequent infusion of a solution of donor feces through a nasoduodenal tube  Vancomycin 500 mg orally four times per day  Vancomycin and bowel lavage | 14 days | 10 weeks | 50  46  77 | 73  66  69 | Mild to moderate, severe too | NA |
| Wenisch et al., 1996 | Randomized study, prospective  Single centre  Austria | 29  31  31  28 | Fusidic acid 500 mg t.i.d  Metronidazole 500 mg t.i.d 10 days  Vancomycin 500 mg t.i.d 10 days  Teicoplanin 400 mg b.i.d, for 10 days | 10 days | 30 days | 51·7  51·6  54·8  50 | 43  44  38  45 | NA | NA |
| Wullt et al., 2004 | Randomized controlled, double-blind, prospective trial  Single centre  Sweden | 59  55 | Fusidic acid 250 mg orally three times daily for 7 days  Metronidazole 400 mg orally three times daily for 7 days | For 7 days | 40 days | 30·8  40 | 58  58·4 | NA | NA |
| Zar et al., 2007 | Randomized, double-blind, placebo-controlled, prospective trial  Single centre  USA | 71  79 | Vancomycin 125 mg 4 times per day for 10 days Metronidazole 250 mg 4 times daily for 10 days | For 10 days | 21 days | 49·4  54·4 | 59·4  57·7 | Mild/severe | 48 |

# ***Table S3:* Baseline Characteristics table – Prevention**

| **Article (First author, year)** | **Study design How many centers Location** | **Patients number (Intervention/Comparison(s))** | **Treatment(s), Dose(s) Control, Dose** | **Duration of treatment** | **Follow up** | **Men %** | **Mean age** | **Severity** |
| --- | --- | --- | --- | --- | --- | --- | --- | --- |
| Allen et al., 2013 | Randomised, double-blind, placebo-controlled, two-group trial Multicentre UK | 1493  1488 | *Lactobacillus* and *Bifidobacterium* 6 × 10¹⁰ live bacteria: two strains of *Lactobacillus* *acidophilus* and two strains of *Bifidobacterium bifidum, B. lactis* Placebo | 21 days | 8 weeks | 52·9  46·2 | 77·2  77 | N/A |
| Beausoleil et al., 2007 | Randomized, double-blind, prospective placebo-controlled study Single centre Canada | 44  45 | *Lactobacilli*-fermented milk at least 50×10^9^ colony forming units of *Lactobacillus acidophilus* CL1285 and L casei (Bio-K+ CL1285, Bio-K+ International Inc, Canada). 49 g (one-half of a container) followed by 98 g Placebo (a lactoserum devoid of microorganisms) | 2 days and the other till the end of the antibiotic treatment | 21 days | 45·5  51·1 | 68·8  72·9 | N/A |
| Can et al., 2006 | Double-blind controlled study Single centre Turkey | 73  78 | *Saccharomyces boulardii* 5×10^9^ *S. boulardii* cells Placebo | 10 days | 4 weeks | 95  89 | N/A | N/A |
| de Bruyn et al., 2021 | Randomised, controlled trial, observer-blind, phase 3  Multicentre 27 countries | 6173  3085 | *C. difficile* toxoid vaccine  *C difficile* toxoid vaccine candidate group Placebo | Intramuscular injection on days 0, 7, and 30. | 30 days | 57·6  58·2 | 65·9  65·8 | Severe |
| Erhardt et al., 2016 | A Randomized, Double-Masked, Placebo-Controlled Trial  Multicentre Germany | 246  231 | *Saccharomyces boulardii* Group 250 mg twice daily Placebo | Till the end of the antibiotic therapy+7 days. Max 8 weeks. | 8 weeks | 56·9  55·8 | 60·1  56·5 | N/A |
| Garey et al., 2011 | Randomized, double-blind, placebo-controlled pilot study Single centre USA | 33  35 | Rifaximin 400 mg three times daily Placebo | 20 days | 3 months | 52  49 | 58  64 | Severe too |
| Hickson et al., 2007 | Randomised double blind placebo-controlled trial Single centre UK | 69  66 | Probiotic; *Lactobacillus* *casei* DN-114 001 (L casei imunitass) (1.0×10^8^ colony forming units/ml), *Streptococcus thermophilus* (1.0×10^8^ cfu/ml), and *L. bulgaricus* (1.0×10^7^ cfu/ml). Placebo | One week | 4 weeks | 43  48 | 74  74 | Severe |
| Johnson et al., 2020 | Randomized, open-label prospective study Single centre USA | 50  50 | Oral Vancomycin Prophylaxis Oral vancomycin, 125 mg once daily for 5 days No prophylaxis | 5 days | 90 days | 40  46 | 75·06  73·32 | Severe too |
| Khanna et al., 2022 | A randomized, double-blind, placebo-controlled, phase III Multicentre USA and Canada | 180  87 | RBX2660 RBX2660-microbiota suspension rectally Placebo | Once | 6 months | 31·7  31 | 64  60 | mild/moderate/severe |
| Laffan et al., 2012 | Randomized double-blind study Single centre USA | 9  13 | Lactoferrin by gastrostomy tube, lactoferrin (5 mg/mL) Placebo | 56 days | 56 days | 55·6  23·1 | 62·1  62·4 | Severe |
| Lewis et al., 1998 | RCT Single UK | 33  36 | *Saccharomyces boulardii* *S. boulardii* 113 mg twice daily Placebo, twice daily | As long as they received antibiotics | 7 weeks | N/A | N/A | N/A |
| Lewis et al., 2005 | A Randomized, Controlled Study Single centre UK | 72  70 | Oligofructose oligofructose (12 g/day) Placebo placebo (sucrose, 12 g/day) | 30 days | 60 days | 43  40 | N/A | N/A |
| Major et al., 2018 | Randomised, placebo controlled, multisite, parallel group trial Multicentre UK | 77  74 | Rifaximin rifaximin 400mg three times a day for 2 weeks, reduced to 200mg three times a day for a further 2weeks Placebo | 4 weeks | 12 weeks | 49  39 | 72·2  71·5 | Mild/severe |
| Morrow et al., 2010 | Randomized, Blinded, Controlled Trial Single centre USA | 73  73 | *Lactobacillus* GG Received 2x 10^9^ colony-forming units (cfu) of *Lactobacillus rhamnosus* GG on a twice-daily Placebo | Until extubation | N/A | 58·9  58·9 | 52·5  54·6 | N/A |
| Mullane et al., 2019 | Double-blind study Multicentre USA | 301  299 | Fidaxomicin Once-daily oral fidaxomicin (200 mg) matching placebo | 2 days of fluoroquinolone prophylaxis and continued until 7 days for up to 40 days | 60 days | 58·5  65·6 | 55·1  55·1 | N/A |
| Nord et al., 1997 | Randomized double-blind parallel group study Single centre Sweden | 11  12 | Lyophilized *Lactobacillus acidophilus,* *Bifidobacterium* *bifidum, Lactobacillus delbrueckii subsp. bulgaricus and Streptococcus salivarius subsp. thermophilus* capsules+ oral clindamycin  placebo, 400 mg three times daily | 7 days and 14 days | 28 days | 27  33·3 | 29·5  29·5 | Mild/moderate |
| Plummer et al., 2004 | Double-blind, placebo-controlled study Single centre UK | 69  69 | Probiotic-beside antibiotic Probiotic product 2×10^10^ cfu *Lactobacillus acidophilus* and *Bifidobacterium bifidum*/capsule  Placebo | For 20 days | 20 days | N/A | N/A | N/A |
| Pozzoni et al., 2012 | Randomized, Double-Blind, Placebo-Controlled Trial Single centre Italy | 141  134 | A probiotic capsule 5 × 10^9^ colony-forming units of *Saccharomyces boulardii* Placebo | 7 days | 12 weeks | 49·6  50 | 79·9 (overall mean) | Severe |
| Prasoon et al., 2022 | Randomized, double blinded study Single centre India | 60  60 | Probiotic  *Lactobacillus acidophilus*, *Lactobacillus rhamnosus*, *Bifidobacterium longum*, and *Saccharomyces boulardii* twice daily and the standard preventive strategies of VAP  Placebo | Until extubation | N/A | 40  63·3 | 55·5  56·6 | N/A |
| Rajkumar et al., 2020 | Randomised, double-blind, placebo-controlled trial Multicentre UK | 549  577 | *Lactobacillus casei* DN114001 (combined as a drink with two regular yoghurt bacterial strains) Placebo | Once | 2 weeks | 48·8  49·2 | 73·7  73·5 | Mild to moderate, severe too |
| Rauseo et al., 2022 | Prospective, randomized controlled trial, double-blinded. Single centre  USA | 44  44 | 1 capsule containing 1×10^10^ cells of *Lactobacillus rhamnosus* GG (LGG) 1 capsule containing 1×10^10^ cells of *Lactobacillus rhamnosus* GG (LGG) twice daily Placebo, twice daily | 3 days | 60 days | 50  54 | 58  60 | N/A |
| Sadahiro et al., 2014 | Prospective randomized trial Single centre Japan | 100  99  95 | *Bifidobacteria*-treated group BFT, *Bifidobacteria* tablet (*Bifidobacterium bifidum*) Oral antibiotics-treated (kanamycin sulfate and metronidazole) group 0,5g+0,5g oral antibiotics+bifidobacteria Control group | 5–15 days | 4 weeks | 49  56·6  53·7 | 67  67  66 | N/A |
| Saviano et al, 2024 | Single-center, randomized, open-label study  Italy | 56  57 | Limosilactobacillus reuteri LMG P-27481 and Lacticaseibacillus rhamnosus GG ATCC 53103  Oral stick 1.4 g (2 × 1010CFU for stick) × 2/day  Placebo | 4 weeks | 4 weeks | 34  52·6 | 67·16  71·9 | N/A |
| Surawicz et al., 1989 | Prospective double-blind controlled study Single centre USA | 32  16 | *Saccharomyces boulardii* Received 1 g of lyophilized *S. boulardii* per day Placebo | 14 days | 18 days | 68·9  66·7 | 48·6  45·4 | Severe |
| Surawicz et al., 2000 | National double-blind, placebo-controlled trial of adult patients with recurrent CDI Multicentre USA | 18  14 | High-dose vancomycin and *Saccharomyces boulardii* 1 g/day for 28 days High-dose vancomycin and placebo | For 28 days | 4 weeks | 44·4  35·7 | 61·8  60·9 | Severe |
| Thomas et al., 2001 | Randomized, double-blind, placebo-controlled, prospective trial Single  USA | 152  150 | *Lactobacillus* GG 1 capsule twice daily for 14 days Placebo | For 14 days | 21 days | 45·7  53·6 | 61·5  55·8 | Severe |
| Tobar et al., 2018 | Prospective, randomized controlled trial  Single centre Mexico | 41  55 | Metronidazol 500 mg orally, every 8 hours for 7 days Placebo | 7 days | N/A | 51·2  52·7 | 65·7  63·5 | Mild to moderate, severe too |
| Wilcox et al., 2017 | Randomized, two double-blind, placebo-controlled, phase 3 trials Multicentre 30 countries | 773  781  232  773 | Actoxumab plus Bezlotoxumab, 10 mg per kilogram each Bezlotoxumab, 10 mg per kilogram each Actoxumab, 10 mg per kilogram each Placebo, 0.9% saline | A single, 60-minute intravenous infusion | 12 weeks | 45·3  43·4  44  41·9 | N/A | Severe |
| Wong et al., 2014 | Randomized controlled trial Single UK | 79  85 | Probiotic (LcS od) Probiotic drink (Yakult Lightw: 65 ml) containing a minimum of 6·5 £ 10^9^ colony-forming units (CFU) LcS/ bottle and skimmed milk Control No LcS/routine care | When the antibiotic course was finished, a further 7 d of study drink was prescribed. | 30 days | 81·6  84·1 | 52·5  51 | N/A |

# ***Table S4:* Baseline Characteristics table – Systematic review**

| **Article (First author, year)** | **Study design How many centers Location** | **Patients number (Intervention/Comparison(s))** | **Treatment(s), Dose(s) Control, Dose** | **Duration of treatment** | **Follow up** | **Men %** | **Mean age** | **Severity** |
| --- | --- | --- | --- | --- | --- | --- | --- | --- |
| Cleary et al., 1998 | Using simple randomization, prospective double-blind study Single centre USA | 42  40 | Three doses of neomycin and erythromycin 1 g/dose twice daily Neomycin and metronidazole 1 g/dose twice daily | Three doses | 90 days | 49  50 | 67·6  62·1 | N/A |
| Gao et al., 2010 | Randomized, double-blind, placebo-controlled dose-ranging study Single centre China | 86  85  84 | Two probiotic capsules per day Probiotic capsule contained 50 billion c.f.u. of live organisms (*Lactobacillus acidophilus* CL1285® + *Lactobacillus casei* LBC80R®Bio-K +CL1285) One probiotic capsules per day Probiotic capsule contained 50 billion c.f.u. (*Lactobacillus acidophilus* CL1285® + *Lactobacillus casei* LBC80R® ) + one placebo Placebo, Two placebo | At least 3 days but no more than 14 days | 21 days | 50  51  54 | 60  60  60 | N/A |
| Mattila et al., 2008 | Controlled, double-blind, randomized, parallel-group, multicentre, comparative trial. Multicentre Finland | 18  20 | CDIW 200 ml t.i.d. for 14 days Metronidazole, 400 mg t.i.d for 14 days | 14 days | 70 days | 38·8  55 | 56·4  65·7 | N/A |
| Wistrom et al., 1994 | Randomized, double-blind Single centre Sweden | 23  24 | teicoplanin, 100 mg twice daily for 7 days teicoplanin, 50 mg 4 times daily for 3 days, followed by teicoplanin 100 mg twice daily for 4 days | for 7 days | 4 weeks | 48·9 | 65  (overall mean) | Severe |
| Wullt et al., 2003 | A Double-blind, Placebo-controlled Trial Multicentre Sweden | 11  9 | Metronidazole and *Lactobacillus plantarum* 299v; Receive metronidazole (400 mg t.i.d.) orally combination with either a fruit drink containing oats fermented with *L. plantarum* 299v (5/10^10^ cfu/d) Metronidazole and placebo, 400 mg t.i.d. | For 10 days | 70-75 days | 8·3  0 | 64·5  62·9 | Mild to moderate, severe too |

# ***Table S5:* Treatments – Cure rate - overall**

| **Article (First author, year)** | **Treatment 1** | **Treatment 2** | **Treatment 3** | **Treatment 4** | **Patients number** |
| --- | --- | --- | --- | --- | --- |
| Dudley et al., 1986 | Bacitracin | Vancomycin |  |  | 15; 15 |
| Young et al., 1985 | Bacitracin | Vancomycin |  |  | 21; 21 |
| Hvas et al., 2019 | Fidaxomicin | FMT | Vancomycin |  | 24; 24; 16 |
| Cornely et al., 2012 | Fidaxomicin | Vancomycin |  |  | 270; 265 |
| Guery et al., 2018 | Fidaxomicin | Vancomycin |  |  | 177; 179 |
| Housman et al., 2016 | Fidaxomicin | Vancomycin |  |  | 18; 16 |
| Louie et al., 2011 | Fidaxomicin | Vancomycin |  |  | 302; 327 |
| Mikamo et al., 2018 | Fidaxomicin | Vancomycin |  |  | 104; 108 |
| Rao et al, 2024 | Fidaxomicin | Vancomycin |  |  | 64; 54 |
| Thabit et al., 2016 | Fidaxomicin | Vancomycin |  |  | 7; 5 |
| Hvas et al., 2019 | FMT | Fidaxomicin | Vancomycin |  | 24; 24; 16 |
| Baunwall et al., 2022 | FMT | Placebo |  |  | 21; 21 |
| Rode et al., 2021 | FMT | Rectal bacteriotherapy | Vancomycin |  | 34; 33; 31 |
| Camacho-Ortiz et al., 2017 | FMT | Vancomycin |  |  | 9; 10 |
| Cammarota et al., 2015 | FMT | Vancomycin |  |  | 20; 19 |
| van Nood et al., 2013 | FMT | Vancomycin | Vancomycin + bowel lavage |  | 16; 13; 13 |
| Wullt et al., 2004 | Fusidic acid | Metronidazole |  |  | 59; 55 |
| Wenisch et al., 1996 | Fusidic acid | Metronidazole | Teicoplanin | Vancomycin | 29; 31; 28; 31 |
| Wullt et al., 2004 | Metronidazole | Fusidic acid |  |  | 55; 59 |
| Wenisch et al., 1996 | Metronidazole | Fusidic acid | Teicoplanin | Vancomycin | 31; 29; 28; 31 |
| Lagrotteria et al., 2006 | Metronidazole | Metronidazole+rifampin |  |  | 20; 19 |
| Musher et al., 2006 | Metronidazole | Nitazoxanide |  |  | 44; 49 |
| Johnson et al., 1992 | Metronidazole | Placebo | Vancomycin |  | 10; 10; 10 |
| Johnson et al., 2014 | Metronidazole | Tolevamer | Vancomycin |  | 289; 563; 266 |
| Teasley et al., 1983 | Metronidazole | Vancomycin |  |  | 42; 52 |
| Zar et al., 2007 | Metronidazole | Vancomycin |  |  | 79; 71 |
| Lagrotteria et al., 2006 | Metronidazole+rifampin | Metronidazole |  |  | 19; 20 |
| Musher et al., 2006 | Nitazoxanide | Metronidazole |  |  | 49; 44 |
| Musher et al., 2009 | Nitazoxanide | Vancomycin |  |  | 23; 27 |
| Baunwall et al., 2022 | Placebo | FMT (encapsulated) |  |  | 21; 21 |
| Johnson et al., 1992 | Placebo | Metronidazole | Vancomycin |  | 10; 10; 10 |
| Feuerstadt et al., 2022 | Placebo | SER 109 |  |  | 93; 89 |
| Rode et al., 2021 | Rectal bacteriotherapy | FMT | Vancomycin |  | 33; 34; 31 |
| Okhuysen et al, 2024 | Ridinilazole | Vancomycin |  |  | 370; 375 |
| Feuerstadt et al., 2022 | SER 109 | Placebo |  |  | 89; 93 |
| Boix et al., 2017 | Surotomycin | Vancomycin |  |  | 290; 280 |
| Daley et al., 2017 | Surotomycin | Vancomycin |  |  | 285; 292 |
| Wenisch et al., 1996 | Teicoplanin | Fusidic acid | Metronidazole | Vancomycin | 28; 29; 31; 31 |
| De Lalla et al., 1992 | Teicoplanin | Vancomycin |  |  | 27; 20 |
| Johnson et al., 2014 | Tolevamer | Metronidazole | Vancomycin |  | 563; 289; 266 |
| Dudley et al., 1986 | Vancomycin | Bacitracin |  |  | 15; 15 |
| Young et al., 1985 | Vancomycin | Bacitracin |  |  | 21; 21 |
| Cornely et al., 2012 | Vancomycin | Fidaxomicin |  |  | 265; 270 |
| Guery et al., 2018 | Vancomycin | Fidaxomicin |  |  | 179; 177 |
| Housman et al., 2016 | Vancomycin | Fidaxomicn |  |  | 16; 18 |
| Louie et al., 2011 | Vancomycin | Fidaxomicin |  |  | 327; 302 |
| Mikamo et al., 2018 | Vancomycin | Fidaxomicin |  |  | 108; 104 |
| Rao et al, 2024 | Vancomycin | Fidaxomicin |  |  | 54; 64 |
| Thabit et al., 2016 | Vancomycin | Fidaxomicin |  |  | 5; 7 |
| Hvas et al., 2019 | Vancomycin | Fidaxomicn | FMT |  | 16; 24; 24 |
| Camacho-Ortiz et al., 2017 | Vancomycin | FMT |  |  | 10; 9 |
| Cammarota et al., 2015 | Vancomycin | FMT |  |  | 19; 20 |
| Rode et al., 2021 | Vancomycin | FMT | Rectal bacteriotherapy |  | 31; 34; 33 |
| van Nood et al., 2013 | Vancomycin | FMT | Vancomycin + bowel lavage |  | 13; 16; 13 |
| Wenisch et al., 1996 | Vancomycin | Fusidic acid | Metronidazole | Teicoplanin | 21; 29; 31; 28 |
| Zar et al., 2007 | Vancomycin | Metronidazole |  |  | 71; 79 |
| Johnson et al., 1992 | Vancomycin | Metronidazole | Placebo |  | 10; 10; 10 |
| Johnson et al., 2014 | Vancomycin | Metronidazole | Tolevamer |  | 266; 289; 563 |
| Teasley et al., 1983 | Vancomycin | Metronidazole |  |  | 52; 42 |
| Musher et al., 2009 | Vancomycin | Nitazoxanide |  |  | 27; 23 |
| Okhuysen et al, 2024 | Vancomycin | Ridinilazole |  |  | 375; 370 |
| Boix et al., 2017 | Vancomycin | Surotomycin |  |  | 280; 290 |
| Daley et al., 2017 | Vancomycin | Surotomycin |  |  | 292; 285 |
| De Lalla et al., 1992 | Vancomycin | Teicoplanin |  |  | 20; 27 |
| van Nood et al., 2013 | Vancomycin + bowel lavage | FMT | Vancomycin |  | 13; 16; 13 |

FMT=Fecal microbiota transplantation

# ***Table S6:* Treatments – Cure rate, recurrent cases**

| **Article (First author, year)** | **Treatment 1** | **Treatment 2** | **Treatment 3** | **Treatment 4** | **Patients number** |
| --- | --- | --- | --- | --- | --- |
| Hvas et al., 2019 | Fidaxomicin | FMT | Vancomycin |  | 24; 24; 16 |
| Guery et al., 2018 | Fidaxomicin | Vancomycin |  |  | 177; 179 |
| Mikamo et al., 2018 | Fidaxomicin | Vancomycin |  |  | 104; 108 |
| Hvas et al., 2019 | FMT | Fidaxomicin | Vancomycin |  | 24; 24; 16 |
| Rode et al., 2021 | FMT | Rectal bacteriotherapy | Vancomycin |  | 34; 33; 31 |
| Cammarota et al., 2015 | FMT | Vancomycin |  |  | 20; 19 |
| van Nood et al., 2013 | FMT | Vancomycin | Vancomycin + bowel lavage |  | 16; 13; 13 |
| Wenisch et al., 1996 | Fusidic acid | Metronidazole | Teicoplanin | Vancomycin | 29; 31; 28; 31 |
| Wenisch et al., 1996 | Metronidazole | Fusidic acid | Teicoplanin | Vancomycin | 31; 29; 28; 31 |
| Johnson et al., 2014 | Metronidazole | Tolevamer | Vancomycin |  | 289; 563; 266 |
| Musher et al., 2009 | Nitazoxanide | Vancomycin |  |  | 23; 27 |
| Rode et al., 2021 | Rectal bacteriotherapy | FMT | Vancomycin |  | 33; 34; 31 |
| Boix et al., 2017 | Surotomycin | Vancomycin |  |  | 290; 280 |
| Wenisch et al., 1996 | Teicoplanin | Fusidic acid | Metronidazole | Vancomycin | 28; 29; 31; 31 |
| Johnson et al., 2014 | Tolevamer | Metronidazole | Vancomycin |  | 563; 289; 266 |
| Guery et al., 2018 | Vancomycin | Fidaxomicin |  |  | 179; 177 |
| Mikamo et al., 2018 | Vancomycin | Fidaxomicin |  |  | 108; 104 |
| Hvas et al., 2019 | Vancomycin | Fidaxomicn | FMT |  | 16; 24; 24 |
| Cammarota et al., 2015 | Vancomycin | FMT |  |  | 19; 20 |
| Rode et al., 2021 | Vancomycin | FMT | Rectal bacteriotherapy |  | 31; 34; 33 |
| van Nood et al., 2013 | Vancomycin | FMT | Vancomycin + bowel lavage |  | 13; 16; 13 |
| Wenisch et al., 1996 | Vancomycin | Fusidic acid | Metronidazole | Teicoplanin | 21; 29; 31; 28 |
| Johnson et al., 2014 | Vancomycin | Metronidazole | Tolevamer |  | 266; 289; 563 |
| Musher et al., 2009 | Vancomycin | Nitazoxanide |  |  | 27; 23 |
| Boix et al., 2017 | Vancomycin | Surotomycin |  |  | 280; 290 |
| van Nood et al., 2013 | Vancomycin + bowel lavage | FMT | Vancomycin |  | 13; 16; 13 |

FMT=Fecal microbiota transplantation

# ***Table S7:* Treatments – Cure rate, non-recurrent cases**

| **Article (First author, year)** | **Treatment 1** | **Treatment 2** | **Treatment 3** | **Patients number** |
| --- | --- | --- | --- | --- |
| Dudley et al., 1986 | Bacitracin | Vancomycin |  | 15; 15 |
| Cornely et al., 2012 | Fidaxomicin | Vancomycin |  | 270; 265 |
| Housman et al., 2016 | Fidaxomicin | Vancomycin |  | 18; 16 |
| Louie et al., 2011 | Fidaxomicin | Vancomycin |  | 302; 327 |
| Rao et al, 2024 | Fidaxomicin | Vancomycin |  | 64; 54 |
| Thabit et al., 2016 | Fidaxomicin | Vancomycin |  | 7; 5 |
| Baunwall et al., 2022 | FMT | Placebo |  | 21; 21 |
| Camacho-Ortiz et al., 2017 | FMT | Vancomycin |  | 9; 10 |
| Wullt et al., 2004 | Fusidic acid | Metronidazole |  | 59; 55 |
| Wullt et al., 2004 | Metronidazole | Fusidic acid |  | 55; 59 |
| Lagrotteria et al., 2006 | Metronidazole | Metronidazole+rifampin |  | 20; 19 |
| Johnson et al., 1992 | Metronidazole | Placebo | Vancomycin | 10; 10; 10 |
| Teasley et al., 1983 | Metronidazole | Vancomycin |  | 42; 52 |
| Zar et al., 2007 | Metronidazole | Vancomycin |  | 79; 71 |
| Lagrotteria et al., 2006 | Metronidazole+rifampin | Metronidazole |  | 19; 20 |
| Musher et al., 2009 | Nitazoxanide | Vancomycin |  | 23; 27 |
| Baunwall et al., 2022 | Placebo | FMT (encapsulated) |  | 21; 21 |
| Johnson et al., 1992 | Placebo | Metronidazole | Vancomycin | 10; 10; 10 |
| Okhuysen et al, 2024 | Ridinilazole | Vancomycin |  | 370; 375 |
| Daley et al., 2017 | Surotomycin | Vancomycin |  | 285; 292 |
| De Lalla et al., 1992 | Teicoplanin | Vancomycin |  | 27; 20 |
| Dudley et al., 1986 | Vancomycin | Bacitracin |  | 15; 15 |
| Cornely et al., 2012 | Vancomycin | Fidaxomicin |  | 265; 270 |
| Housman et al., 2016 | Vancomycin | Fidaxomicn |  | 16; 18 |
| Louie et al., 2011 | Vancomycin | Fidaxomicin |  | 327; 302 |
| Rao et al, 2024 | Vancomycin | Fidaxomicin |  | 54; 64 |
| Thabit et al., 2016 | Vancomycin | Fidaxomicin |  | 5; 7 |
| Camacho-Ortiz et al., 2017 | Vancomycin | FMT |  | 10; 9 |
| Teasley et al., 1983 | Vancomycin | Metronidazole |  | 52; 42 |
| Zar et al., 2007 | Vancomycin | Metronidazole |  | 71; 79 |
| Johnson et al., 1992 | Vancomycin | Metronidazole | Placebo | 10; 10; 10 |
| Musher et al., 2009 | Vancomycin | Nitazoxanide |  | 27; 23 |
| Okhuysen et al, 2024 | Vancomycin | Ridinilazole |  | 375; 370 |
| Daley et al., 2017 | Vancomycin | Surotomycin |  | 292; 285 |
| De Lalla et al., 1992 | Vancomycin | Teicoplanin |  | 20; 27 |

FMT=Fecal microbiota transplantation

# ***Table S8:* Treatments – Recurrence**

| **Article (First author, year)** | **Treatment 1** | **Treatment 2** | **Treatment 3** | **Treatment 4** | **Patients number** |
| --- | --- | --- | --- | --- | --- |
| McFarland et al., 1994 | Antibiotics | *Saccharomyces boulardii* + antibiotics |  |  | 67; 57 |
| Dudley et al., 1986 | Bacitracin | Vancomycin |  |  | 15; 15 |
| Cornely et al., 2012 | Fidaxomicin | Vancomycin |  |  | 270; 265 |
| Guery et al., 2018 | Fidaxomicin | Vancomycin |  |  | 177; 179 |
| Housman et al., 2016 | Fidaxomicin | Vancomycin |  |  | 18; 16 |
| Louie et al., 2011 | Fidaxomicin | Vancomycin |  |  | 302; 327 |
| Mikamo et al., 2018 | Fidaxomicin | Vancomycin |  |  | 104; 108 |
| Rao et al, 2024 | Fidaxomicin | Vancomycin |  |  | 64; 54 |
| Thabit et al., 2016 | Fidaxomicin | Vancomycin |  |  | 7; 5 |
| Allegreti et al, 2024 | FMT | FMT + Bezlotoxumab |  |  | 30; 31 |
| Garza-Gonzalez et al., 2019 | FMT | FMT + *Lactobacillus* |  |  | 13; 8 |
| Hota et al., 2017 | FMT | Vancomycin |  |  | 16; 12 |
| van Nood et al., 2013 | FMT | Vancomycin | Vancomycin + bowel lavage |  | 16; 13; 13 |
| Allegreti et al, 2024 | FMT + Bezlotoxumab | FMT |  |  | 31; 30 |
| Garza-Gonzalez et al., 2019 | FMT + *Lactobacillus* | FMT |  |  | 8; 13 |
| Garza-Gonzalez et al., 2019 | FMT + *Lactobacillus* | FMT |  |  | 8; 13 |
| Wullt et al., 2004 | Fusidic acid | Metronidazole |  |  | 59; 55 |
| Wenisch et al., 1996 | Fusidic acid | Metronidazole | Teicoplanin | Vancomycin | 29; 31; 28; 31 |
| Wullt et al., 2004 | Metronidazole | Fusidic acid |  |  | 55; 59 |
| Wenisch et al., 1996 | Metronidazole | Fusidic acid | Teicoplanin | Vancomycin | 31; 29; 28; 31 |
| Lagrotteria et al., 2006 | Metronidazole | Metronidazole+rifampin |  |  | 20; 19 |
| Johnson et al., 2014 | Metronidazole | Tolevamer | Vancomycin |  | 289; 563; 266 |
| Zar et al., 2007 | Metronidazole | Vancomycin |  |  | 79; 71 |
| Lagrotteria et al., 2006 | Metronidazole+rifampin | Metronidazole |  |  | 19; 20 |
| Musher et al., 2009 | Nitazoxanide | Vancomycin |  |  | 23; 27 |
| Okhuysen et al, 2024 | Ridinilazole | Vancomycin |  |  | 370; 375 |
| McFarland et al., 1994 | *Saccharomyces boulardii* + antibiotics | Antibiotics |  |  | 57; 67 |
| Boix et al., 2017 | Surotomycin | Vancomycin |  |  | 290; 280 |
| Daley et al., 2017 | Surotomycin | Vancomycin |  |  | 285; 292 |
| Wenisch et al., 1996 | Teicoplanin | Fusidic acid | Metronidazole | Vancomycin | 28; 29; 31; 31 |
| De Lalla et al., 1992 | Teicoplanin | Vancomycin |  |  | 27; 20 |
| Johnson et al., 2014 | Tolevamer | Metronidazole | Vancomycin |  | 563; 289; 266 |
| Dudley et al., 1986 | Vancomycin | Bacitracin |  |  | 15; 15 |
| Cornely et al., 2012 | Vancomycin | Fidaxomicin |  |  | 265; 270 |
| Guery et al., 2018 | Vancomycin | Fidaxomicin |  |  | 179; 177 |
| Housman et al., 2016 | Vancomycin | Fidaxomicn |  |  | 16; 18 |
| Louie et al., 2011 | Vancomycin | Fidaxomicin |  |  | 327; 302 |
| Mikamo et al., 2018 | Vancomycin | Fidaxomicin |  |  | 108; 104 |
| Rao et al, 2024 | Vancomycin | Fidaxomicin |  |  | 54; 64 |
| Thabit et al., 2016 | Vancomycin | Fidaxomicin |  |  | 5; 7 |
| Hota et al., 2017 | Vancomycin | FMT |  |  | 12; 16 |
| van Nood et al., 2013 | Vancomycin | FMT | Vancomycin + bowel lavage |  | 13; 16; 13 |
| Wenisch et al., 1996 | Vancomycin | Fusidic acid | Metronidazole | Teicoplanin | 21; 29; 31; 28 |
| Zar et al., 2007 | Vancomycin | Metronidazole |  |  | 71; 79 |
| Johnson et al., 2014 | Vancomycin | Metronidazole | Tolevamer |  | 266; 289; 563 |
| Musher et al., 2009 | Vancomycin | Nitazoxanide |  |  | 27; 23 |
| Okhuysen et al, 2024 | Vancomycin | Ridinilazole |  |  | 375; 370 |
| Boix et al., 2017 | Vancomycin | Surotomycin |  |  | 280; 290 |
| Daley et al., 2017 | Vancomycin | Surotomycin |  |  | 292; 285 |
| De Lalla et al., 1992 | Vancomycin | Teicoplanin |  |  | 20; 27 |
| van Nood et al., 2013 | Vancomycin + bowel lavage | FMT | Vancomycin |  | 13; 16; 13 |

FMT=Fecal microbiota transplantation

# ***Table S9:* Treatments – Prevention**

| **Article (First author, year)** | **Treatment 1** | **Treatment 2** | **Treatment 3** | **Treatment 4** | **Patients number** |
| --- | --- | --- | --- | --- | --- |
| Wilcox et al., 2017 | Actoxumab | Actoxumab + Bezlotoxumab | Bezlotoxumab | Placebo | 232; 773; 781; 773 |
| Wilcox et al., 2017 | Actoxumab + Bezlotoxumab | Actoxumab | Bezlotoxumab | Placebo | 773; 232; 781; 773 |
| Johnson et al., 2020 | Antibiotics | No treatment |  |  | 50; 50 |
| Garey et al., 2011 | Antibiotics | Placebo |  |  | 33; 35 |
| Major et al., 2018 | Antibiotics | Placebo |  |  | 77; 74 |
| Mullane et al., 2019 | Antibiotics | Placebo |  |  | 301; 299 |
| Tobar et al., 2018 | Antibiotics | Placebo |  |  | 41; 55 |
| Sadahiro et al., 2014 | Antibiotics | Probiotics | No treatment |  | 99; 100; 95 |
| Wilcox et al., 2017 | Bezlotoxumab | Actoxumab | Actoxumab + Bezlotoxumab | Placebo | 781; 232; 773; 773 |
| Laffan et al., 2012 | Lactoferrin | Placebo |  |  | 9; 13 |
| Johnson et al., 2020 | No treatment | Antibiotics |  |  | 50; 50 |
| Wong et al., 2014 | No treatment | Probiotics |  |  | 85; 79 |
| Sadahiro et al., 2014 | No treatment | Probiotics | Antibiotics |  | 95; 100; 99 |
| Lewis et al., 2005 | Oligofructose | Placebo |  |  | 72; 70 |
| Wilcox et al., 2017 | Placebo | Actoxumab | Actoxumab + Bezlotoxumab | Bezlotoxumab | 773; 232; 773; 781 |
| Garey et al., 2011 | Placebo | Antibiotics |  |  | 35; 33 |
| Major et al., 2018 | Placebo | Antibiotics |  |  | 74; 77 |
| Mullane et al., 2019 | Placebo | Antibiotics |  |  | 299; 301 |
| Tobar et al., 2018 | Placebo | Antibiotics |  |  | 55; 41 |
| Laffan et al., 2012 | Placebo | Lactoferrin |  |  | 13; 9 |
| Lewis et al., 2005 | Placebo | Oligofructose |  |  | 70; 72 |
| Allen et al., 2013 | Placebo | Probiotics |  |  | 1488; 1493 |
| Beausoleil et al., 2007 | Placebo | Probiotics |  |  | 45; 44 |
| Can et al., 2006 | Placebo | Probiotics |  |  | 78; 73 |
| Erhardt et al., 2016 | Placebo | Probiotics |  |  | 231; 246 |
| Hickson et al., 2007 | Placebo | Probiotics |  |  | 66; 69 |
| Lewis et al., 1998 | Placebo | Probiotics |  |  | 36; 33 |
| Morrow et al., 2010 | Placebo | Probiotics |  |  | 73; 73 |
| Nord et al., 1997 | Placebo | Probiotics |  |  | 12; 11 |
| Plummer et al., 2004 | Placebo | Probiotics |  |  | 69; 69 |
| Pozzoni et al., 2012 | Placebo | Probiotics |  |  | 134; 141 |
| Prasoon et al., 2022 | Placebo | Probiotics |  |  | 60; 60 |
| Rajkumar et al., 2020 | Placebo | Probiotics |  |  | 577; 549 |
| Rauseo et al., 2022 | Placebo | Probiotics |  |  | 44; 44 |
| Saviano et al, 2024 | Placebo | Probiotics |  |  | 57; 56 |
| Surawicz et al., 1989 | Placebo | Probiotics |  |  | 16; 32 |
| Surawicz et al., 2000 | Placebo | Probiotics |  |  | 14; 18 |
| Thomas et al., 2001 | Placebo | Probiotics |  |  | 150; 152 |
| Khanna et al., 2022 | Placebo | RBX2660 |  |  | 87; 180 |
| de Bruyn et al., 2021 | Placebo | Vaccine |  |  | 3085; 6173 |
| Sadahiro et al., 2014 | Probiotics | Antibiotics | No treatment |  | 100; 99; 95 |
| Wong et al., 2014 | Probiotics | No treatment |  |  | 79; 85 |
| Allen et al., 2013 | Probiotics | Placebo |  |  | 1493; 1488 |
| Beausoleil et al., 2007 | Probiotics | Placebo |  |  | 44; 45 |
| Can et al., 2006 | Probiotics | Placebo |  |  | 73; 78 |
| Erhardt et al., 2016 | Probiotics | Placebo |  |  | 246; 231 |
| Hickson et al., 2007 | Probiotics | Placebo |  |  | 69; 66 |
| Lewis et al., 1998 | Probiotics | Placebo |  |  | 33; 36 |
| Morrow et al., 2010 | Probiotics | Placebo |  |  | 73; 73 |
| Nord et al., 1997 | Probiotics | Placebo |  |  | 11; 12 |
| Plummer et al., 2004 | Probiotics | Placebo |  |  | 69; 69 |
| Pozzoni et al., 2012 | Probiotics | Placebo |  |  | 141; 134 |
| Prasoon et al., 2022 | Probiotics | Placebo |  |  | 60; 60 |
| Rajkumar et al., 2020 | Probiotics | Placebo |  |  | 549; 577 |
| Rauseo et al., 2022 | Probiotics | Placebo |  |  | 44; 44 |
| Saviano et al, 2024 | Probiotics | Placebo |  |  | 56; 57 |
| Surawicz et al., 1989 | Probiotics | Placebo |  |  | 32; 16 |
| Surawicz et al., 2000 | Probiotics | Placebo |  |  | 18; 14 |
| Thomas et al., 2001 | Probiotics | Placebo |  |  | 152; 150 |
| Khanna et al., 2022 | RBX2660 | Placebo |  |  | 180; 87 |
| de Bruyn et al., 2021 | Vaccine | Placebo |  |  | 6173; 3085 |


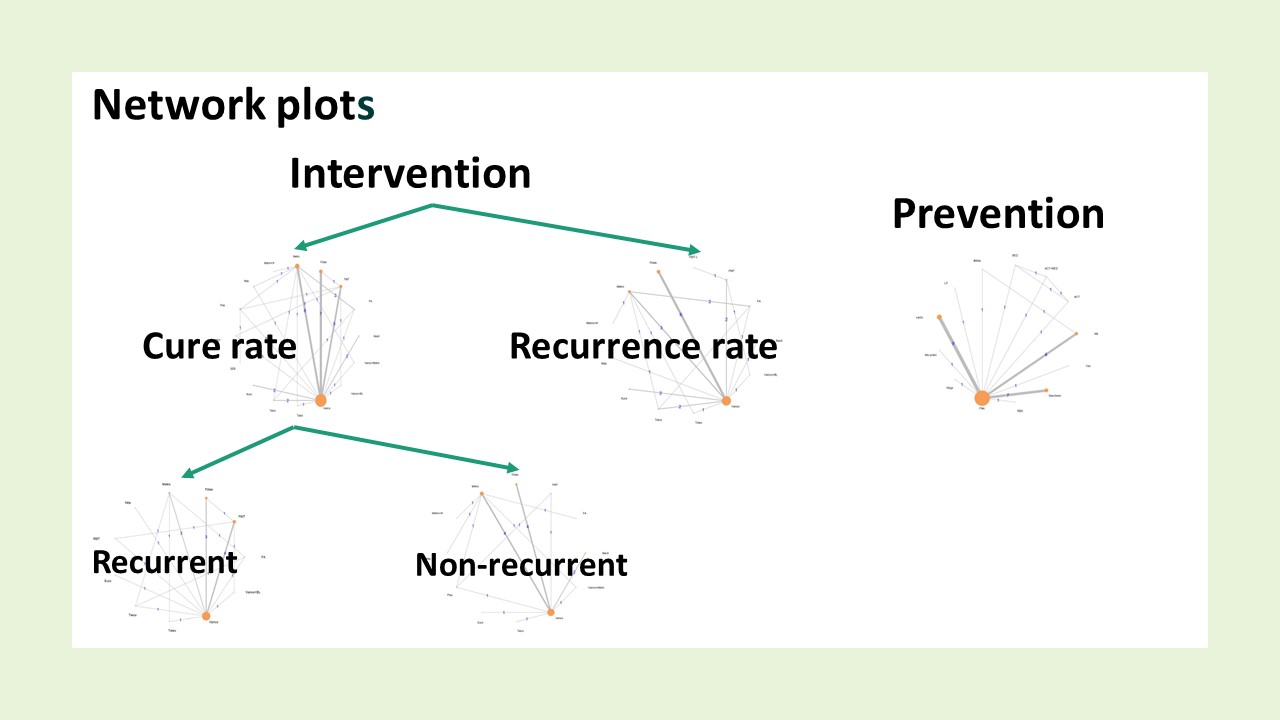


# ***Figure S1:* Analysis endpoints**

We examined the cure rate of all patients and separately examined the recurrent and non-recurrent sub-groups. We also analyzed how recurrence developed with different therapies. The third main investigated endpoint was prevention, with the aim of determining which therapy had the greatest potential to prevent CDI.

| **Summary of network table** | | | | |
| --- | --- | --- | --- | --- |
| **Characteristic** | | | | **Value** |
| Number of Interventions | | | | 16 |
| Number of Studies | | | | 29 |
| Total Number of Patients in Network | | | | 5654 |
| Total Possible Pairwise Comparisons | | | | 120 |
| Total Number of Pairwise Comparisons with Direct Data | | | | 25 |
| Number of Two-arm Studies | | | | 23 |
| Number of Multi-Arms Studies | | | | 6 |
| Total Number of Events in Network | | | | 4189 |
| Number of Studies With No Zero Events | | | | 29 |
| Number of Studies With At Least One Zero Event | | | | 0 |
| Number of Studies With All Zero Events | | | | 0 |
|  |  |  |  | |
| **Treatment** | **Studies (n)** | **Events (n)** | **Patients (n)** | |
| Bacit | 2 | 23 | 32 | |
| FA | 2 | 76 | 88 | |
| Fidax | 8 | 704 | 803 | |
| FMT | 6 | 102 | 116 | |
| Metro | 8 | 417 | 493 | |
| Metro+rif | 1 | 12 | 19 | |
| Nita | 2 | 85 | 94 | |
| Plac | 3 | 65 | 124 | |
| RBT | 1 | 12 | 25 | |
| Ridin | 1 | 270 | 370 | |
| SER | 1 | 78 | 89 | |
| Suro | 2 | 356 | 575 | |
| Teico | 2 | 52 | 54 | |
| Tolev | 1 | 206 | 440 | |
| Vanco | 24 | 1728 | 2319 | |
| Vanco+BL | 1 | 3 | 13 | |

# ***Table S10:* Network summary table of the treatments for cure rates**

Key data of analysis and treatments.

Bacit=Bacitracin; FA=Fusidic acid; Fidax=Fidaxomicin; FMT=Fecal microbiota transplantation; Metro=Metronidazole; Metro+rif=Metronidazole+Rifampin; Nita=Nitazoxanide; Plac=Placebo; RBT=Rectal bacteriotherapy; Ridin= Ridinilazole; SER=SER 109; Suro=Surotomycin; Teico=Teicoplanin; Tolev=Tolevamer; Vanco=Vancomycin; Vanco+BL=Vancomycin+bowel lavage


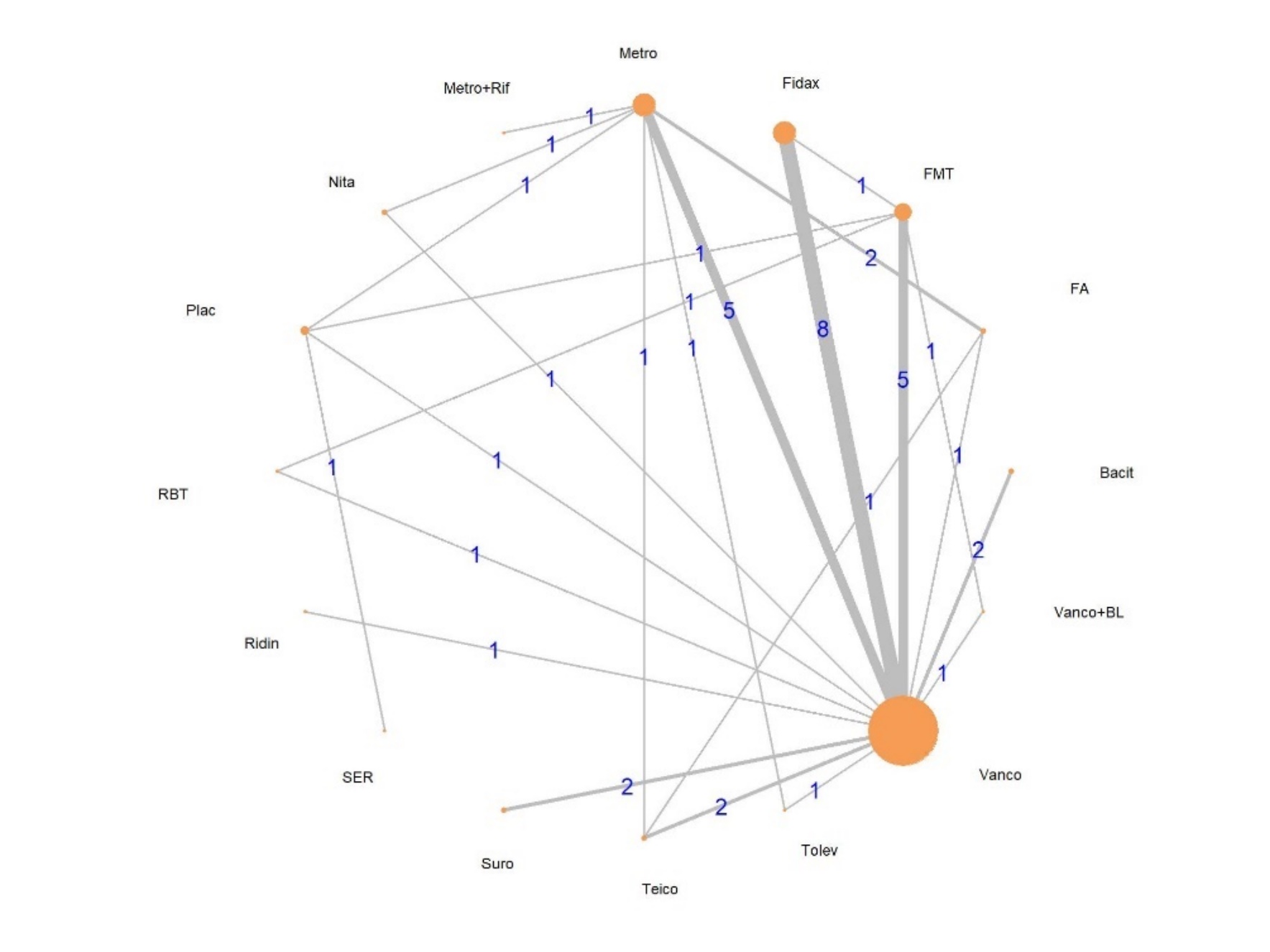


# ***Figure S2:* Network plot of possible treatments for cure rate**

Every knot represents a different therapy for CDI. The larger the knot, the more studies included the treatment. Every edge compares different therapies. The width and the number above indicate how many studies investigated this comparison.

Bacit=Bacitracin; FA=Fusidic acid; Fidax=Fidaxomicin; FMT=Fecal microbiota transplantation; Metro=Metronidazole; Metro+rif=Metronidazole+Rifampin; Nita=Nitazoxanide; Plac=Placebo; RBT=Rectal bacteriotherapy; Ridin=Ridinilazole; SER=SER 109; Suro=Surotomycin; Teico=Teicoplanin; Tolev=Tolevamer; Vanco=Vancomycin; Vanco+BL=Vancomycin+bowel lavage

Figure S2 is identical to Figure 2.

|  | P-score (random) |
| --- | --- |
| FMT | 0·9952 |
| Fidax | 0·7616 |
| Nita | 0·7152 |
| RBT | 0·6538 |
| SER | 0·6536 |
| Ridin | 0·5806 |
| Suro | 0·5680 |
| Vanco | 0·5321 |
| Teico | 0·5096 |
| Metro+rif | 0·4373 |
| Metro | 0·4363 |
| Bacit | 0·3658 |
| Vanco+BL | 0·3080 |
| FA | 0·2543 |
| Plac | 0·1812 |
| Tolev | 0·0475 |

# ***Table S11:* P-score table (SUCRA) of the treatments in case of cure rate**

The possible therapies are ranked based on the P-score. P-score shows the average confidence with which we can say that one treatment is better than another. P-score can range from 0 to 1.

Bacit=Bacitracin; FA=Fusidic acid; Fidax=Fidaxomicin; FMT=Fecal microbiota transplantation; Metro=Metronidazole; Metro+rif=Metronidazole+Rifampin; Nita=Nitazoxanide; Plac=Placebo; RBT=Rectal bacteriotherapy; Ridin=Ridinilazole; SER=SER 109; Suro=Surotomycin; Teico=Teicoplanin; Tolev=Tolevamer; Vanco=Vancomycin; Vanco+BL=Vancomycin+bowel lavage


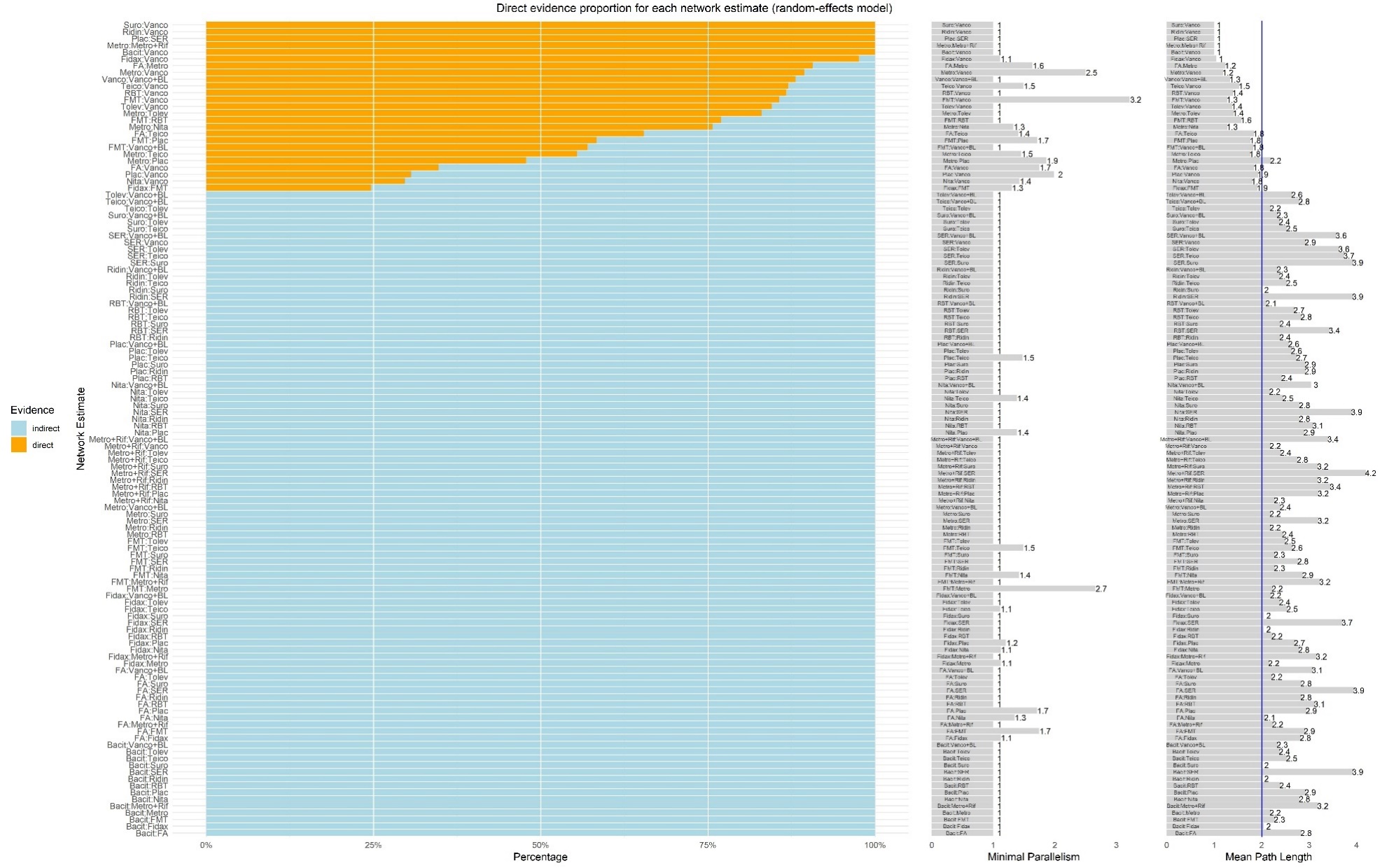


# ***Figure S3:* Evidence plot of the treatments in case of cure rate**

It shows what percentage of the result comes from the direct comparison and how much comes from the indirect/estimated data. The direct ones are marked orange, while the estimated ones are marked blue. In the Mean Path Length diagram, if the given comparison is greater than 2, then these network estimations should be interpreted carefully. Higher values of parallelism indicate greater robustness of the estimate.

Bacit=Bacitracin; FA=Fusidic acid; Fidax=Fidaxomicin; FMT=Fecal microbiota transplantation; Metro=Metronidazole; Metro+rif=Metronidazole+Rifampin; Nita=Nitazoxanide; Plac=Placebo; RBT=Rectal bacteriotherapy; Ridin=Ridinilazole; SER=SER 109; Suro=Surotomycin; Teico=Teicoplanin; Tolev=Tolevamer; Vanco=Vancomycin; Vanco+BL=Vancomycin+bowel lavage


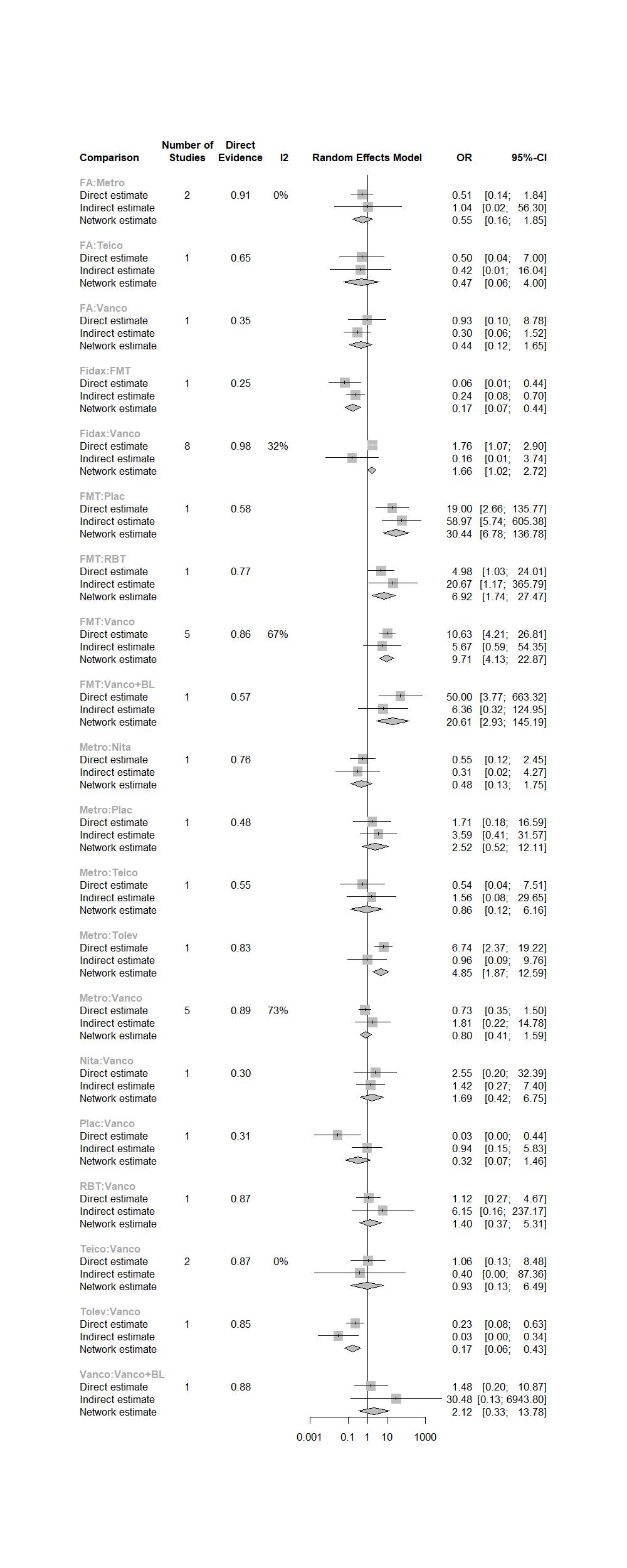


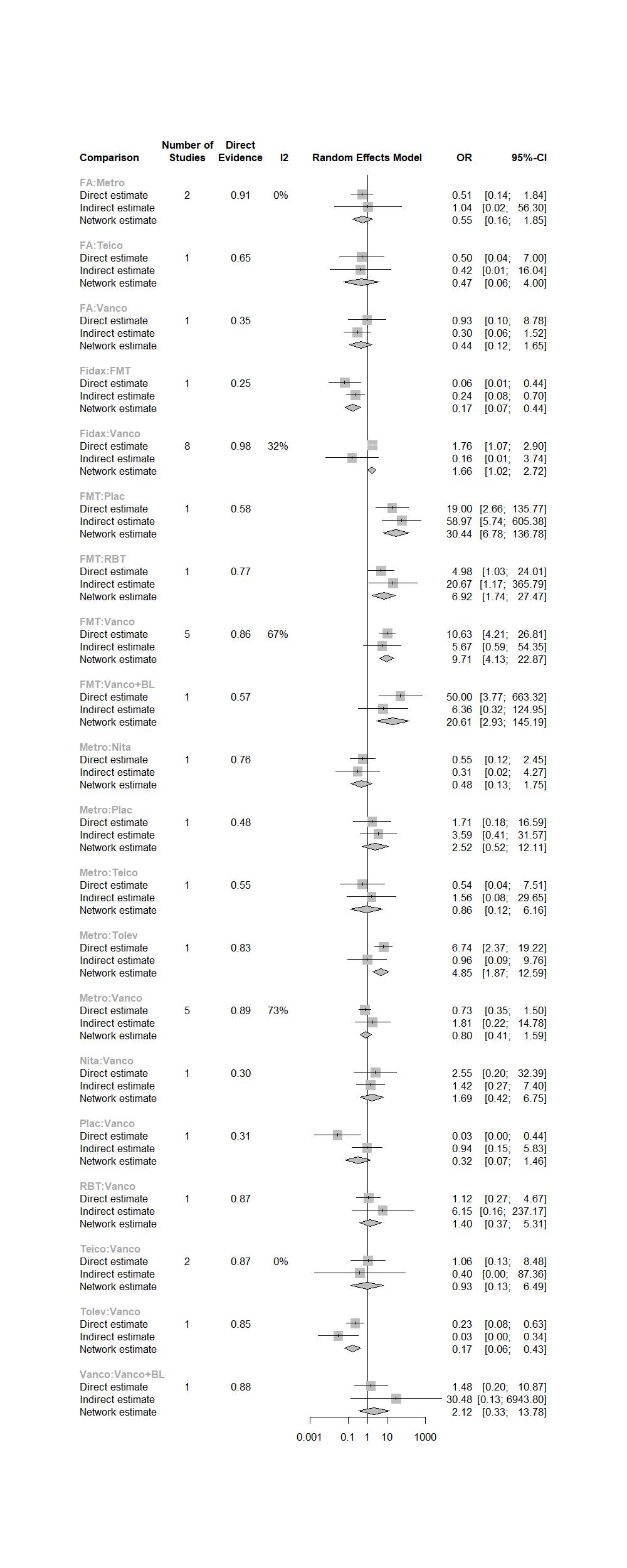


# ***Figure S4:* Forest plot for results of consistency analysis for the treatments in case of cure rate**

The Forest plot shows the result of the consistency analysis. The direct and indirect comparisons do not contradict each other, so the network can be considered consistent.

FA=Fusidic acid; Fidax=Fidaxomicin; FMT=Fecal microbiota transplantation; Metro=Metronidazole; Nita=Nitazoxanide; Plac=Placebo; RBT=Rectal bacteriotherapy; Teico=Teicoplanin; Tolev=Tolevamer; Vanco=Vancomycin; Vanco+BL=Vancomycin+bowel lavage


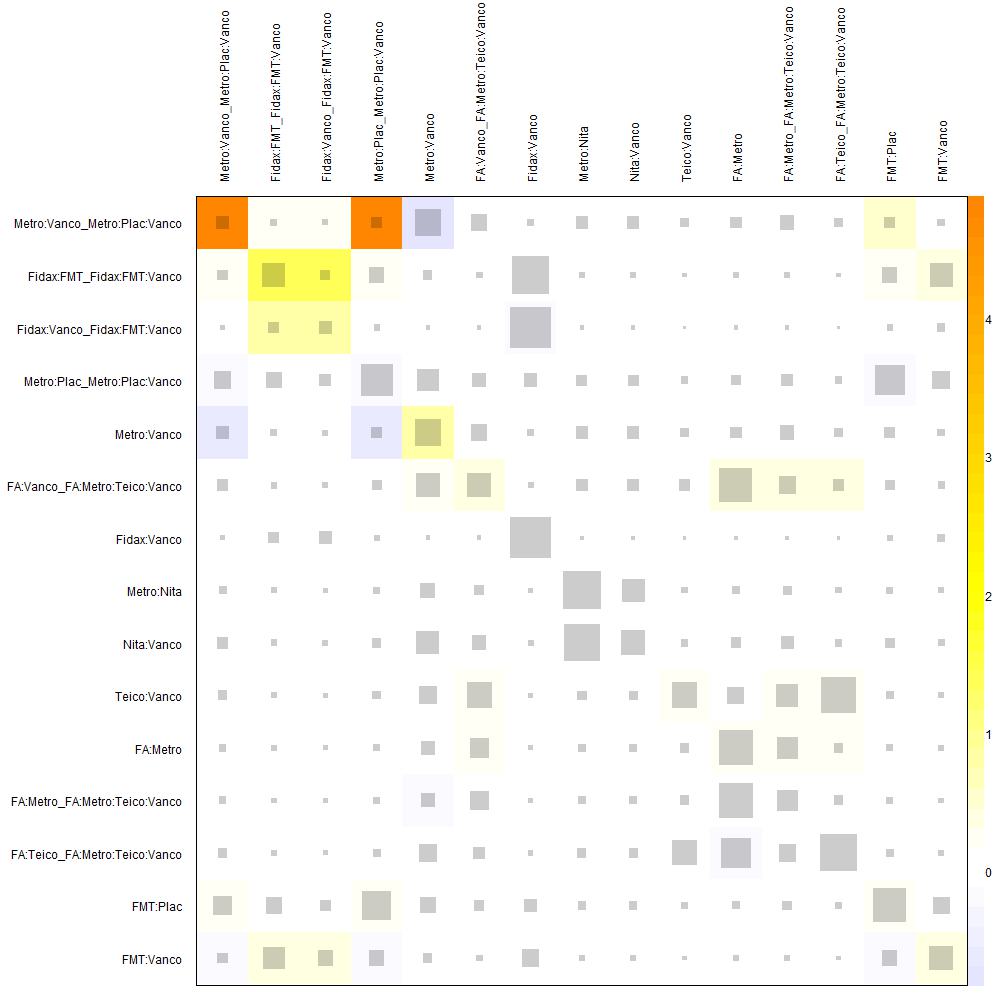


# ***Figure S5:* Net heat plot of the treatments in case of cure rate**

It assesses if there is a consistency problem or not. It shows the difference between direct estimation and the network estimation. The right column shows that the redder the difference, the more inconsistent the result. The area of a gray square represents the contribution of the direct estimate from a design in the column to the network estimate in the row.

Bacit=Bacitracin; FA=Fusidic acid; Fidax=Fidaxomicin; FMT=Fecal microbiota transplantation; FMT+Vanco=FMT+Vancomycin; Metro=Metronidazole;Nita=Nitazoxanide; Plac=Placebo; Teico=Teicoplanin; Vanco=Vancomycin


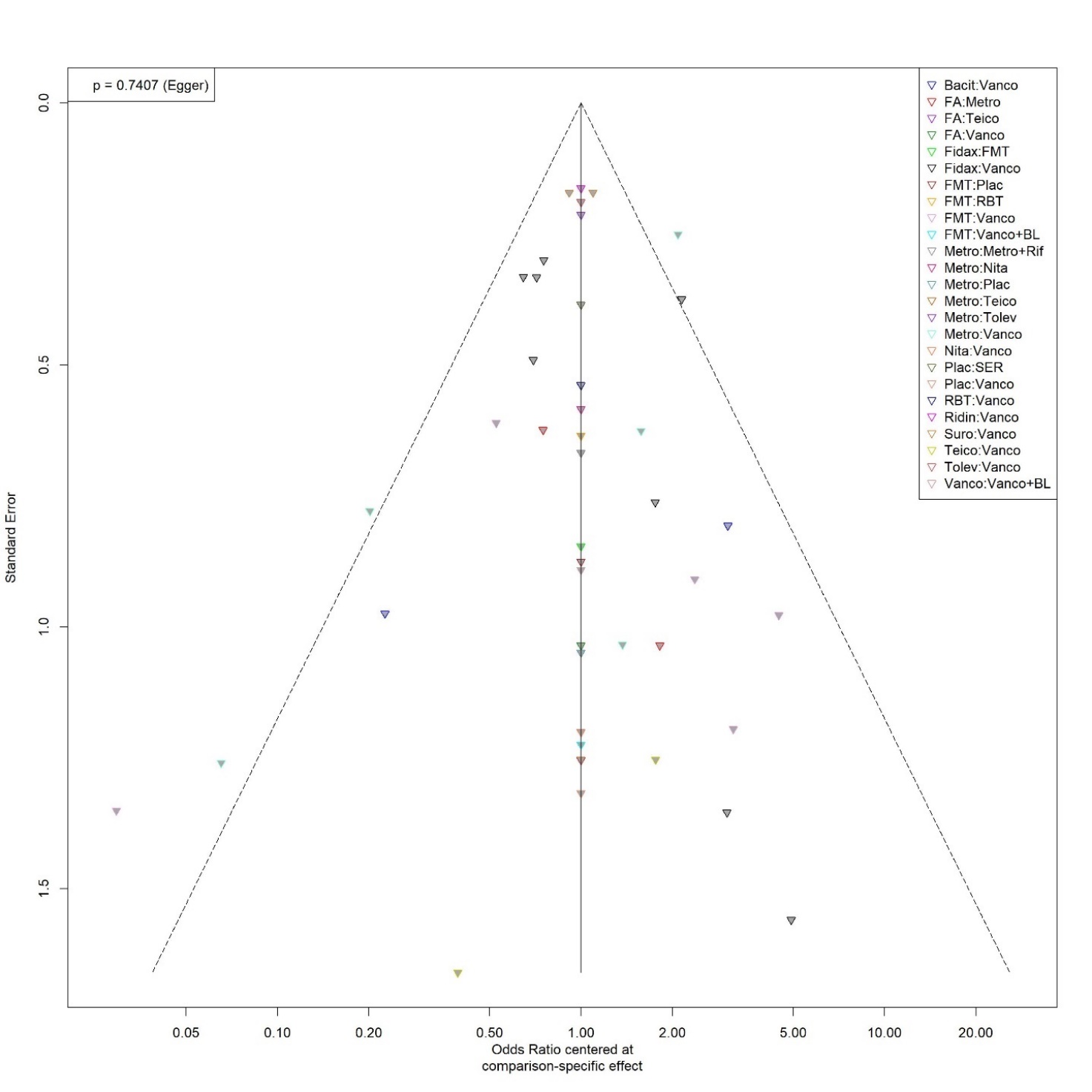


# ***Figure S6:* Funnel plot of the treatments in case of cure rate**

It shows the extent to which the analysis is affected by the small study effect. If the points are under the tent, symmetrically distributed, and the Egger number is bigger than 0.05, in that case the small study effect does not affect our analysis.

Bacit=Bacitracin; FA=Fusidic acid; Fidax=Fidaxomicin; FMT=Fecal microbiota transplantation; FMT+Vanco=FMT+Vancomycin; Metro=Metronidazole; Metro+rif=Metronidazole+Rifampin; Nita=Nitazoxanide; Plac=Placebo; RBT=Rectal bacteriotherapy; Ridin=Ridinilazole; SER=SER 109; Suro=Surotomycin Teico=Teicoplanin; Tolev=Tolevamer; Vanco=Vancomycin; Vanco+BL=Vancomycin+bowel lavage

| **Summary of network table** | | | | |
| --- | --- | --- | --- | --- |
| **Characteristic** | | | | **Value** |
| Number of Interventions | | | | 11 |
| Number of Studies | | | | 10 |
| Total Number of Patients in Network | | | | 2283 |
| Total Possible Pairwise Comparisons | | | | 55 |
| Total Number of Pairwise Comparisons with Direct Data | | | | 17 |
| Number of Two-arm Studies | | | | 5 |
| Number of Multi-Arms Studies | | | | 5 |
| Total Number of Events in Network | | | | 1541 |
| Number of Studies With No Zero Events | | | | 10 |
| Number of Studies With At Least One Zero Event | | | | 0 |
| Number of Studies With All Zero Events | | | | 0 |
|  |  |  |  | |
| **Treatment** | **Studies (n)** | **Events (n)** | **Patients (n)** | |
| FA | 1 | 27 | 29 | |
| Fidax | 3 | 189 | 236 | |
| FMT | 4 | 78 | 88 | |
| Metro | 2 | 219 | 253 | |
| Nita | 1 | 17 | 18 | |
| RBT | 1 | 12 | 25 | |
| Suro | 1 | 176 | 290 | |
| Teico | 1 | 27 | 28 | |
| Tolev | 1 | 206 | 440 | |
| Vanco | 10 | 587 | 863 | |
| Vanco+BL | 1 | 3 | 13 | |

# ***Table S12:* Network summary table of the treatments for cure rates in recurrent cases**

Key data of analysis and treatments.

FA=Fusidic acid; Fidax=Fidaxomicin; FMT=Fecal microbiota transplantation; Metro=Metronidazole; Nita=Nitazoxanide; RBT=Rectal bacteriotherapy; Suro=Surotomycin; Teico=Teicoplanin; Tolev=Tolevamer; Vanco=Vancomycin; Vanco+BL=Vancomycin+bowel lavage


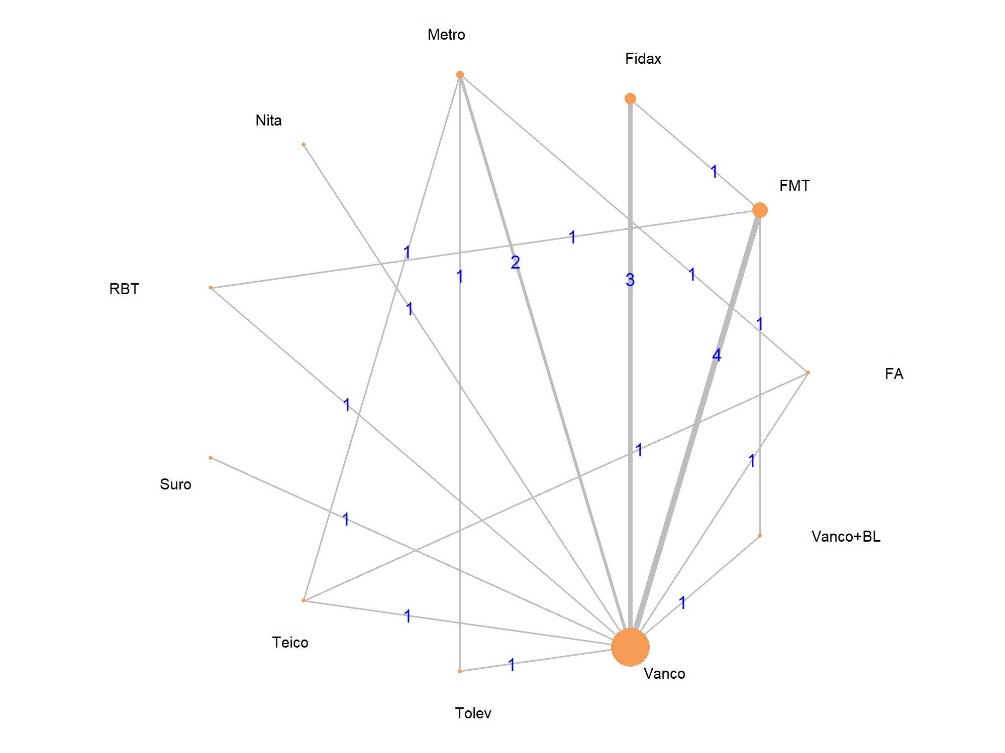


# ***Figure S7:* Network plot of possible treatments for cure rates in recurrent cases**

Every knot represents a different therapy for CDI. The larger a knot, the more studies included that treatment. Every edge compares different therapies. The width and the number above indicate how many studies investigated this comparison.

FA=Fusidic acid; Fidax=Fidaxomicin; FMT=Fecal microbiota transplantation; Metro=Metronidazole; Nita=Nitazoxanide; RBT=Rectal bacteriotherapy; Suro=Surotomycin; Teico=Teicoplanin; Tolev=Tolevamer; Vanco=Vancomycin; Vanco+BL=Vancomycin+bowel lavage

|  | P-score (random) |
| --- | --- |
| FMT | 0·9836 |
| Fidax | 0·6734 |
| Nita | 0·6452 |
| Teico | 0·6200 |
| RBT | 0·5857 |
| Metro | 0·5214 |
| FA | 0·4363 |
| Suro | 0·3830 |
| Vanco | 0·3677 |
| Vanco+BL | 0·2471 |
| Tolev | 0·0365 |

# ***Table S13:* P-score table (SUCRA) of the treatments in case of cure rate in the recurrent cases**

The possible therapies are ranked based on the P-score. P-score shows the average confidence with which we can say that one treatment is better than another. P-score can range from 0 to 1.

FA=Fusidic acid; Fidax=Fidaxomicin; FMT=Fecal microbiota transplantation; Metro=Metronidazole; Nita=Nitazoxanide; RBT=Rectal bacteriotherapy; Suro=Surotomycin; Teico=Teicoplanin; Tolev=Tolevamer; Vanco=Vancomycin; Vanco+BL=Vancomycin+bowel lavage


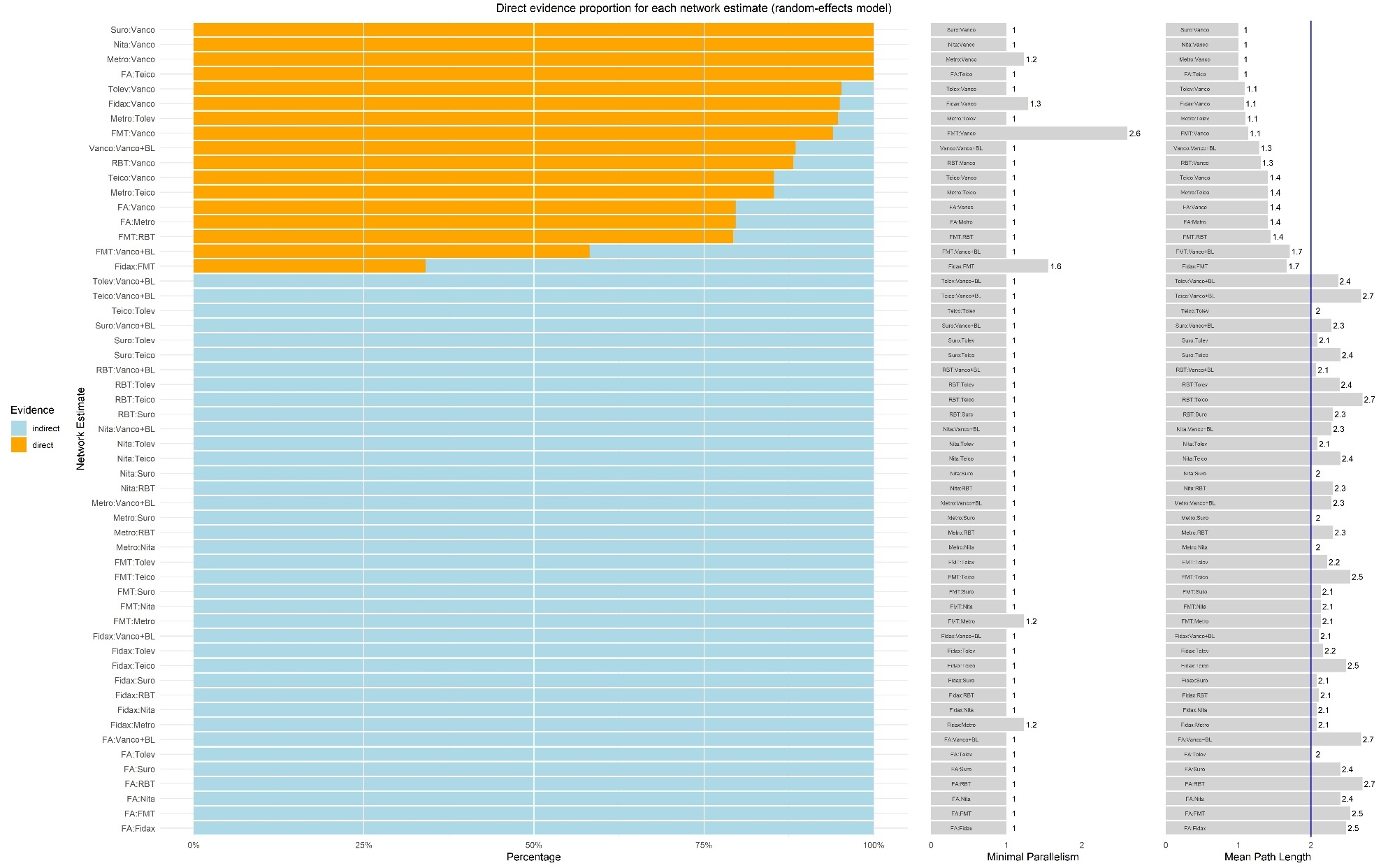


# ***Figure S8:* Evidence plot of the treatments in case of cure rate in the recurrent cases**

It shows what percentage of the result comes from the direct comparison and how much comes from the indirect/estimated data. The direct ones are marked orange, while the estimated ones are marked blue. In the Mean Path Length diagram, if the given comparison is greater than 2, then these network estimations should be interpreted carefully. Higher values of parallelism indicate greater robustness of the estimate.

FA=Fusidic acid; Fidax=Fidaxomicin; FMT=Fecal microbiota transplantation; Metro=Metronidazole; Nita=Nitazoxanide; RBT=Rectal bacteriotherapy; Suro=Surotomycin; Teico=Teicoplanin; Tolev=Tolevamer; Vanco=Vancomycin; Vanco+BL=Vancomycin+bowel lavage


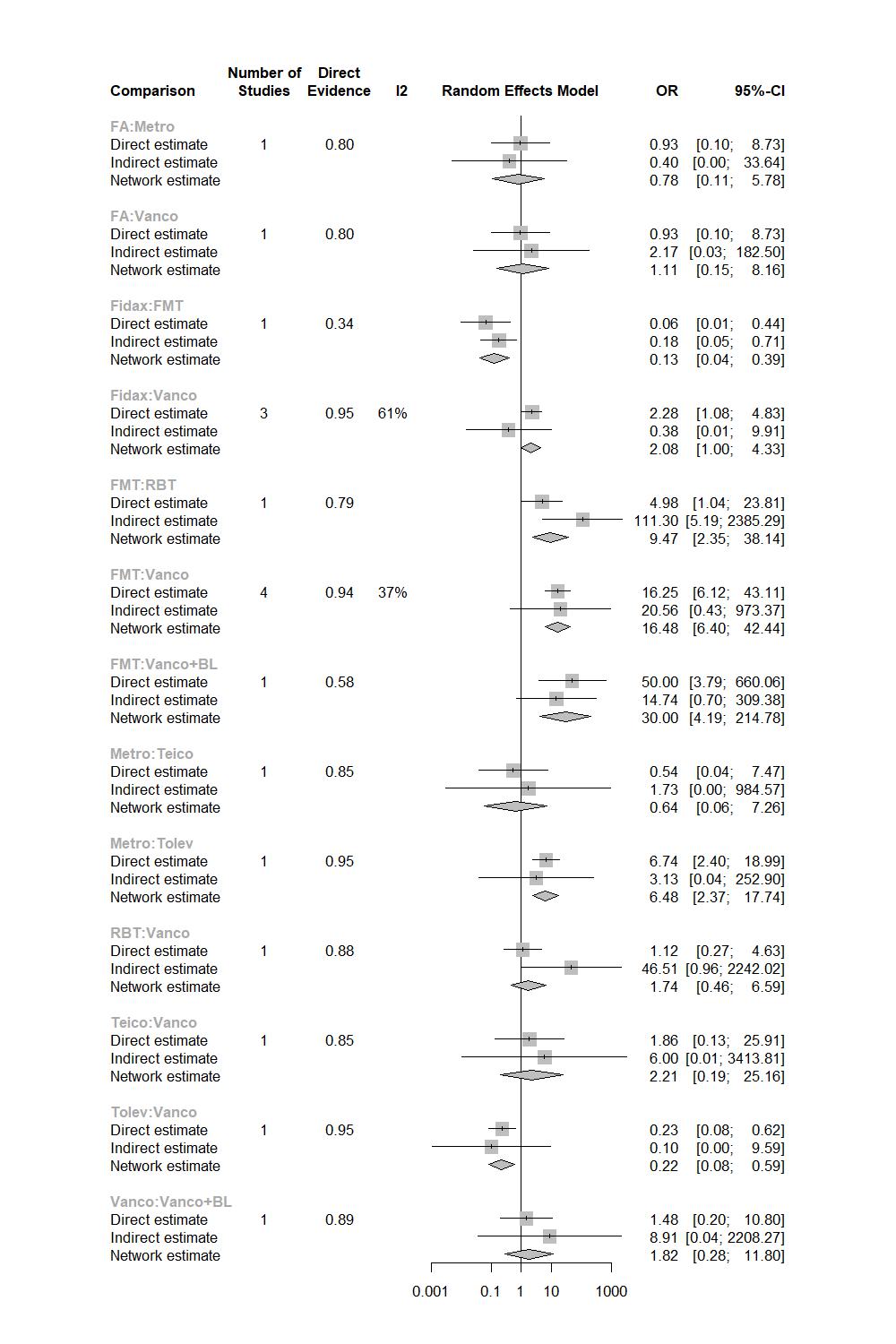


# ***Figure S9:* Forest plot for results of consistency analysis for the treatments in case of cure rate in the recurrent cases**

The Forest plot shows the result of the consistency analysis. The direct and indirect comparisons do not contradict each other, so the network can be considered consistent.

FA=Fusidic acid; Fidax=Fidaxomicin; FMT=Fecal microbiota transplantation; Metro=Metronidazole; RBT=Rectal bacteriotherapy; Teico=Teicoplanin; Tolev=Tolevamer; Vanco=Vancomycin; Vanco+BL=Vancomycin+bowel lavage


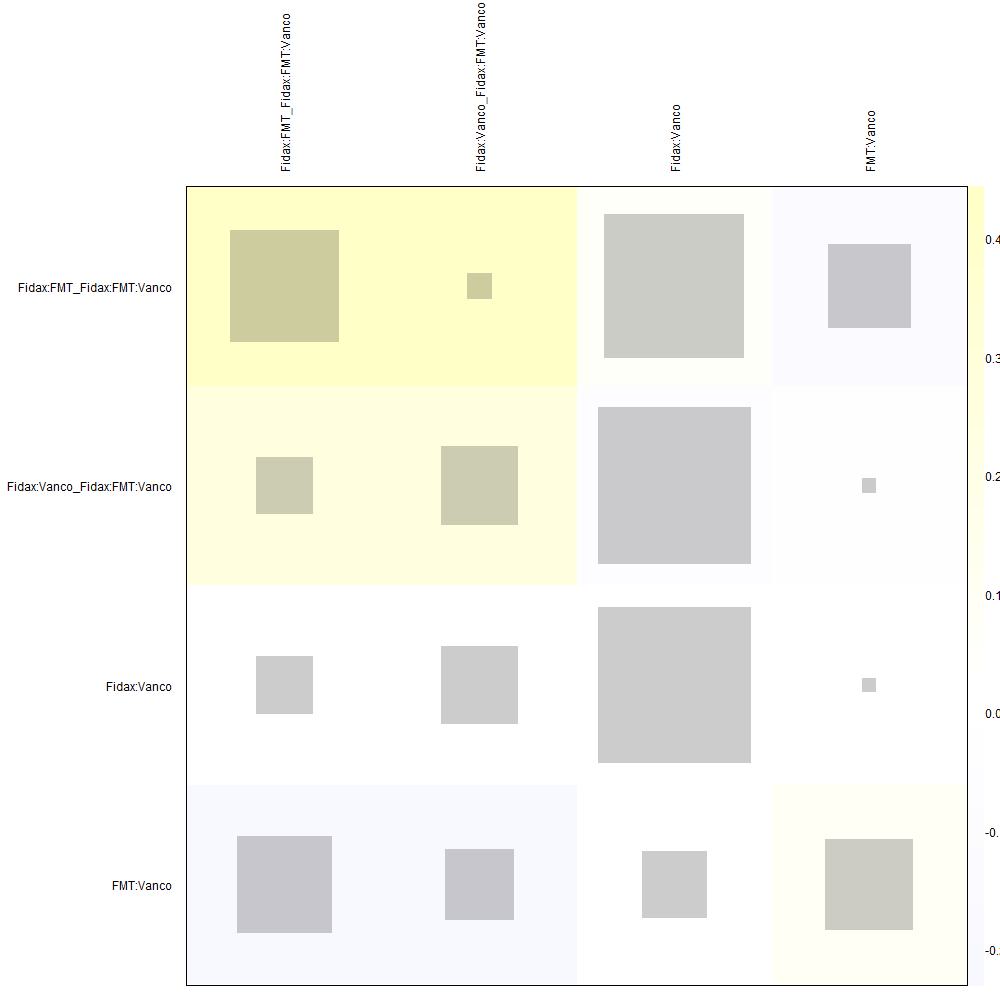


# ***Figure S10:* Net heat plot of the treatments in case of cure rate in the recurrent cases**

It assesses if there is a consistency problem or not. It shows the difference between direct estimation and the network estimation. The right column shows that the redder the difference, the more inconsistent the result. The area of a gray square represents the contribution of the direct estimate from a design in the column to the network estimate in the row.

FMT=Fecal microbiota transplantation; Fidax=Fidaxomicin; Vanco=Vancomycin


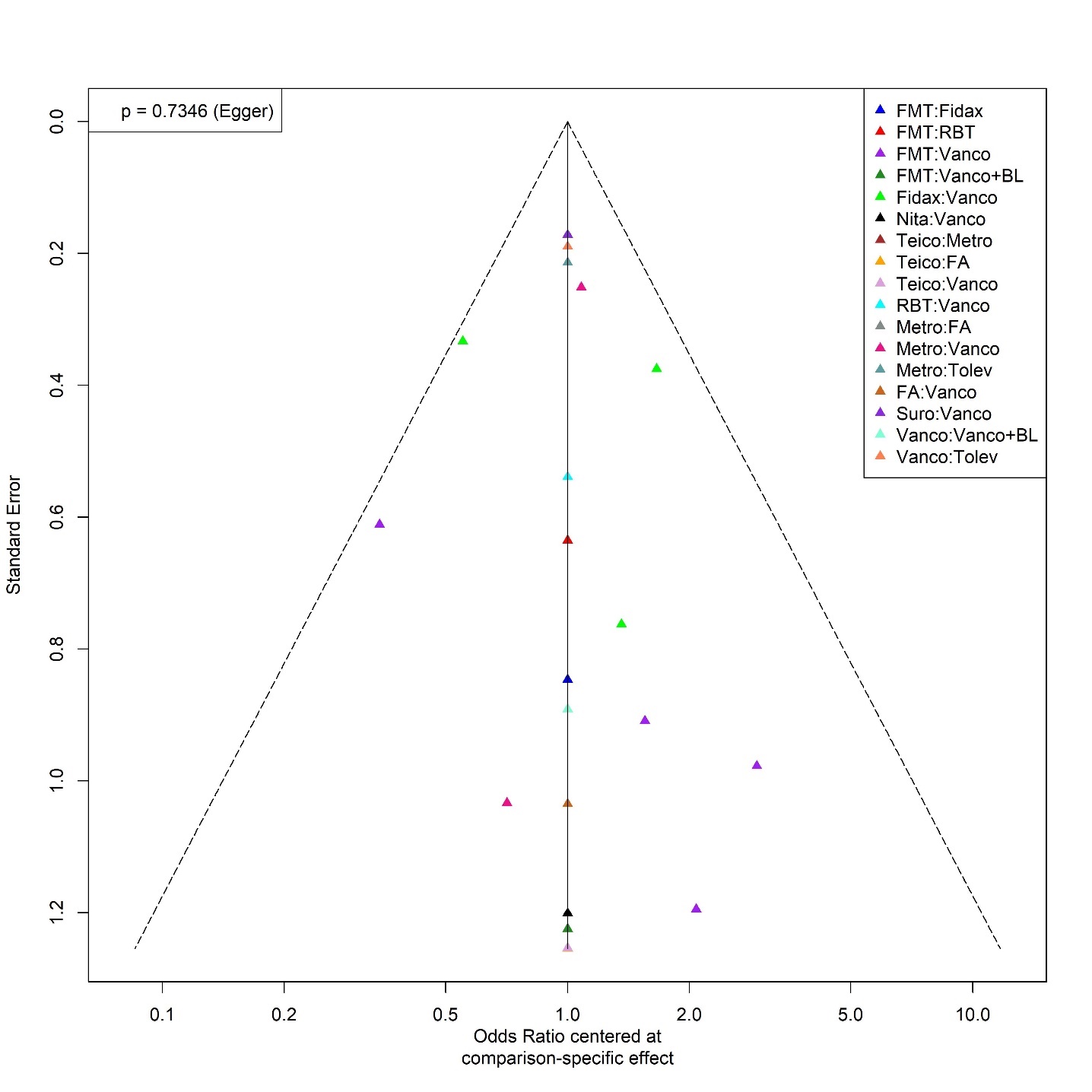


#

# ***Figure S11:* Funnel plot of the treatments in case of cure rate in the recurrent cases**

It shows the extent to which the analysis is affected by the small study effect. If the points are under the tent, symmetrically distributed, and the Egger number is bigger than 0.05, in that case the small study effect does not affect our analysis.

FA=Fusidic acid; Fidax=Fidaxomicin; FMT=Fecal microbiota transplantation; Metro=Metronidazole; Nita=Nitazoxanide; RBT=Rectal bacteriotherapy; Suro=Surotomycin; Teico=Teicoplanin; Tolev=Tolevamer; Vanco=Vancomycin; Vanco+BL=Vancomycin+bowel lavage

| **Summary of network table** | | | | |
| --- | --- | --- | --- | --- |
| **Characteristic** | | | | **Value** |
| Number of Interventions | | | | 12 |
| Number of Studies | | | | 18 |
| Total Number of Patients in Network | | | | 3189 |
| Total Possible Pairwise Comparisons | | | | 66 |
| Total Number of Pairwise Comparisons with Direct Data | | | | 13 |
| Number of Two-arm Studies | | | | 17 |
| Number of Multi-Arms Studies | | | | 1 |
| Total Number of Events in Network | | | | 2515 |
| Number of Studies With No Zero Events | | | | 18 |
| Number of Studies With At Least One Zero Event | | | | 0 |
| Number of Studies With All Zero Events | | | | 0 |
|  |  |  |  | |
| **Treatment** | **Studies (n)** | **Events (n)** | **Patients (n)** | |
| Bacit | 2 | 23 | 32 | |
| FA | 1 | 49 | 59 | |
| Fidax | 5 | 515 | 567 | |
| FMT | 2 | 24 | 28 | |
| Metro | 6 | 198 | 240 | |
| Metro+rif | 1 | 12 | 19 | |
| Nita | 1 | 68 | 76 | |
| Plac | 2 | 9 | 31 | |
| Ridin | 1 | 270 | 370 | |
| Suro | 1 | 180 | 285 | |
| Teico | 1 | 25 | 26 | |
| Vanco | 14 | 1141 | 1456 | |

# ***Table S14:* Network summary table of the treatments for cure rates in non-recurrent cases**

Key data of analysis and treatments.

Bacit=Bacitracin; FA=Fusidic acid; Fidax=Fidaxomicin; FMT=Fecal microbiota transplantation; Metro=Metronidazole; Metro+rif=Metronidazole+Rifampin; Nita=Nitazoxanide; Plac=Placebo; Ridin=Ridinilazole; Suro=Surotomycin; Teico=Teicoplanin; Vanco=Vancomycin


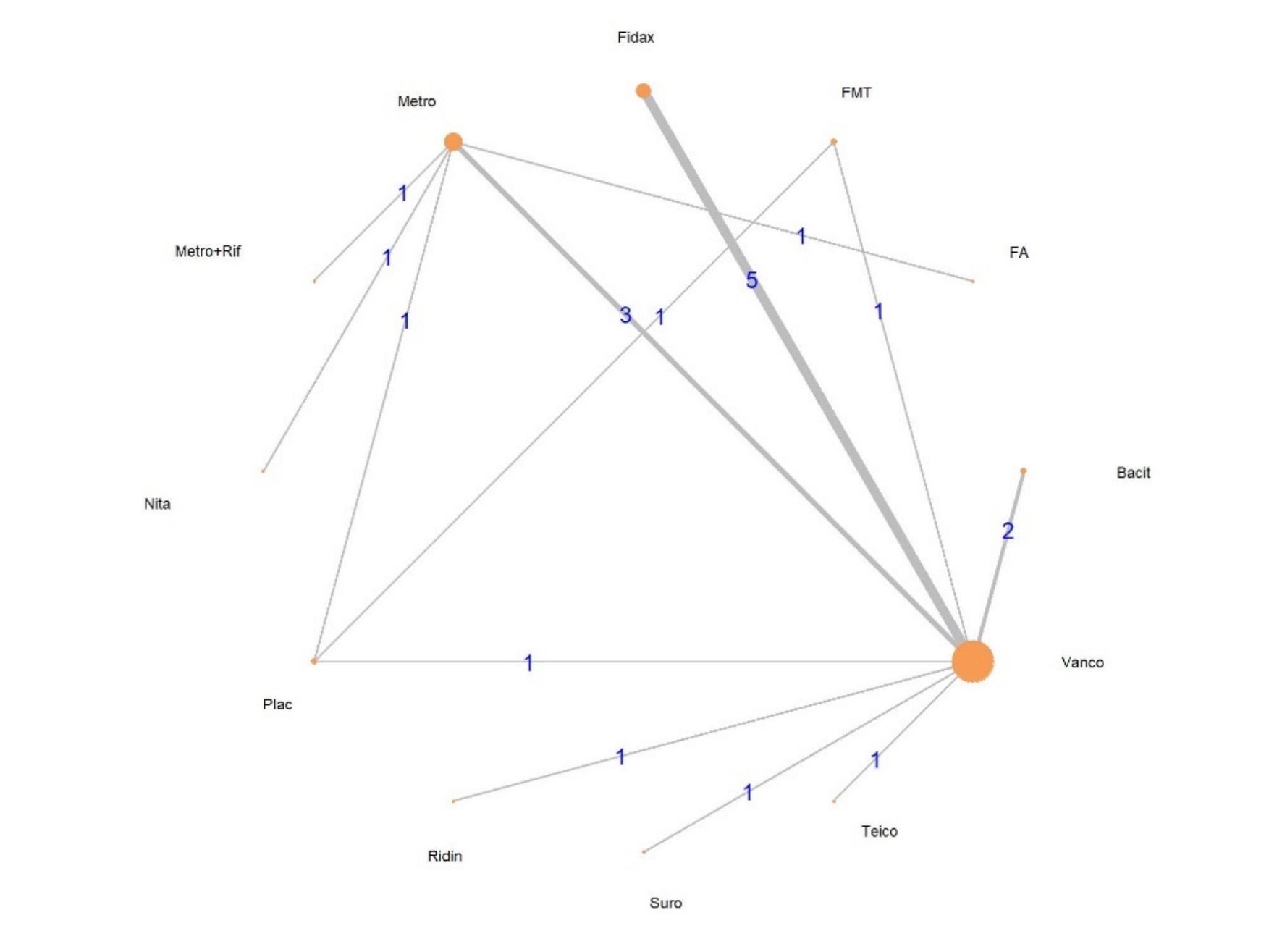


# ***Figure S12:* Network plot of possible treatments for cure rates in non-recurrent cases**

Every knot represents a different therapy for CDI. The larger a knot, the more studies included that treatment. Every edge compares different therapies. The width and the number above indicate how many studies investigated this comparison.

Bacit=Bacitracin; FA=Fusidic acid; Fidax=Fidaxomicin; FMT=Fecal microbiota transplantation; Metro=Metronidazole; Metro+rif=Metronidazole+Rifampin; Nita=Nitazoxanide; Plac=Placebo; Ridin=Ridinilazole; Suro=Surotomycin; Teico=Teicoplanin; Vanco=Vancomycin

|  | P-score (random) |
| --- | --- |
| Fidax | 0·8420 |
| Suro | 0·7448 |
| Ridin | 0·7287 |
| Vanco | 0·6934 |
| FMT | 0·5932 |
| Bacit | 0·5341 |
| Nita | 0·5161 |
| Teico | 0·4679 |
| Metro+Rif | 0·3370 |
| Metro | 0·3231 |
| FA | 0·1526 |
| Plac | 0·0674 |

# ***Table S15:* P-score table (SUCRA) of the treatments in case of cure rate in the non-recurrent cases**

The possible therapies are ranked based on the P-score. P-score shows the average confidence with which we can say that one treatment is better than another. P-score can range from 0 to 1.

Bacit=Bacitracin; FA=Fusidic acid; Fidax=Fidaxomicin; FMT=Fecal microbiota transplantation; Metro=Metronidazole; Metro+rif=Metronidazole+Rifampin; Nita=Nitazoxanide; Plac=Placebo; Ridin=Ridinilazole; Suro=Surotomycin; Teico=Teicoplanin; Vanco=Vancomycin


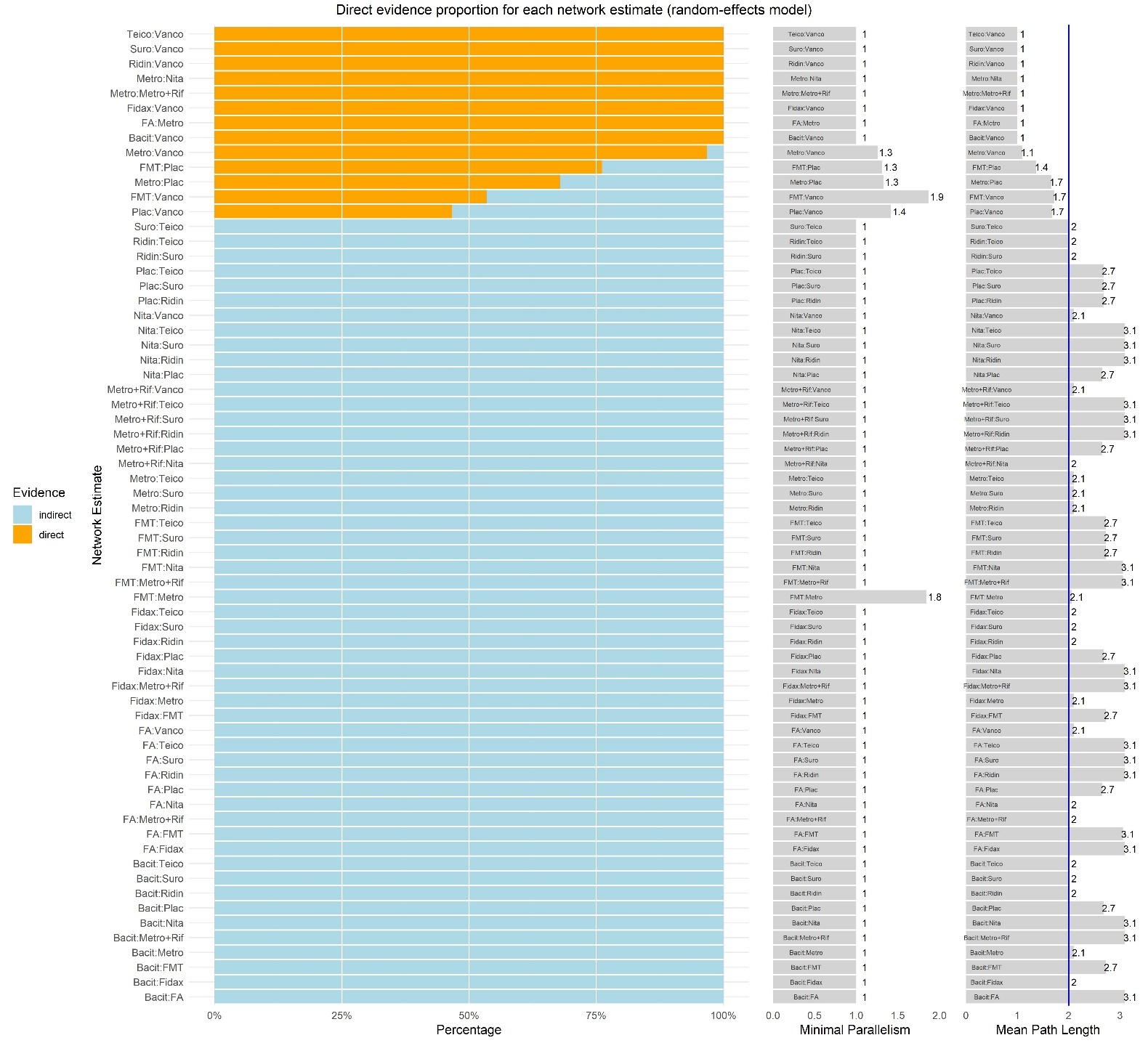


# ***Figure S13:* Evidence plot of the treatments in case of cure rate in the non-recurrent cases**

It shows what percentage of the result comes from the direct comparison and how much comes from the indirect/estimated data. The direct ones are marked orange, while the estimated ones are marked blue. In the Mean Path Length diagram, if the given comparison is greater than 2, then these network estimations should be interpreted carefully. Higher values of parallelism indicate greater robustness of the estimate.

Bacit=Bacitracin; FA=Fusidic acid; Fidax=Fidaxomicin; FMT=Fecal microbiota transplantation; Metro=Metronidazole; Metro+rif=Metronidazole+Rifampin; Nita=Nitazoxanide; Plac=Placebo; Ridin=Ridinilazole; Suro=Surotomycin; Teico=Teicoplanin; Vanco=Vancomycin


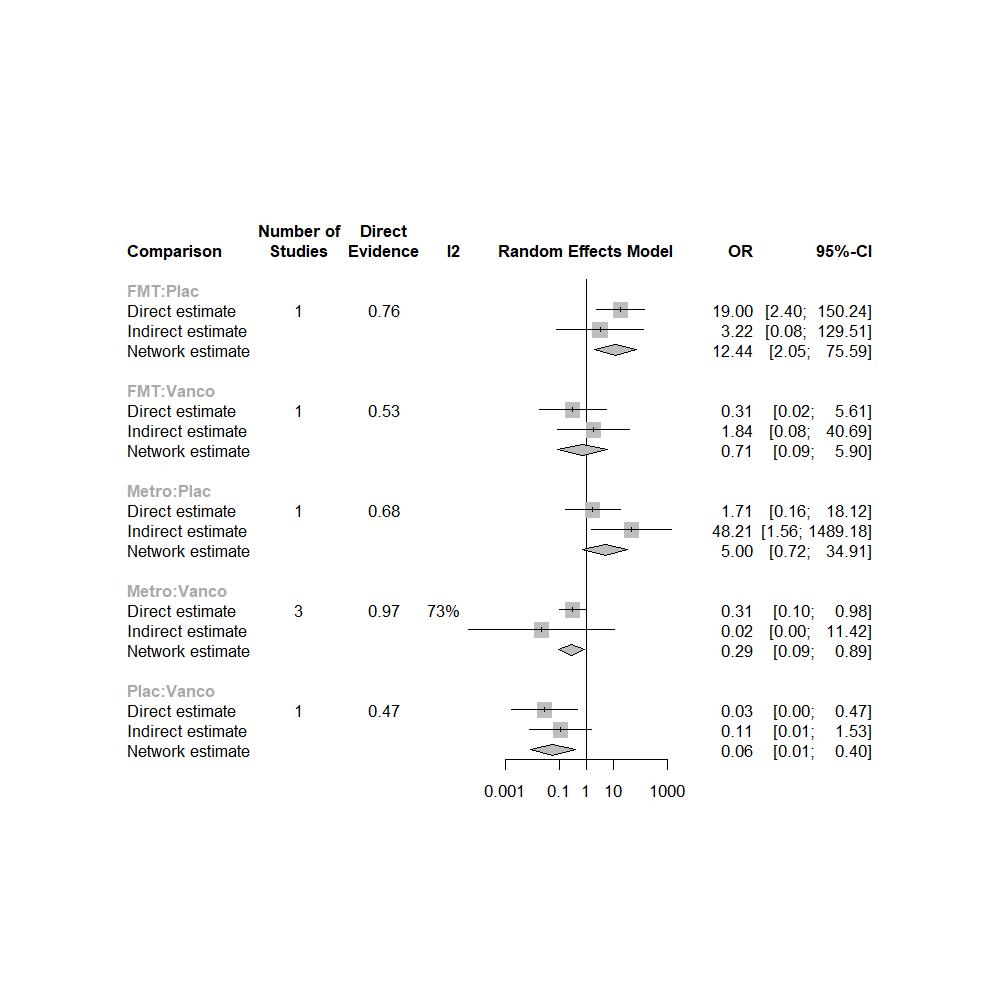


# ***Figure S14:* Forest plot for results of consistency analysis for the treatments in case of cure rate in the non-recurrent cases**

The Forest plot shows the result of the consistency analysis. The direct and indirect comparisons do not contradict each other, so the network can be considered consistent.

FMT=Fecal microbiota transplantation; Metro=Metronidazole; Plac=Placebo; Vanco=Vancomycin


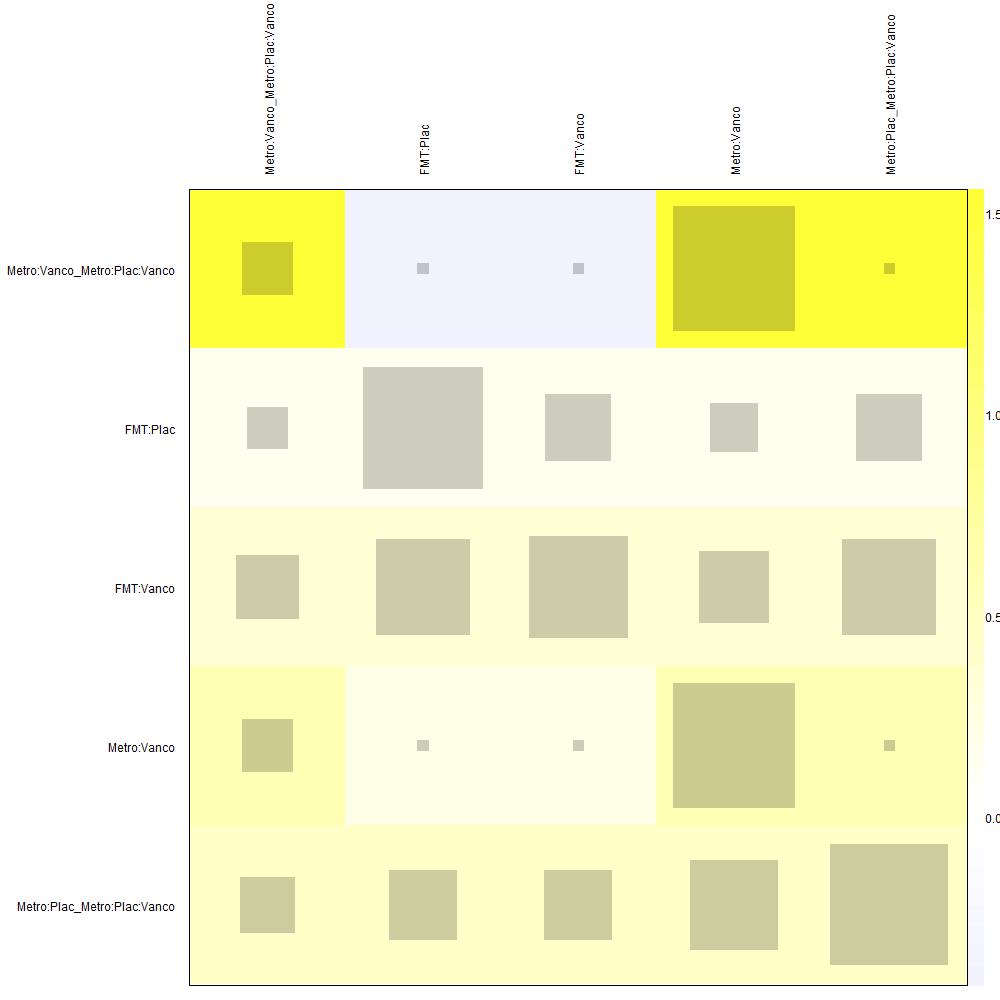


# ***Figure S15:* Net heat plot of the treatments in case of cure rate in the non-recurrent cases**

It assesses if there is a consistency problem or not. It shows the difference between direct estimation and the network estimation. The right column shows that the redder the difference, the more inconsistent the result. The area of a gray square represents the contribution of the direct estimate from a design in the column to the network estimate in the row.

FMT=Fecal microbiota transplantation; Metro=Metronidazole; Plac=Placebo; Vanco=Vancomycin


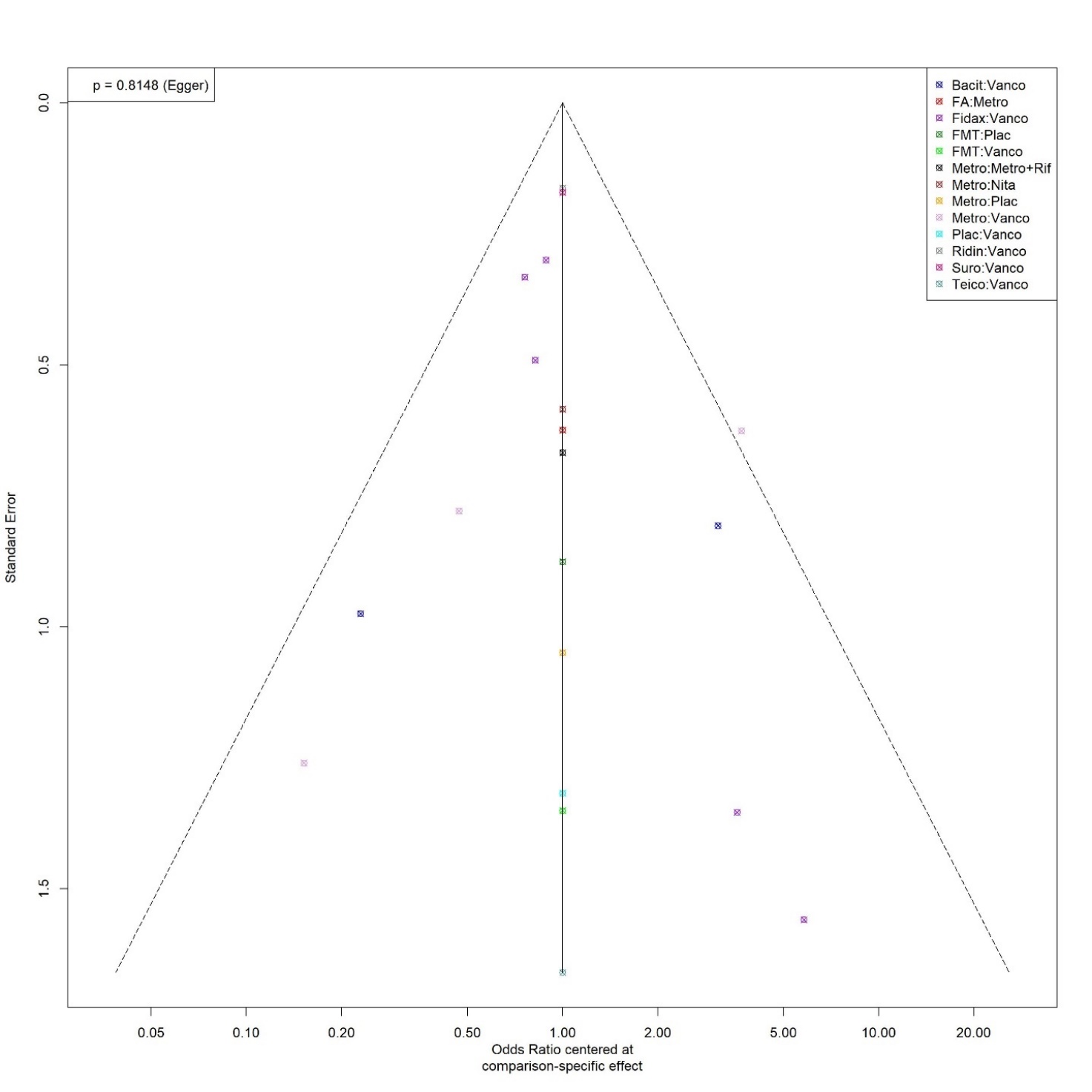


# ***Figure S16:* Funnel plot of the treatments in case of cure rate in the non-recurrent cases**

It shows the extent to which the analysis is affected by the small study effect. If the points are under the tent, symmetrically distributed, and the Egger number is bigger than 0.05, in that case the small study effect does not affect our analysis.

Bacit=Bacitracin; FA=Fusidic acid; Fidax=Fidaxomicin; FMT=Fecal microbiota transplantation; Metro=Metronidazole; Metro+rif=Metronidazole+Rifampin; Nita=Nitazoxanide; Plac=Placebo; Ridin=Ridinilazole; Suro=Surotomycin; Teico=Teicoplanin; Vanco=Vancomycin

| **Summary of network table** | | | | |
| --- | --- | --- | --- | --- |
|  | | | |  |
| Number of Interventions | | | | 15 |
| Number of Studies | | | | 22 |
| Total Number of Patients in Network | | | | 4592 |
| Total Possible Pairwise Comparisons | | | | 105 |
| Total Number of Pairwise Comparisons with Direct Data | | | | 19 |
| Number of Two-arm Studies | | | | 19 |
| Number of Multi-Arms Studies | | | | 3 |
| Total Number of Events in Network | | | | 815 |
| Number of Studies With No Zero Events | | | | 21 |
| Number of Studies With At Least One Zero Event | | | | 1 |
| Number of Studies With All Zero Events | | | | 0 |
|  | 13 |  |  | |
| **Treatment** | **Studies (n)** | **Events (n)** | **Patients (n)** | |
| Bacit | 1 | 5 | 12 | |
| FA | 2 | 22 | 76 | |
| FMT | 4 | 11 | 76 | |
| FMT-Bez | 1 | 4 | 30 | |
| FMT-L | 1 | 1 | 8 | |
| Fidax | 7 | 85 | 722 | |
| Metro | 5 | 75 | 351 | |
| Metro+rif | 1 | 5 | 12 | |
| Nita | 1 | 1 | 17 | |
| Ridin | 1 | 30 | 370 | |
| Suro | 2 | 110 | 584 | |
| Teico | 2 | 4 | 54 | |
| Tolev | 1 | 10 | 202 | |
| Vanco | 18 | 445 | 2065 | |
| Vanco+BL | 1 | 7 | 13 | |

# ***Table S16:* Network summary table of the treatments in recurrence**

Key data of analysis and treatments.

Bacit=Bacitracin; FA=Fusidic acid; Fidax=Fidaxomicin; FMT=Fecal microbiota transplantation; FMT-Bez=Fecal microbiota transplantation+Bezlotoxumab; FMT-L=Fecal microbiota transplantation+*Lactobacillus*; Metro=Metronidazole; Metro+rif=Metronidazole+rifampin; Nita=Nitazoxanide; Ridin=Ridinilazole; Suro=Surotomycin; Teico=Teicoplanin; Tolev=Tolevamer; Vanco=Vancomycin; Vanco+BL=Vancomycin+bowel lavage


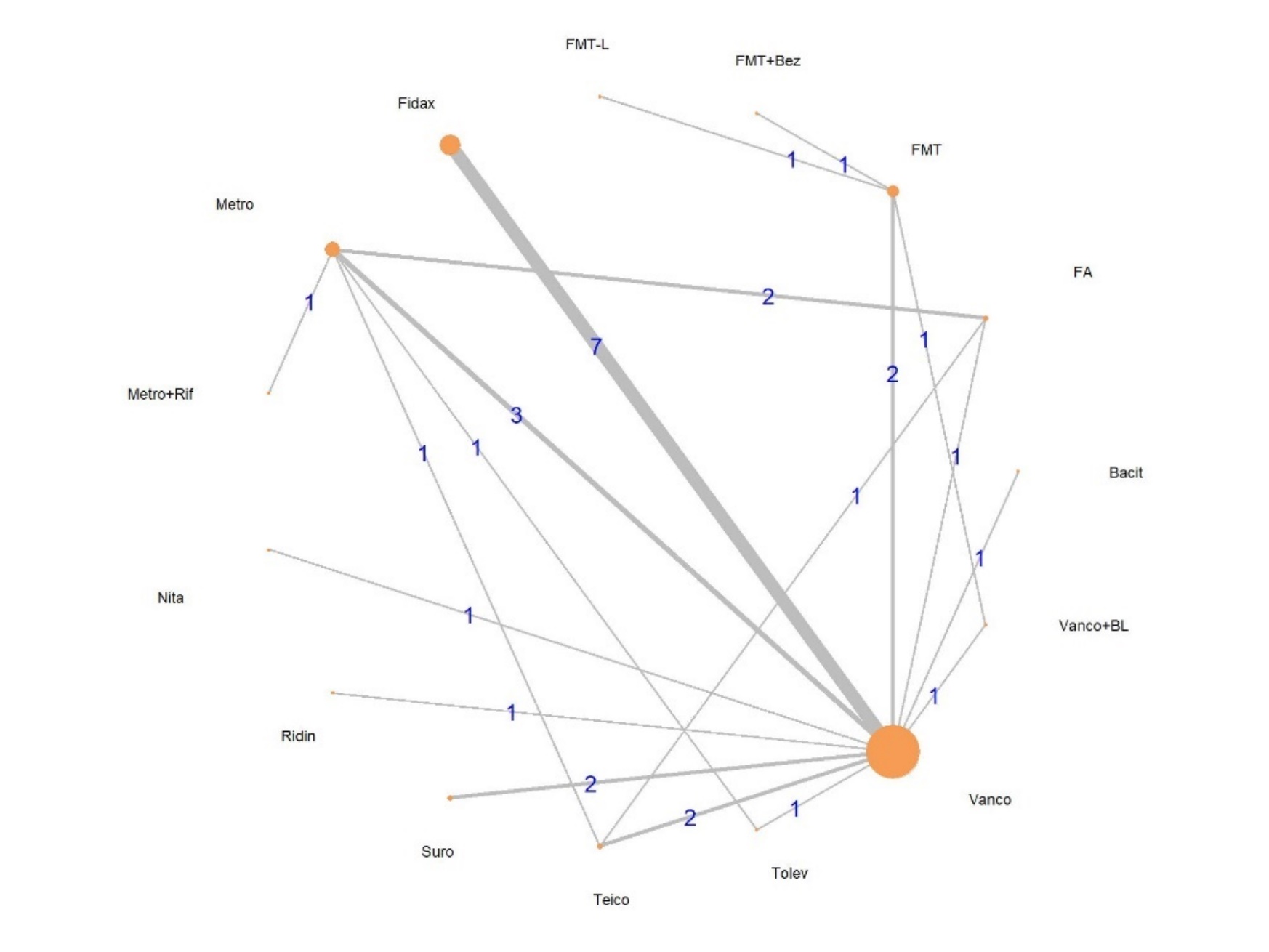


# ***Figure S17:* Network plot of possible treatments in recurrence**

Every knot represents a different therapy for CDI. The larger a knot, the more studies included that treatment. Every edge compares different therapies. The width and the number above indicate how many studies investigated this comparison.

Bacit=Bacitracin; FA=Fusidic acid; Fidax=Fidaxomicin; FMT=Fecal microbiota transplantation; FMT-Bez=Fecal microbiota transplantation+Bezlotoxumab; FMT-L=Fecal microbiota transplantation+*Lactobacillus*; Metro=Metronidazole; Metro+rif=Metronidazole+Rifampin; Nita=Nitazoxanide; Ridin=Ridinilazole; Suro=Surotomycin; Teico=Teicoplanin; Tolev=Tolevamer; Vanco=Vancomycin; Vanco+BL=Vancomycin+bowel lavage

|  | P-score (random) |
| --- | --- |
| Tolev | 0·9490 |
| Teico | 0·7966 |
| Ridin | 0·7671 |
| Fidax | 0·7627 |
| FMT | 0·6455 |
| Nita | 0·6074 |
| Suro | 0·5176 |
| Vanco | 0·4164 |
| Metro | 0·3755 |
| Metro+Rif | 0·3723 |
| Vanco+BL | 0·3411 |
| FA | 0·2820 |
| FMT-L | 0·2588 |
| FMT-Bez | 0·2368 |
| Bacit | 0·1711 |

# ***Table S17:* P-score table (SUCRA) of the treatments in case of recurrence**

The possible therapies are ranked based on the P-score. P-score shows the average confidence with which we can say that one treatment is better than another. P-score can range from 0 to 1.

Bacit=Bacitracin; FA=Fusidic acid; Fidax=Fidaxomicin; FMT=Fecal microbiota transplantation; FMT-Bez=Fecal microbiota transplantation+Bezlotoxumab; FMT-L=Fecal microbiota transplantation+*Lactobacillus*; Metro=Metronidazole; Metro+rif=Metronidazole+Rifampin; Nita=Nitazoxanide; Ridin=Ridinilazole; Suro=Surotomycin; Teico=Teicoplanin; Tolev=Tolevamer; Vanco=Vancomycin; Vanco+BL=Vancomycin+bowel lavage


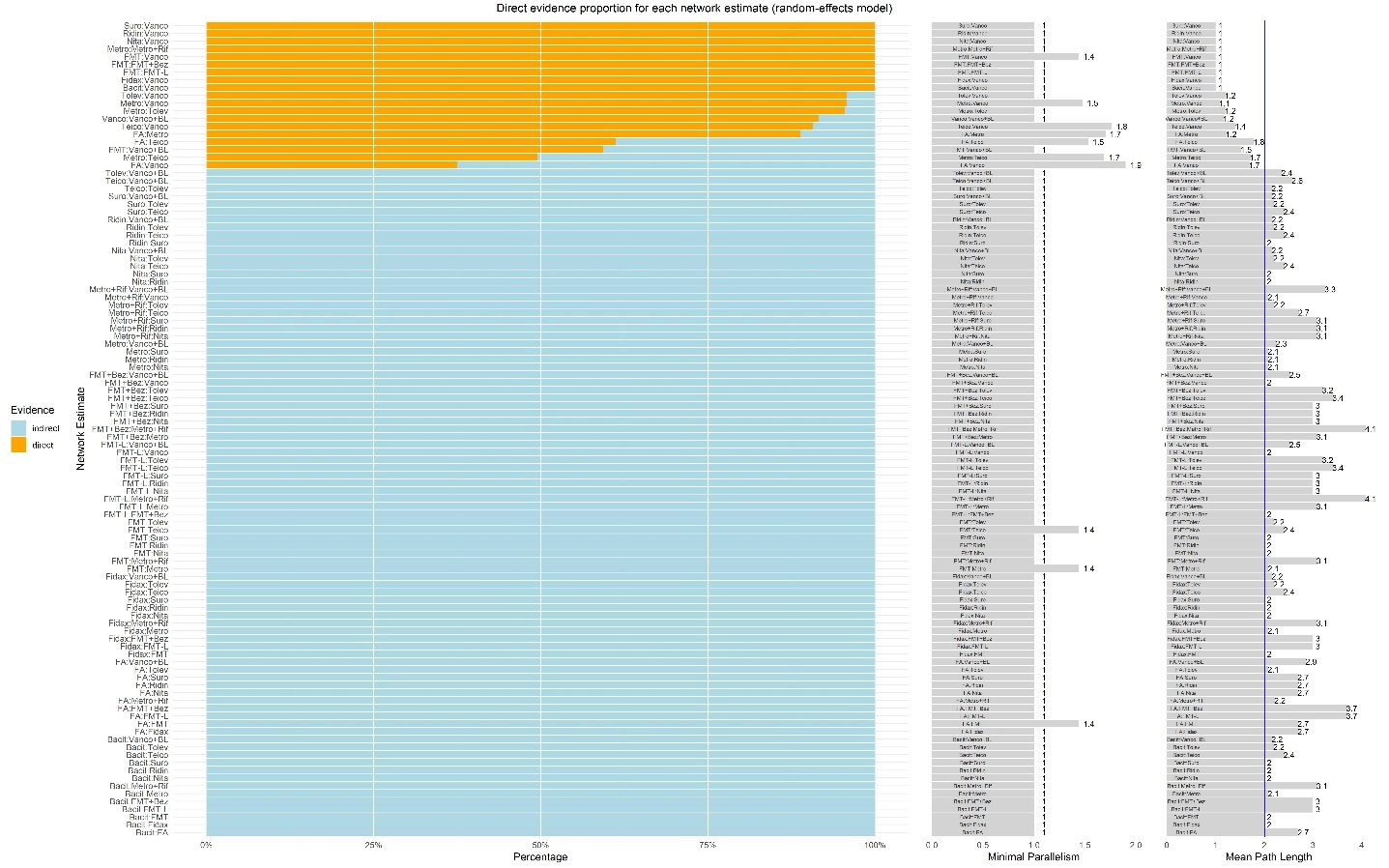


# ***Figure S18:* Evidence plot of the treatments in case of recurrence**

It shows what percentage of the result comes from the direct comparison and how much comes from the indirect/estimated data. The direct ones are marked orange, while the estimated ones are marked blue. In the Mean Path Length diagram, if the given comparison is greater than 2, then these network estimations should be interpreted carefully. Higher values of parallelism indicate greater robustness of the estimate.

Bacit=Bacitracin; FA=Fusidic acid; Fidax=Fidaxomicin; FMT=Fecal microbiota transplantation; FMT-Bez=Fecal microbiota transplantation+Bezlotoxumab; FMT-L=Fecal microbiota transplantation+*Lactobacillus*; Metro=Metronidazole; Metro+rif=Metronidazole+Rifampin; Nita=Nitazoxanide; Ridin=Ridinilazole; Suro=Surotomycin; Teico=Teicoplanin; Tolev=Tolevamer; Vanco=Vancomycin; Vanco+BL=Vancomycin+bowel lavage


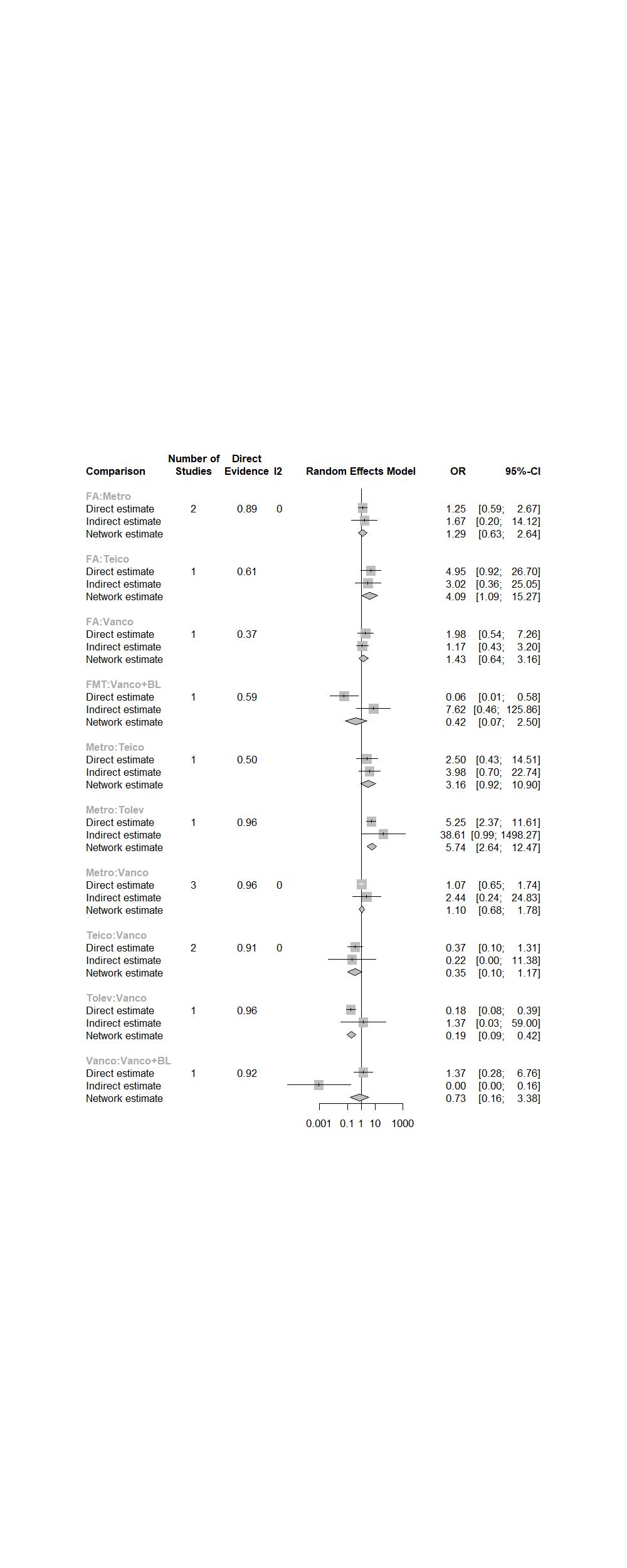


# ***Figure S19:* Forest plot for results of consistency analysis for the treatments in case of recurrence**

The Forest plot shows the result of the consistency analysis. The direct and indirect comparisons do not contradict each other, so the network can be considered consistent.

FA=Fusidic acid; FMT=Fecal microbiota transplantation; Metro=Metronidazole; Teico=Teicoplanin; Tolev=Tolevamer; Vanco=Vancomycin; Vanco+BL=Vancomycin+bowel lavage


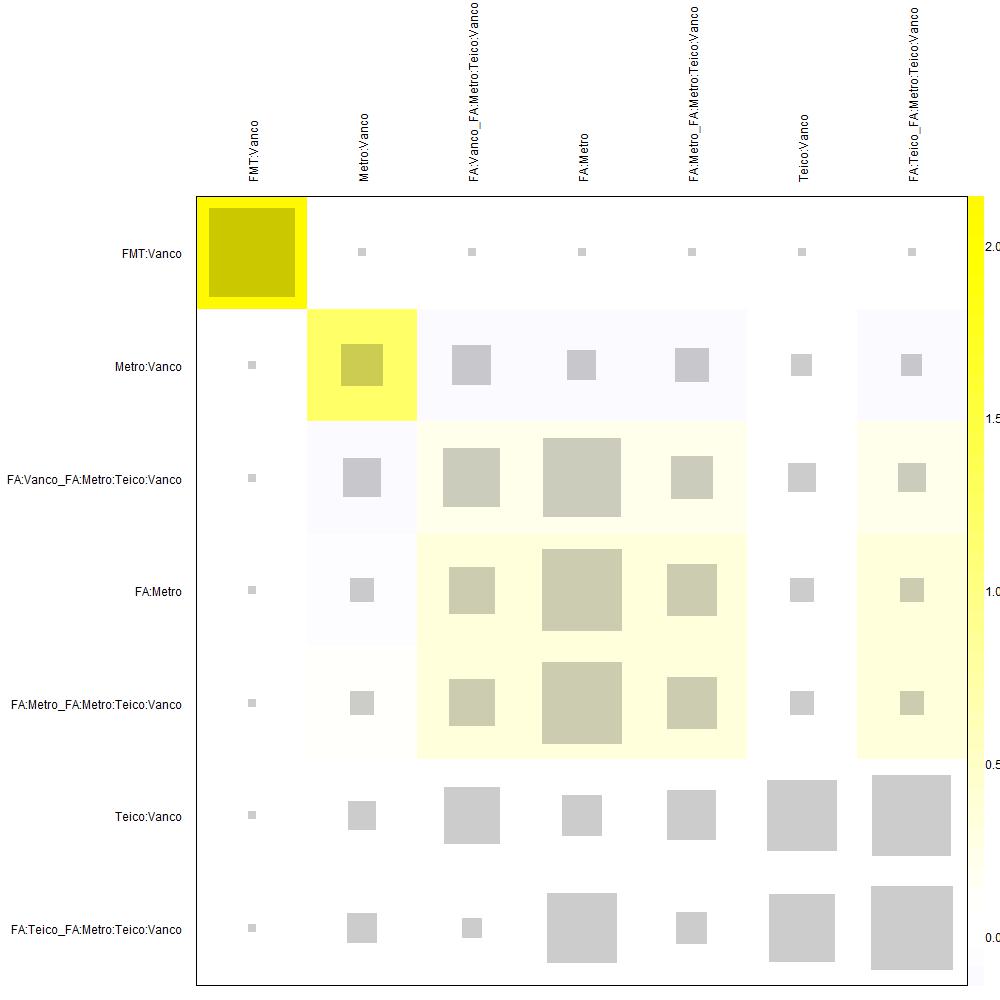


# ***Figure S20:* Net heat plot of the treatments in case of recurrence**

It assesses if there is a consistency problem or not. It shows the difference between direct estimation and the network estimation. The right column shows that the redder the difference, the more inconsistent the result. The area of a gray square represents the contribution of the direct estimate from a design in the column to the network estimate in the row.

FA=Fusidic acid; FMT=Fecal microbiota transplantation; Metro=Metronidazole; Teico=Teicoplanin; Vanco=Vancomycin


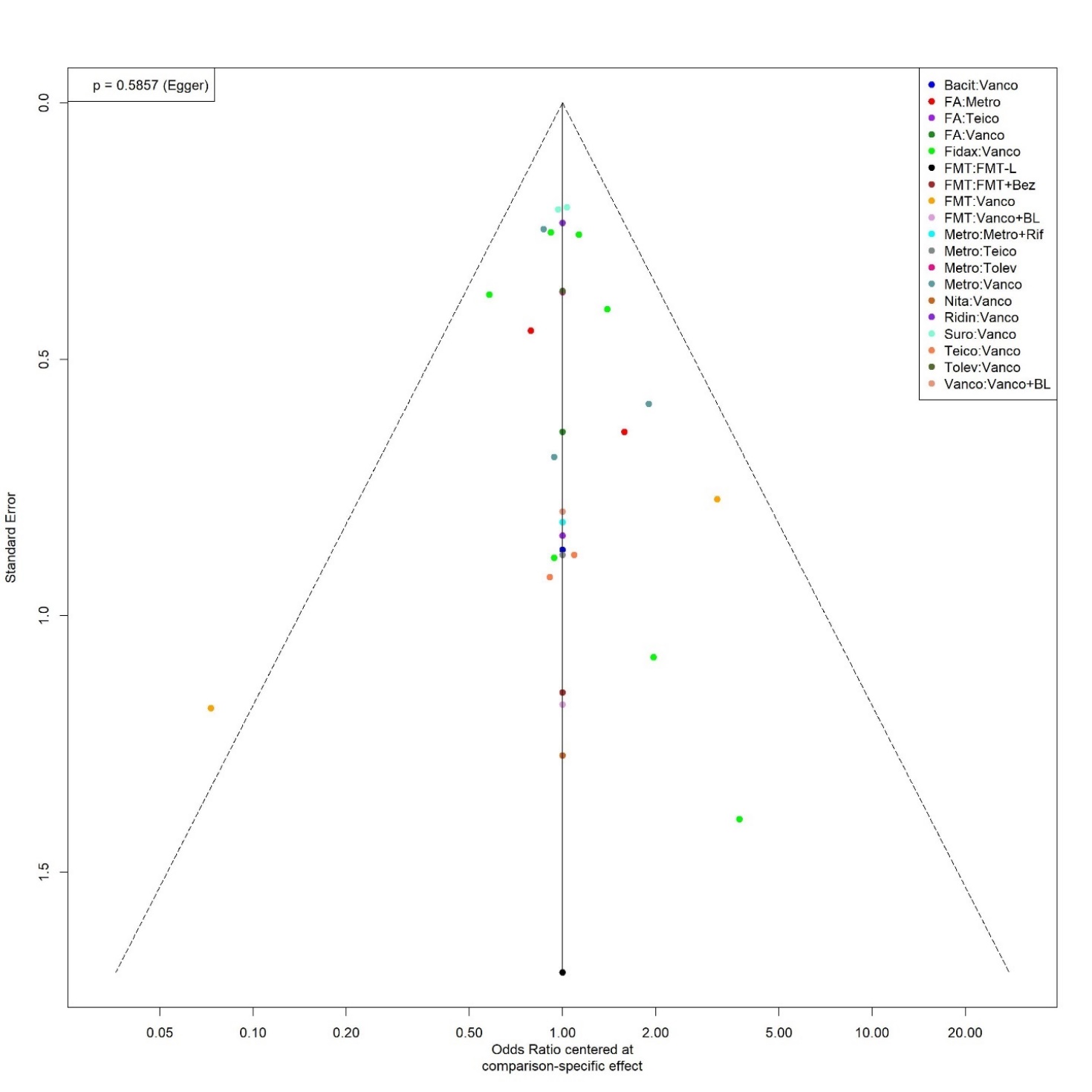


# ***Figure S21:* Funnel plot of the treatments in case of recurrence**

It shows the extent to which the analysis is affected by the small study effect. If the points are under the tent, symmetrically distributed, and the Egger number is bigger than 0.05, in that case the small study effect does not affect our analysis.

Bacit=Bacitracin; FA=Fusidic acid; Fidax=Fidaxomicin; FMT=Fecal microbiota transplantation; FMT-Bez=Fecal microbiota transplantation+Bezlotoxumab; FMT-L=Fecal microbiota transplantation+Lactobacillus; Metro=Metronidazole; Metro+rif=Metronidazole+Rifampin; Nita=Nitazoxanide; Ridin=Ridinilazole; Suro=Surotomycin; Teico=Teicoplanin; Tolev=Tolevamer; Vanco=Vancomycin; Vanco+BL=Vancomycin+bowel lavage

| **Summary of network table** | | | | |
| --- | --- | --- | --- | --- |
| **Characteristic** | | | | **Value** |
| Number of Interventions | | | | 10 |
| Number of Studies | | | | 29 |
| Total Number of Patients in Network | | | | 17 713 |
| Total Possible Pairwise Comparisons | | | | 45 |
| Total Number of Pairwise Comparisons with Direct Data | | | | 13 |
| Number of Two-arm Studies | | | | 27 |
| Number of Multi-Arms Studies | | | | 2 |
| Total Number of Events in Network | | | | 16 554 |
| Number of Studies With No Zero Events | | | | 29 |
| Number of Studies With At Least One Zero Event | | | | 0 |
| Number of Studies With All Zero Events | | | | 0 |
|  |  |  |  | |
| **Treatment** | **Studies (n)** | **Events (n)** | **Patients (n)** | |
| AB | 6 | 470 | 583 | |
| ACT | 1 | 652 | 781 | |
| ACT+BEZ | 1 | 654 | 773 | |
| BEZ | 1 | 172 | 232 | |
| LF | 1 | 7 | 9 | |
| Oligo | 1 | 66 | 72 | |
| Plac | 29 | 6500 | 7043 | |
| Probi | 19 | 3133 | 3257 | |
| RBX | 1 | 148 | 177 | |
| Vac | 1 | 4752 | 4786 | |

# ***Table S18:* Network summary table of the treatments for prevention**

Key data of analysis and treatments.

AB=Antibiotic; ACT=Actoxumab; ACT+BEZ=Actoxumab+Bezlotoxumab; BEZ=Bezlotoxumab; LF=Lactoferrin; Oligo=Oligofructose; Plac=Placebo; Probi=Probiotics; RBX=RBX2660; Vac=Vaccine


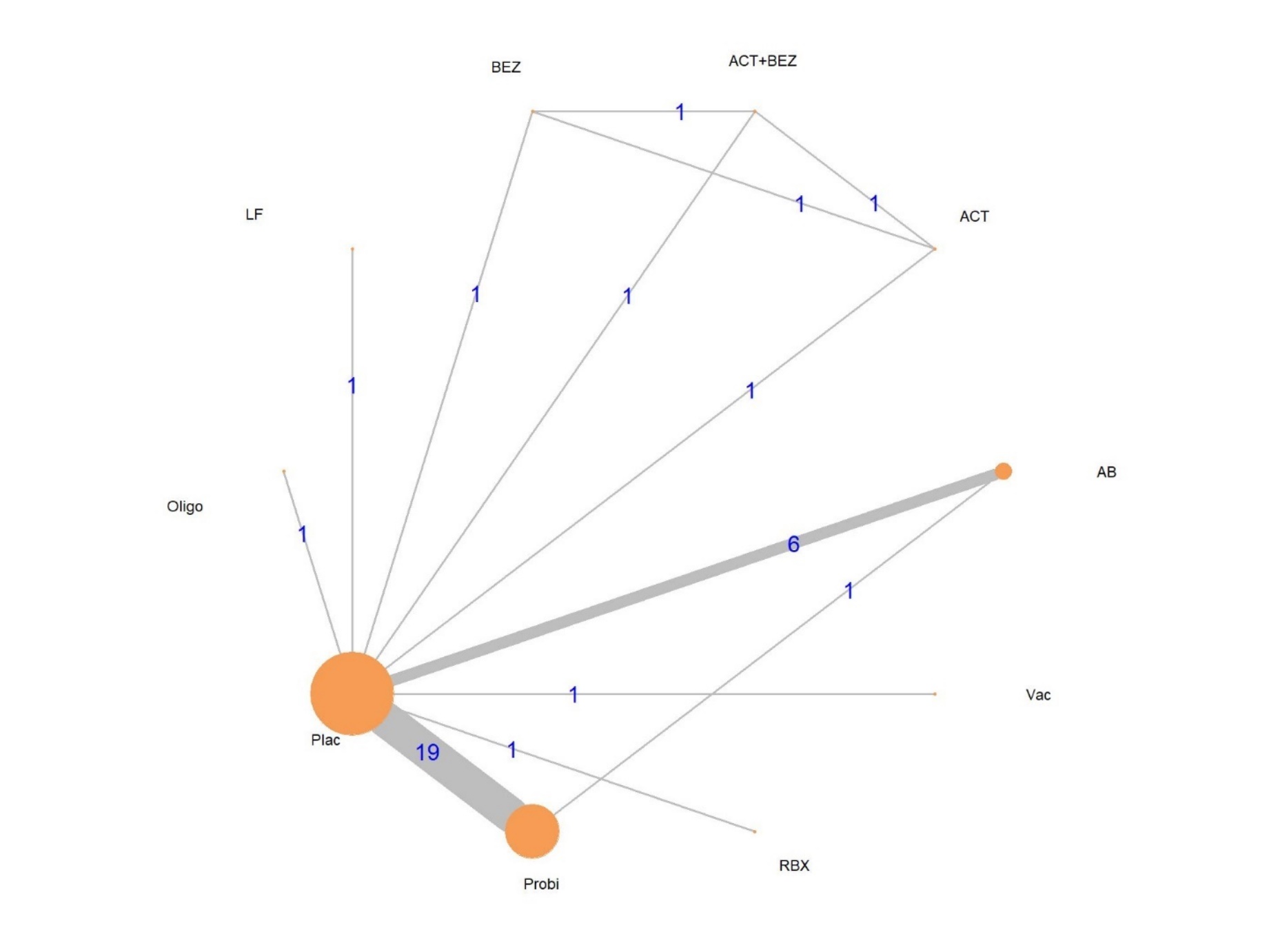


# ***Figure S22:* Network plot of possible treatments for prevention**

Every knot represents a different therapy for CDI. The larger a knot, the more studies included that treatment. Every edge compares different therapies. The width and the number above indicate how many studies investigated this comparison.

AB=Antibiotic; ACT=Actoxumab; ACT+BEZ=Actoxumab+Bezlotoxumab; BEZ=Bezlotoxumab; LF=Lactoferrin; Oligo=Oligofructose; Plac=Placebo; Probi=Probiotics; RBX=RBX2660; Vac=Vaccine

|  | P-score (random) |
| --- | --- |
| Oligo | 0·9582 |
| ACT+BEZ | 0·7024 |
| ACT | 0·6564 |
| AB | 0·5991 |
| Probi | 0·5140 |
| RBX | 0·4493 |
| BEZ | 0·3175 |
| Vac | 0·2959 |
| LF | 0·2572 |
| Plac | 0·2499 |

# ***Table S19:* P-score table (SUCRA) of the treatments in case of prevention**

The possible therapies are ranked based on the P-score. P-score shows the average confidence with which we can say that one treatment is better than another. P-score can range from 0 to 1.

AB=Antibiotic; ACT=Actoxumab; ACT+BEZ=Actoxumab+Bezlotoxumab; BEZ=Bezlotoxumab; LF=Lactoferrin; Oligo=Oligofructose; Plac=Placebo; Probi=Probiotics; RBX=RBX2660; Vac=Vaccine


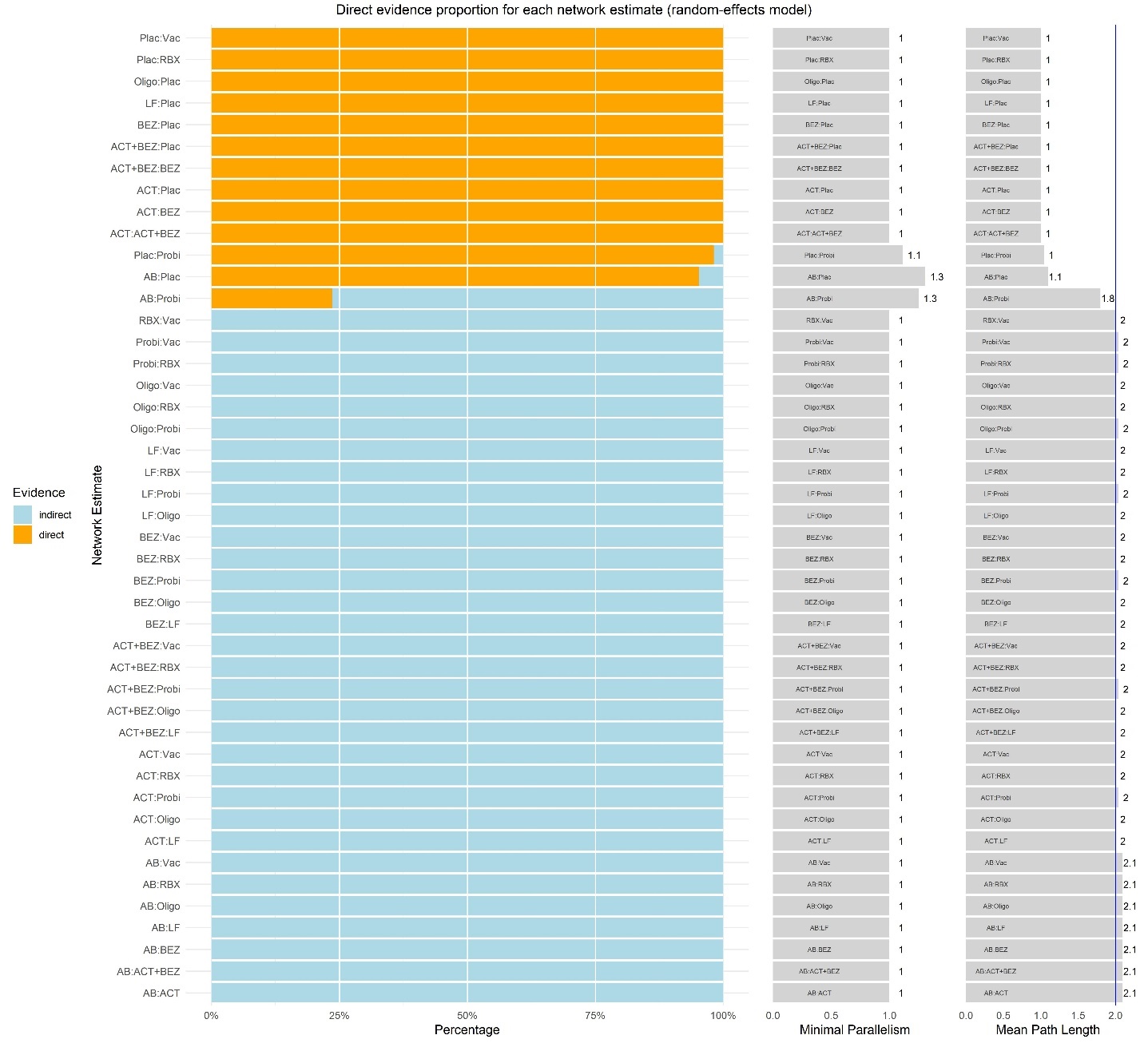


# ***Figure S23:* Evidence plot of the treatments in case of prevention**

It shows what percentage of the result comes from the direct comparison and how much comes from the indirect/estimated data. The direct ones are marked orange, while the estimated ones are marked blue. In the Mean Path Length diagram, if the given comparison is greater than 2, then these network estimations should be interpreted carefully. Higher values of parallelism indicate greater robustness of the estimate.

AB=Antibiotic; ACT=Actoxumab; ACT+BEZ=Actoxumab+Bezlotoxumab; BEZ=Bezlotoxumab; LF=Lactoferrin; Oligo=Oligofructose; Plac=Placebo; Probi=Probiotics; RBX=RBX2660; Vac=Vaccine


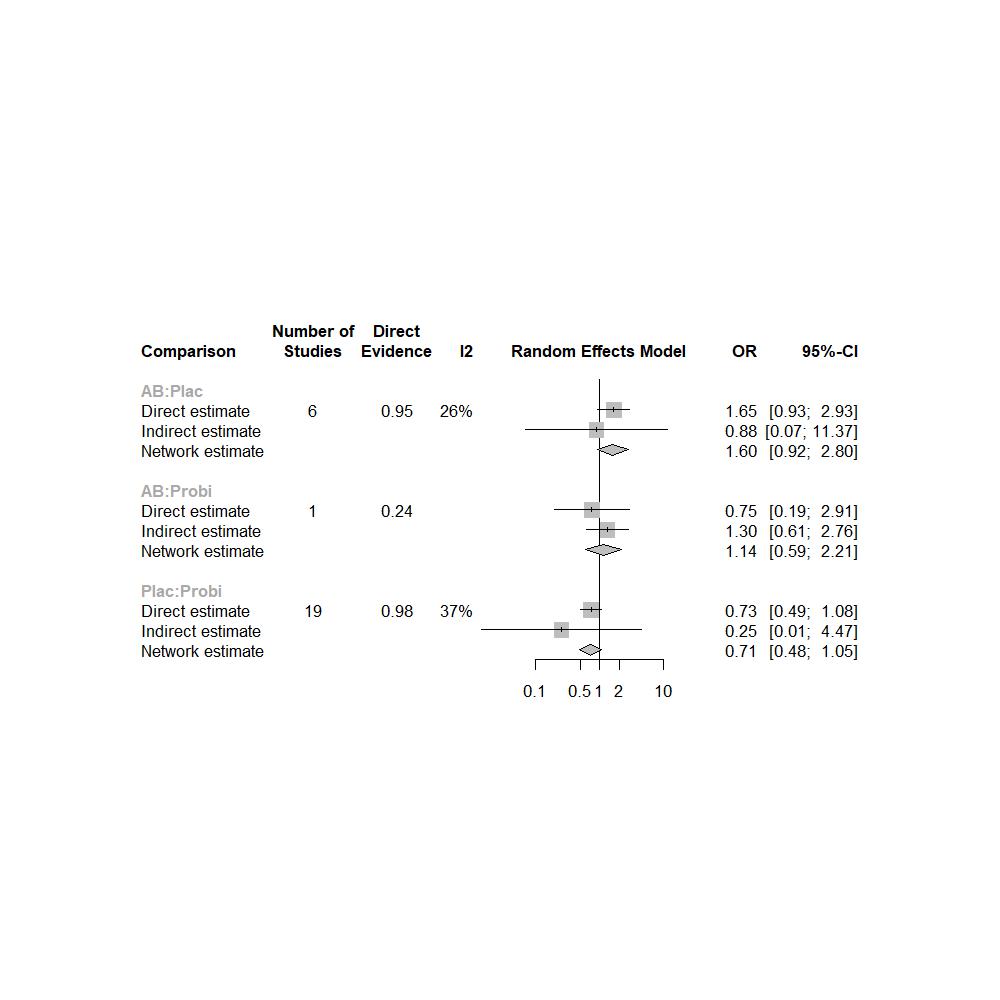


# ***Figure S24:* Forest plot for results of consistency analysis for the treatments in case of prevention**

The Forest plot shows the result of the consistency analysis. The direct and indirect comparisons do not contradict each other, so the network can be considered consistent.

AB=Antibiotic; Plac=Placebo; Probi=Probiotics


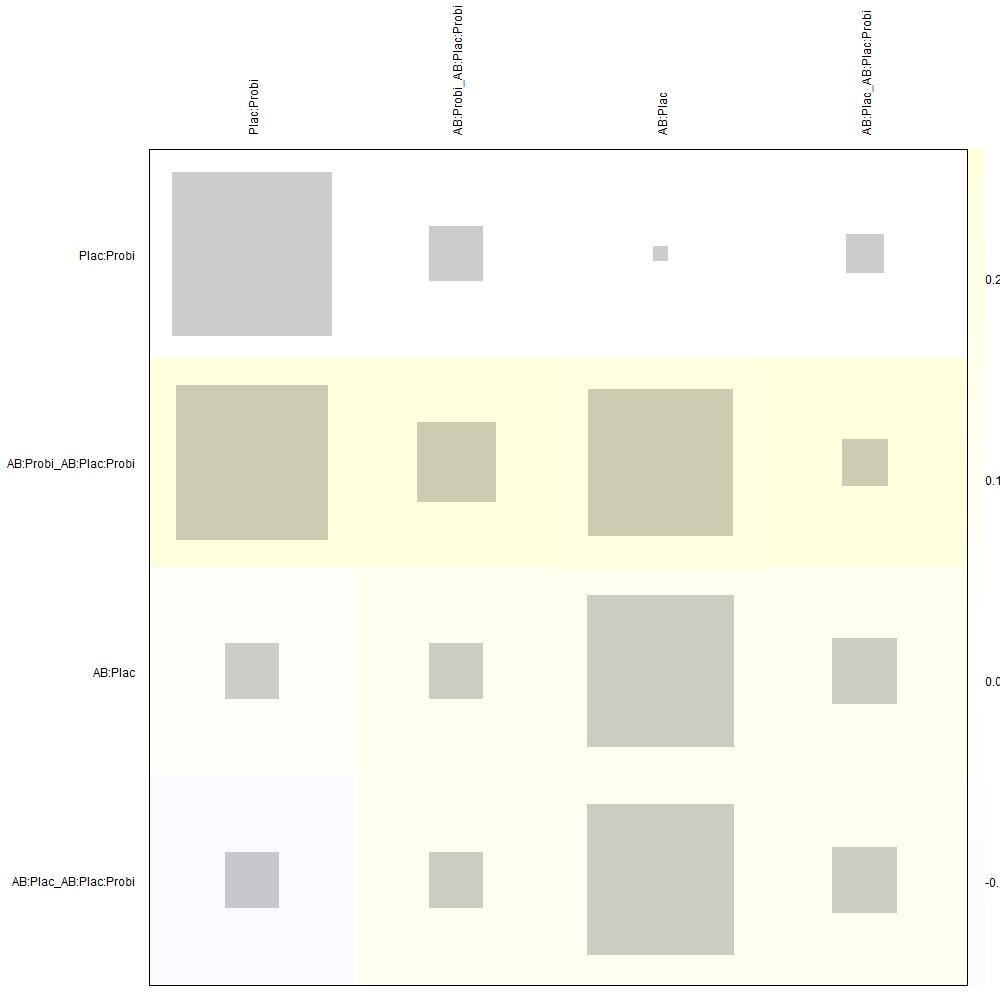


# ***Figure S25:* Net heat plot of the treatments in case of prevention**

It assesses if there is a consistency problem or not. It shows the difference between direct estimation and the network estimation. The right column shows that the redder the difference, the more inconsistent the result. The area of a gray square represents the contribution of the direct estimate from a design in the column to the network estimate in the row.

AB=Antibiotic; Plac=Placebo; Probi=Probiotics


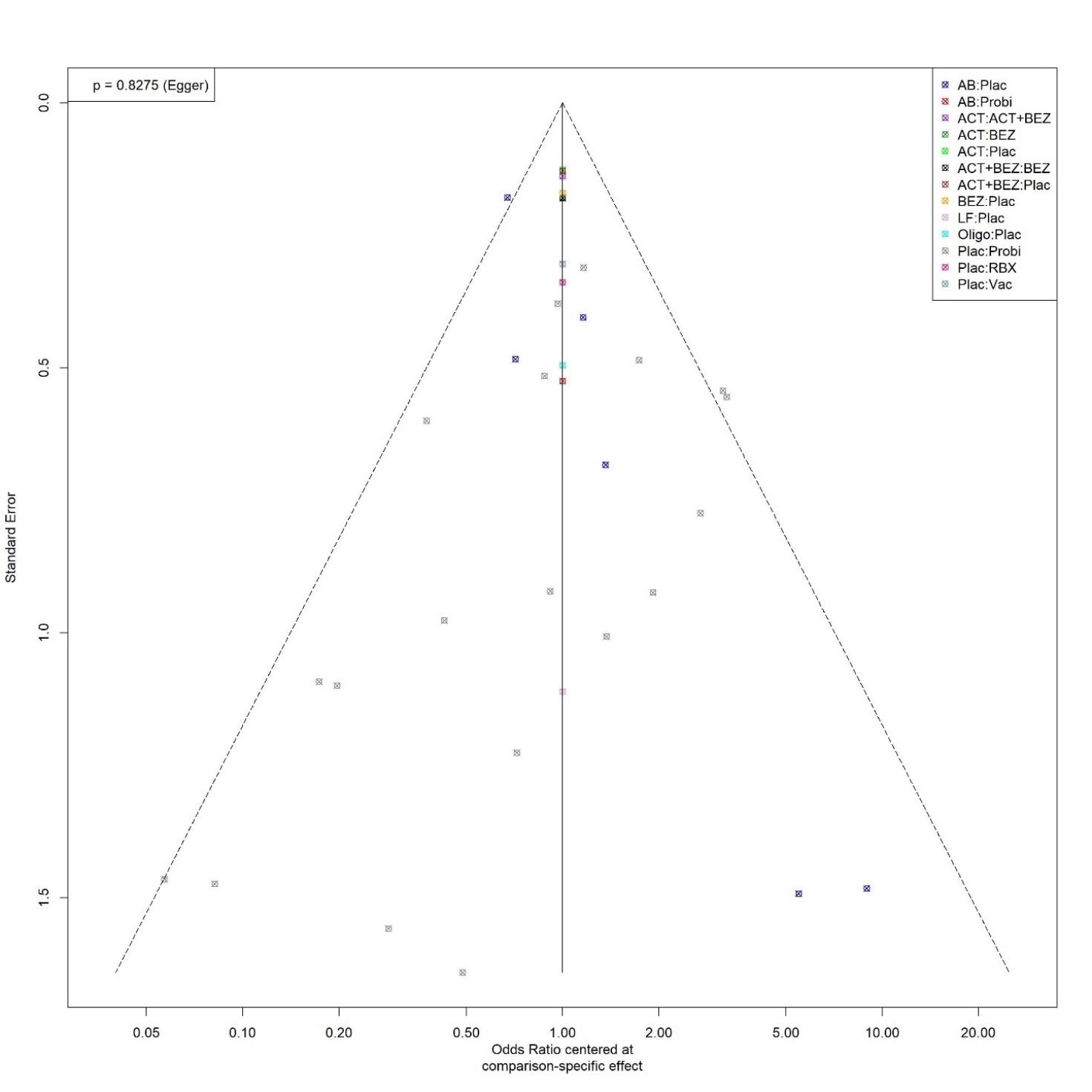


# ***Figure S26:* Funnel plot of the treatments in case of prevention**

It shows the extent to which the analysis is affected by the small study effect. If the points are under the tent, symmetrically distributed, and the Egger number is bigger than 0.05, in that case the small study effect does not affect our analysis.

AB=Antibiotic; ACT=Actoxumab; ACT+BEZ=Actoxumab+Bezlotoxumab; BEZ=Bezlotoxumab; LF=Lactoferrin; Oligo=Oligofructose; Plac=Placebo; Probi=Probiotics; RBX=RBX2660; Vac=Vaccine

# **Supplementary Results S1 – Subgroup analyses of cure rate results by dose of vancomycin treatments**

For the cure rate analysis, we conducted a dose-based subgroup analysis of vancomycin, the most commonly used treatment for CDI. Three different dosing regimens were identified in the trials: 125 mg, 250 mg, and 500 mg, administered four times daily. Based on these, we stratified the previously uniform vancomycin endpoint into three separate categories and re-analyzed the cure rates (overall, recurrent, and non-recurrent).

The results were inconsistent, with extremely wide confidence intervals (results not shown). The 125 mg and 500 mg doses were used in multiple trials with a significant number of cases, whereas the 250 mg dose was tested in only one trial with a very small sample size. Due to this, we excluded the 250 mg trial from the analysis because it caused unstable standard error estimation. We then repeated the analysis after this correction and present the updated results here.

For both the overall cure rate and the analysis of non-recurrent cases, the site-specific substitutions were minor, and the treatment ranking remained unchanged in the analysis of recurrent cases. The 125 mg dose of vancomycin appeared to be more effective than the 500 mg dose in treating CDI, but this result was only statistically significant in non-recurrent cases, and not in the overall or recurrent case analyses.

The detailed results of the analyses are presented in Supplementary Figures S27-S40 and Supplementary Tables S20-S28.

| **Summary of network table** | | | | |
| --- | --- | --- | --- | --- |
| **Characteristic** | | | | **Value** |
| Number of Interventions | | | | 17 |
| Number of Studies | | | | 28 |
| Total Number of Patients in Network | | | | 5638 |
| Total Possible Pairwise Comparisons | | | | 136 |
| Total Number of Pairwise Comparisons with Direct Data | | | | 27 |
| Number of Two-arm Studies | | | | 22 |
| Number of Multi-Arms Studies | | | | 6 |
| Total Number of Events in Network | | | | 4176 |
| Number of Studies With No Zero Events | | | | 29 |
| Number of Studies With At Least One Zero Event | | | | 0 |
| Number of Studies With All Zero Events | | | | 0 |
|  |  |  |  | |
| **Treatment** | **Studies (n)** | **Events (n)** | **Patients (n)** | |
| Bacit | 2 | 23 | 32 | |
| FA | 2 | 76 | 88 | |
| Fidax | 8 | 704 | 803 | |
| FMT | 5 | 97 | 109 | |
| Metro | 8 | 417 | 493 | |
| Metro+rif | 1 | 12 | 19 | |
| Nita | 2 | 85 | 94 | |
| Plac | 3 | 65 | 124 | |
| RBT | 1 | 12 | 25 | |
| Ridin | 1 | 270 | 370 | |
| SER | 1 | 78 | 89 | |
| Suro | 2 | 356 | 575 | |
| Teico | 2 | 52 | 54 | |
| Tolev | 1 | 206 | 440 | |
| Vanco125 | 17 | 1594 | 2159 | |
| Vanco500 | 6 | 126 | 151 | |
| Vanco+BL | 1 | 3 | 13 | |

# ***Table S20:* Network summary table of the treatments for cure rates, vancomycin dosages subgroup analysis**

Key data of analysis and treatments.

Bacit=Bacitracin; FA=Fusidic acid; Fidax=Fidaxomicin; FMT=Fecal microbiota transplantation; Metro=Metronidazole; Metro+rif=Metronidazole+Rifampin; Nita=Nitazoxanide; Plac=Placebo; RBT=Rectal bacteriotherapy; Ridin=Ridinilazole; SER=SER 109; Suro=Surotomycin; Teico=Teicoplanin; Tolev=Tolevamer; Vanco125=Vancomycin 125 mg four times daily; Vanco500=Vancomycin 500 mg four times daily; Vanco+BL=Vancomycin+bowel lavage


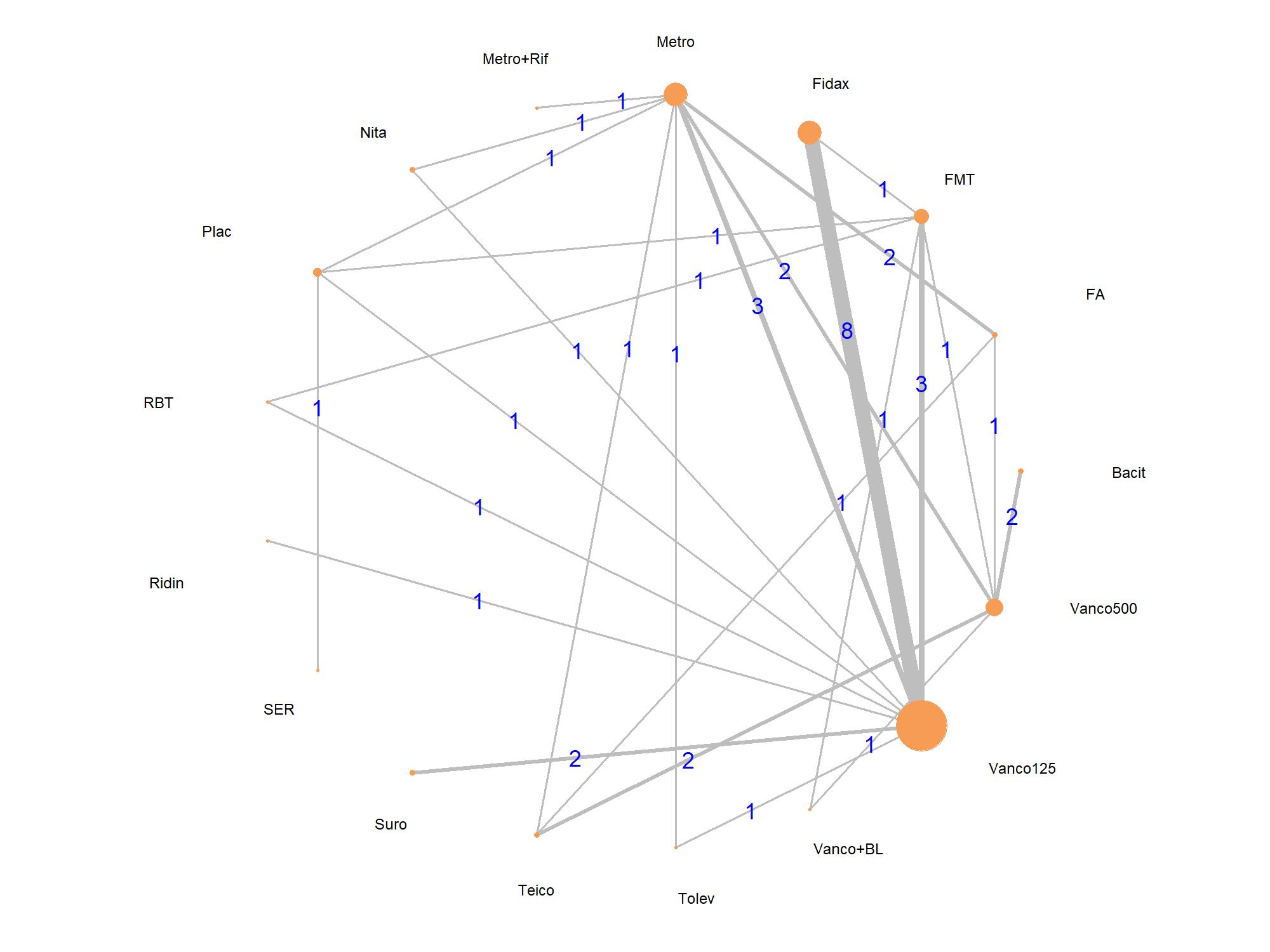


# ***Figure S27:* Network plot of possible treatments for cure rate, vancomycin dosages subgroup analysis**

Every knot represents a different therapy for CDI. The larger the knot, the more studies included the treatment. Every edge compares different therapies. The width and the number above indicate how many studies investigated this comparison.

Bacit=Bacitracin; FA=Fusidic acid; Fidax=Fidaxomicin; FMT=Fecal microbiota transplantation; Metro=Metronidazole; Metro+rif=Metronidazole+Rifampin; Nita=Nitazoxanide; Plac=Placebo; RBT=Rectal bacteriotherapy; Ridin=Ridinilazole; SER=SER 109; Suro=Surotomycin; Teico=Teicoplanin; Tolev=Tolevamer; Vanco125=Vancomycin 125 mg four times daily; Vanco500=Vancomycin 500 mg four times daily; Vanco+BL=Vancomycin+bowel lavage

|  | P-score (random) |
| --- | --- |
| FMT | 0·9982 |
| Fidax | 0·7881 |
| RBT | 0·7177 |
| Nita | 0·7175 |
| SER | 0·7131 |
| Ridin | 0·6183 |
| Suro | 0·6065 |
| Vanco125 | 0·5731 |
| Metro | 0·4418 |
| Metro+rif | 0·4383 |
| Teico | 0·44381 |
| Vanco500 | 0·3843 |
| Vanco+BL | 0·2652 |
| Bacit | 0·2593 |
| Plac | 0·2450 |
| FA | 0·2348 |
| Tolev | 0·0607 |

# ***Table S21:* P-score table (SUCRA) of the treatments in case of cure rate, vancomycin dosages subgroup analysis**

The possible therapies are ranked based on the P-score. P-score shows the average confidence with which we can say that one treatment is better than another. P-score can range from 0 to 1.

Bacit=Bacitracin; FA=Fusidic acid; Fidax=Fidaxomicin; FMT=Fecal microbiota transplantation; Metro=Metronidazole; Metro+rif=Metronidazole+Rifampin; Nita=Nitazoxanide; Plac=Placebo; RBT=Rectal bacteriotherapy; Ridin=Ridinilazole; SER=SER 109; Suro=Surotomycin; Teico=Teicoplanin; Tolev=Tolevamer; Vanco125=Vancomycin 125 mg four times daily; Vanco500=Vancomycin 500 mg four times daily; Vanco+BL=Vancomycin+bowel lavage

| FMT | 15·40  [2·38; 99·69] | 4·98  [1·10; 22·60] | ·· | ·· | ·· | ·· | 14·07  [5·03; 39·40] | ·· | ·· | ·· | 33·75  [2·79;408·63] | 50·00  [3·91;639·62] | ·· | 19·00  [2·79;129·40] | ·· | ·· |
| --- | --- | --- | --- | --- | --- | --- | --- | --- | --- | --- | --- | --- | --- | --- | --- | --- |
| 7·54  [2·86; 19·85] | Fidax | ·· | ·· | ·· | ·· | ·· | 1·74  [1·09; 2·77] | ·· | ·· | ·· | ·· | ·· | ·· | ·· | ·· | ·· |
| 8·11  [2·13; 30·90] | 1·08  [0·28; 4·14] | RBT | ·· | ·· | ·· | ·· | 1·12  [0·29; 4·37] | ·· | ·· | ·· | ·· | ·· | ·· | ·· | ·· | ·· |
| 8·22  [1·68; 40·18] | 1·09  [0·26; 4·60] | 1·01  [0·16; 6·48] | Nita | ·· | ·· | ·· | 2·55  [0·21; 31·22] | 1·82  [0·44; 7·62] | ·· | ·· | ·· | ·· | ·· | ·· | ·· | ·· |
| 7·54  [1·17; 48·66] | 1·00  [0·14; 6·97] | 0·93  [0·10; 8·43] | 0·92  [0·10; 8·80] | SER | ·· | ·· | ·· | ·· | ·· | ·· | ·· | ·· | ·· | 4·69  [1·49; 14·69] | ·· | ·· |
| 11·22  [3·13; 40·24] | 1·49  [0·53; 4·14] | 1·38  [0·29; 6·65] | 1·36  [0·26; 7·06] | 1·49 [ 0·18; 12·22] | Ridin | ·· | 1·12  [0·45; 2·80] | ·· | ·· | ·· | ·· | ·· | ·· | ·· | ·· | ·· |
| 11·67  [3·87; 35·20] | 1·55  [0·70; 3·44] | 1·44  [0·34; 6·03] | 1·42  [0·31; 6·44] | 1·55 [ 0·21; 11·50] | 1·04 [ 0·34; 3·20] | Suro | 1·08  [0·56; 2·07] | ·· | ·· | ·· | ·· | ·· | ·· | ·· | ·· | ·· |
| 12·57  [5·15; 30·66] | 1·67  [1·05; 2·64] | 1·55  [0·43; 5·56] | 1·53  [0·39; 5·99] | 1·67 [ 0·25; 11·11] | 1·12 [ 0·45; 2·80] | 1·08 [ 0·56; 2·07] | Vanco125 | 1·53  [0·68; 3·47] | ·· | ·· | ·· | ·· | ·· | 36·00  [2·37;547·02] | ·· | 4·42  [1·74; 11·25] |
| 17·70  [6·00; 52·19] | 2·35  [0·98; 5·65] | 2·18  [0·51; 9·36] | 2·15  [0·61; 7·56] | 2·35 [ 0·34; 16·09] | 1·58 [ 0·48; 5·16] | 1·52 [ 0·56; 4·10] | 1·41 [ 0·66; 2·99] | Metro | 1·08  [0·23; 5·18] | 0·54  [0·04; 7·25] | 1·10  [0·32; 3·80] | ·· | ·· | 1·71  [0·18; 15·91] | 1·97  [0·57; 6·78] | 6·74  [2·60; 17·51] |
| 19·17  [2·86; 128·44] | 2·54  [0·42; 15·29] | 2·36  [0·28; 20·04] | 2·33  [0·31; 17·34] | 2·54 [ 0·21; 30·38] | 1·71 [ 0·24; 12·16] | 1·64 [ 0·26; 10·49] | 1·53 [ 0·27; 8·65] | 1·08 [ 0·23; 5·18] | Metro+Rif | ·· | ·· | ·· | ·· | ·· | ·· | ·· |
| 19·81  [2·14; 182·91] | 2·63  [0·30; 22·89] | 2·44  [0·21; 28·19] | 2·41  [0·23; 25·56] | 2·63 [ 0·17; 41·22] | 1·77 [ 0·18; 17·75] | 1·70 [ 0·19; 15·57] | 1·58 [ 0·19; 13·11] | 1·12 [ 0·15; 8·32] | 1·03 [ 0·08; 13·15] | Teico | 1·06  [0·14; 8·31] | ·· | ·· | ·· | 2·00  [0·15; 27·04] | ·· |
| 22·08  [5·52; 88·23] | 2·93  [0·78; 11·05] | 2·72  [0·48; 15·49] | 2·68  [0·52; 13·95] | 2·93 [ 0·34; 25·17] | 1·97 [ 0·42; 9·29] | 1·89 [ 0·46; 7·77] | 1·76 [ 0·50; 6·15] | 1·25 [ 0·42; 3·69] | 1·15 [ 0·17; 7·73] | 1·11 [ 0·16; 7·86] | Vanco500 | 1·48  [0·21; 10·37] | 1·61  [0·41; 6·31] | ·· | 1·07  [0·12; 9·72] | ·· |
| 36·87  [4·66; 291·85] | 4·89  [0·60; 40·17] | 4·55  [0·43; 48·24] | 4·48  [0·42; 47·40] | 4·89 [ 0·33; 71·64] | 3·29 [ 0·34; 31·39] | 3·16 [ 0·36; 27·48] | 2·93 [ 0·37; 23·07] | 2·08 [ 0·28; 15·69] | 1·92 [ 0·15; 24·74] | 1·86 [ 0·13; 26·40] | 1·67 [ 0·26; 10·68] | Vanco+BL | ·· | ·· | ·· | ·· |
| 35·56  [5·09; 248·62] | 4·72  [0·70; 31·68] | 4·39  [0·48; 39·99] | 4·33  [0·51; 36·73] | 4·72 [ 0·37; 60·26] | 3·17 [ 0·40; 25·04] | 3·05 [ 0·43; 21·72] | 2·83 [ 0·44; 18·05] | 2·01 [ 0·35; 11·49] | 1·85 [ 0·18; 19·31] | 1·80 [ 0·17; 19·45] | 1·61 [ 0·41; 6·31] | 0·96 [ 0·10; 9·66] | Bacit | ·· | ·· | ·· |
| 35·31  [8·09; 154·22] | 4·68  [0·98; 22·51] | 4·35  [0·66; 28·71] | 4·29  [0·61; 30·25] | 4·69 [ 1·49; 14·69] | 3·15 [ 0·54; 18·46] | 3·03 [ 0·58; 15·72] | 2·81 [ 0·62; 12·76] | 2·00 [ 0·42; 9·39] | 1·84 [ 0·20; 16·64] | 1·78 [ 0·15; 21·82] | 1·60 [ 0·26; 9·90] | 0·96 [ 0·08; 10·87] | 0·99 [ 0·10; 9·67] | Plac | ·· | ·· |
| 35·72  [7·34; 173·74] | 4·74  [1·09; 20·56] | 4·40  [0·68; 28·48] | 4·34  [0·77; 24·49] | 4·74 [ 0·50; 45·06] | 3·18 [ 0·60; 16·91] | 3·06 [ 0·66; 14·29] | 2·84 [ 0·70; 11·49] | 2·02 [ 0·61; 6·65] | 1·86 [ 0·26; 13·32] | 1·80 [ 0·21; 15·16] | 1·62 [ 0·38; 6·92] | 0·97 [ 0·10; 9·32] | 1·00 [ 0·14; 7·37] | 1·01 [ 0·15; 7·05] | FA | ·· |
| 80·28  [23·78; 271·03] | 10·65  [3·95; 28·74] | 9·90  [2·12; 46·17] | 9·76  [2·19; 43·51] | 10·65 [ 1·39; 81·75] | 7·16 [ 2·01; 25·49] | 6·88 [ 2·30; 20·58] | 6·39 [ 2·64; 15·42] | 4·54 [ 1·86; 11·09] | 4·19 [ 0·69; 25·37] | 4·05 [ 0·46; 36·01] | 3·64 [ 0·92; 14·37] | 2·18 [ 0·25; 18·92] | 2·26 [ 0·33; 15·65] | 2·27 [ 0·42; 12·29] | 2·25 [ 0·51; 9·91] | Tolev |

# ***Table S22:* League table of possible treatments in cure rate, vancomycin dosages subgroup analysis**

Possible treatments are arranged in descending order of the P score in the blue bars. P scores are given in brackets after treatment names. Direct comparisons are shown above these blue bars, whereas below, the direct and indirect (estimated) ones are pooled together as network estimates. Odds ratios are given in the cells. We compare one treatment on the left side with another one on the right side, indicating a greater odds ratio of recovery. The 95% confidence interval is shown in brackets. Significant results are marked in green.

Bacit=Bacitracin; FA=Fusidic acid; Fidax=Fidaxomicin; FMT=Fecal microbiota transplantation; Metro=Metronidazole; Metro+rif=Metronidazole+Rifampin; Nita=Nitazoxanide; Plac=Placebo; RBT=Rectal bacteriotherapy; Ridin=Ridinilazole; SER=SER 109; Suro=Surotomycin; Teico=Teicoplanin; Tolev=Tolevamer; Vanco125=Vancomycin 125 mg four times daily; Vanco500=Vancomycin 500 mg four times daily; Vanco+BL=Vancomycin+bowel lavage


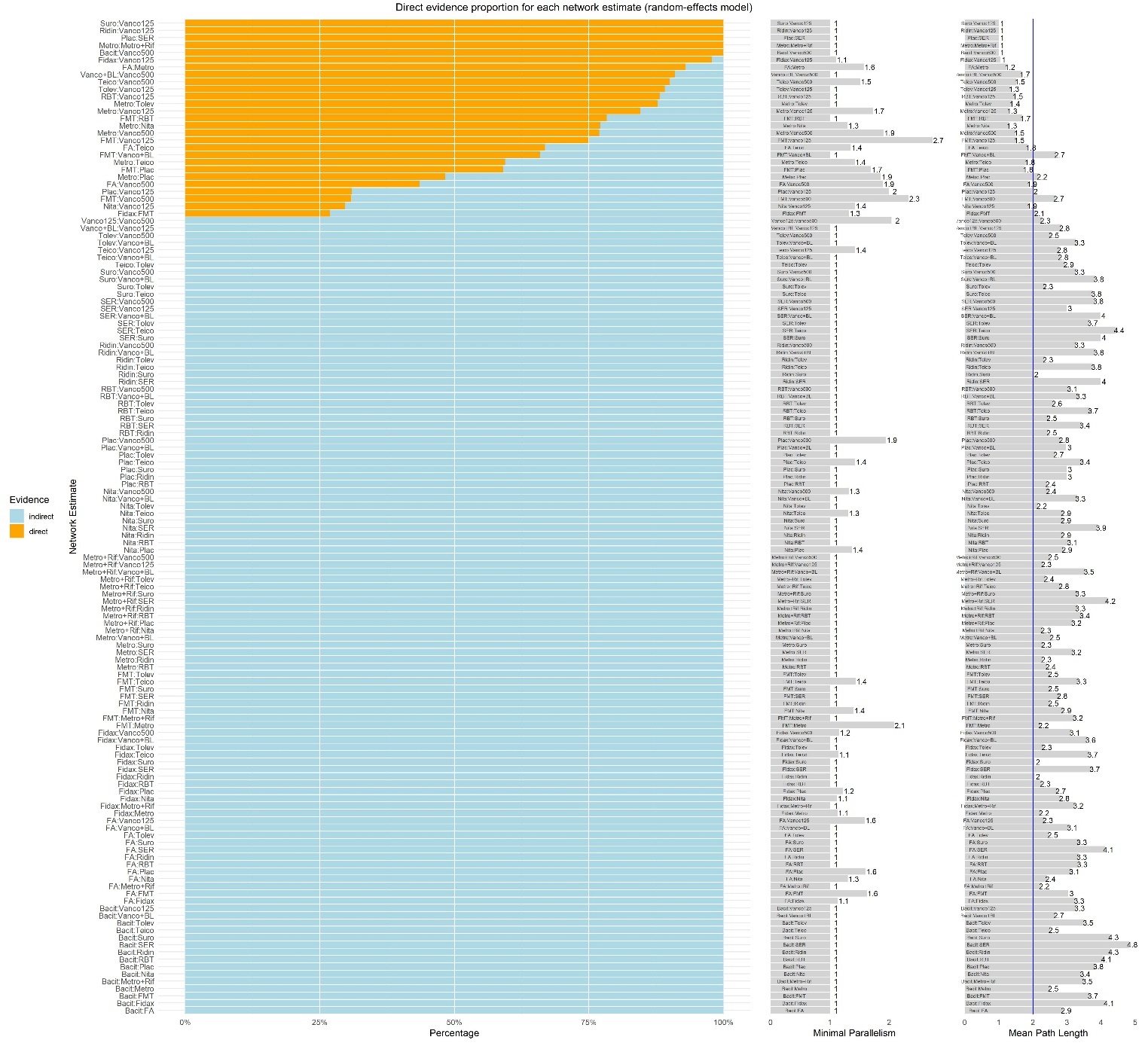


# ***Figure S28:* Evidence plot of the treatments in case of cure rate, vancomycin dosages subgroup analysis**

It shows what percentage of the result comes from the direct comparison and how much comes from the indirect/estimated data. The direct ones are marked orange, while the estimated ones are marked blue. In the Mean Path Length diagram, if the given comparison is greater than 2, then these network estimations should be interpreted carefully. Higher values of parallelism indicate greater robustness of the estimate.

Bacit=Bacitracin; FA=Fusidic acid; Fidax=Fidaxomicin; FMT=Fecal microbiota transplantation; Metro=Metronidazole; Metro+rif=Metronidazole+Rifampin; Nita=Nitazoxanide; Plac=Placebo; RBT=Rectal bacteriotherapy; Ridin=Ridinilazole; SER=SER 109; Suro=Surotomycin; Teico=Teicoplanin; Tolev=Tolevamer; Vanco125=Vancomycin 125 mg four times daily; Vanco500=Vancomycin 500 mg four times daily; Vanco+BL=Vancomycin+bowel lavage


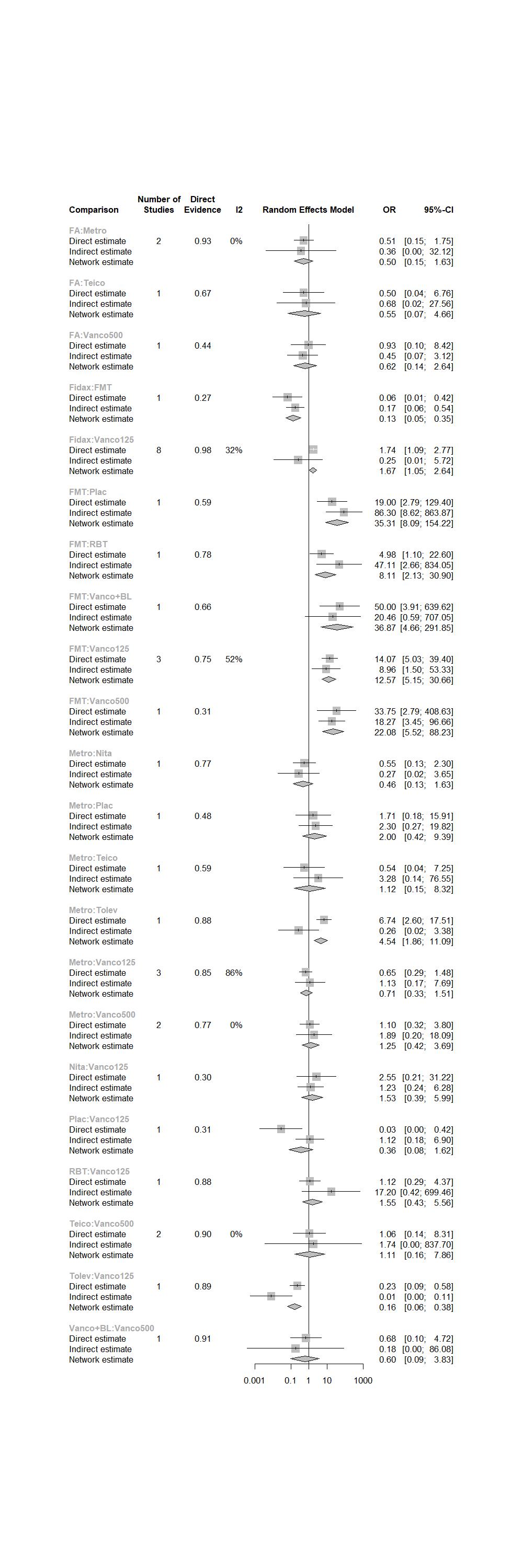


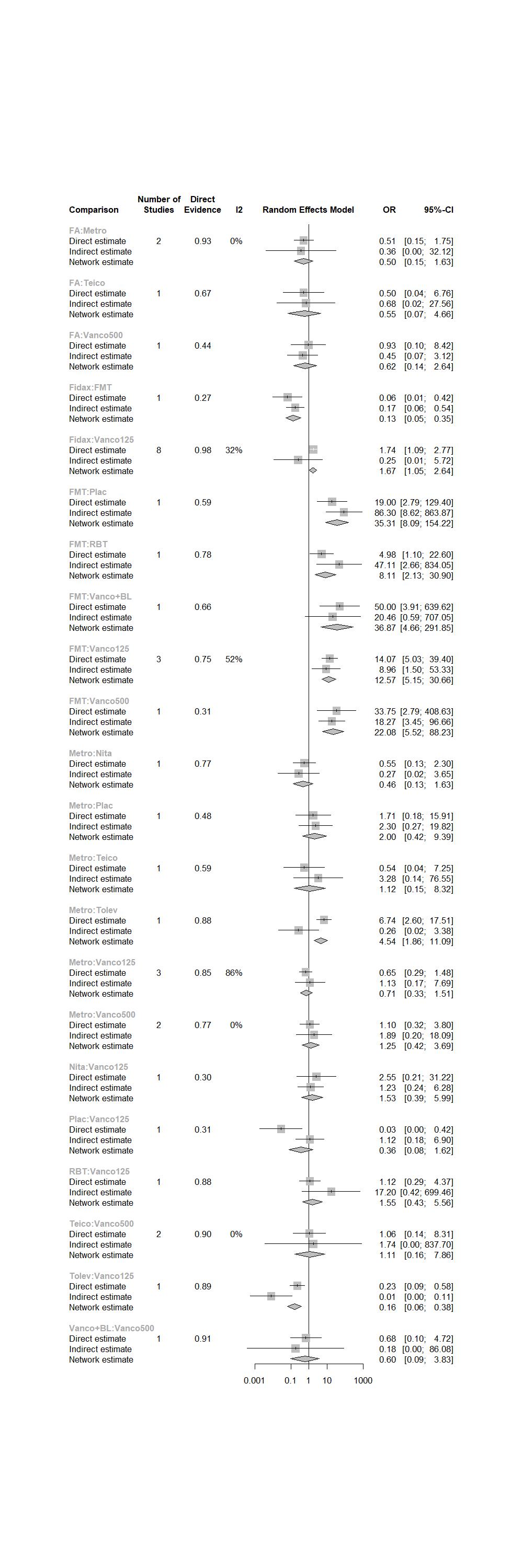


# ***Figure S29:* Forest plot for results of consistency analysis for the treatments in case of cure rate**

The Forest plot shows the result of the consistency analysis. The direct and indirect comparisons do not contradict each other, so the network can be considered consistent.

FA=Fusidic acid; Fidax=Fidaxomicin; FMT=Fecal microbiota transplantation; Metro=Metronidazole; Nita=Nitazoxanide; Plac=Placebo; RBT=Rectal bacteriotherapy; Teico=Teicoplanin; Tolev=Tolevamer; Vanco125=Vancomycin 125 mg four times daily; Vanco500=Vancomycin 500 mg four times daily; Vanco+BL=Vancomycin+bowel lavage


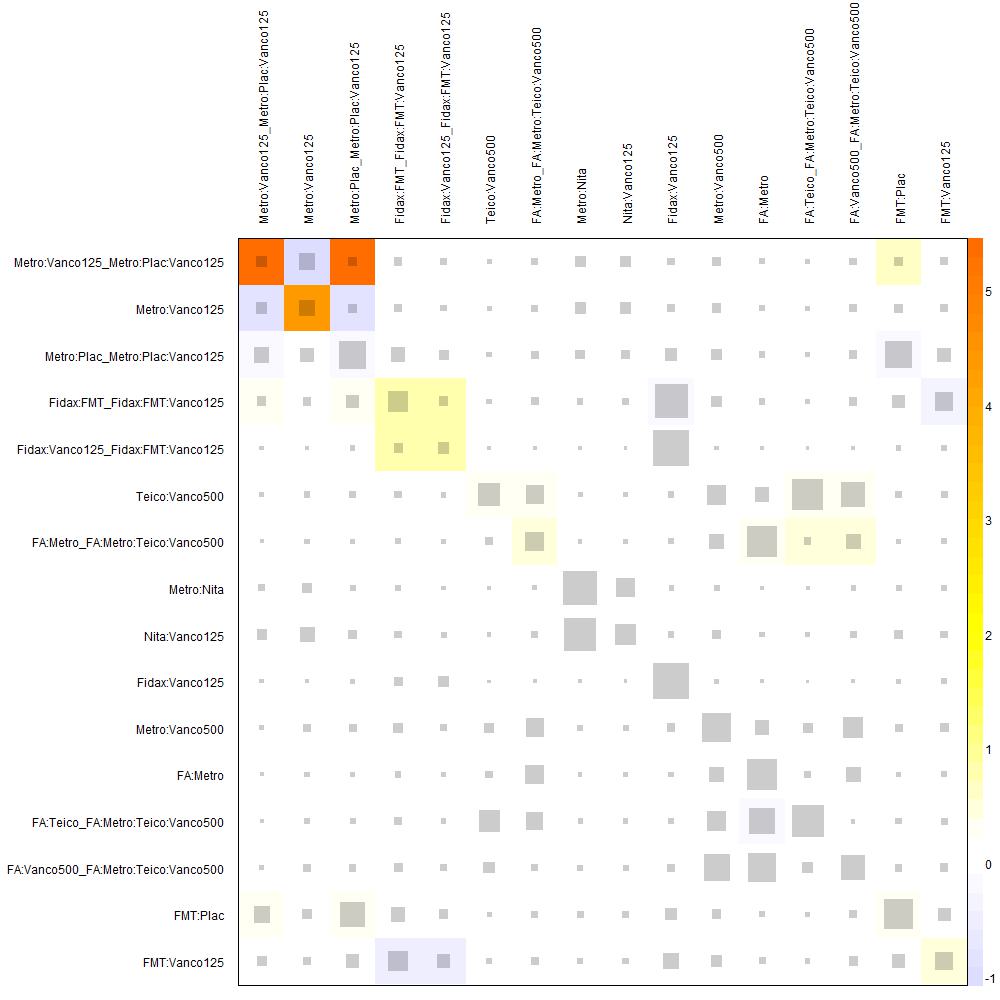


# ***Figure S30:* Net heat plot of the treatments in case of cure rate, vancomycin dosages subgroup analysis**

It assesses if there is a consistency problem or not. It shows the difference between direct estimation and the network estimation. The right column shows that the redder the difference, the more inconsistent the result. The area of a gray square represents the contribution of the direct estimate from a design in the column to the network estimate in the row.

FA=Fusidic acid; Fidax=Fidaxomicin; FMT=Fecal microbiota transplantation; Metro=Metronidazole; Nita=Nitazoxanide; Plac=Placebo; Teico=Teicoplanin; Tolev=Tolevamer; Vanco125=Vancomycin 125 mg four times daily; Vanco500=Vancomycin 500 mg four times daily


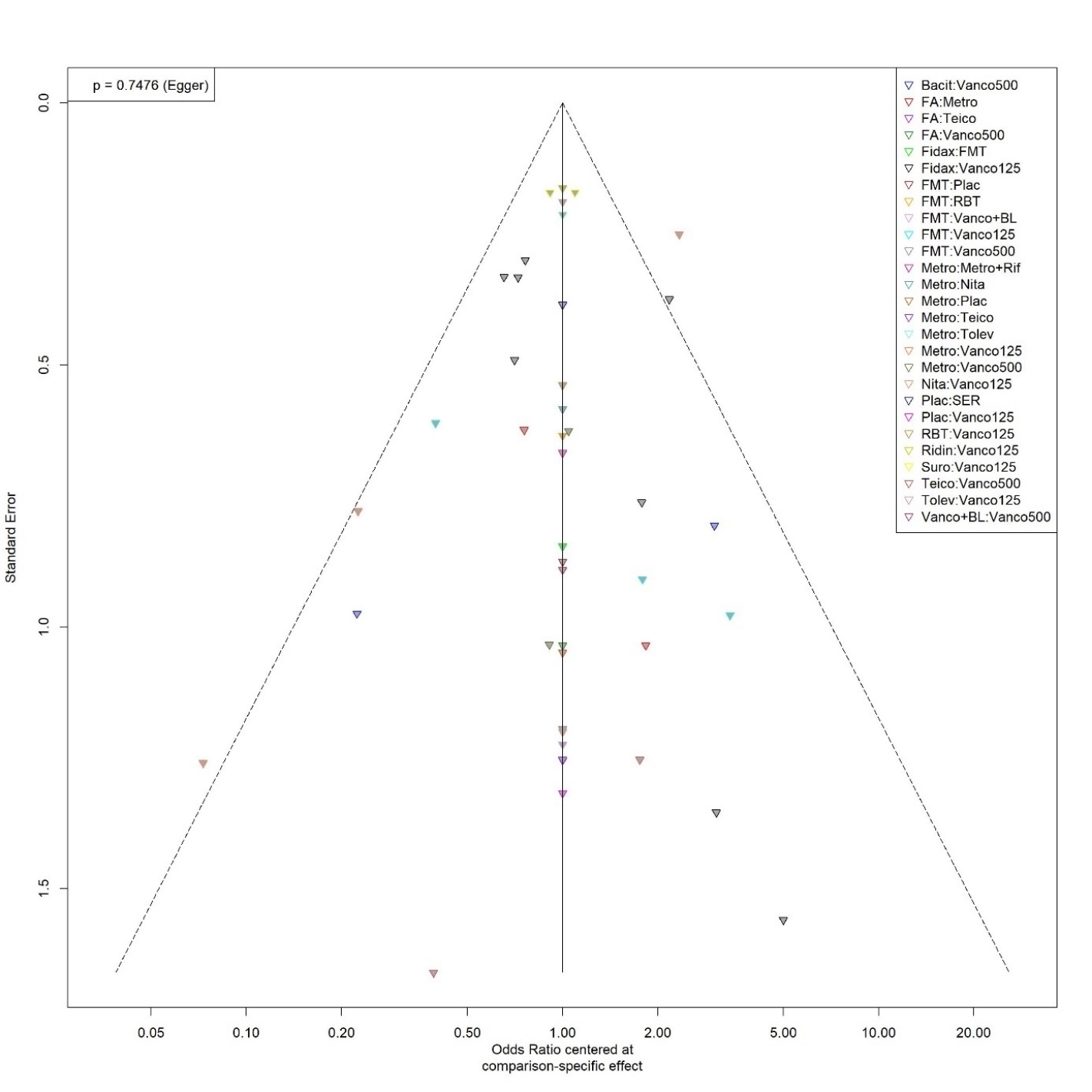


# ***Figure S31:* Funnel plot of the treatments in case of cure rate, vancomycin dosages subgroup analysis**

It shows the extent to which the analysis is affected by the small study effect. If the points are under the tent, symmetrically distributed, and the Egger number is bigger than 0.05, in that case the small study effect does not affect our analysis.

Bacit=Bacitracin; FA=Fusidic acid; Fidax=Fidaxomicin; FMT=Fecal microbiota transplantation; FMT+Vanco=FMT+Vancomycin; Metro=Metronidazole; Metro+rif=Metronidazole+Rifampin; Nita=Nitazoxanide; Plac=Placebo; RBT=Rectal bacteriotherapy; Ridin=Ridinilazole; SER=SER 109; Suro=Surotomycin Teico=Teicoplanin; Tolev=Tolevamer; Vanco125=Vancomycin 125 mg four times daily; Vanco500=Vancomycin 500 mg four times daily; Vanco+BL=Vancomycin+bowel lavage

| **Summary of network table** | | | | |
| --- | --- | --- | --- | --- |
| **Characteristic** | | | | **Value** |
| Number of Interventions | | | | 12 |
| Number of Studies | | | | 10 |
| Total Number of Patients in Network | | | | 2283 |
| Total Possible Pairwise Comparisons | | | | 66 |
| Total Number of Pairwise Comparisons with Direct Data | | | | 19 |
| Number of Two-arm Studies | | | | 5 |
| Number of Multi-Arms Studies | | | | 5 |
| Total Number of Events in Network | | | | 1541 |
| Number of Studies With No Zero Events | | | | 10 |
| Number of Studies With At Least One Zero Event | | | | 0 |
| Number of Studies With All Zero Events | | | | 0 |
|  |  |  |  | |
| **Treatment** | **Studies (n)** | **Events (n)** | **Patients (n)** | |
| FA | 1 | 27 | 29 | |
| Fidax | 3 | 189 | 236 | |
| FMT | 4 | 78 | 88 | |
| Metro | 2 | 219 | 236 | |
| Nita | 1 | 17 | 18 | |
| RBT | 1 | 12 | 25 | |
| Suro | 1 | 176 | 290 | |
| Teico | 1 | 27 | 28 | |
| Tolev | 1 | 206 | 440 | |
| Vanco125 | 8 | 554 | 819 | |
| Vanco500 | 2 | 33 | 44 | |
| Vanco+BL | 1 | 3 | 13 | |

# ***Table S23:* Network summary table of the treatments for cure rates in recurrent cases, vancomycin dosages subgroup analysis**

Key data of analysis and treatments.

FA=Fusidic acid; Fidax=Fidaxomicin; FMT=Fecal microbiota transplantation; Metro=Metronidazole; Nita=Nitazoxanide; RBT=Rectal bacteriotherapy; Suro=Surotomycin; Teico=Teicoplanin; Tolev=Tolevamer; Vanco125=Vancomycin 125 mg four times daily; Vanco500=Vancomycin 500 mg four times daily; Vanco+BL=Vancomycin+bowel lavage


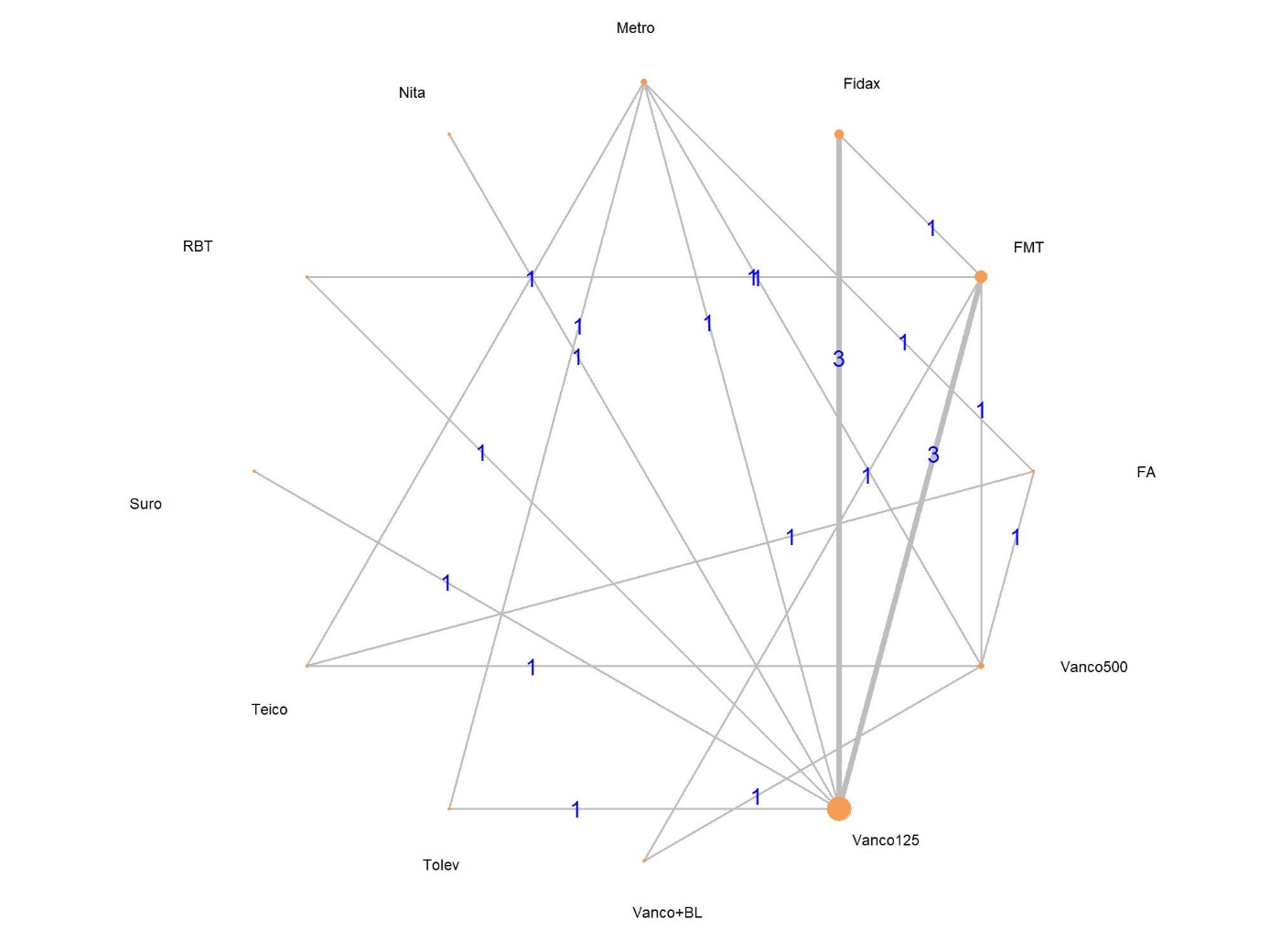


# ***Figure S32:* Network plot of possible treatments for cure rates in recurrent cases, vancomycin dosages subgroup analysis**

Every knot represents a different therapy for CDI. The larger a knot, the more studies included that treatment. Every edge compares different therapies. The width and the number above indicate how many studies investigated this comparison.

FA=Fusidic acid; Fidax=Fidaxomicin; FMT=Fecal microbiota transplantation; Metro=Metronidazole; Nita=Nitazoxanide; RBT=Rectal bacteriotherapy; Suro=Surotomycin; Teico=Teicoplanin; Tolev=Tolevamer; Vanco125=Vancomycin 125 mg four times daily; Vanco500=Vancomycin 500 mg four times daily; Vanco+BL=Vancomycin+bowel lavage

|  | P-score (random) |
| --- | --- |
| FMT | 0·9832 |
| Fidax | 0·6787 |
| Nita | 0·6534 |
| Teico | 0·6101 |
| RBT | 0·6020 |
| Metro | 0·5259 |
| FA | 0·4351 |
| Suro | 0·4123 |
| Vanco125 | 0·3996 |
| Vanco500 | 0·3847 |
| Vanco+BL | 0·2565 |
| Tolev | 0·0556 |

# ***Table S24:* P-score table (SUCRA) of the treatments in case of cure rate in the recurrent cases, vancomycin dosages subgroup analysis**

The possible therapies are ranked based on the P-score. P-score shows the average confidence with which we can say that one treatment is better than another. P-score can range from 0 to 1.

FA=Fusidic acid; Fidax=Fidaxomicin; FMT=Fecal microbiota transplantation; Metro=Metronidazole; Nita=Nitazoxanide; RBT=Rectal bacteriotherapy; Suro=Surotomycin; Teico=Teicoplanin; Tolev=Tolevamer; Vanco125=Vancomycin 125 mg four times daily; Vanco500=Vancomycin 500 mg four times daily; Vanco+BL=Vancomycin+bowel lavage

| FMT | 15·40  [2·09; 113·37] | ·· | ·· | 4·98  [0·94; 26·42] | ·· | ·· | ·· | 14·69  [4·82; 44·75] | 33·75  [2·53; 450·57] | 50·00  [3·55; 703·84] | ·· |
| --- | --- | --- | --- | --- | --- | --- | --- | --- | --- | --- | --- |
| 7·95  [2·36; 26·74] | Fidax | ·· | ·· | ·· | ·· | ·· | ·· | 2·30  [1·01; 5·28] | ·· | ·· | ·· |
| 6·50  [0·40; 106·85] | 0·82  [0·05; 12·47] | Nita | ·· | ·· | ·· | ·· | ·· | 2·55  [0·19; 34·41] | ·· | ·· | ·· |
| 8·26  [0·49; 139·42] | 1·04  [0·06; 18·05] | 1·27  [0·03; 56·08] | Teico | ·· | 1·86  [0·13; 27·60] | 2·00  [0·13; 29·69] | ·· | ·· | 1·86  [0·13; 27·60] | ·· | ·· |
| 9·38  [2·10; 41·91] | 1·18  [0·23; 5·95] | 1·44  [0·07; 28·21] | 1·14  [0·05; 24·24] | RBT | ·· | ·· | ·· | 1·12  [0·24; 5·19] | ·· | ·· | ·· |
| 12·30  [2·83; 53·46] | 1·55  [0·38; 6·25] | 1·89  [0·11; 32·56] | 1·49  [0·11; 20·14] | 1·31  [0·21; 8·06] | Metro | 1·07  [0·11; 10·85] | ·· | 1·52  [0·45; 5·14] | 1·00  [0·10; 10·08] | ·· | 6·74  [2·06; 22·08] |
| 16·52  [1·41; 193·92] | 2·08  [0·17; 25·21] | 2·54  [0·07; 86·25] | 2·00  [0·13; 29·69] | 1·76  [0·11; 26·97] | 1·34  [0·15; 12·18] | FA | ·· | ·· | 0·93  [0·09; 9·40] | ·· | ·· |
| 16·85  [3·57; 79·55] | 2·12  [0·52; 8·70] | 2·59  [0·15; 44·74] | 2·04  [0·10; 40·41] | 1·80  [0·28; 11·39] | 1·37  [0·27; 7·02] | 1·02  [0·07; 14·37] | Suro | 0·98  [0·31; 3·14] | ·· | ·· | ·· |
| 16·58  [5·91; 46·52] | 2·09  [0·93; 4·67] | 2·55  [0·19; 34·41] | 2·01  [0·13; 31·46] | 1·77  [0·42; 7·44] | 1·35  [0·43; 4·26] | 1·00  [0·09; 10·82] | 0·98  [0·31; 3·14] | Vanco125 | ·· | ·· | 4·42  [1·37; 14·25] |
| 19·23  [2·85; 129·76] | 2·42  [0·31; 18·69] | 2·96  [0·12; 74·66] | 2·33  [0·17; 31·49] | 2·05  [0·21; 20·36] | 1·56  [0·25; 9·88] | 1·16  [0·13; 10·56] | 1·14  [0·12; 10·67] | 1·16  [0·17; 7·84] | Vanco500 | 1·48  [0·19; 11·74] | ·· |
| 33·70  [3·24; 350·19] | 4·24  [0·34; 52·11] | 5·18  [0·15; 179·70] | 4·08  [0·17; 97·36] | 3·59  [0·24; 53·44] | 2·74  [0·24; 31·41] | 2·04  [0·12; 35·39] | 2·00  [0·14; 28·98] | 2·03  [0·18; 22·60] | 1·75  [0·24; 12·98] | Vanco+BL | ·· |
| 77·88  [17·18; 353·17] | 9·80  [2·41; 39·90] | 11·98  [0·69; 206·44] | 9·43  [0·57; 156·04] | 8·30  [1·33; 51·81] | 6·33  [1·97; 20·39] | 4·71  [0·41; 54·10] | 4·62  [0·90; 23·75] | 4·70  [1·48; 14·91] | 4·05  [0·52; 31·55] | 2·31  [0·18; 29·95] | Tolev |

# ***Table S25:* League table of possible treatments for cure rates in recurrent cases, vancomycin dosages subgroup analysis**

Possible treatments are arranged in descending order of the P score in the blue bars. P scores are given in brackets after treatment names. Direct comparisons are shown above these blue bars, whereas below, direct and indirect (estimated) ones are pooled together as network estimates. Odds ratios are given in the cells. We compare one treatment on the left side with another one on the right side, indicating a greater odds ratio of recovery. The 95% confidence interval is shown in brackets. Significant results are marked in green.

FA=Fusidic acid; Fidax=Fidaxomicin; FMT=Fecal microbiota transplantation; Metro=Metronidazole; Nita=Nitazoxanide; RBT=Rectal bacteriotherapy; Suro=Surotomycin; Teico=Teicoplanin; Tolev=Tolevamer; Vanco125=Vancomycin 125 mg four times daily; Vanco500=Vancomycin 500 mg four times daily; Vanco+BL=Vancomycin+bowel lavage


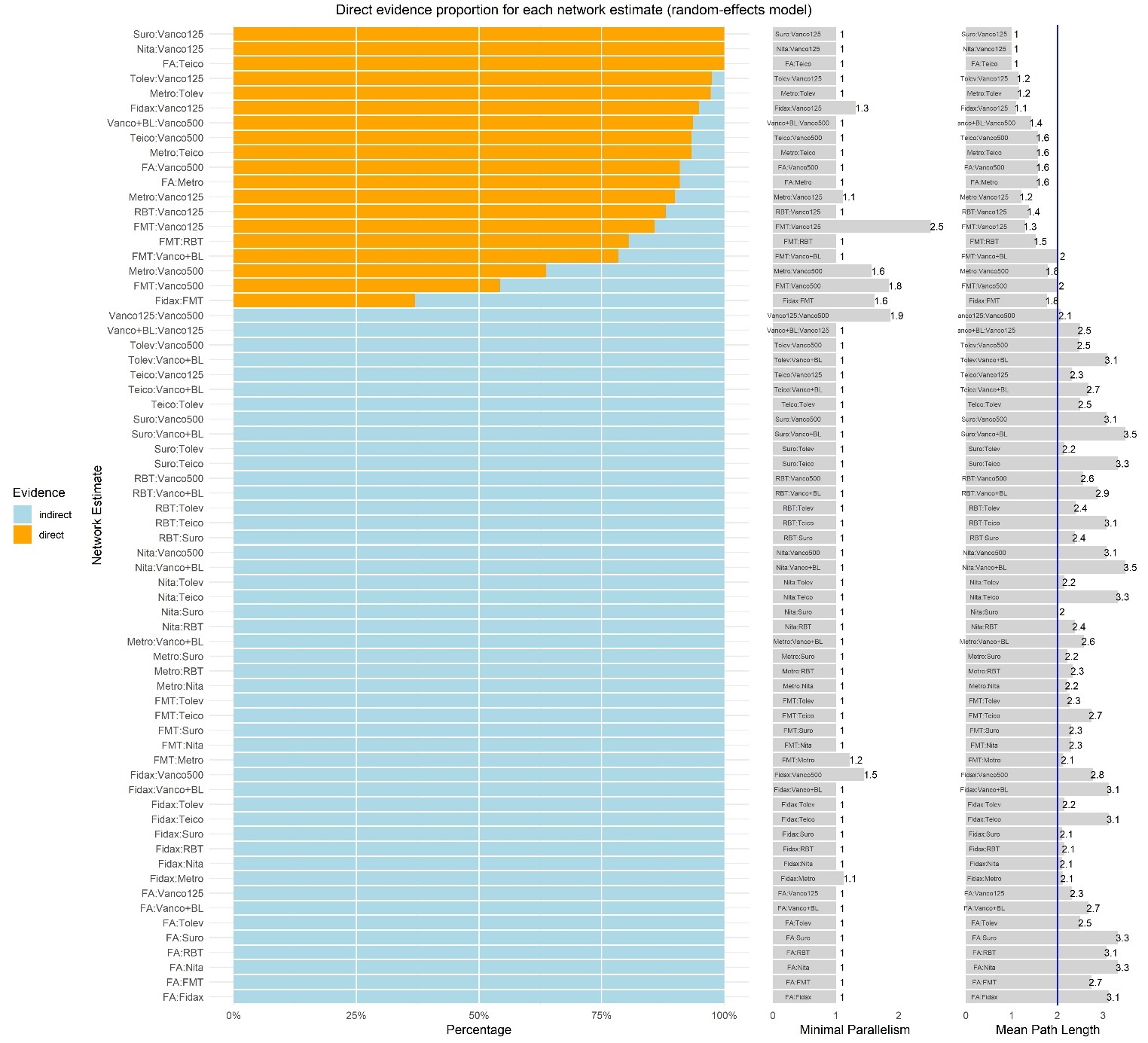


# ***Figure S33:* Evidence plot of the treatments in case of cure rate in the recurrent cases, vancomycin dosages subgroup analysis**

It shows what percentage of the result comes from the direct comparison and how much comes from the indirect/estimated data. The direct ones are marked orange, while the estimated ones are marked blue. In the Mean Path Length diagram, if the given comparison is greater than 2, then these network estimations should be interpreted carefully. Higher values of parallelism indicate greater robustness of the estimate.

FA=Fusidic acid; Fidax=Fidaxomicin; FMT=Fecal microbiota transplantation; Metro=Metronidazole; Nita=Nitazoxanide; RBT=Rectal bacteriotherapy; Suro=Surotomycin; Teico=Teicoplanin; Tolev=Tolevamer; Vanco125=Vancomycin 125 mg four times daily; Vanco500=Vancomycin 500 mg four times daily; Vanco+BL=Vancomycin+bowel lavage


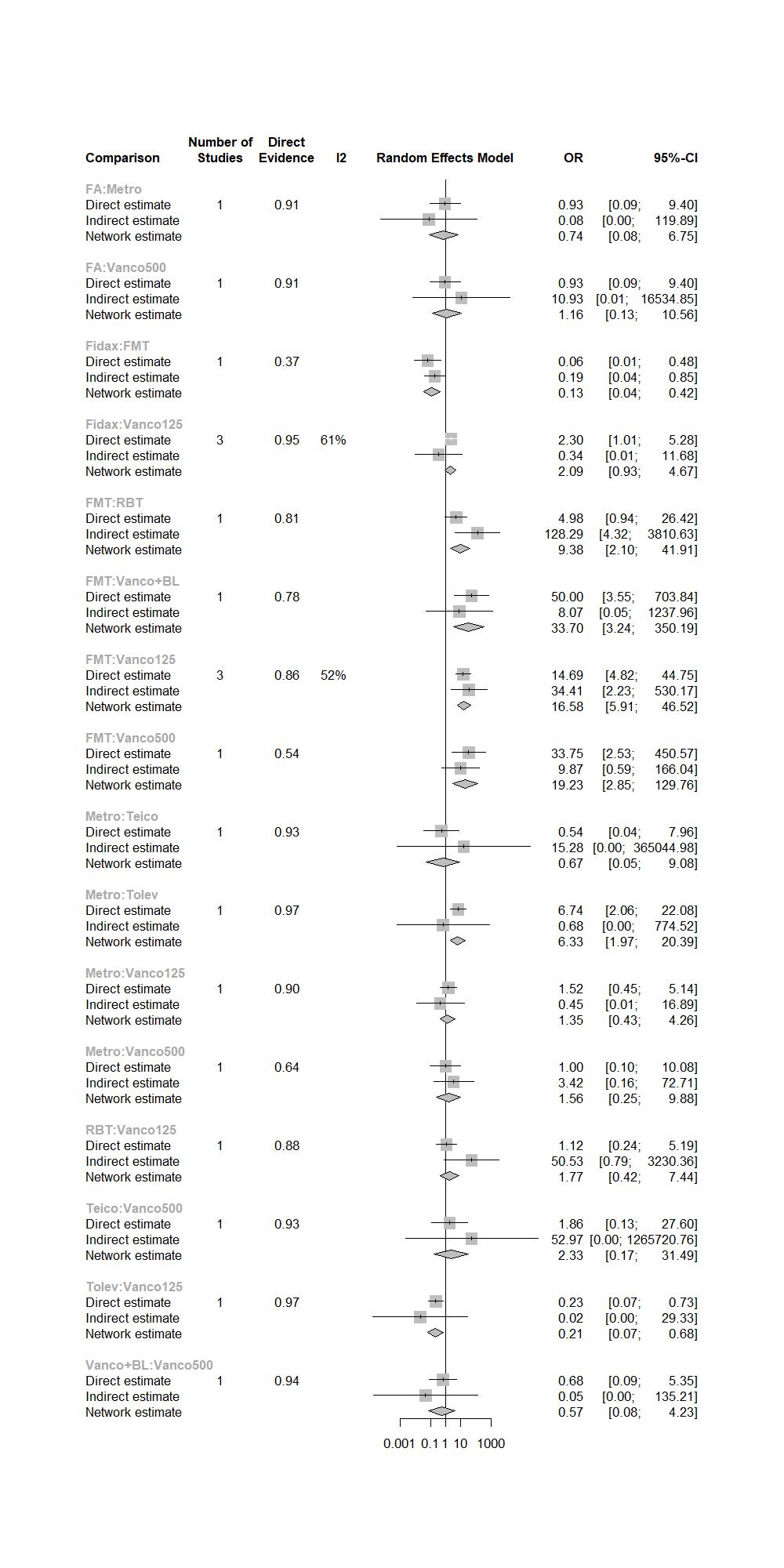


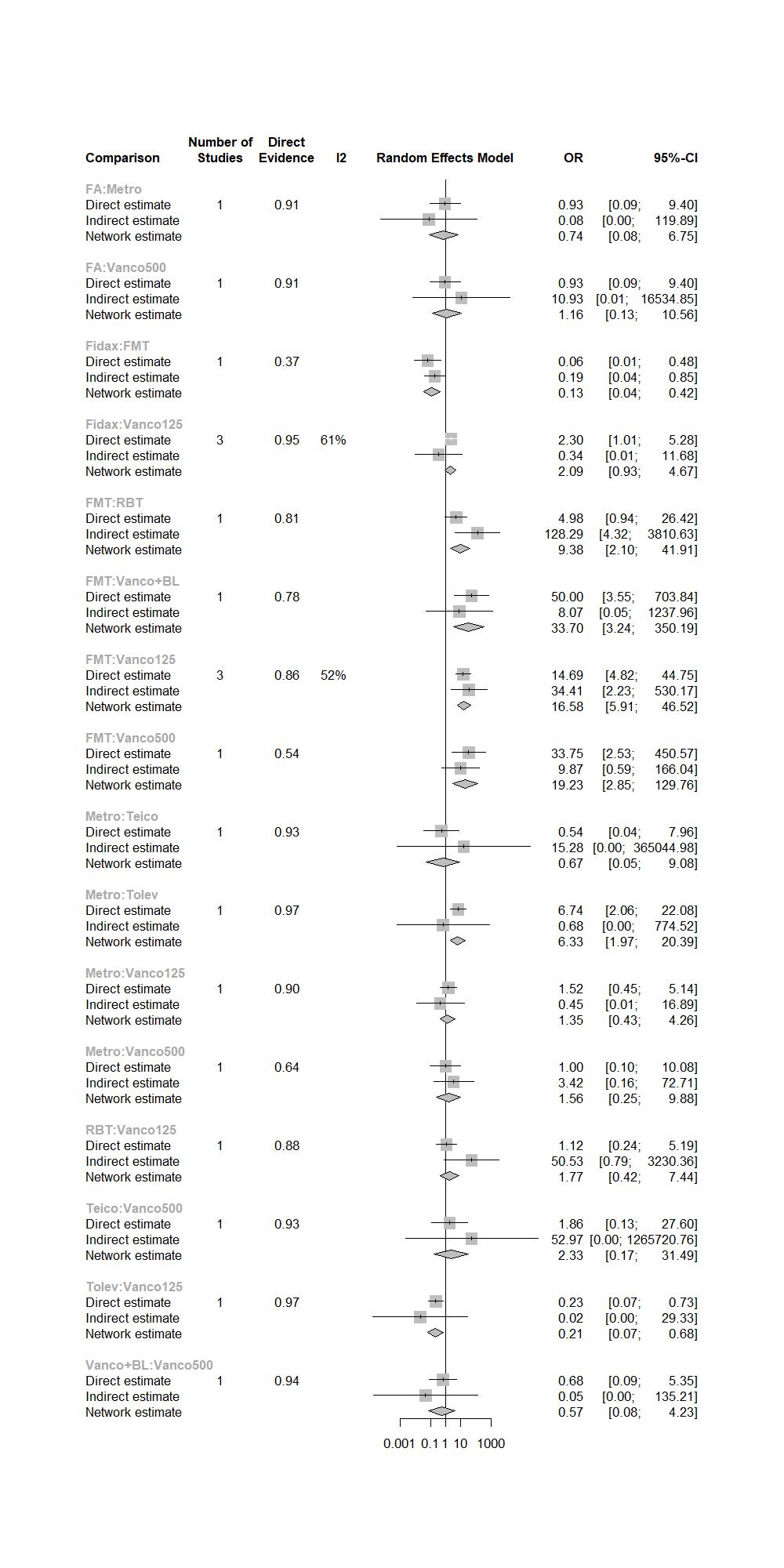


# ***Figure S34:* Forest plot for results of consistency analysis for the treatments in case of cure rate in the recurrent cases, vancomycin dosages subgroup analysis**

The Forest plot shows the result of the consistency analysis. The direct and indirect comparisons do not contradict each other, so the network can be considered consistent.

FA=Fusidic acid; Fidax=Fidaxomicin; FMT=Fecal microbiota transplantation; Metro=Metronidazole; RBT=Rectal bacteriotherapy; Teico=Teicoplanin; Tolev=Tolevamer; Vanco125=Vancomycin 125 mg four times daily; Vanco500=Vancomycin 500 mg four times daily; Vanco+BL=Vancomycin+bowel lavage


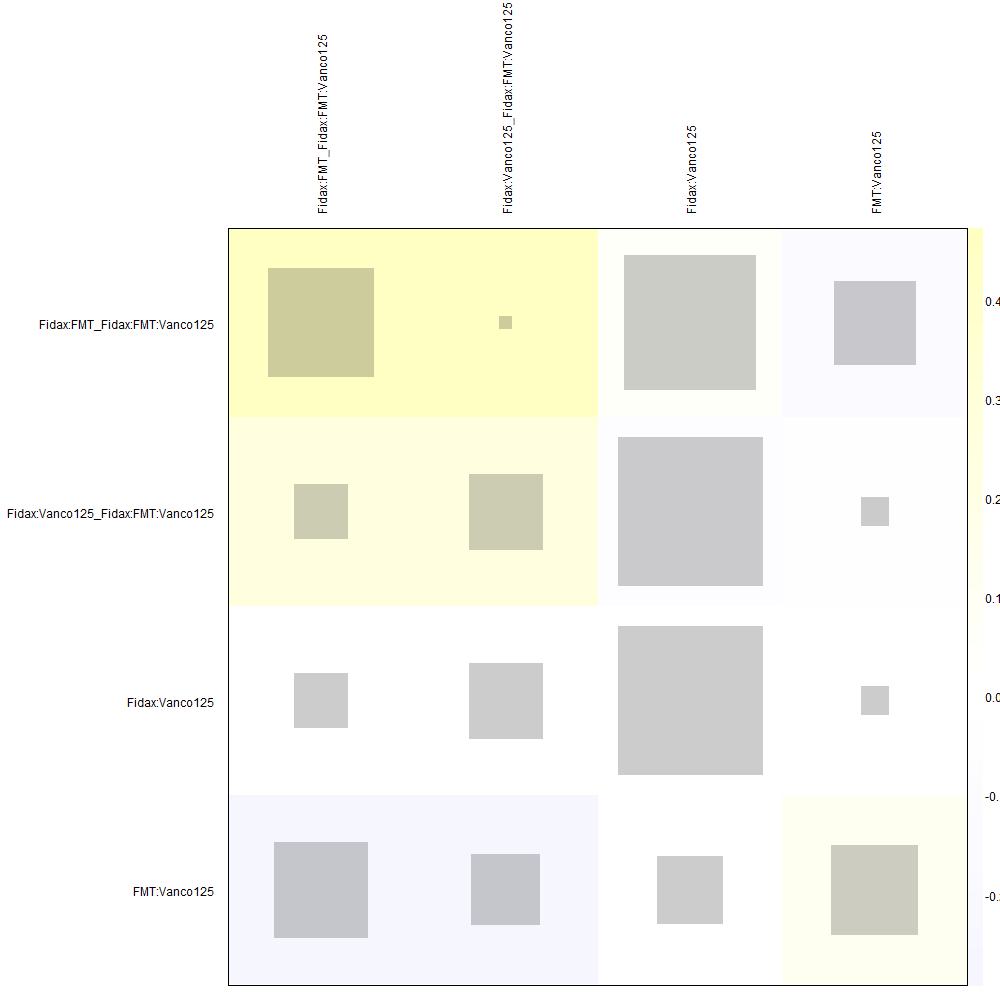


# ***Figure S35:* Net heat plot of the treatments in case of cure rate in the recurrent cases, vancomycin dosages subgroup analysis**

It assesses if there is a consistency problem or not. It shows the difference between direct estimation and the network estimation. The right column shows that the redder the difference, the more inconsistent the result. The area of a gray square represents the contribution of the direct estimate from a design in the column to the network estimate in the row.

FA=Fusidic acid; Fidax=Fidaxomicin; FMT=Fecal microbiota transplantation; Metro=Metronidazole; Vanco125=Vancomycin 125 mg four times daily


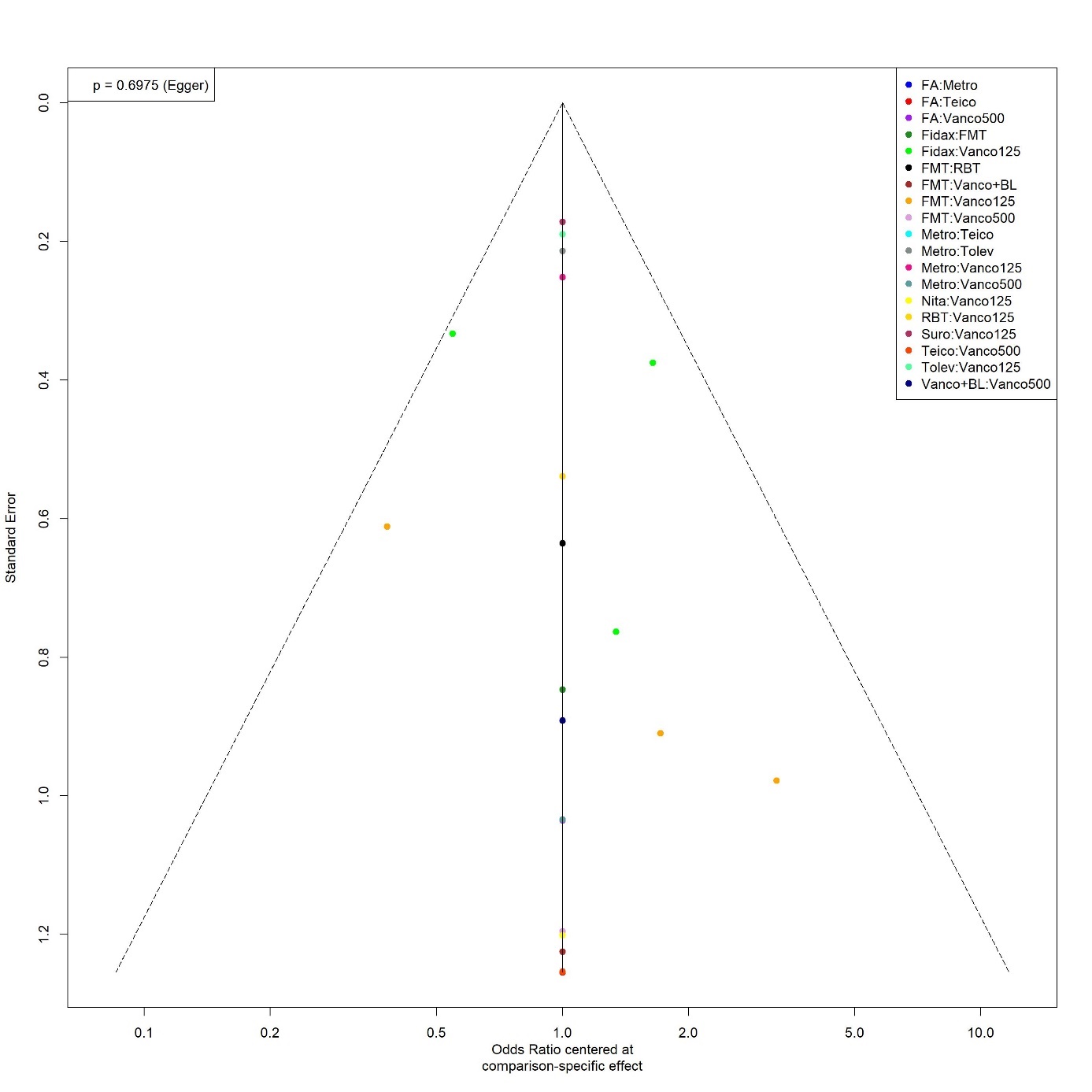


# ***Figure S36:* Funnel plot of the treatments in case of cure rate in the recurrent cases, vancomycin dosages subgroup analysis**

It shows the extent to which the analysis is affected by the small study effect. If the points are under the tent, symmetrically distributed, and the Egger number is bigger than 0.05, in that case the small study effect does not affect our analysis.

FA=Fusidic acid; Fidax=Fidaxomicin; FMT=Fecal microbiota transplantation; Metro=Metronidazole; Nita=Nitazoxanide; RBT=Rectal bacteriotherapy; Suro=Surotomycin; Teico=Teicoplanin; Tolev=Tolevamer; Vanco125=Vancomycin 125 mg four times daily; Vanco500=Vancomycin 500 mg four times daily; Vanco+BL=Vancomycin+bowel lavage

| **Summary of network table** | | | | |
| --- | --- | --- | --- | --- |
| **Characteristic** | | | | **Value** |
| Number of Interventions | | | | 13 |
| Number of Studies | | | | 17 |
| Total Number of Patients in Network | | | | 3173 |
| Total Possible Pairwise Comparisons | | | | 78 |
| Total Number of Pairwise Comparisons with Direct Data | | | | 13 |
| Number of Two-arm Studies | | | | 16 |
| Number of Multi-Arms Studies | | | | 1 |
| Total Number of Events in Network | | | | 2501 |
| Number of Studies With No Zero Events | | | | 17 |
| Number of Studies With At Least One Zero Event | | | | 0 |
| Number of Studies With All Zero Events | | | | 0 |
|  |  |  |  | |
| **Treatment** | **Studies (n)** | **Events (n)** | **Patients (n)** | |
| Bacit | 2 | 23 | 32 | |
| FA | 1 | 49 | 59 | |
| Fidax | 5 | 515 | 567 | |
| FMT | 1 | 19 | 21 | |
| Metro | 6 | 198 | 240 | |
| Metro+rif | 1 | 12 | 19 | |
| Nita | 1 | 68 | 76 | |
| Plac | 2 | 9 | 31 | |
| Ridin | 1 | 270 | 370 | |
| Suro | 1 | 180 | 285 | |
| Teico | 1 | 25 | 26 | |
| Vanco125 | 9 | 1040 | 1340 | |
| Vanco500 | 4 | 93 | 107 | |

# ***Table S26:* Network summary table of the treatments for cure rates in non-recurrent cases, vancomycin dosages subgroup analysis**

Key data of analysis and treatments.

Bacit=Bacitracin; FA=Fusidic acid; Fidax=Fidaxomicin; FMT=Fecal microbiota transplantation; Metro=Metronidazole; Metro+rif=Metronidazole+Rifampin; Nita=Nitazoxanide; Plac=Placebo; Ridin=Ridinilazole; Suro=Surotomycin; Teico=Teicoplanin; Vanco125=Vancomycin 125 mg four times daily; Vanco500=Vancomycin 500 mg four times daily


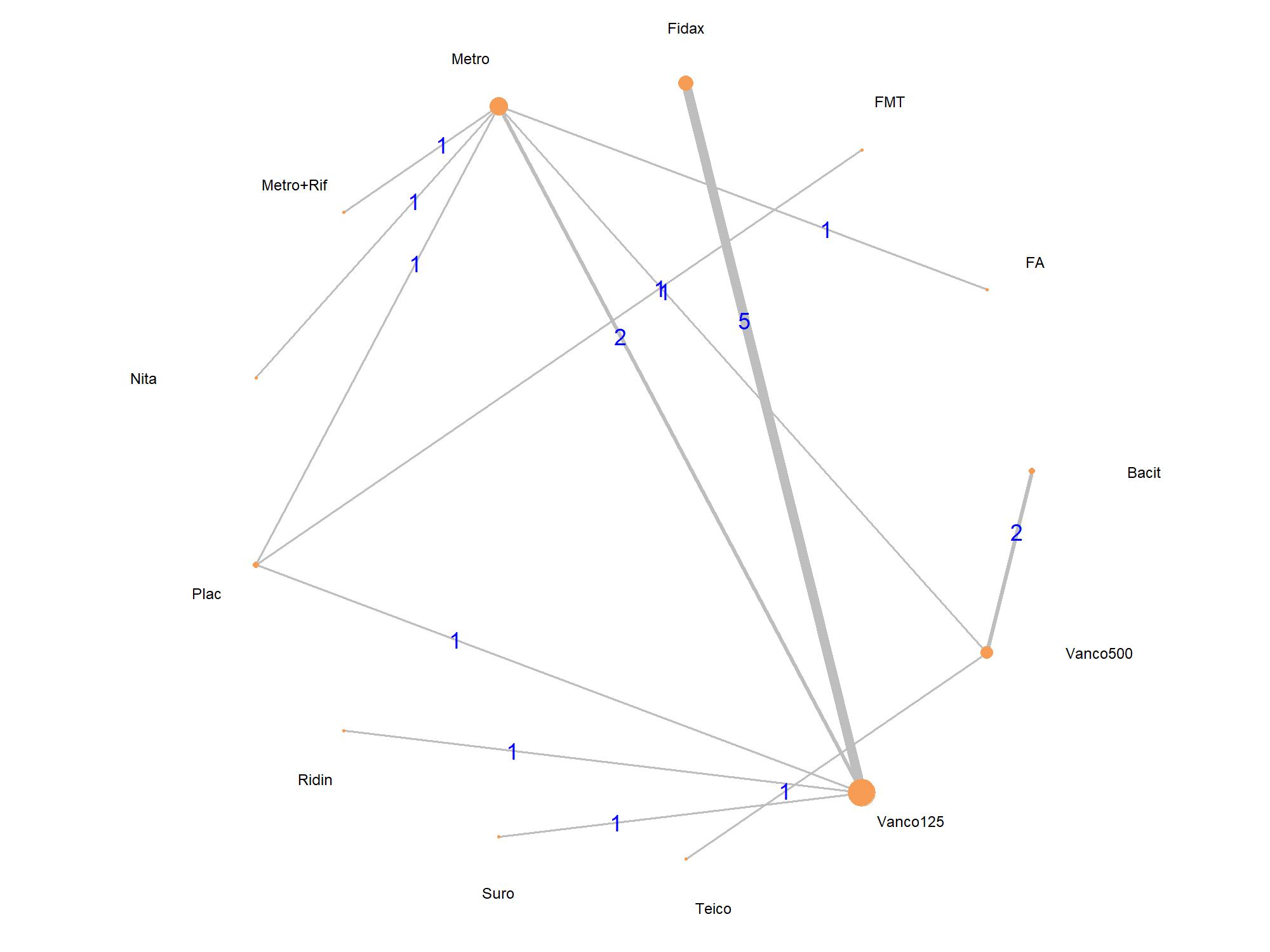


# ***Figure S37:* Network plot of possible treatments for cure rates in non-recurrent cases, vancomycin dosages subgroup analysis**

Every knot represents a different therapy for CDI. The larger a knot, the more studies included that treatment. Every edge compares different therapies. The width and the number above indicate how many studies investigated this comparison.

Bacit=Bacitracin; FA=Fusidic acid; Fidax=Fidaxomicin; FMT=Fecal microbiota transplantation; Metro=Metronidazole; Metro+rif=Metronidazole+Rifampin; Nita=Nitazoxanide; Plac=Placebo; Ridin=Ridinilazole; Suro=Surotomycin; Teico=Teicoplanin; Vanco125=Vancomycin 125 mg four times daily; Vanco500=Vancomycin 500 mg four times daily

|  | P-score (random) |
| --- | --- |
| Fidax | 0·8945 |
| Suro | 0·8380 |
| Ridin | 0·8206 |
| FMT | 0·7693 |
| Vanco125 | 0·7663 |
| Nita | 0·5107 |
| Metro | 0·3756 |
| Metro+Rif | 0·3507 |
| Vanco500 | 0·3466 |
| Bacit | 0·2370 |
| Teico | 0·2252 |
| Plac | 0·2083 |
| FA | 0·1573 |

# ***Table S27:* P-score table (SUCRA) of the treatments in case of cure rate in the non-recurrent cases, vancomycin dosages subgroup analysis**

The possible therapies are ranked based on the P-score. P-score shows the average confidence with which we can say that one treatment is better than another. P-score can range from 0 to 1.

Bacit=Bacitracin; FA=Fusidic acid; Fidax=Fidaxomicin; FMT=Fecal microbiota transplantation; Metro=Metronidazole; Metro+rif=Metronidazole+Rifampin; Nita=Nitazoxanide; Plac=Placebo; Ridin=Ridinilazole; Suro=Surotomycin; Teico=Teicoplanin; Vanco125=Vancomycin 125 mg four times daily; Vanco500=Vancomycin 500 mg four times daily

| Fidax | ·· | ·· | ·· | 1·37  [0·82; 2·27] | ·· | ·· | ·· | ·· | ·· | ·· | ·· | ·· |
| --- | --- | --- | --- | --- | --- | --- | --- | --- | --- | --- | --- | --- |
| 1·16  [0·50; 2·66] | Suro | ·· | ·· | 1·18  [0·61; 2·28] | ·· | ·· | ·· | ·· | ·· | ·· | ·· | ·· |
| 1·22  [0·53; 2·78] | 1·05  [0·42; 2·66] | Ridin | ·· | 1·12  [0·58; 2·15] | ·· | ·· | ·· | ·· | ·· | ·· | ·· | ·· |
| 1·49  [0·08; 26·87] | 1·29  [0·07; 23·90] | 1·22  [0·07; 22·68] | FMT | ·· | ·· | ·· | ·· | ·· | ·· | ·· | 19·00  [3·12; 115·85] | ·· |
| 1·37  [0·82; 2·27] | 1·18  [0·61; 2·28] | 1·12  [0·58; 2·15] | 0·92  [0·05; 15·76] | Vanco125 | ·· | 9·45  [2·40; 37·19] | ·· | ·· | ·· | ·· | 36·00  [2·56; 506·57] | ·· |
| 7·09  [1·02; 49·43] | 6·12  [0·84; 44·63] | 5·82  [0·80; 42·30] | 4·75  [0·24; 95·75] | 5·19  [0·80; 33·82] | Nita | 1·82  [0·51; 6·55] | ·· | ·· | ·· | ·· | ·· | ·· |
| 12·91  [2·99; 55·67] | 11·14  [2·44; 50·98] | 10·59  [2·32; 48·29] | 8·65  [0·57; 131·01] | 9·45  [2·40; 37·19] | 1·82  [0·51; 6·55] | Metro | 1·08  [0·26; 4·51] | 1·15  [0·30; 4·45] | ·· | ·· | 1·71  [0·20; 14·48] | 2·60  [0·68; 10·03] |
| 13·99  [1·81; 107·81] | 12·07  [1·50; 97·12] | 11·47  [1·43; 92·08] | 9·37  [0·44; 201·77] | 10·24  [1·42; 74·01] | 1·97  [0·29; 13·41] | 1·08  [0·26; 4·51] | Metro+Rif | ·· | ·· | ·· | ·· | ·· |
| 14·86  [2·03; 108·83] | 12·83  [1·68; 98·15] | 12·19  [1·60; 93·04] | 9·96  [0·48; 207·24] | 10·88  [1·59; 74·58] | 2·10  [0·33; 13·49] | 1·15  [0·30; 4·45] | 1·06  [0·15; 7·59] | Vanco500 | 1·57  [0·44; 5·69] | 2·41  [0·09; 65·53] | ·· | ·· |
| 23·38  [2·19; 249·99] | 20·18  [1·82; 223·90] | 19·18  [1·73; 212·35] | 15·67  [0·58; 423·14] | 17·11  [1·69; 173·17] | 3·30  [0·34; 31·67] | 1·81  [0·28; 11·69] | 1·67  [0·16; 17·50] | 1·57  [0·44; 5·69] | Bacit | ·· | ·· | ·· |
| 35·85  [0·76;1694·48] | 30·94  [0·64;1496·33] | 29·41  [0·61;1420·28] | 24·02  [0·27;2130·69] | 26·24  [0·57;1199·29] | 5·06  [0·11; 223·94] | 2·78  [0·08; 98·44] | 2·56  [0·05; 119·58] | 2·41  [0·09; 65·53] | 1·53  [0·04; 53·01] | Teico | ·· | ·· |
| 28·35  [2·97; 270·46] | 24·47  [2·47; 242·68] | 23·26  [2·35; 230·14] | 19·00  [3·12; 115·85] | 20·75  [2·31; 186·82] | 4·00  [0·36; 44·02] | 2·20  [0·29; 16·70] | 2·03  [0·17; 24·21] | 1·91  [0·17; 21·84] | 1·21  [0·08; 19·08] | 0·79  [0·01; 47·95] | Plac | ·· |
| 33·60  [4·60; 245·51] | 29·00  [3·80; 221·42] | 27·56  [3·62; 209·91] | 22·52  [1·08; 467·85] | 24·59  [3·59; 168·23] | 4·74  [0·74; 30·42] | 2·60  [0·68; 10·03] | 2·40  [0·34; 17·11] | 2·26  [0·33; 15·27] | 1·44  [0·14; 14·36] | 0·94  [0·02; 42·53] | 1·19  [0·10; 13·55] | FA |

# ***Table S28:* League table of possible treatments for cure rates in non-recurrent cases, vancomycin dosages subgroup analysis**

Possible treatments are arranged in descending order of the P score in the blue bars. P scores are given in brackets after treatment names. Direct comparisons are shown above these blue bars, whereas below, the direct and indirect (estimated) ones are pooled together as network estimates. Odds ratios are given in the cells. We compare one treatment on the left side with another one on the right side, indicating a greater odds ratio of recovery. The 95% confidence interval is shown in brackets. Significant results are marked in green.

Bacit=Bacitracin; FA=Fusidic acid; Fidax=Fidaxomicin; FMT=Fecal microbiota transplantation; Metro=Metronidazole; Metro+rif=Metronidazole+Rifampin; Nita=Nitazoxanide; Plac=Placebo; Ridin=Ridinilazole; Suro=Surotomycin; Teico=Teicoplanin; Vanco125=Vancomycin 125 mg four times daily; Vanco500=Vancomycin 500 mg four times daily


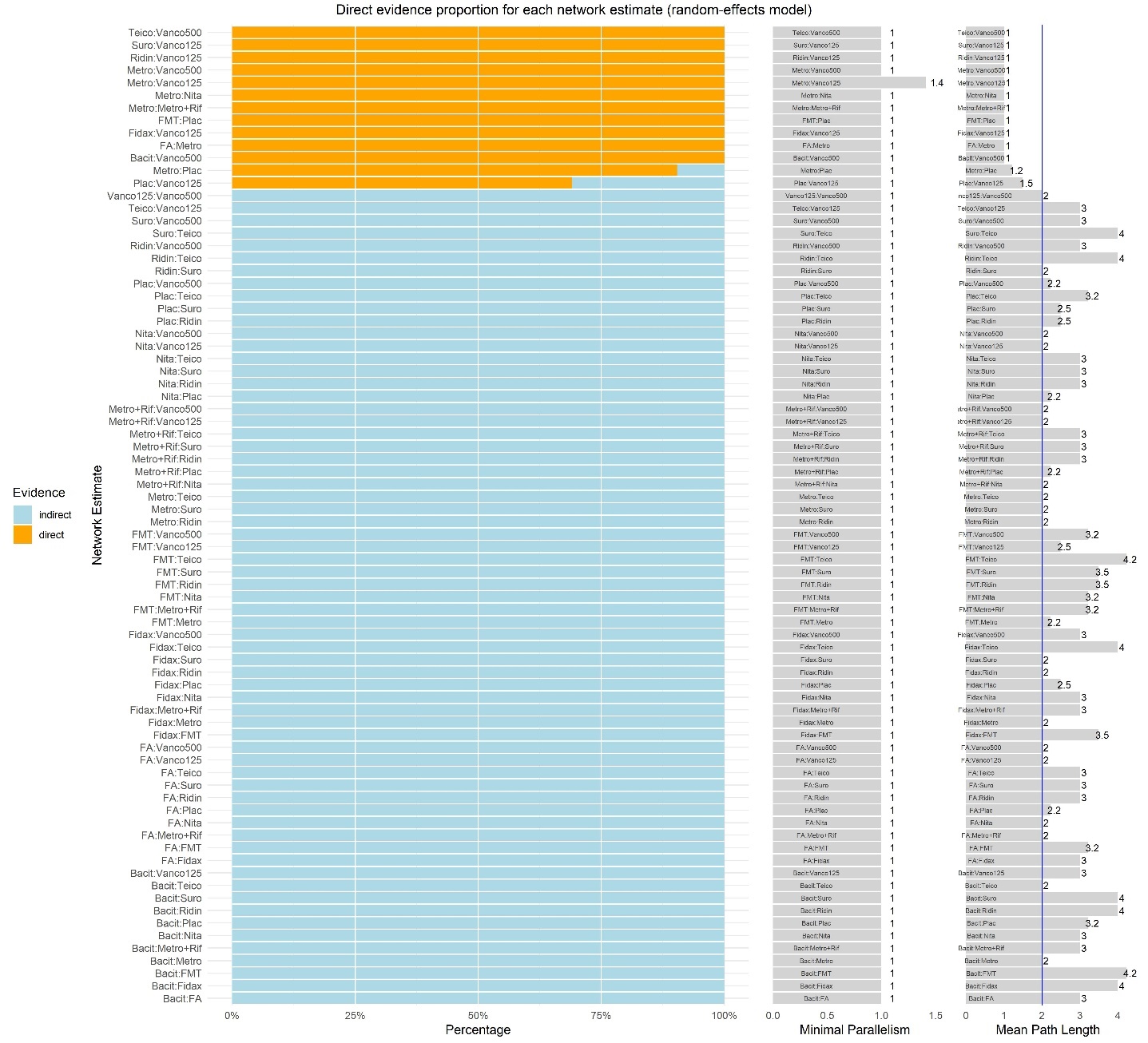


# ***Figure S38:* Evidence plot of the treatments in case of cure rate in the non-recurrent cases, vancomycin dosages subgroup analysis**

It shows what percentage of the result comes from the direct comparison and how much comes from the indirect/estimated data. The direct ones are marked orange, while the estimated ones are marked blue. In the Mean Path Length diagram, if the given comparison is greater than 2, then these network estimations should be interpreted carefully. Higher values of parallelism indicate greater robustness of the estimate.

Bacit=Bacitracin; FA=Fusidic acid; Fidax=Fidaxomicin; FMT=Fecal microbiota transplantation; Metro=Metronidazole; Metro+rif=Metronidazole+Rifampin; Nita=Nitazoxanide; Plac=Placebo; Ridin=Ridinilazole; Suro=Surotomycin; Teico=Teicoplanin; Vanco125=Vancomycin 125 mg four times daily; Vanco500=Vancomycin 500 mg four times daily


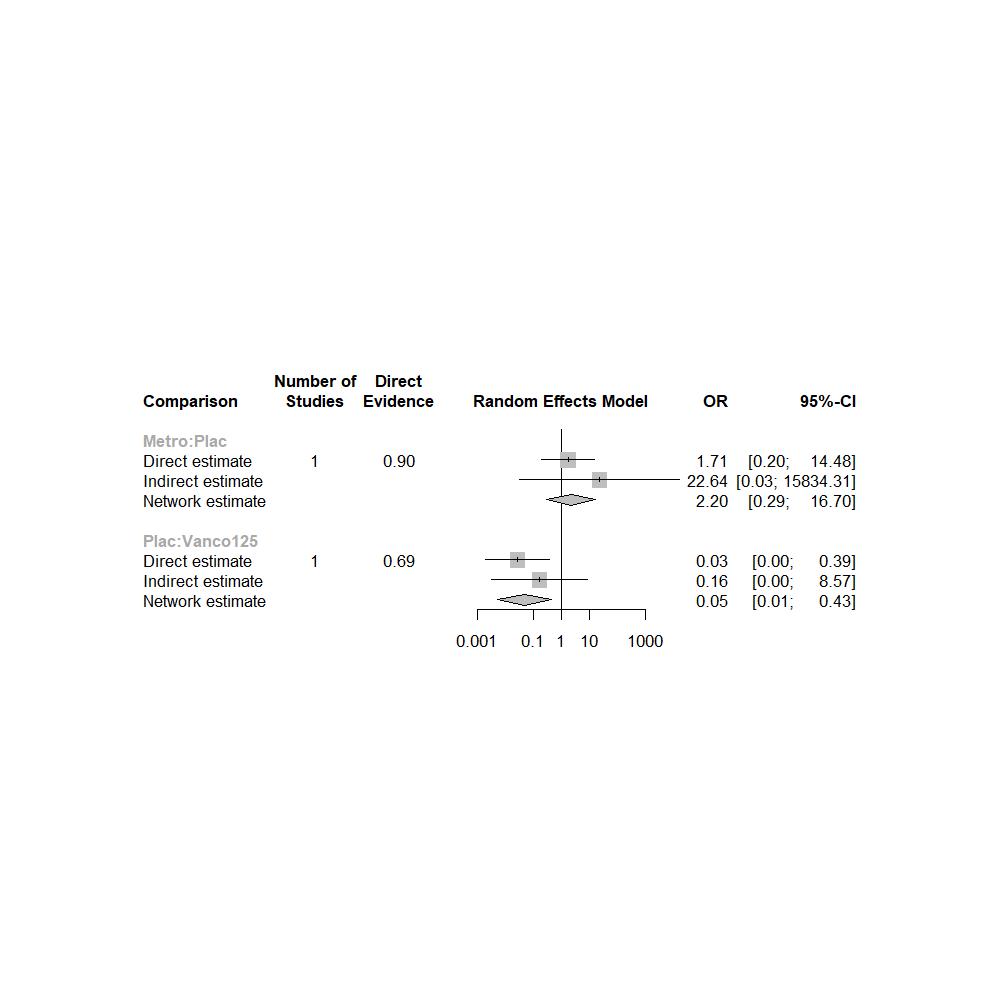


# ***Figure S39:* Forest plot for results of consistency analysis for the treatments in case of cure rate in the non-recurrent cases, vancomycin dosages subgroup analysis**

The Forest plot shows the result of the consistency analysis. The direct and indirect comparisons do not contradict each other, so the network can be considered consistent.

Metro=Metronidazole; Plac=Placebo; Vanco125=Vancomycin 125 mg four times daily


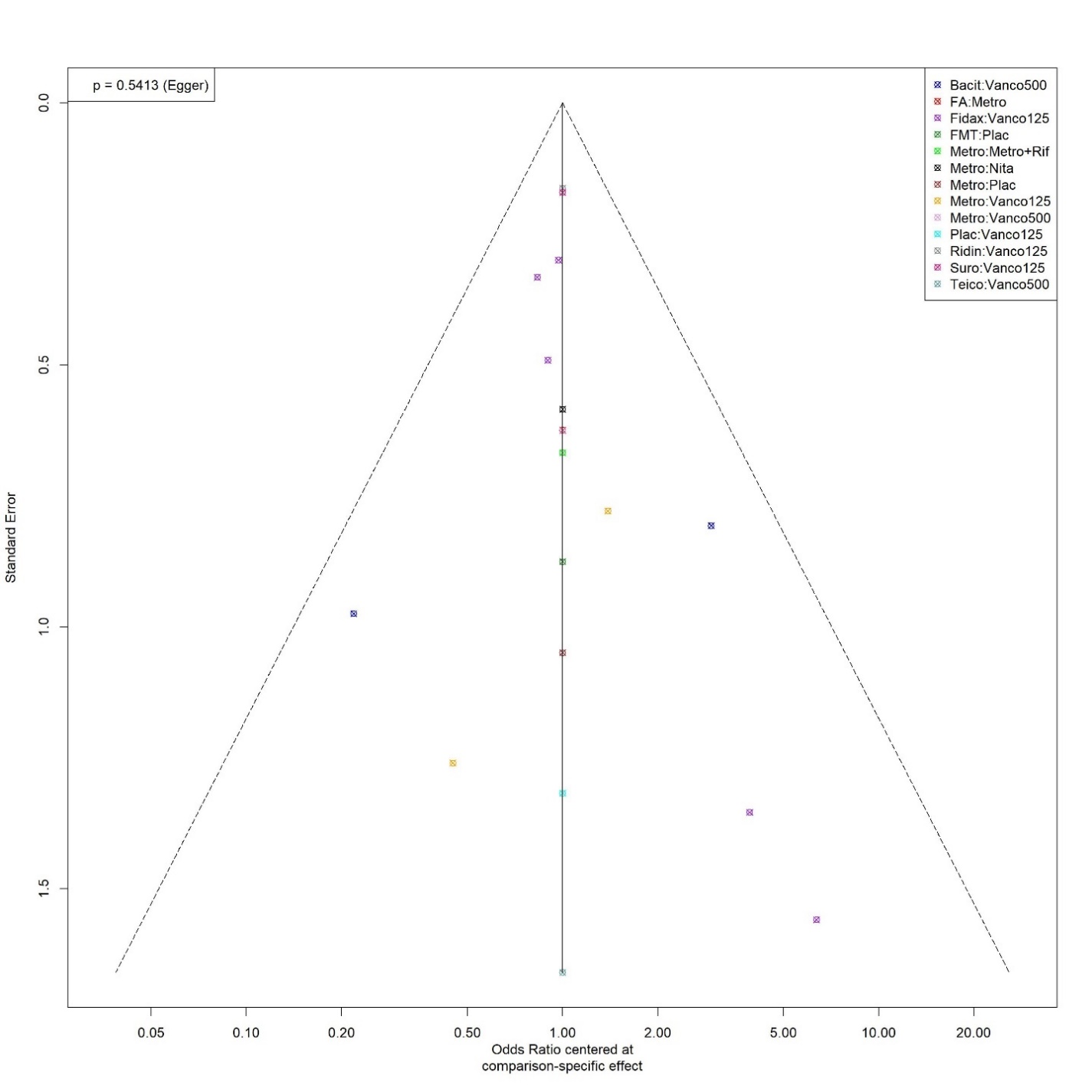


# ***Figure S40:* Funnel plot of the treatments in case of cure rate in the non-recurrent cases, vancomycin dosages subgroup analysis**

It shows the extent to which the analysis is affected by the small study effect. If the points are under the tent, symmetrically distributed, and the Egger number is bigger than 0.05, in that case the small study effect does not affect our analysis.

Bacit=Bacitracin; FA=Fusidic acid; Fidax=Fidaxomicin; FMT=Fecal microbiota transplantation; Metro=Metronidazole; Metro+rif=Metronidazole+Rifampin; Nita=Nitazoxanide; Plac=Placebo; Ridin=Ridinilazole; Suro=Surotomycin; Teico=Teicoplanin; Vanco125=Vancomycin 125 mg four times daily; Vanco250=Vancomycin 250 mg four times daily; Vanco500=Vancomycin 500 mg four times daily

# **Supplementary Results S2 – Subgroup analysis of recurrence result by dose of vancomycin treatments**

Similar to the cure rate analysis, a dose-based subgroup analysis of vancomycin was conducted for CDI recurrence. In this case, only two doses (125 mg and 500 mg) were used in the trials. The order of treatments remained largely unchanged compared to the original analysis, with only minor changes. The 125 mg vancomycin dose appeared to be more effective than the 500 mg dose in preventing CDI recurrence, although the result was not statistically significant.

The detailed results of the analysis are provided in Supplementary Figures S41-S45 and Supplementary Tables S29-S31.

| **Summary of network table** | | | | |
| --- | --- | --- | --- | --- |
|  | | | |  |
| Number of Interventions | | | | 16 |
| Number of Studies | | | | 22 |
| Total Number of Patients in Network | | | | 4592 |
| Total Possible Pairwise Comparisons | | | | 120 |
| Total Number of Pairwise Comparisons with Direct Data | | | | 21 |
| Number of Two-arm Studies | | | | 19 |
| Number of Multi-Arms Studies | | | | 3 |
| Total Number of Events in Network | | | | 815 |
| Number of Studies With No Zero Events | | | | 21 |
| Number of Studies With At Least One Zero Event | | | | 1 |
| Number of Studies With All Zero Events | | | | 0 |
|  | 13 |  |  | |
| **Treatment** | **Studies (n)** | **Events (n)** | **Patients (n)** | |
| Bacit | 1 | 5 | 12 | |
| FA | 2 | 22 | 76 | |
| FMT | 4 | 11 | 76 | |
| FMT-Bez | 1 | 4 | 30 | |
| FMT-L | 1 | 1 | 8 | |
| Fidax | 7 | 85 | 722 | |
| Metro | 5 | 75 | 351 | |
| Metro+rif | 1 | 5 | 12 | |
| Nita | 1 | 1 | 17 | |
| Ridin | 1 | 30 | 370 | |
| Suro | 2 | 110 | 584 | |
| Teico | 2 | 4 | 54 | |
| Tolev | 1 | 10 | 202 | |
| Vanco125 | 14 | 425 | 1986 | |
| Vanco500 | 4 | 20 | 79 | |
| Vanco+BL | 1 | 7 | 13 | |

# ***Table S29:* Network summary table of the treatments in recurrence, vancomycin dosages subgroup analysis**

Key data of analysis and treatments.

Bacit=Bacitracin; FA=Fusidic acid; Fidax=Fidaxomicin; FMT=Fecal microbiota transplantation; FMT-Bez=Fecal microbiota transplantation+Bezlotoxumab; FMT-L=Fecal microbiota transplantation+*Lactobacillus*; Metro=Metronidazole; Metro+rif=Metronidazole+Rifampin; Nita=Nitazoxanide; Ridin=Ridinilazole; Suro=Surotomycin; Teico=Teicoplanin; Tolev=Tolevamer; Vanco125=Vancomycin 125 mg four times daily; Vanco500=Vancomycin 500 mg four times daily; Vanco+BL=Vancomycin+bowel lavage


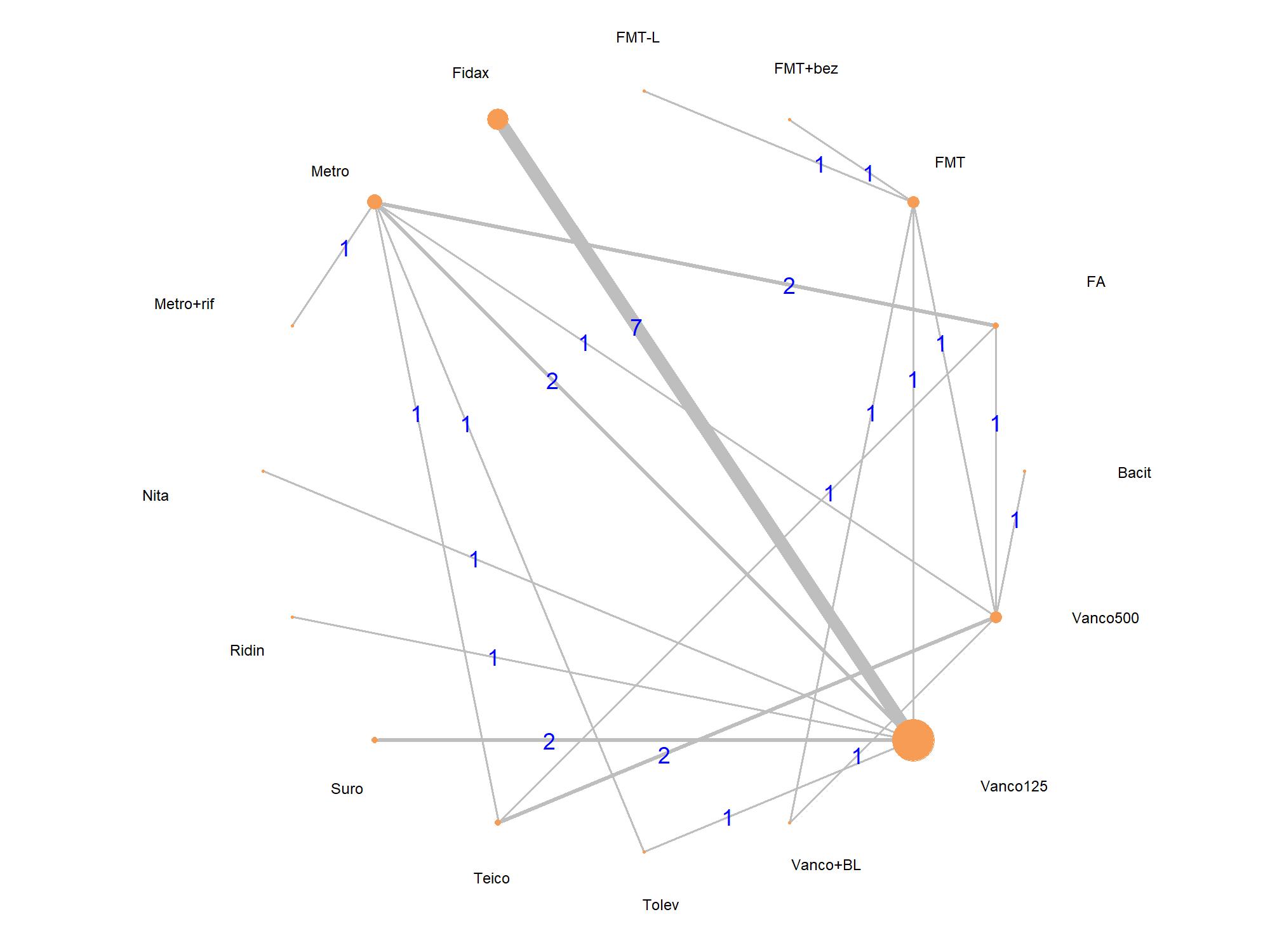


# ***Figure S41:* Network plot of possible treatments in recurrence, vancomycin dosages subgroup analysis**

Every knot represents a different therapy for CDI. The larger a knot, the more studies included that treatment. Every edge compares different therapies. The width and the number above indicate how many studies investigated this comparison.

Bacit=Bacitracin; FA=Fusidic acid; Fidax=Fidaxomicin; FMT=Fecal microbiota transplantation; FMT-Bez=Fecal microbiota transplantation+Bezlotoxumab; FMT-L=Fecal microbiota transplantation+*Lactobacillus*; Metro=Metronidazole; Metro+rif=Metronidazole+Rifampin; Nita=Nitazoxanide; Ridin=Ridinilazole; Suro=Surotomycin; Teico=Teicoplanin; Tolev=Tolevamer; Vanco125=Vancomycin 125 mg four times daily; Vanco500=Vancomycin 500 mg four times daily; Vanco+BL=Vancomycin+bowel lavage

|  | P-score (random) |
| --- | --- |
| Tolev | 0·9569 |
| Ridin | 0·8067 |
| Fidax | 0·8083 |
| Teico | 0·7241 |
| Nita | 0·6477 |
| FMT | 0·6452 |
| Suro | 0·5815 |
| Vanco125 | 0·4912 |
| Metro | 0·4131 |
| Metro+Rif | 0·4017 |
| Vanco500 | 0·3124 |
| FA | 0·2898 |
| Vanco+BL | 0·2824 |
| FMT-L | 0·2671 |
| FMT-Bez | 0·2480 |
| Bacit | 0·1287 |

# ***Table S30:* P-score table (SUCRA) of the treatments in case of recurrence, vancomycin dosages subgroup analysis**

The possible therapies are ranked based on the P-score. P-score shows the average confidence with which we can say that one treatment is better than another. P-score can range from 0 to 1.

Bacit=Bacitracin; FA=Fusidic acid; Fidax=Fidaxomicin; FMT=Fecal microbiota transplantation; FMT-Bez=Fecal microbiota transplantation+Bezlotoxumab; FMT-L=Fecal microbiota transplantation+*Lactobacillus*; Metro=Metronidazole; Metro+rif=Metronidazole+Rifampin; Nita=Nitazoxanide; Ridin=Ridinilazole; Suro=Surotomycin; Teico=Teicoplanin; Tolev=Tolevamer; Vanco125=Vancomycin 125 mg four times daily; Vanco500=Vancomycin 500 mg four times daily; Vanco+BL=Vancomycin+bowel lavage

| Tolev | ·· | ·· | ·· | ·· | ·· | ·· | 0·18  [0·08; 0·39] | 0·19  [0·08; 0·43] | ·· | ·· | ·· | ·· | ·· | ·· | ·· |
| --- | --- | --- | --- | --- | --- | --- | --- | --- | --- | --- | --- | --- | --- | --- | --- |
| 0·48  [0·18; 1·29] | Ridin | ·· | ·· | ·· | ·· | ·· | 0·42  [0·23; 0·76] | ·· | ·· | ·· | ·· | ·· | ·· | ·· | ·· |
| 0·47  [0·20; 1·11] | 0·98  [0·50; 1·92] | Fidax | ·· | ·· | ·· | ·· | 0·43  [0·31; 0·60] | ·· | ·· | ·· | ·· | ·· | ·· | ·· | ·· |
| 0·39  [0·08; 1·94] | 0·81  [0·17; 4·00] | 0·83  [0·18; 3·79] | Teico | ·· | ·· | ·· | ·· | 0·40  [0·07; 2·34] | ·· | 0·37  [0·10; 1·31] | 0·20  [0·04; 1·10] | ·· | ·· | ·· | ·· |
| 0·36  [0·03; 5·04] | 0·75  [0·06; 9·94] | 0·76  [0·06; 9·72] | 0·92  [0·05; 17·05] | Nita | ·· | ·· | 0·56  [0·05; 6·99] | ·· | ·· | ·· | ·· | ·· | ·· | ·· | ·· |
| 0·30  [0·06; 1·40] | 0·63  [0·14; 2·72] | 0·64  [0·16; 2·57] | 0·77  [0·12; 4·84] | 0·84  [0·05; 14·59] | FMT | ·· | 1·80  [0·38; 8·54] | ·· | ·· | 0·04  [0·00; 0·43] | ·· | 0·06  [0·01; 0·59] | 0·19  [0·01; 5·24] | 0·22  [0·02; 2·12] | ·· |
| 0·24  [0·10; 0·59] | 0·51  [0·25; 1·02] | 0·52  [0·31; 0·87] | 0·62  [0·14; 2·87] | 0·68  [0·05; 8·69] | 0·81  [0·20; 3·29] | Suro | 0·83  [0·56; 1·22] | ·· | ·· | ·· | ·· | ·· | ·· | ·· | ·· |
| 0·20  [0·09; 0·45] | 0·42  [0·23; 0·76] | 0·43  [0·31; 0·60] | 0·52  [0·12; 2·26] | 0·56  [0·05; 6·99] | 0·67  [0·17; 2·58] | 0·83  [0·56; 1·22] | Vanco125 | 0·92  [0·54; 1·59] | ·· | ·· | ·· | ·· | ·· | ·· | ·· |
| 0·17  [0·07; 0·37] | 0·35  [0·16; 0·76] | 0·35  [0·19; 0·66] | 0·42  [0·10; 1·72] | 0·46  [0·04; 6·06] | 0·55  [0·14; 2·23] | 0·68  [0·35; 1·31] | 0·82  [0·48; 1·40] | Metro | 0·88  [0·17; 4·52] | 1·00  [0·25; 4·06] | 0·80  [0·37; 1·71] | ·· | ·· | ·· | ·· |
| 0·14  [0·02; 0·90] | 0·30  [0·05; 1·87] | 0·31  [0·05; 1·79] | 0·37  [0·04; 3·21] | 0·40  [0·02; 8·56] | 0·48  [0·06; 4·16] | 0·59  [0·10; 3·49] | 0·72  [0·13; 4·04] | 0·88  [0·17; 4·52] | Metro+rif | ·· | ·· | ·· | ·· | ·· | ·· |
| 0·12  [0·03; 0·45] | 0·24  [0·06; 0·92] | 0·25  [0·07; 0·86] | 0·30  [0·08; 1·06] | 0·32  [0·02; 5·27] | 0·39  [0·08; 1·80] | 0·48  [0·14; 1·67] | 0·58  [0·17; 1·90] | 0·70  [0·23; 2·13] | 0·80  [0·11; 5·82] | Vanco500 | 0·50  [0·14; 1·87] | 1·37  [0·28; 6·81] | ·· | ·· | 0·35  [0·06; 2·01] |
| 0·12  [0·04; 0·34] | 0·24  [0·08; 0·71] | 0·25  [0·09; 0·65] | 0·29  [0·07; 1·18] | 0·32  [0·02; 4·68] | 0·38  [0·08; 1·76] | 0·47  [0·18; 1·27] | 0·57  [0·23; 1·42] | 0·70  [0·33; 1·48] | 0·79  [0·13; 4·85] | 0·99  [0·33; 3·00] | FA | ·· | ·· | ·· | ·· |
| 0·09  [0·01; 0·65] | 0·19  [0·03; 1·33] | 0·20  [0·03; 1·28] | 0·24  [0·03; 1·70] | 0·26  [0·01; 5·85] | 0·31  [0·05; 2·08] | 0·38  [0·06; 2·48] | 0·46  [0·07; 2·88] | 0·56  [0·09; 3·40] | 0·64  [0·06; 7·35] | 0·80  [0·17; 3·77] | 0·81  [0·13; 5·02] | Vanco+BL | ·· | ·· | ·· |
| 0·06  [0·00; 2·21] | 0·12  [0·00; 4·47] | 0·12  [0·00; 4·43] | 0·14  [0·00; 6·47] | 0·16  [0·00; 12·61] | 0·19  [0·01; 5·24] | 0·23  [0·01; 8·57] | 0·28  [0·01; 10·14] | 0·34  [0·01; 12·59] | 0·38  [0·01; 20·52] | 0·48  [0·01; 18·98] | 0·48  [0·01; 19·09] | 0·60  [0·01; 27·91] | FMT-L | ·· | ·· |
| 0·07  [0·00; 1·02] | 0·14  [0·01; 2·05] | 0·14  [0·01; 2·01] | 0·17  [0·01; 3·13] | 0·18  [0·00; 7·03] | 0·22  [0·02; 2·12] | 0·27  [0·02; 3·89] | 0·32  [0·02; 4·57] | 0·39  [0·03; 5·72] | 0·45  [0·02; 10·39] | 0·56  [0·04; 8·79] | 0·57  [0·04; 8·83] | 0·70  [0·04; 13·62] | 1·17  [0·02; 67·00] | FMT+bez | ·· |
| 0·04  [0·00; 0·37] | 0·08  [0·01; 0·76] | 0·09  [0·01; 0·74] | 0·10  [0·01; 0·90] | 0·11  [0·00; 3·04] | 0·14  [0·01; 1·39] | 0·17  [0·02; 1·43] | 0·20  [0·02; 1·67] | 0·25  [0·03; 1·95] | 0·28  [0·02; 3·94] | 0·35  [0·06; 2·01] | 0·35  [0·04; 2·80] | 0·44  [0·04; 4·49] | 0·73  [0·01; 42·93] | 0·62  [0·02; 16·27] | Bacit |

# ***Table S31:* League table of possible treatments in recurrence, vancomycin dosages subgroup analysis**

Possible treatments are arranged in descending order of the P score in the blue bars. P scores are given in brackets after treatment names. Direct comparisons are shown above these blue bars, whereas below, the direct and indirect (estimated) ones are pooled together as network estimates. Odds ratios are given in the cells. We compare one treatment on the left side with another one on the right side, indicating a greater odds ratio of recovery. The 95% confidence interval is shown in brackets. Significant results are marked in green.

Bacit=Bacitracin; FA=Fusidic acid; Fidax=Fidaxomicin; FMT=Fecal microbiota transplantation; FMT-Bez=Fecal microbiota transplantation+Bezlotoxumab; FMT-L=Fecal microbiota transplantation+Lactobacillus; Metro=Metronidazole; Metro+rif=Metronidazole+Rifampin; Nita=Nitazoxanide; Ridin=Ridinilazole; Suro=Surotomycin; Teico=Teicoplanin; Tolev=Tolevamer; Vanco125=Vancomycin 125 mg four times daily; Vanco500=Vancomycin 500 mg four times daily; Vanco+BL=Vancomycin+bowel lavage


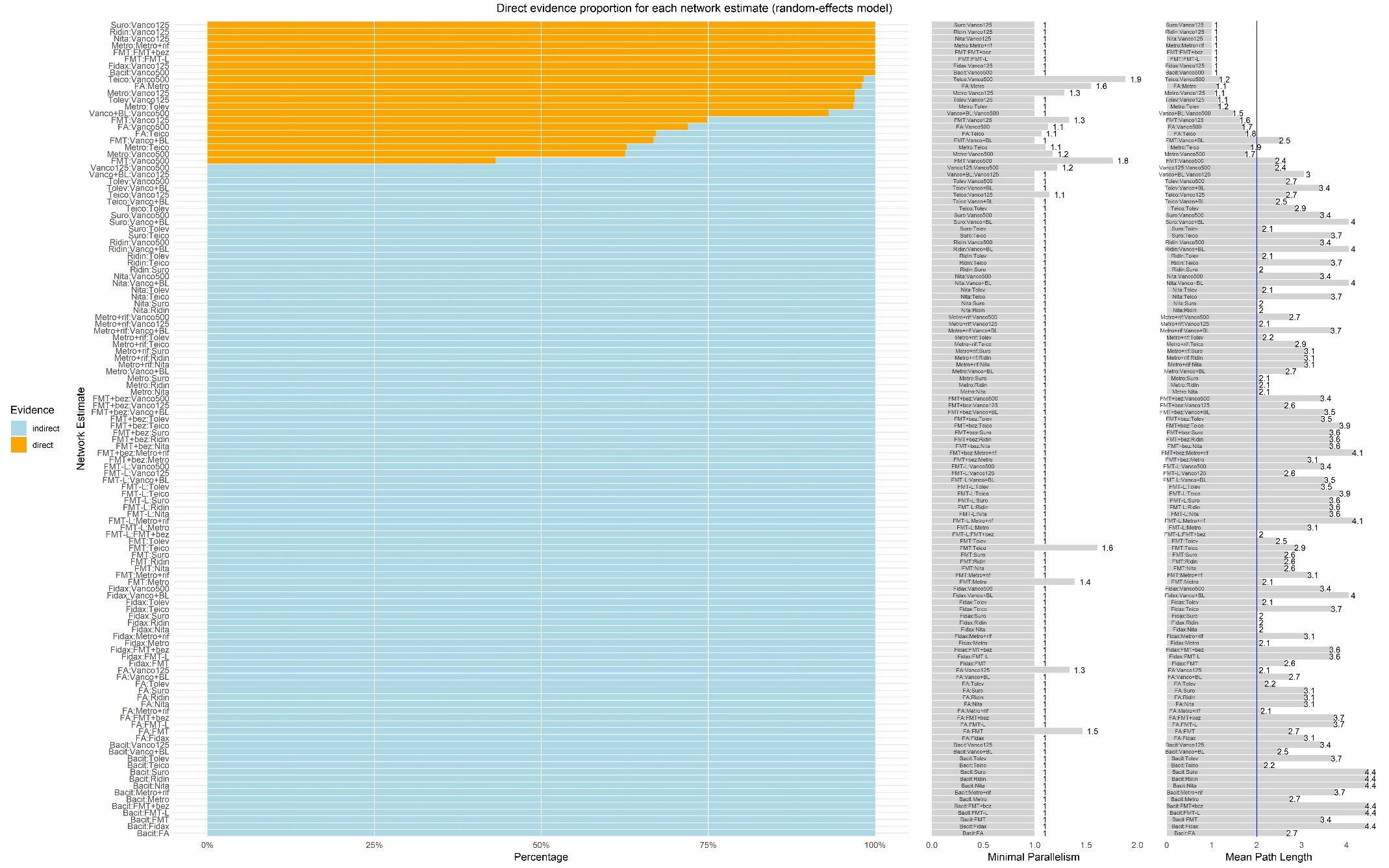


# ***Figure S42:* Evidence plot of the treatments in case of recurrence, vancomycin dosages subgroup analysis**

It shows what percentage of the result comes from the direct comparison and how much comes from the indirect/estimated data. The direct ones are marked orange, while the estimated ones are marked blue. In the Mean Path Length diagram, if the given comparison is greater than 2, then these network estimations should be interpreted carefully. Higher values of parallelism indicate greater robustness of the estimate.

Bacit=Bacitracin; FA=Fusidic acid; Fidax=Fidaxomicin; FMT=Fecal microbiota transplantation; FMT-Bez=Fecal microbiota transplantation+Bezlotoxumab; FMT-L=Fecal microbiota transplantation+Lactobacillus; Metro=Metronidazole; Metro+rif=Metronidazole+Rifampin; Nita=Nitazoxanide; Ridin=Ridinilazole; Suro=Surotomycin; Teico=Teicoplanin; Tolev=Tolevamer; Vanco125=Vancomycin 125 mg four times daily; Vanco500=Vancomycin 500 mg four times daily; Vanco+BL=Vancomycin+bowel lavage


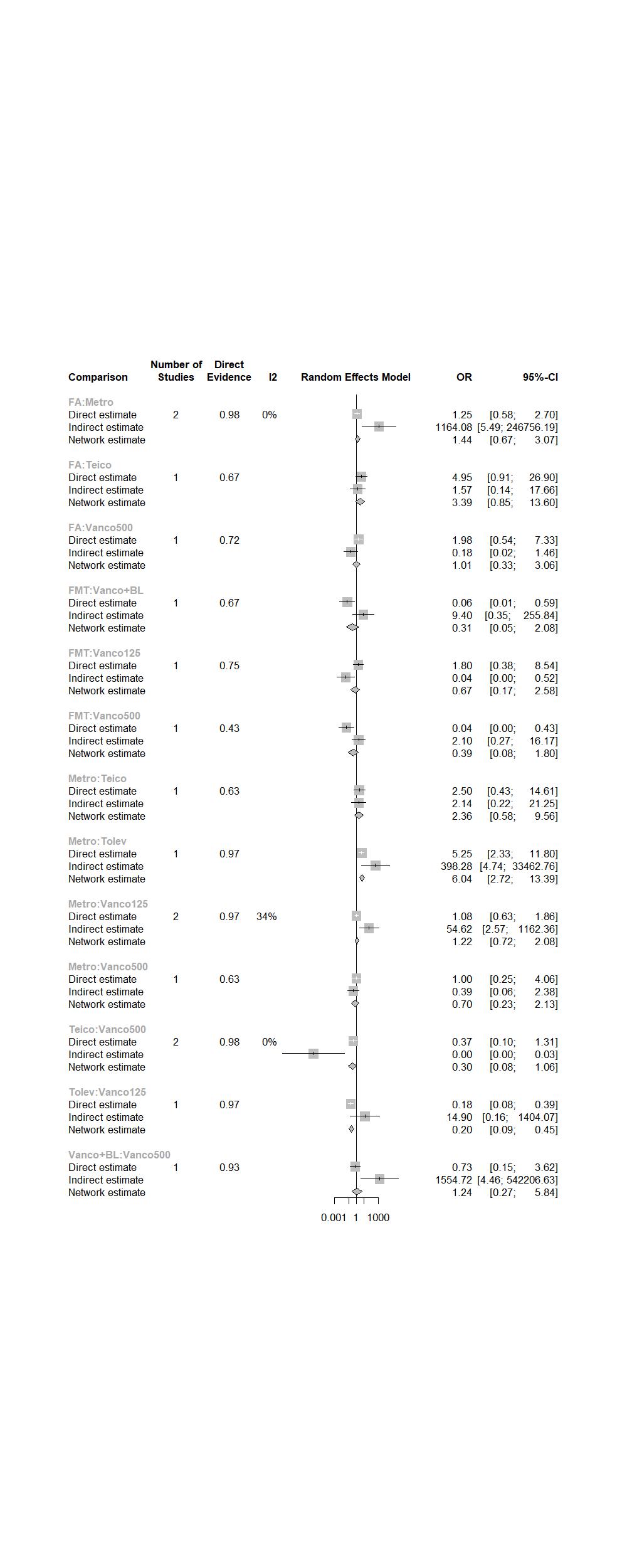


# ***Figure S43:* Forest plot for results of consistency analysis for the treatments in case of recurrence, vancomycin dosages subgroup analysis**

The Forest plot shows the result of the consistency analysis. The direct and indirect comparisons do not contradict each other, so the network can be considered consistent.

FA=Fusidic acid; FMT=Fecal microbiota transplantation; Metro=Metronidazole; Teico=Teicoplanin; Tolev=Tolevamer; Vanco125=Vancomycin 125 mg four times daily; Vanco500=Vancomycin 500 mg four times daily; Vanco+BL=Vancomycin+bowel lavage


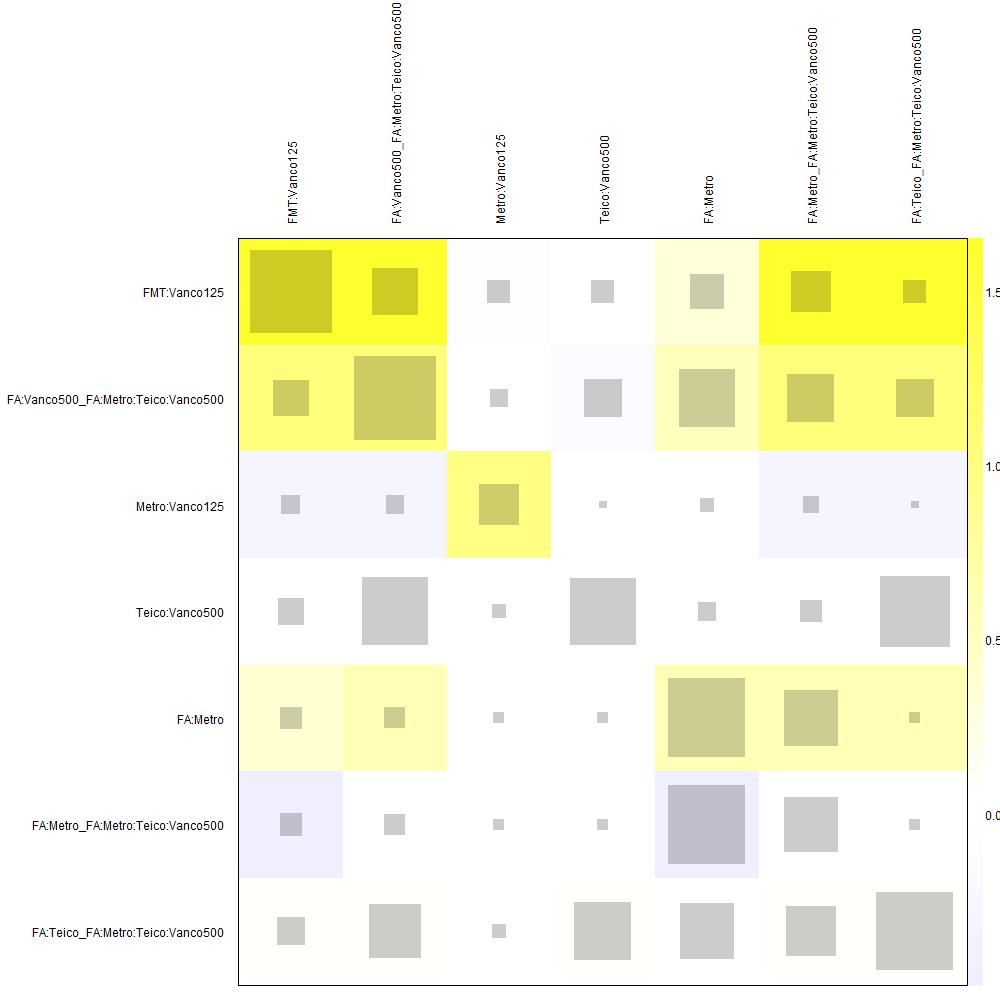


# ***Figure S44:* Net heat plot of the treatments in case of recurrence, vancomycin dosages subgroup analysis**

It assesses if there is a consistency problem or not. It shows the difference between direct estimation and the network estimation. The right column shows that the redder the difference, the more inconsistent the result. The area of a gray square represents the contribution of the direct estimate from a design in the column to the network estimate in the row.

FA=Fusidic acid; Fidax=Fidaxomicin; FMT=Fecal microbiota transplantation; Metro=Metronidazole; Teico=Teicoplanin; Vanco125=Vancomycin 125 mg four times daily; Vanco500=Vancomycin 500 mg four times daily


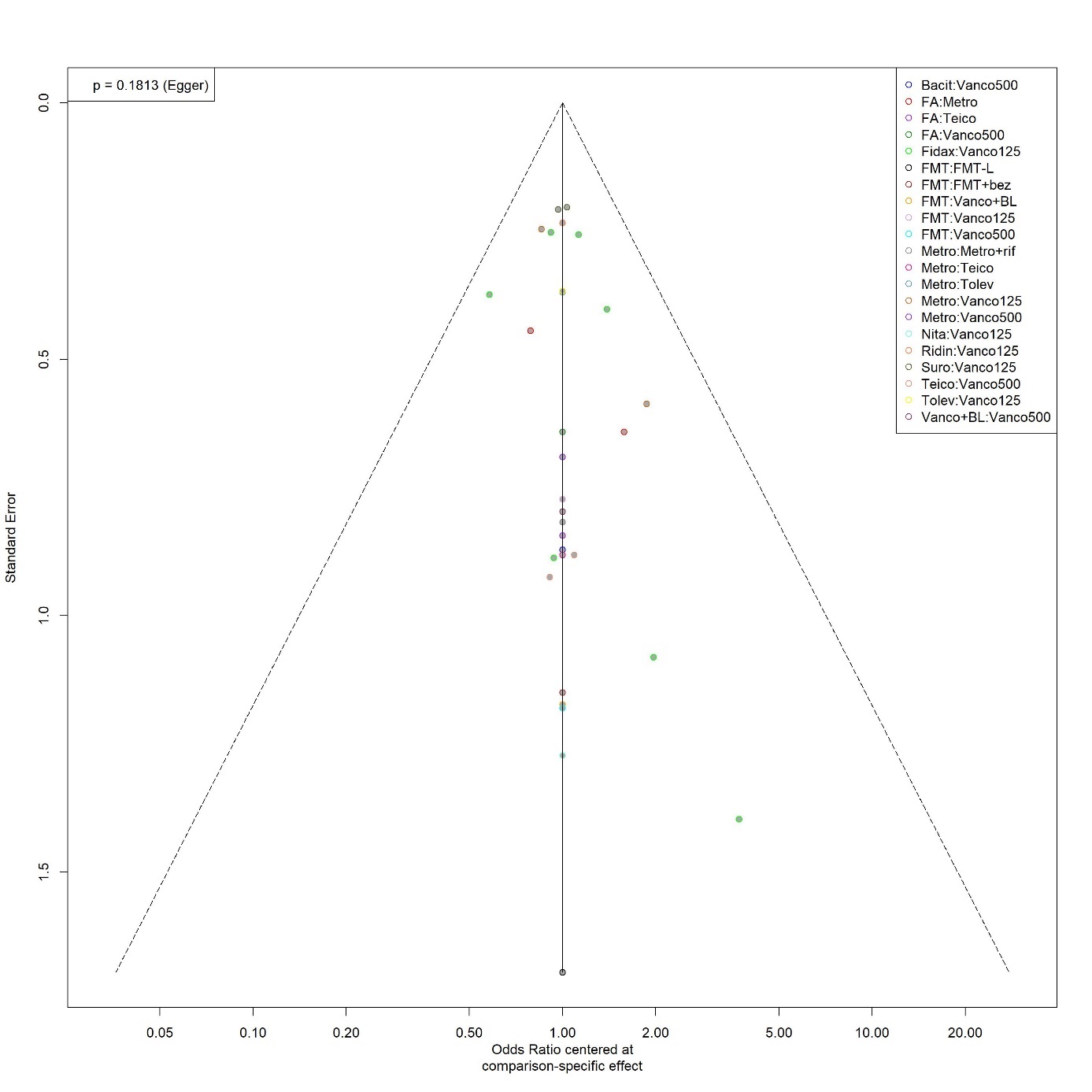


# ***Figure S45:* Funnel plot of the treatments in case of recurrence, vancomycin dosages subgroup analysis**

It shows the extent to which the analysis is affected by the small study effect. If the points are under the tent, symmetrically distributed, and the Egger number is bigger than 0.05, in that case the small study effect does not affect our analysis.

Bacit=Bacitracin; FA=Fusidic acid; Fidax=Fidaxomicin; FMT=Fecal microbiota transplantation; FMT-Bez=Fecal microbiota transplantation+Bezlotoxumab; FMT-L=Fecal microbiota transplantation+Lactobacillus; Metro=Metronidazole; Metro+rif=Metronidazole+Rifampin; Nita=Nitazoxanide; Ridin=Ridinilazole; Suro=Surotomycin; Teico=Teicoplanin; Tolev=Tolevamer; Vanco125=Vancomycin 125 mg four times daily; Vanco500=Vancomycin 500 mg four times daily; Vanco+BL=Vancomycin+bowel lavage

# **Supplementary Results S3 – Additional analysis of prevention therapies against the development of CDI**

We also separately examined probiotics as the most commonly used preventative treatment. We divided probiotics into three sub-groups: *Lactobacillaceae,* *Saccharomyces*, or combined therapy (*Lactobacillaceae* and *Saccharomyces*).

We examined 4 interventions from 18 studies^8,9,18–25,10–17^ involving 6293 patients. There were six total potential pairwise comparisons and three pairwise comparisons with direct data (Table S32). The network plot is shown in Figure S46.

| **Summary of network table** | | | | |
| --- | --- | --- | --- | --- |
| **Characteristic** | | | | **Value** |
| Number of Interventions | | | | 4 |
| Number of Studies | | | | 18 |
| Total Number of Patients in Network | | | | 6293 |
| Total Possible Pairwise Comparisons | | | | 6 |
| Total Number of Pairwise Comparisons with Direct Data | | | | 3 |
| Number of Two-arm Studies | | | | 18 |
| Number of Multi-Arms Studies | | | | 0 |
| Total Number of Events in Network | | | | 6040 |
| Number of Studies With No Zero Events | | | | 18 |
| Number of Studies With At Least One Zero Event | | | | 0 |
| Number of Studies With All Zero Events | | | | 0 |
|  | 4 |  |  | |
| **Treatment** | **Studies (n)** | **Events (n)** | **Patients (n)** | |
| Lacto | 8 | 1006 | 1026 | |
| Mix probi | 4 | 1576 | 1502 | |
| Plac | 18 | 3000 | 3136 | |
| Saccharo | 6 | 458 | 529 | |

***Table S32:* Network summary table of possible treatments for prevention; probiotics compared with placebo**

Key data of analysis and treatments.

Lacto=*Lactobacillaceae*; Mix probi=Mixed probiotics; Plac=Placebo; Saccharo=*Saccharomyces*


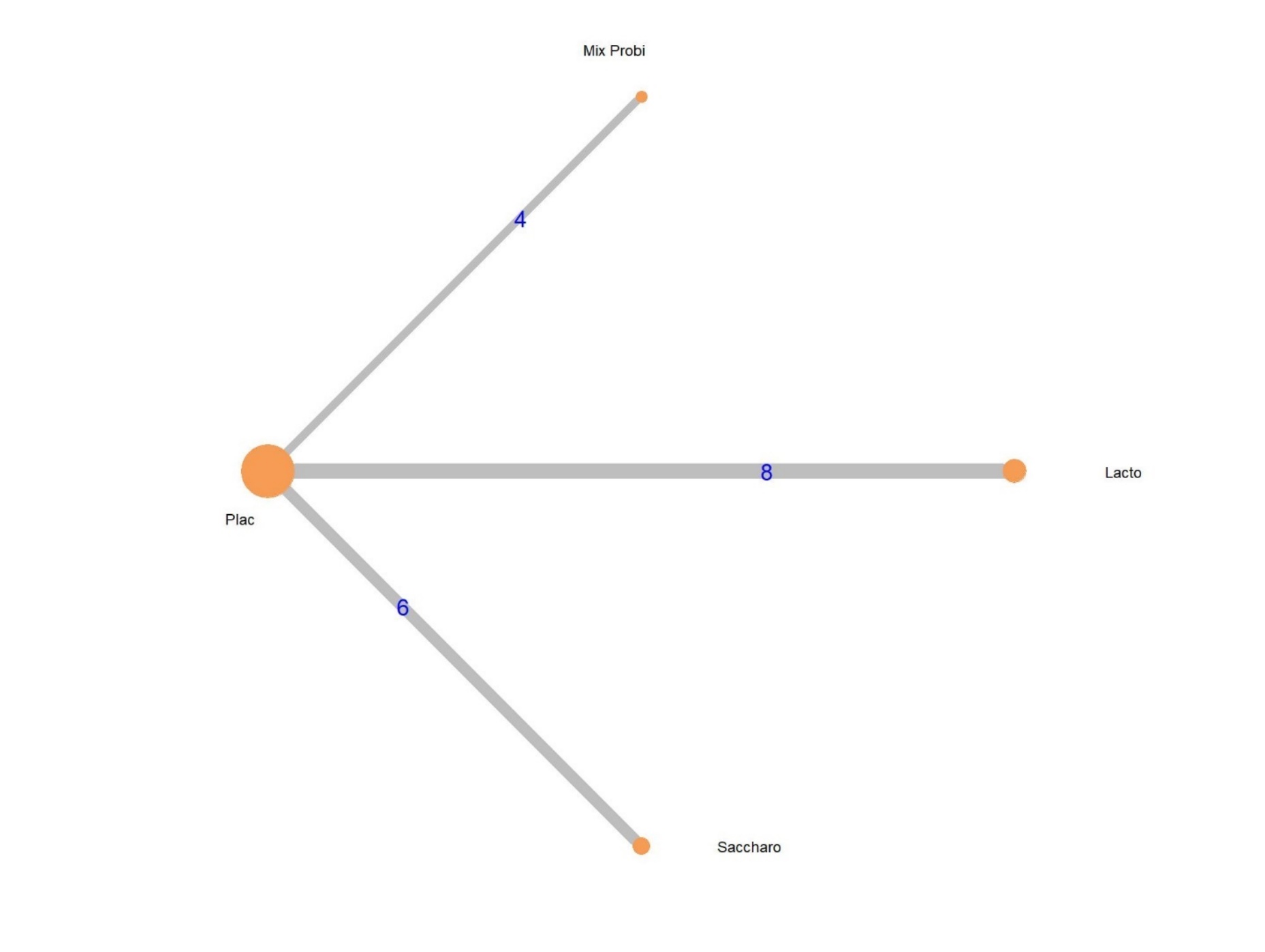


***Figure S46:* Network plot of possible treatments for prevention; probiotics compared with placebo**

Every knot represents a different therapy for CDI. The larger a knot, the more studies included that treatment. Every edge compares different therapies. The width and the number above indicate how many studies investigated this comparison.

Lacto=*Lactobacillaceae*; Mix probi=Mixed probiotics; Plac=Placebo; Saccharo=*Saccharomyces*

Tables S33 and S34 show that *Lactobacillaceae* demonstrated the highest efficacy in preventing CDI over the mixed probiotic, whereas *Saccharomyces* exhibited the least efficacy. *Lactobacillaceae* proved to be significantly more effective than placebo based on Table S33.

| Lacto  (0·89819 | ·· | 2·37  [1·07; 5·24] | ·· |
| --- | --- | --- | --- |
| 1·48  [0·46; 4·75] | Mix Probi  (0·5639) | 1·61  [0·68; 3·79] | ·· |
| 2·37  [1·07; 5·24] | 1·61  [0·68; 3·79] | Plac  (0·3247) | 1·22  [0·58; 2·58] |
| 2·90  [0·97; 8·62] | 1·96  [0·63; 6·13] | 1·22  [0·58; 2·58] | Saccharo  (0·2133) |

***Table S33:* League table of possible treatments for prevention; probiotics compared with placebo**

Possible treatments are arranged in descending order of the P-score in the blue bars. P-scores are given in brackets after treatment names. Direct comparisons are shown above these blue bars, whereas below, direct and indirect (estimated) ones are pooled together as network estimates. Odds ratios are given in the cells. We compare one treatment on the left side with another one on the right side, indicating a greater odds ratio of recovery. The 95% confidence interval is shown in brackets. Significant results are marked in green.

Lacto=*Lactobacillaceae*; Mix probi=Mixed probiotics; Plac=Placebo; Saccharo=*Saccharomyces*

|  | P-score (random) |
| --- | --- |
| Lacto | 0·8981 |
| Mix probi | 0·5639 |
| Plac | 0·3247 |
| Saccharo | 0·2133 |

*Table S34:* P-score table (SUCRA) of the possible treatments in case of prevention; probiotics compared with placebo

The possible therapies are ranked based on the P-score. P-score shows the average confidence with which we can say that one treatment is better than another. P-score can range from 0 to 1.

Lacto=*Lactobacillaceae*; Mix probi=Mixed probiotics; Plac=Placebo; Saccharo=*Saccharomyces*

The evidence plot and funnel plot for the analysis can be viewed in Figures S47 and S48.


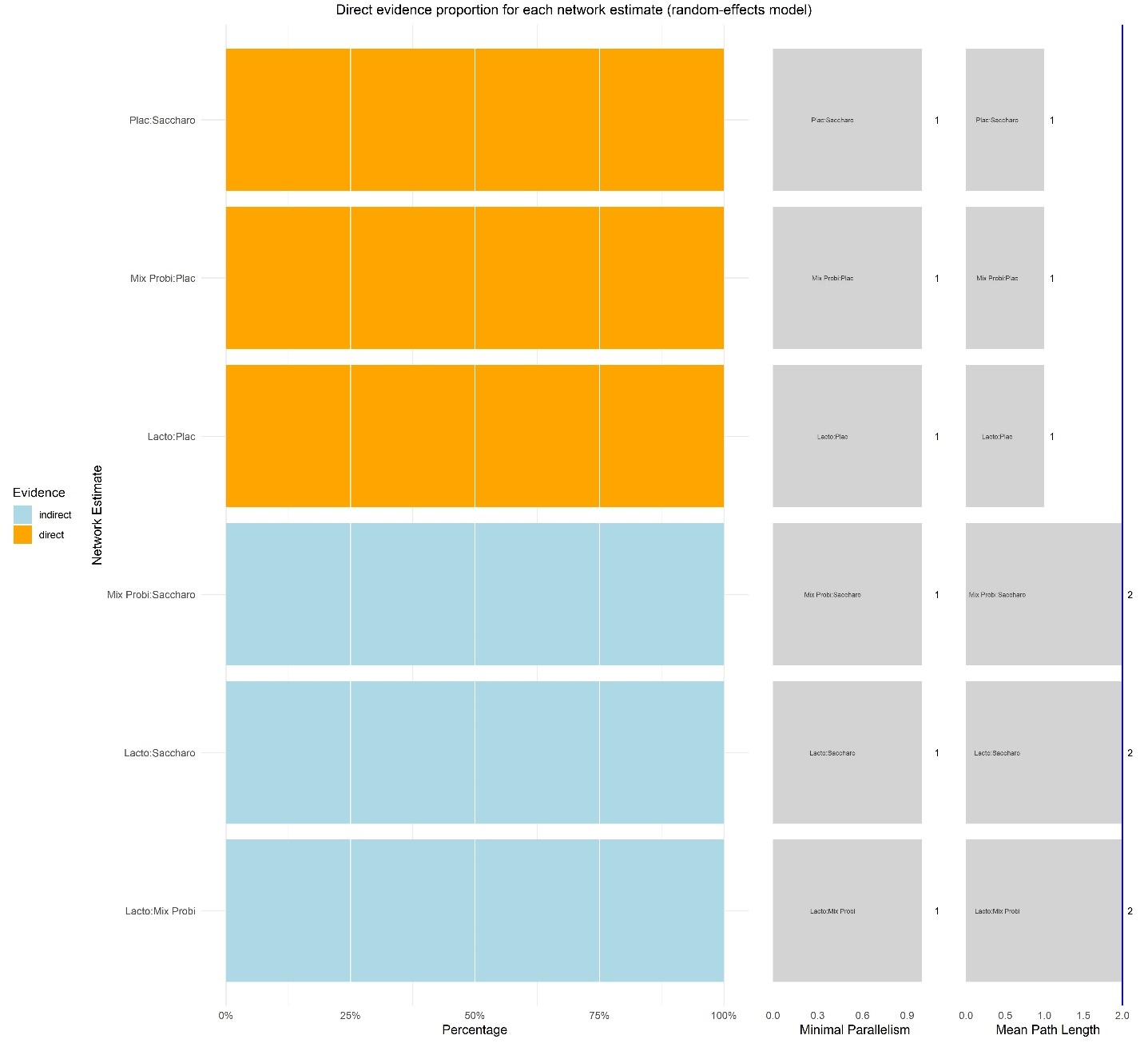


*Figure S47:* Evidence plot of the treatments in case of prevention

It shows what percentage of the result comes from the direct comparison and how much comes from the indirect/estimated data. The direct ones are marked orange, while the estimated ones are marked blue. In the Mean Path Length diagram, if the given comparison is greater than 2, then these network estimations should be interpreted carefully. Higher values of parallelism indicate greater robustness of the estimate.

Lacto=*Lactobacillaceae*; Mix probi=Mixed probiotics; Plac=Placebo; Saccharo=*Saccharomyces*


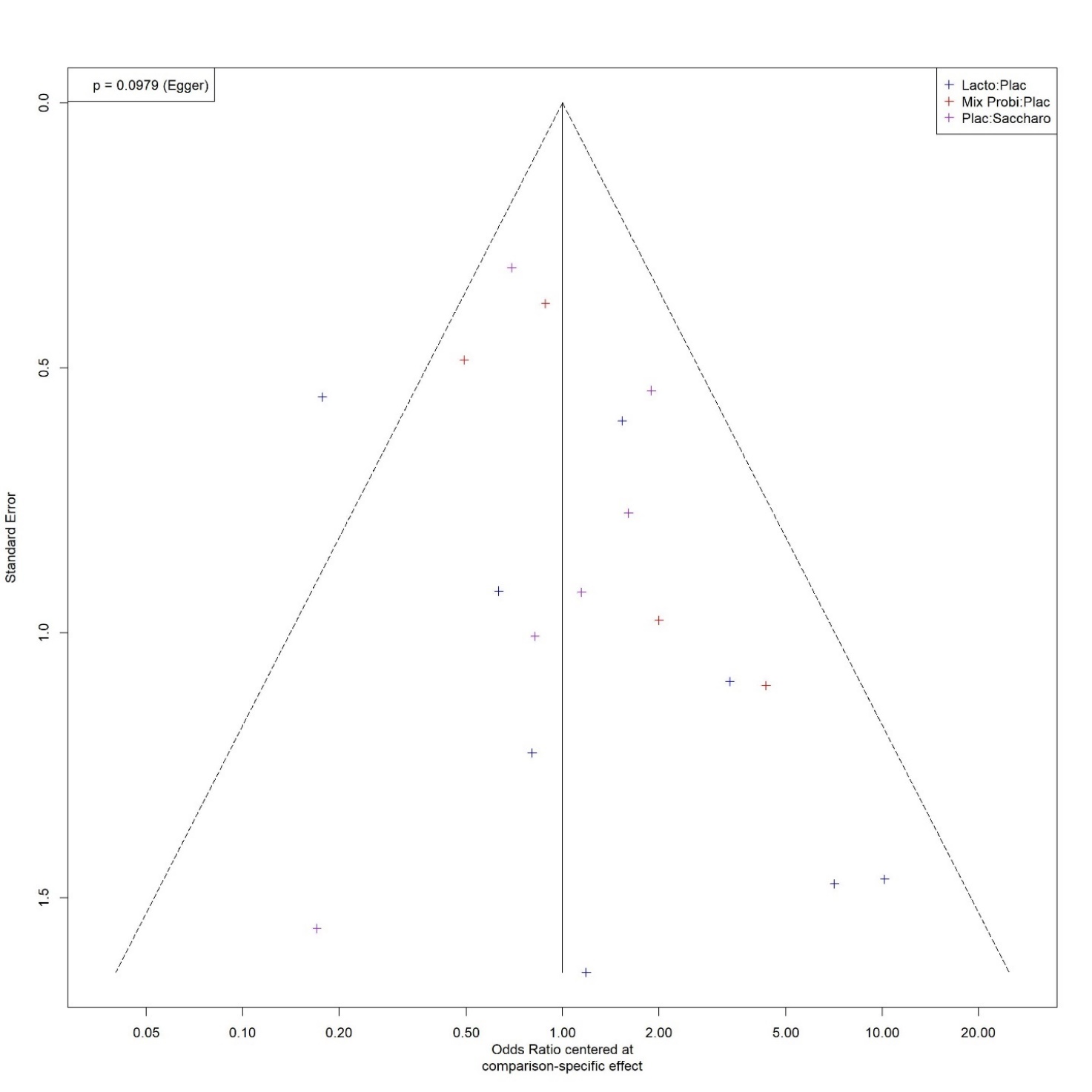


*Figure S48:* Funnel plot of the treatments in case of prevention

It shows the extent to which the analysis is affected by the small study effect. If the points are under the tent, symmetrically distributed, and the Egger number is bigger than 0.05, in that case the small study effect does not affect our analysis.

Lacto=*Lactobacillaceae*; Mix probi=Mixed probiotics; Plac=Placebo; Saccharo=*Saccharomyces*

The tests for small study effects were visualized by Funnel plot. The Egger's test (p > 0·05) showed no small study effect (Figure S48).

Furthermore, we compared probiotics with placebo through pairwise comparisons. We compared Lactobacillus with placebo in seven RCTs involving 2088 patients^8,10,13,15–17,19^. *Lactobacillaceae* showed no statistically significant efficacy in preventing CDI over placebo (Figure S49).


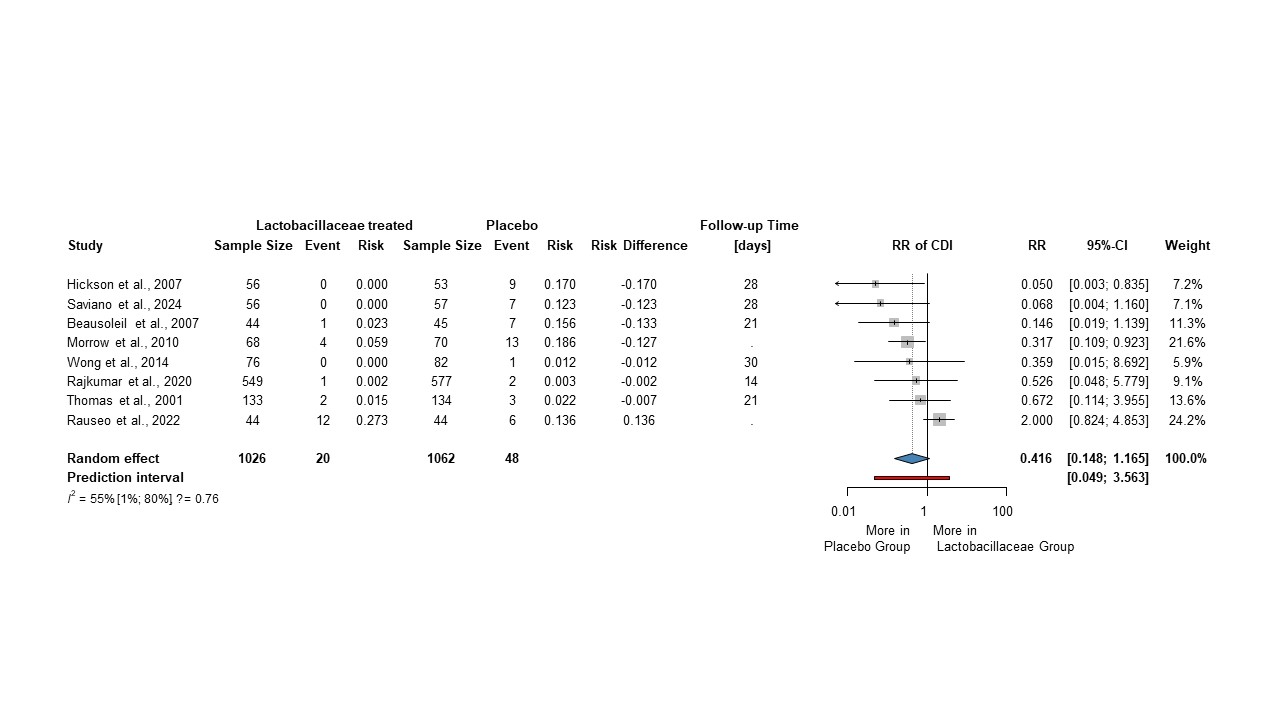


*Figure S49:* Forest plot on cure rates-*Lactobacillaceae* compared with placebo

The chance of recovery is not significantly better in the *Lactobacillaceae* arm compared to the placebo arm.

RR=Risk ratio. CI=confidence interval. CDI=*Clostridioides difficile* infection.

A comparison of the efficacy of *Saccharomyces* and placebo in CDI prevention in six studies with 1001 patients^9,11,12,14,18,23^ revealed no statistically significant difference (Figure S50).

*Figure S50:* Forest plot on cure rates-*Saccharomyces* compared with placebo

The chance of recovery is not significantly better in the *Saccharomyces* arm, compared to the placebo arm.

RR=Risk ratio. CI=confidence interval. CDI=*Clostridioides difficile* infection.

Mixed probiotics showed no statistical superiority over placebo in comparative analysis of data from four studies involving 3204 patients^20–22,24^ (Figure S51).

*Figure S51:* Forest plot on cure rates-Mixed probiotics compared with placebo

The chance of recovery in the mixed probiotic arm is not significantly better compared to the placebo arm. RR=Risk ratio. CI=confidence interval. CDI=*Clostridioides difficile* infection.


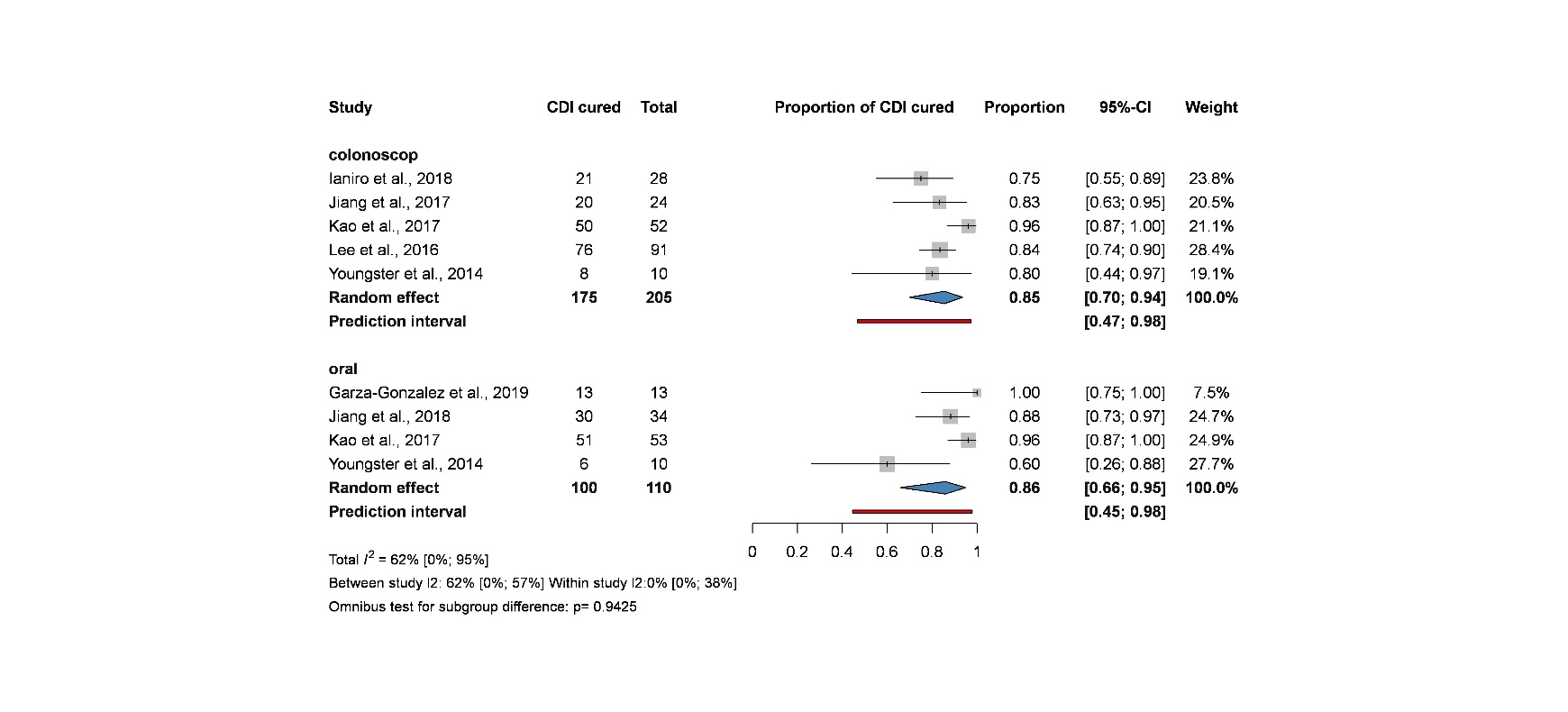


# ***Figure S52:* Forest plots on FMT cure rates via colonoscopy or oral administration**

There is no difference in the effectiveness of FMT between the oral and colonoscopic methods.

FMT=Fecal microbiota transplantation, CDI=*Clostridioides difficile* infection.

# **Supplementary Results S4 – Additional analysis of two armed FMT studies**

To further study the colonoscopic and oral methods of FMT, we compared two articles where the difference between the two methods was examined as a two-arm study.^26,27^ The difference between the cure rate was given by Risk ratio, which it was 1·01 with a confidence interval of 0·999 and 1·333 (Figure S53). The result is not statistically significant and there is no clinically significant difference between the two methods.


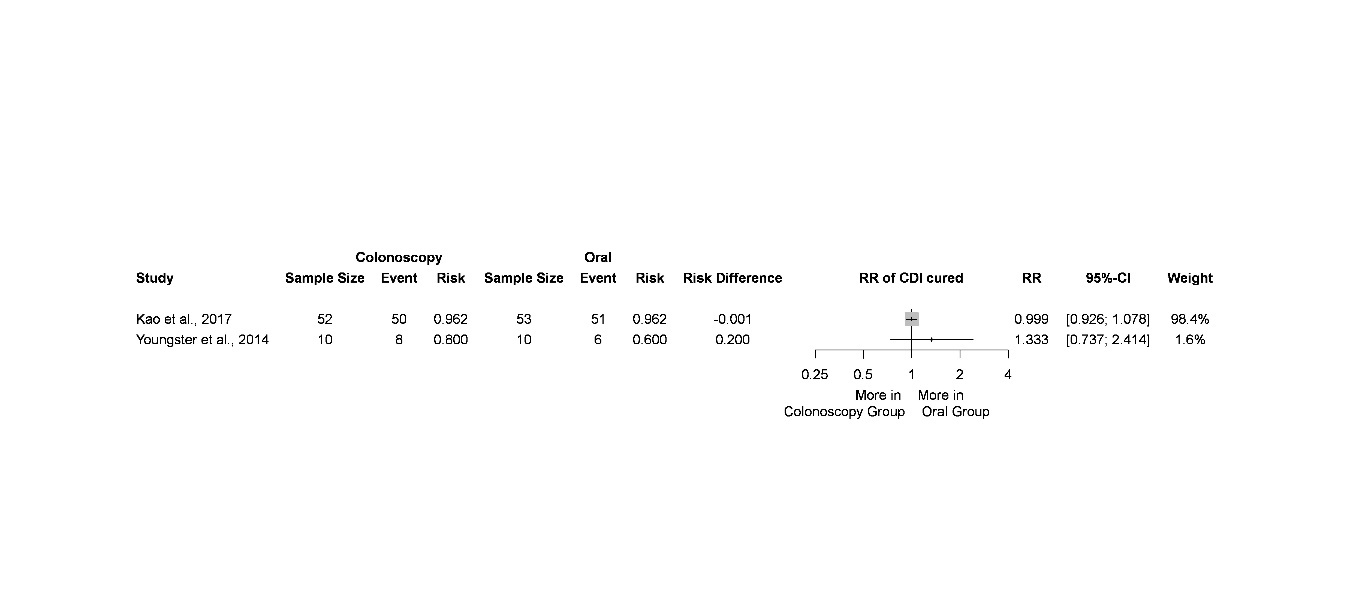


*Figure S53:* Direct comparison of the colonoscopy way and the oral way in the case of cure rate using FMT

There is no difference between the effectiveness of FMT given orally and FMT given with colonoscope.

FMT=Fecal microbiota transplantation, CDI=*Clostridioides difficile* infection.

Although the Prisma 2020 statement^28^ allows for a statistical analysis of two studies, the small number of items is definitely a limitation of this result. That said, this two-arm analysis also supports our finding that no significant difference between the two methods can be detected.

# ***Table S35:* Risk of Bias assessment**


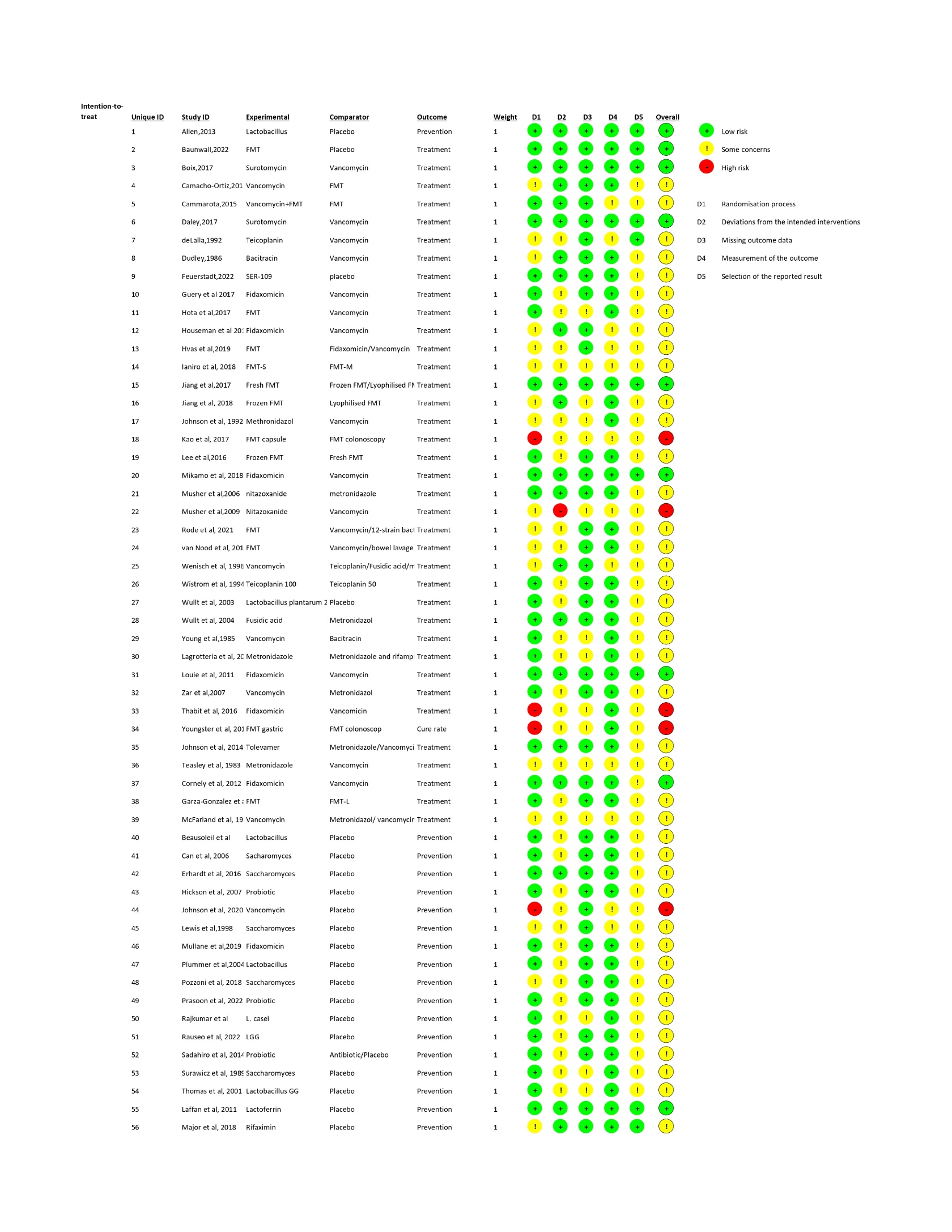


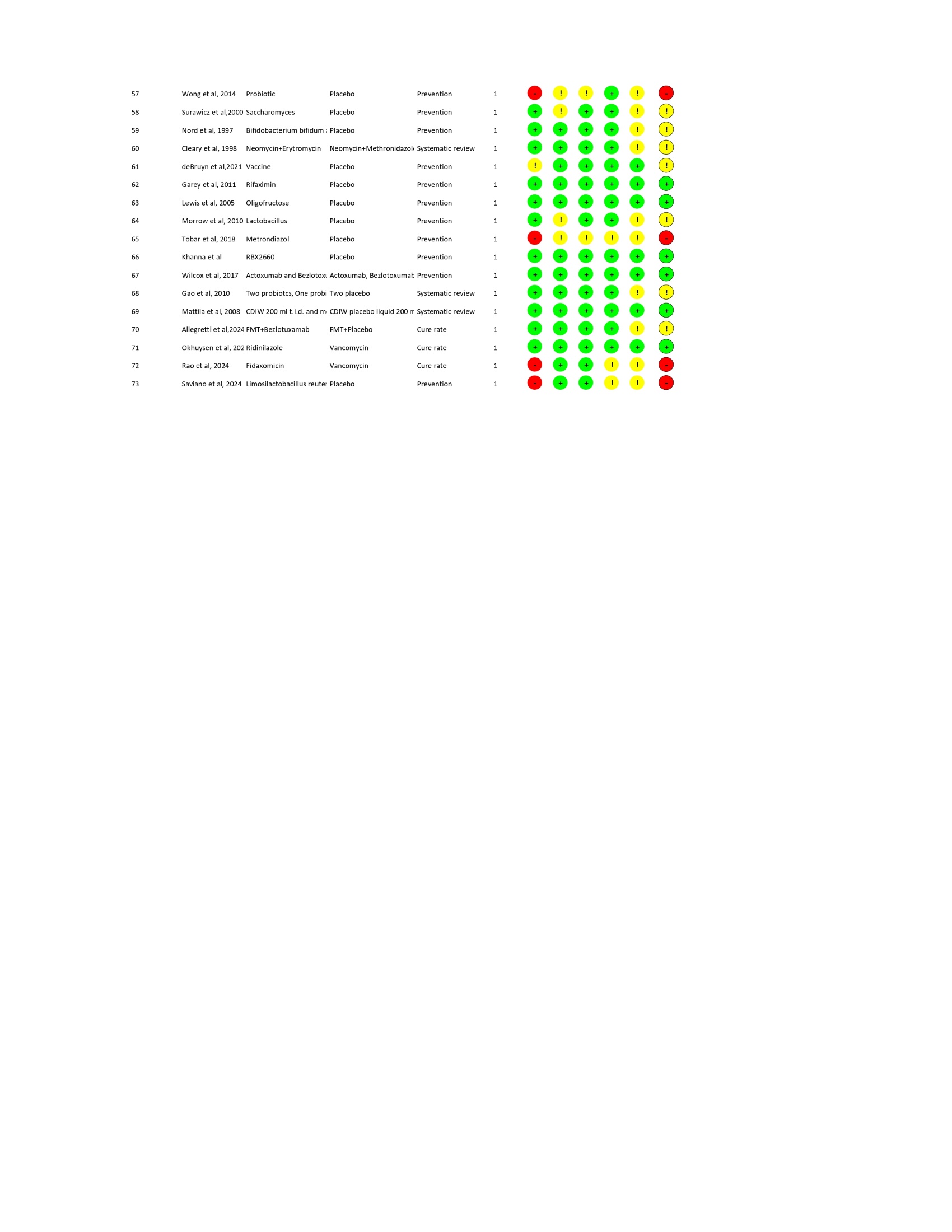


# ***Table S36:* Grade: cure rate**

| Comparison | Number of studies | Within-study bias | Reporting bias | Indirectness | Imprecision | Heterogeneity | Incoherence | Confidence rating |
| --- | --- | --- | --- | --- | --- | --- | --- | --- |
| Bacit:Vanco | 2 | Some concerns | Some concerns | No concerns | Major concerns | No concerns | No concerns | Very low |
| FA:Metro | 2 | Some concerns | Some concerns | No concerns | Major concerns | No concerns | No concerns | Very low |
| FA:Teico | 1 | Some concerns | Some concerns | No concerns | Major concerns | No concerns | No concerns | Very low |
| FA:Vanco | 1 | Some concerns | Some concerns | No concerns | Major concerns | No concerns | No concerns | Very low |
| Fidax:FMT | 1 | Some concerns | Some concerns | No concerns | No concerns | Major concerns | No concerns | Very low |
| FMT:Plac | 1 | Some concerns | Some concerns | No concerns | No concerns | No concerns | No concerns | High |
| FMT:RBT | 1 | Some concerns | Some concerns | No concerns | No concerns | Some concerns | No concerns | Moderate |
| FMT:Vanco | 5 | Some concerns | Some concerns | No concerns | No concerns | No concerns | Major concerns | Very low |
| FMT:Vanco+BL | 1 | Some concerns | Some concerns | No concerns | No concerns | No concerns | No concerns | High |
| Fidax:Vanco | 8 | Some concerns | Some concerns | No concerns | No concerns | Major concerns | No concerns | Very low |
| Metro:Metro+rif | 1 | Some concerns | Some concerns | No concerns | Major concerns | No concerns | No concerns | Very low |
| Metro:Nita | 1 | Some concerns | Some concerns | No concerns | Major concerns | No concerns | No concerns | Very low |
| Metro:Plac | 1 | Some concerns | Some concerns | No concerns | Major concerns | No concerns | No concerns | Very low |
| Metro:Teico | 1 | Some concerns | Some concerns | No concerns | Major concerns | No concerns | No concerns | Very low |
| Metro:Tolev | 1 | Some concerns | Some concerns | No concerns | No concerns | Some concerns | No concerns | Moderate |
| Metro:Vanco | 5 | Some concerns | Some concerns | No concerns | Major concerns | No concerns | No concerns | Very low |
| Nita:Vanco | 1 | Some concerns | Some concerns | No concerns | Major concerns | No concerns | No concerns | Very low |
| Plac:SER | 1 | Some concerns | Some concerns | No concerns | No concerns | Major concerns | No concerns | Very low |
| Plac:Vanco | 1 | Some concerns | Some concerns | No concerns | Major concerns | No concerns | Major concerns | Very low |
| RBT:Vanco | 1 | Some concerns | Some concerns | No concerns | Major concerns | No concerns | No concerns | Very low |
| Ridin:Vanco | 1 | No concerns | Some concerns | No concerns | Major concerns | No concerns | No concerns | Very low |
| Suro:Vanco | 2 | No concerns | Some concerns | No concerns | Major concerns | No concerns | No concerns | Very low |
| Teico:Vanco | 2 | Some concerns | Some concerns | No concerns | Major concerns | No concerns | No concerns | Very low |
| Tolev:Vanco | 1 | Some concerns | Some concerns | No concerns | No concerns | No concerns | No concerns | High |
| Vanco:Vanco+BL | 1 | Some concerns | Some concerns | No concerns | Major concerns | No concerns | No concerns | Very low |
| Bacit:FA | 0 | Some concerns | Some concerns | No concerns | Major concerns | No concerns | No concerns | Very low |
| Bacit:FMT | 0 | Some concerns | Some concerns | No concerns | No concerns | No concerns | No concerns | High |
| Bacit:Fidax | 0 | Some concerns | Some concerns | No concerns | Major concerns | No concerns | No concerns | Very low |
| Bacit:Metro | 0 | Some concerns | Some concerns | No concerns | Major concerns | No concerns | No concerns | Very low |
| Bacit:Metro+rif | 0 | Some concerns | Some concerns | No concerns | Major concerns | No concerns | No concerns | Very low |
| Bacit:Nita | 0 | Some concerns | Some concerns | No concerns | Major concerns | No concerns | No concerns | Very low |
| Bacit:Plac | 0 | Some concerns | Some concerns | No concerns | Major concerns | No concerns | No concerns | Very low |
| Bacit:RBT | 0 | Some concerns | Some concerns | No concerns | Major concerns | No concerns | No concerns | Very low |
| Bacit:Ridin | 0 | Some concerns | Some concerns | No concerns | Major concerns | No concerns | No concerns | Very low |
| Bacit:SER | 0 | Some concerns | Some concerns | No concerns | Major concerns | No concerns | No concerns | Very low |
| Bacit:Suro | 0 | Some concerns | Some concerns | No concerns | Major concerns | No concerns | No concerns | Very low |
| Bacit:Teico | 0 | Some concerns | Some concerns | No concerns | Major concerns | No concerns | No concerns | Very low |
| Bacit:Tolev | 0 | Some concerns | Some concerns | No concerns | Major concerns | No concerns | No concerns | Very low |
| Bacit:Vanco+BL | 0 | Some concerns | Some concerns | No concerns | Major concerns | No concerns | No concerns | Very low |
| FA:FMT | 0 | Some concerns | Some concerns | No concerns | No concerns | No concerns | No concerns | High |
| FA:Fidax | 0 | Some concerns | Some concerns | No concerns | No concerns | Major concerns | No concerns | Very low |
| FA:Metro+rif | 0 | Some concerns | Some concerns | No concerns | Major concerns | No concerns | No concerns | Very low |
| FA:Nita | 0 | Some concerns | Some concerns | No concerns | Major concerns | No concerns | No concerns | Very low |
| FA:Plac | 0 | Some concerns | Some concerns | No concerns | Major concerns | No concerns | No concerns | Very low |
| FA:RBT | 0 | Some concerns | Some concerns | No concerns | Major concerns | No concerns | No concerns | Very low |
| FA:Ridin | 0 | Some concerns | Some concerns | No concerns | Major concerns | No concerns | No concerns | Very low |
| FA:SER | 0 | Some concerns | Some concerns | No concerns | Major concerns | No concerns | No concerns | Very low |
| FA:Suro | 0 | Some concerns | Some concerns | No concerns | Major concerns | No concerns | No concerns | Very low |
| FA:Tolev | 0 | Some concerns | Some concerns | No concerns | Major concerns | No concerns | No concerns | Very low |
| FA:Vanco+BL | 0 | Some concerns | Some concerns | No concerns | Major concerns | No concerns | No concerns | Very low |
| FMT:Metro | 0 | Some concerns | Some concerns | No concerns | No concerns | No concerns | No concerns | High |
| FMT:Metro+rif | 0 | Some concerns | Some concerns | No concerns | No concerns | Some concerns | No concerns | Moderate |
| FMT:Nita | 0 | Some concerns | Some concerns | No concerns | Some concerns | Some concerns | No concerns | Low |
| FMT:Ridin | 0 | Some concerns | Some concerns | No concerns | No concerns | No concerns | No concerns | High |
| FMT:SER | 0 | Some concerns | Some concerns | No concerns | Major concerns | No concerns | No concerns | Very low |
| FMT:Suro | 0 | Some concerns | Some concerns | No concerns | No concerns | No concerns | No concerns | High |
| FMT:Teico | 0 | Some concerns | Some concerns | No concerns | Some concerns | Some concerns | No concerns | Low |
| FMT:Tolev | 0 | Some concerns | Some concerns | No concerns | No concerns | No concerns | No concerns | High |
| Fidax:Metro | 0 | Some concerns | Some concerns | No concerns | No concerns | Major concerns | No concerns | Very low |
| Fidax:Metro+rif | 0 | Some concerns | Some concerns | No concerns | Major concerns | No concerns | No concerns | Very low |
| Fidax:Nita | 0 | Some concerns | Some concerns | No concerns | Major concerns | No concerns | No concerns | Very low |
| Fidax:Plac | 0 | Some concerns | Some concerns | No concerns | No concerns | Some concerns | No concerns | Moderate |
| Fidax:RBT | 0 | Some concerns | Some concerns | No concerns | Major concerns | No concerns | No concerns | Very low |
| Fidax:Ridin | 0 | No concerns | Some concerns | No concerns | Major concerns | No concerns | No concerns | Very low |
| Fidax:SER | 0 | Some concerns | Some concerns | No concerns | Major concerns | No concerns | No concerns | Very low |
| Fidax:Suro | 0 | No concerns | Some concerns | No concerns | Major concerns | No concerns | No concerns | Very low |
| Fidax:Teico | 0 | Some concerns | Some concerns | No concerns | Major concerns | No concerns | No concerns | Very low |
| Fidax:Tolev | 0 | Some concerns | Some concerns | No concerns | No concerns | No concerns | No concerns | High |
| Fidax:Vanco+BL | 0 | Some concerns | Some concerns | No concerns | Major concerns | No concerns | No concerns | Very low |
| Metro:RBT | 0 | Some concerns | Some concerns | No concerns | Major concerns | No concerns | No concerns | Very low |
| Metro:Ridin | 0 | Some concerns | Some concerns | No concerns | Major concerns | No concerns | No concerns | Very low |
| Metro:SER | 0 | Some concerns | Some concerns | No concerns | Major concerns | No concerns | No concerns | Very low |
| Metro:Suro | 0 | Some concerns | Some concerns | No concerns | Major concerns | No concerns | No concerns | Very low |
| Metro:Vanco+BL | 0 | Some concerns | Some concerns | No concerns | Major concerns | No concerns | No concerns | Very low |
| Metro+rif:Nita | 0 | Some concerns | Some concerns | No concerns | Major concerns | No concerns | No concerns | Very low |
| Metro+rif:Plac | 0 | Some concerns | Some concerns | No concerns | Major concerns | No concerns | No concerns | Very low |
| Metro+rif:RBT | 0 | Some concerns | Some concerns | No concerns | Major concerns | No concerns | No concerns | Very low |
| Metro+rif:Ridin | 0 | Some concerns | Some concerns | No concerns | Major concerns | No concerns | No concerns | Very low |
| Metro+rif:SER | 0 | Some concerns | Some concerns | No concerns | Major concerns | No concerns | No concerns | Very low |
| Metro+rif:Suro | 0 | Some concerns | Some concerns | No concerns | Major concerns | No concerns | No concerns | Very low |
| Metro+rif:Teico | 0 | Some concerns | Some concerns | No concerns | Major concerns | No concerns | No concerns | Very low |
| Metro+rif:Tolev | 0 | Some concerns | Some concerns | No concerns | Major concerns | No concerns | No concerns | Very low |
| Metro+rif:Vanco | 0 | Some concerns | Some concerns | No concerns | Major concerns | No concerns | No concerns | Very low |
| Metro+rif:Vanco+BL | 0 | Some concerns | Some concerns | No concerns | Major concerns | No concerns | No concerns | Very low |
| Nita:Plac | 0 | Some concerns | Some concerns | No concerns | Major concerns | No concerns | No concerns | Very low |
| Nita:RBT | 0 | Some concerns | Some concerns | No concerns | Major concerns | No concerns | No concerns | Very low |
| Nita:Ridin | 0 | Some concerns | Some concerns | No concerns | Major concerns | No concerns | No concerns | Very low |
| Nita:SER | 0 | Some concerns | Some concerns | No concerns | Major concerns | No concerns | No concerns | Very low |
| Nita:Suro | 0 | Some concerns | Some concerns | No concerns | Major concerns | No concerns | No concerns | Very low |
| Nita:Teico | 0 | Some concerns | Some concerns | No concerns | Major concerns | No concerns | No concerns | Very low |
| Nita:Tolev | 0 | Some concerns | Some concerns | No concerns | No concerns | No concerns | No concerns | High |
| Nita:Vanco+BL | 0 | Some concerns | Some concerns | No concerns | Major concerns | No concerns | No concerns | Very low |
| Plac:RBT | 0 | Some concerns | Some concerns | No concerns | Major concerns | No concerns | No concerns | Very low |
| Plac:Ridin | 0 | Some concerns | Some concerns | No concerns | Major concerns | No concerns | No concerns | Very low |
| Plac:Suro | 0 | Some concerns | Some concerns | No concerns | Major concerns | No concerns | No concerns | Very low |
| Plac:Teico | 0 | Some concerns | Some concerns | No concerns | Major concerns | No concerns | No concerns | Very low |
| Plac:Tolev | 0 | Some concerns | Some concerns | No concerns | Major concerns | No concerns | No concerns | Very low |
| Plac:Vanco+BL | 0 | Some concerns | Some concerns | No concerns | Major concerns | No concerns | No concerns | Very low |
| RBT:Ridin | 0 | Some concerns | Some concerns | No concerns | Major concerns | No concerns | No concerns | Very low |
| RBT:SER | 0 | Some concerns | Some concerns | No concerns | Major concerns | No concerns | No concerns | Very low |
| RBT:Suro | 0 | Some concerns | Some concerns | No concerns | Major concerns | No concerns | No concerns | Very low |
| RBT:Teico | 0 | Some concerns | Some concerns | No concerns | Major concerns | No concerns | No concerns | Very low |
| RBT:Tolev | 0 | Some concerns | Some concerns | No concerns | No concerns | Some concerns | No concerns | Moderate |
| RBT:Vanco+BL | 0 | Some concerns | Some concerns | No concerns | Major concerns | No concerns | No concerns | Very low |
| Ridin:SER | 0 | Some concerns | Some concerns | No concerns | Major concerns | No concerns | No concerns | Very low |
| Ridin:Suro | 0 | No concerns | Some concerns | No concerns | Major concerns | No concerns | No concerns | Very low |
| Ridin:Teico | 0 | Some concerns | Some concerns | No concerns | Major concerns | No concerns | No concerns | Very low |
| Ridin:Tolev | 0 | Some concerns | Some concerns | No concerns | No concerns | Some concerns | No concerns | Moderate |
| Ridin:Vanco+BL | 0 | Some concerns | Some concerns | No concerns | Major concerns | No concerns | No concerns | Very low |
| SER:Suro | 0 | Some concerns | Some concerns | No concerns | Major concerns | No concerns | No concerns | Very low |
| SER:Teico | 0 | Some concerns | Some concerns | No concerns | Major concerns | No concerns | No concerns | Very low |
| SER:Tolev | 0 | Some concerns | Some concerns | No concerns | Major concerns | No concerns | No concerns | Very low |
| SER:Vanco | 0 | Some concerns | Some concerns | No concerns | Major concerns | No concerns | No concerns | Very low |
| SER:Vanco+BL | 0 | Some concerns | Some concerns | No concerns | Major concerns | No concerns | No concerns | Very low |
| Suro:Teico | 0 | Some concerns | Some concerns | No concerns | Major concerns | No concerns | No concerns | Very low |
| Suro:Tolev | 0 | Some concerns | Some concerns | No concerns | No concerns | No concerns | No concerns | High |
| Suro:Vanco+BL | 0 | Some concerns | Some concerns | No concerns | Major concerns | No concerns | No concerns | Very low |
| Teico:Tolev | 0 | Some concerns | Some concerns | No concerns | Major concerns | No concerns | No concerns | Very low |
| Teico:Vanco+BL | 0 | Some concerns | Some concerns | No concerns | Major concerns | No concerns | No concerns | Very low |
| Tolev:Vanco+BL | 0 | Some concerns | Some concerns | No concerns | Major concerns | No concerns | No concerns | Very low |

# ***Table S37:* Grade: recurrency**

| Comparison | Number of studies | Within-study bias | Reporting bias | Indirectness | Imprecision | Heterogeneity | Incoherence | Confidence rating |
| --- | --- | --- | --- | --- | --- | --- | --- | --- |
| Bacit:Vanco | 1 | Some concerns | Some concerns | Some concerns | Major concerns | No concerns | Some concerns | Very low |
| FA:Metro | 2 | Some concerns | Some concerns | No concerns | Major concerns | No concerns | No concerns | Very low |
| FA:Teico | 1 | Some concerns | Some concerns | No concerns | No concerns | Some concerns | No concerns | Low |
| FA:Vanco | 1 | Some concerns | Some concerns | No concerns | Major concerns | No concerns | No concerns | Very low |
| FMT:FMT+bez | 1 | Some concerns | Some concerns | No concerns | Major concerns | No concerns | Some concerns | Very low |
| FMT:FMT-L | 1 | Some concerns | Some concerns | No concerns | Major concerns | No concerns | Some concerns | Very low |
| FMT:Vanco | 2 | Some concerns | Some concerns | No concerns | Major concerns | No concerns | Some concerns | Very low |
| FMT:Vanco+BL | 1 | Some concerns | Some concerns | No concerns | Major concerns | No concerns | Major concerns | Very low |
| Fidax:Vanco | 8 | No concerns | Some concerns | No concerns | No concerns | No concerns | Some concerns | High |
| Metro:Metro+rif | 1 | Some concerns | Some concerns | No concerns | Major concerns | No concerns | Some concerns | Very low |
| Metro:Teico | 1 | Some concerns | Some concerns | No concerns | Some concerns | Some concerns | No concerns | Moderate |
| Metro:Tolev | 1 | Some concerns | Some concerns | Some concerns | No concerns | No concerns | No concerns | Moderate |
| Metro:Vanco | 3 | Some concerns | Some concerns | Some concerns | Major concerns | No concerns | No concerns | Very low |
| Ridin:Vanco | 1 | No concerns | Some concerns | No concerns | No concerns | No concerns | Some concerns | High |
| Suro:Vanco | 2 | No concerns | Some concerns | Some concerns | Some concerns | Some concerns | Some concerns | Low |
| Teico:Vanco | 2 | Some concerns | Some concerns | Some concerns | Some concerns | Some concerns | No concerns | Low |
| Tolev:Vanco | 1 | Some concerns | Some concerns | Some concerns | No concerns | No concerns | No concerns | Moderate |
| Vanco:Vanco+BL | 1 | Some concerns | Some concerns | No concerns | Major concerns | No concerns | Major concerns | Very low |
| Bacit:FA | 0 | Some concerns | Some concerns | Some concerns | Major concerns | No concerns | Some concerns | Very low |
| Bacit:FMT | 0 | Some concerns | Some concerns | No concerns | Major concerns | No concerns | Some concerns | Very low |
| Bacit:FMT+bez | 0 | Some concerns | Some concerns | No concerns | Major concerns | No concerns | Some concerns | Very low |
| Bacit:FMT-L | 0 | Some concerns | Some concerns | No concerns | Major concerns | No concerns | Some concerns | Very low |
| Bacit:Fidax | 0 | Some concerns | Some concerns | Some concerns | No concerns | Some concerns | Some concerns | Very low |
| Bacit:Metro | 0 | Some concerns | Some concerns | Some concerns | Major concerns | No concerns | Some concerns | Very low |
| Bacit:Metro+rif | 0 | Some concerns | Some concerns | Some concerns | Major concerns | No concerns | Some concerns | Very low |
| Bacit:Ridin | 0 | Some concerns | Some concerns | Some concerns | No concerns | Some concerns | Some concerns | Very low |
| Bacit:Suro | 0 | Some concerns | Some concerns | Some concerns | Major concerns | No concerns | Some concerns | Very low |
| Bacit:Teico | 0 | Some concerns | Some concerns | Some concerns | Some concerns | Some concerns | Some concerns | Low |
| Bacit:Tolev | 0 | Some concerns | Some concerns | Some concerns | No concerns | No concerns | Some concerns | Low |
| Bacit:Vanco+BL | 0 | Some concerns | Some concerns | No concerns | Major concerns | No concerns | Some concerns | Very low |
| FA:FMT | 0 | Some concerns | Some concerns | No concerns | Major concerns | No concerns | Some concerns | Very low |
| FA:FMT+bez | 0 | Some concerns | Some concerns | No concerns | Major concerns | No concerns | Some concerns | Very low |
| FA:FMT-L | 0 | Some concerns | Some concerns | No concerns | Major concerns | No concerns | Some concerns | Very low |
| FA:Fidax | 0 | Some concerns | Some concerns | No concerns | No concerns | No concerns | Some concerns | Moderate |
| FA:Metro+rif | 0 | Some concerns | Some concerns | No concerns | Major concerns | No concerns | Some concerns | Very low |
| FA:Ridin | 0 | Some concerns | Some concerns | No concerns | No concerns | No concerns | Some concerns | Moderate |
| FA:Suro | 0 | Some concerns | Some concerns | No concerns | Major concerns | No concerns | Some concerns | Very low |
| FA:Tolev | 0 | Some concerns | Some concerns | Some concerns | No concerns | No concerns | Some concerns | Low |
| FA:Vanco+BL | 0 | Some concerns | Some concerns | No concerns | Major concerns | No concerns | Some concerns | Very low |
| Fidax:FMT | 0 | Some concerns | Some concerns | No concerns | Major concerns | No concerns | Some concerns | Very low |
| FMT:Metro | 0 | Some concerns | Some concerns | No concerns | Major concerns | No concerns | Some concerns | Very low |
| FMT:Metro+rif | 0 | Some concerns | Some concerns | No concerns | Major concerns | No concerns | Some concerns | Very low |
| FMT:Ridin | 0 | Some concerns | Some concerns | No concerns | Major concerns | No concerns | Some concerns | Very low |
| FMT:Suro | 0 | Some concerns | Some concerns | No concerns | Major concerns | No concerns | Some concerns | Very low |
| FMT:Teico | 0 | Some concerns | Some concerns | No concerns | Major concerns | No concerns | Some concerns | Very low |
| FMT:Tolev | 0 | Some concerns | Some concerns | Some concerns | Major concerns | No concerns | Some concerns | Very low |
| FMT+bez:FMT-L | 0 | Some concerns | Some concerns | No concerns | Major concerns | No concerns | Some concerns | Very low |
| Fidax:FMT+bez | 0 | Some concerns | Some concerns | No concerns | Major concerns | No concerns | Some concerns | Very low |
| FMT+bez:Metro | 0 | Some concerns | Some concerns | No concerns | Major concerns | No concerns | Some concerns | Very low |
| FMT+bez:Metro+rif | 0 | Some concerns | Some concerns | No concerns | Major concerns | No concerns | Some concerns | Very low |
| FMT+bez:Ridin | 0 | Some concerns | Some concerns | No concerns | Major concerns | No concerns | Some concerns | Very low |
| FMT+bez:Suro | 0 | Some concerns | Some concerns | No concerns | Major concerns | No concerns | Some concerns | Very low |
| FMT+bez:Teico | 0 | Some concerns | Some concerns | No concerns | Major concerns | No concerns | Some concerns | Very low |
| FMT+bez:Tolev | 0 | Some concerns | Some concerns | No concerns | Some concerns | Some concerns | Some concerns | Low |
| FMT+bez:Vanco | 0 | Some concerns | Some concerns | No concerns | Major concerns | No concerns | Some concerns | Very low |
| FMT+bez:Vanco+BL | 0 | Some concerns | Some concerns | No concerns | Major concerns | No concerns | Some concerns | Very low |
| Fidax:FMT-L | 0 | Some concerns | Some concerns | No concerns | Major concerns | No concerns | Some concerns | Very low |
| FMT-L:Metro | 0 | Some concerns | Some concerns | No concerns | Major concerns | No concerns | Some concerns | Very low |
| FMT-L:Metro+rif | 0 | Some concerns | Some concerns | No concerns | Major concerns | No concerns | Some concerns | Very low |
| FMT-L:Ridin | 0 | Some concerns | Some concerns | No concerns | Major concerns | No concerns | Some concerns | Very low |
| FMT-L:Suro | 0 | Some concerns | Some concerns | No concerns | Major concerns | No concerns | Some concerns | Very low |
| FMT-L:Teico | 0 | Some concerns | Some concerns | No concerns | Major concerns | No concerns | Some concerns | Very low |
| FMT-L:Tolev | 0 | Some concerns | Some concerns | No concerns | Major concerns | No concerns | Some concerns | Very low |
| FMT-L:Vanco | 0 | Some concerns | Some concerns | No concerns | Major concerns | No concerns | Some concerns | Very low |
| FMT-L:Vanco+BL | 0 | Some concerns | Some concerns | No concerns | Major concerns | No concerns | Some concerns | Very low |
| Fidax:Metro | 0 | Some concerns | Some concerns | Some concerns | No concerns | No concerns | Some concerns | Low |
| Fidax:Metro+rif | 0 | Some concerns | Some concerns | No concerns | Major concerns | No concerns | Some concerns | Very low |
| Fidax:Ridin | 0 | No concerns | Some concerns | No concerns | Major concerns | No concerns | Some concerns | Very low |
| Fidax:Suro | 0 | No concerns | Some concerns | No concerns | No concerns | No concerns | Some concerns | Moderate |
| Fidax:Teico | 0 | Some concerns | Some concerns | No concerns | Major concerns | No concerns | Some concerns | Very low |
| Fidax:Tolev | 0 | Some concerns | Some concerns | Some concerns | No concerns | Some concerns | Some concerns | Low |
| Fidax:Vanco+BL | 0 | Some concerns | Some concerns | No concerns | Major concerns | No concerns | Some concerns | Very low |
| Metro:Ridin | 0 | Some concerns | Some concerns | No concerns | No concerns | No concerns | Some concerns | Moderate |
| Metro:Suro | 0 | Some concerns | Some concerns | Some concerns | Major concerns | No concerns | Some concerns | Very low |
| Metro:Vanco+BL | 0 | Some concerns | Some concerns | No concerns | Major concerns | No concerns | Some concerns | Very low |
| Metro+rif:Ridin | 0 | Some concerns | Some concerns | No concerns | Major concerns | No concerns | Some concerns | Very low |
| Metro+rif:Suro | 0 | Some concerns | Some concerns | No concerns | Major concerns | No concerns | Some concerns | Very low |
| Metro+rif:Teico | 0 | Some concerns | Some concerns | No concerns | Major concerns | No concerns | Some concerns | Very low |
| Metro+rif:Tolev | 0 | Some concerns | Some concerns | Some concerns | No concerns | Some concerns | Some concerns | Low |
| Metro+rif:Vanco | 0 | Some concerns | Some concerns | No concerns | Major concerns | No concerns | Some concerns | Very low |
| Metro+rif:Vanco+BL | 0 | Some concerns | Some concerns | No concerns | Major concerns | No concerns | Some concerns | Very low |
| Ridin:Suro | 0 | No concerns | Some concerns | No concerns | No concerns | Some concerns | Some concerns | Moderate |
| Ridin:Teico | 0 | Some concerns | Some concerns | No concerns | Major concerns | No concerns | Some concerns | Very low |
| Ridin:Tolev | 0 | Some concerns | Some concerns | Some concerns | Some concerns | Some concerns | Some concerns | Low |
| Ridin:Vanco+BL | 0 | Some concerns | Some concerns | No concerns | Major concerns | No concerns | Some concerns | Very low |
| Suro:Teico | 0 | Some concerns | Some concerns | Some concerns | Major concerns | No concerns | Some concerns | Very low |
| Suro:Tolev | 0 | Some concerns | Some concerns | Some concerns | No concerns | No concerns | Some concerns | Low |
| Suro:Vanco+BL | 0 | Some concerns | Some concerns | No concerns | Major concerns | No concerns | Some concerns | Very low |
| Teico:Tolev | 0 | Some concerns | Some concerns | Some concerns | Major concerns | No concerns | Some concerns | Very low |
| Teico:Vanco+BL | 0 | Some concerns | Some concerns | No concerns | Major concerns | No concerns | Some concerns | Very low |
| Tolev:Vanco+BL | 0 | Some concerns | Some concerns | Some concerns | No concerns | No concerns | Some concerns | Low |

# ***Table S38:* Grade: prevention**

| Comparison | Number of studies | Within-study bias | Reporting bias | Indirectness | Imprecision | Heterogeneity | Incoherence | Confidence rating |
| --- | --- | --- | --- | --- | --- | --- | --- | --- |
| AB:Plac | 6 | Some concerns | Some concerns | No concerns | Some concerns | Some concerns | No concerns | Moderate |
| AB:Probi | 1 | Some concerns | Some concerns | Some concerns | Major concerns | No concerns | No concerns | Very low |
| ACT:ACT+BEZ | 1 | No concerns | Some concerns | No concerns | Major concerns | No concerns | No concerns | Very low |
| ACT:BEZ | 1 | No concerns | Some concerns | No concerns | Major concerns | No concerns | No concerns | Very low |
| ACT:Plac | 1 | No concerns | Some concerns | No concerns | Major concerns | No concerns | No concerns | Very low |
| ACT+BEZ:BEZ | 1 | No concerns | Some concerns | No concerns | Major concerns | No concerns | No concerns | Very low |
| ACT+BEZ:Plac | 1 | No concerns | Some concerns | No concerns | Some concerns | Some concerns | No concerns | Low |
| BEZ:Plac | 1 | No concerns | Some concerns | No concerns | Major concerns | No concerns | No concerns | Very low |
| LF:Plac | 1 | No concerns | Some concerns | No concerns | Major concerns | No concerns | No concerns | Very low |
| Oligo:Plac | 1 | No concerns | Some concerns | No concerns | No concerns | No concerns | No concerns | High |
| Plac:Probi | 19 | Some concerns | Some concerns | Some concerns | Some concerns | Some concerns | No concerns | Low |
| Plac:RBX | 1 | No concerns | Some concerns | No concerns | Major concerns | No concerns | No concerns | Very low |
| Plac:Vac | 1 | Some concerns | Some concerns | No concerns | Major concerns | No concerns | No concerns | Very low |
| AB:ACT | 0 | No concerns | Some concerns | No concerns | Major concerns | No concerns | No concerns | Very low |
| AB:ACT+BEZ | 0 | No concerns | Some concerns | No concerns | Major concerns | No concerns | No concerns | Very low |
| AB:BEZ | 0 | No concerns | Some concerns | No concerns | Major concerns | No concerns | No concerns | Very low |
| AB:LF | 0 | No concerns | Some concerns | No concerns | Major concerns | No concerns | No concerns | Very low |
| AB:Oligo | 0 | No concerns | Some concerns | No concerns | Some concerns | Some concerns | No concerns | Low |
| AB:RBX | 0 | No concerns | Some concerns | No concerns | Major concerns | No concerns | No concerns | Very low |
| AB:Vac | 0 | Some concerns | Some concerns | No concerns | Major concerns | No concerns | No concerns | Very low |
| ACT:LF | 0 | No concerns | Some concerns | No concerns | Major concerns | No concerns | No concerns | Very low |
| ACT:Oligo | 0 | No concerns | Some concerns | No concerns | Major concerns | No concerns | No concerns | Very low |
| ACT:Probi | 0 | No concerns | Some concerns | No concerns | Major concerns | No concerns | No concerns | Very low |
| ACT:RBX | 0 | No concerns | Some concerns | No concerns | Major concerns | No concerns | No concerns | Very low |
| ACT:Vac | 0 | No concerns | Some concerns | No concerns | Major concerns | No concerns | No concerns | Very low |
| ACT+BEZ:LF | 0 | No concerns | Some concerns | No concerns | Major concerns | No concerns | No concerns | Very low |
| ACT+BEZ:Oligo | 0 | No concerns | Some concerns | No concerns | Major concerns | No concerns | No concerns | Very low |
| ACT+BEZ:Probi | 0 | No concerns | Some concerns | No concerns | Major concerns | No concerns | No concerns | Very low |
| ACT+BEZ:RBX | 0 | No concerns | Some concerns | No concerns | Major concerns | No concerns | No concerns | Very low |
| ACT+BEZ:Vac | 0 | No concerns | Some concerns | No concerns | Major concerns | No concerns | No concerns | Very low |
| BEZ:LF | 0 | No concerns | Some concerns | No concerns | Major concerns | No concerns | No concerns | Very low |
| BEZ:Oligo | 0 | No concerns | Some concerns | No concerns | No concerns | Major concerns | No concerns | Very low |
| BEZ:Probi | 0 | No concerns | Some concerns | No concerns | Major concerns | No concerns | No concerns | Very low |
| BEZ:RBX | 0 | No concerns | Some concerns | No concerns | Major concerns | No concerns | No concerns | Very low |
| BEZ:Vac | 0 | No concerns | Some concerns | No concerns | Major concerns | No concerns | No concerns | Very low |
| LF:Oligo | 0 | No concerns | Some concerns | No concerns | Major concerns | No concerns | No concerns | Very low |
| LF:Probi | 0 | No concerns | Some concerns | No concerns | Major concerns | No concerns | No concerns | Very low |
| LF:RBX | 0 | No concerns | Some concerns | No concerns | Major concerns | No concerns | No concerns | Very low |
| LF:Vac | 0 | Some concerns | Some concerns | No concerns | Major concerns | No concerns | No concerns | Very low |
| Oligo:Probi | 0 | No concerns | Some concerns | No concerns | No concerns | Major concerns | No concerns | Very low |
| Oligo:RBX | 0 | No concerns | Some concerns | No concerns | Some concerns | Some concerns | No concerns | Low |
| Oligo:Vac | 0 | Some concerns | Some concerns | No concerns | No concerns | Some concerns | No concerns | Low |
| Probi:RBX | 0 | No concerns | Some concerns | No concerns | Major concerns | No concerns | No concerns | Very low |
| Probi:Vac | 0 | Some concerns | Some concerns | No concerns | Major concerns | No concerns | No concerns | Very low |
| RBX:Vac | 0 | Some concerns | Some concerns | No concerns | Major concerns | No concerns | No concerns | Very low |

# **Supplementary Discussion – Systematic review**

Cleary et al.^29^ examined whether metronidazole given during preparation for surgery in cases of pseudomembranous colitis promoted healing or the occurrence of CD infection. Although there was a lower incidence of CD infection in the group receiving metronidazole, the result was also not statistically significant.

Mattila et al.^30^ compared Metronidazole and CD immune whey, but the trial ended prematurely due to the sponsor's bankruptcy, although initial results were promising. McFarland et al.^31^ investigated whether *Saccharomyces* added to antibiotics reduces the rate of recurrence. In their study, the recurrence rate was significantly lower among those who received *Saccharomyces* than among those who received placebo. In the case of Teicoplanin, Wiström et al.^32^ performed a dose comparison, so their findings fell out from the network of this research. Patients who received higher doses of medication showed significantly better recovery. The study was terminated early due to a high relapse rate in the other group.

Wullt et al.’s^33^ result was not significant due to the small sample size. They compared methods of prevention that fell out of the network of prevention. One group received *Lactobacillus plantarum* in addition to metronidazole, while the other group received a placebo with metronidazole. Gao et al. also^34^ studied patients divided into three groups: the first who received a probiotic twice a day (*Lactobacillus acidophilus* CL1285® + *Lactobacillus casei* LBC80R®Bio-K + CL1285), the second who received probiotic once a day alongside a placebo, and the third group that received a placebo twice a day. They found that the incidence of CD infection was lowest among those who received two doses of probiotics, and highest among those who received two doses of placebos. The results were not significant, confirming the need for further, large-scare studies to evaluate the effectiveness of probiotics.

# **Supplementary References**

1 Robins J, Greenland S, Breslow N. A general estimator for the variance of the mantel-haenszel odds ratio. *Am J Epidemiol* 1986; : 23.

2 Mantel N, Haenszel W. Statistical Aspects of the Analysis of Data From Retrospective Studies of Disease. *JNCI J Natl Cancer Inst*  1959; **22**: 48.

3 Sweeting MJ, Sutton AJ, Lambert PC. What to add to nothing? Use and avoidance of continuity corrections in meta-analysis of sparse data. *Stat Med* 2004; **23**: 1351–75.

4 Cooper H, Hedges L, Valentine J. The handbook of research synthesis and meta-analysis, 2nd edn. New York: Russell Sage Foundation, 2009.

5 Paule R, Mandel J. Consensus values and weighting factors. *J Res Natl Bur Stand (1934)* 1982; **87**: 87.

6 Veroniki AA, Jackson D, Viechtbauer W, *et al.* Methods to estimate the between‐study variance and its uncertainty in meta‐analysis. *Res Synth Methods* 2016; **7**: 55–79.

7 Knapp G, Hartung J. Improved tests for a random effects meta-regression with a single covariate. *Stat Med* 2003; **22**: 2693–710.

8 Beausoleil M, Fortier N, Guénette S, *et al.* Effect of a fermented milk combining Lactobacillus acidophilus CL1285 and Lactobacillus casei in the prevention of antibiotic-associated diarrhea: A randomized, double-blind, placebo-controlled trial. *Can J Gastroenterol* 2007; **21**: 732–6.

9 Can M, Beşirbellioglu BA, Avci IY, Beker CM, Pahsa A. Prophylactic Saccharomyces boulardii in the prevention of antibiotic-associated diarrhea: A prospective study. *Med Sci Monit* 2006; **12**: 19–23.

10 Hickson M, D’Souza AL, Muthu N, *et al.* Use of probiotic Lactobacillus preparation to prevent diarrhoea associated with antibiotics: Randomised double blind placebo controlled trial. *Br Med J* 2007; **335**: 80–3.

11 Lewis SJ, Potts LF, Barry RE. The lack of therapeutic effect of Saccharomyces boulardii in the prevention of antibiotic-related diarrhoea in elderly patients. *J Infect* 1998; **36**: 171–4.

12 Pozzoni P, Riva A, Bellatorre AG, *et al.* Saccharomyces boulardii for the prevention of antibiotic-associated diarrhea in adult hospitalized patients: A single-center, randomized, double-blind, placebo-controlled trial. *Am J Gastroenterol* 2012; **107**: 922–31.

13 Rajkumar C, Wilks M, Islam J, *et al.* Do probiotics prevent antibiotic-associated diarrhoea? Results of a multicentre randomized placebo-controlled trial. *J Hosp Infect* 2020; **105**: 280–8.

14 Surawicz CM, Elmer GW, Speelman P, McFarland L V., Chinn J, Van Belle G. Prevention of antibiotic-associated diarrhea by Saccharomyces boulardii: A prospective study. *Gastroenterology* 1989; **96**: 981–8.

15 Thomas MR, Litin SC, Osmon DR, Corr AP, Weaver AL, Lohse CM. Lack of effect of Lactobacillus GG on antibiotic-associated diarrhea: A randomized, placebo-controlled trial. *Mayo Clin Proc* 2001; **76**: 883–9.

16 Wong S, Jamous A, O’Driscoll J, *et al.* A Lactobacillus casei Shirota probiotic drink reduces antibiotic-associated diarrhoea in patients with spinal cord injuries: A randomised controlled trial. *Br J Nutr* 2014; **111**: 672–8.

17 Rauseo AM, Hink T, Reske KA, *et al.* A randomized controlled trial of Lactobacillus rhamnosus GG on antimicrobial-resistant organism colonization. *Infect Control Hosp Epidemiol* 2022; **43**: 167–73.

18 Surawicz CM, McFarland L V., McFarland L V., *et al.* The search for a better treatment for recurrent Clostridium difficile disease: Use of high-dose vancomycin combined with Saccharomyces boulardii. *Clin Infect Dis* 2000; **31**: 1012–7.

19 Morrow LE, Kollef MH, Casale TB. Probiotic prophylaxis of ventilator-associated pneumonia: A blinded, randomized, controlled trial. *Am J Respir Crit Care Med* 2010; **182**: 1058–64.

20 Allen SJ, Wareham K, Wang D, *et al.* Lactobacilli and bifidobacteria in the prevention of antibiotic-associated diarrhoea and Clostridium difficile diarrhoea in older inpatients (PLACIDE): a randomised, double-blind, placebo-controlled, multicentre trial. *Lancet* 2013; **382**: 1249–57.

21 Plummer S, Weaver MA, Harris JC, Dee P, Huter J. Clostridium difficile pilot study: Effects of probiotic supplementation on the incidence of C. difficile diarrhoea. *Int Microbiol* 2004; **7**: 59–62.

22 Nord CE, Lidbeck A, Orrhage K, Sjöstedt S. Oral supplementation with lactic acid-producing bacteria during intake of clindamycin. *Clin Microbiol Infect* 1997; **3**: 124–32.

23 Ehrhardt S, Guo N, Hinz R, *et al.* Saccharomyces boulardii to prevent antibiotic-associated diarrhea: A randomized, double-masked, placebo-controlled trial. *Open Forum Infect Dis* 2016; **3**: 1–7.

24 Prasoon A, Singh R, Anand R, Kumar S, Singh S, Singh A. A Randomized Controlled Trial to Evaluate the Use of Probiotics in Prevention of Ventilator-Associated Pneumonia in Critically Ill ICU Patients. *J Card Crit Care TSS* 2022; **06**: 108–13.

25 Saviano A, Petruzziello C, Cancro C, *et al.* The Efficacy of a Mix of Probiotics (Limosilactobacillus reuteri LMG P-27481 and Lacticaseibacillus rhamnosus GG ATCC 53103) in Preventing Antibiotic-Associated Diarrhea and Clostridium difficile Infection in Hospitalized Patients: Single-Center, Open-Label, Randomized Trial. *Microorganisms* 2024; **12**. DOI:10.3390/microorganisms12010198.

26 Kao D, Roach B, Silva M, *et al.* Effect of oral capsule– vs colonoscopy-delivered fecal microbiota transplantation on recurrent Clostridium difficile infection: A randomized clinical trial. *JAMA - J Am Med Assoc* 2017; **318**: 1985–93.

27 Youngster I, Sauk J, Pindar C, *et al.* Fecal microbiota transplant for relapsing clostridium difficile infection using a frozen inoculum from unrelated donors: A randomized, open-label, controlled pilot study. *Clin Infect Dis* 2014; **58**: 1515–22.

28 Page MJ, McKenzie JE, Bossuyt PM, *et al.* The PRISMA 2020 statement: An updated guideline for reporting systematic reviews. *Int J Surg* 2021; **88**. DOI:10.1016/j.ijsu.2021.105906.

29 Cleary RK, Grossmann R, Fernandez FB, *et al.* Metronidazole may inhibit intestinal colonization with Clostridium difficile. *Dis Colon Rectum* 1998; **41**: 464–7.

30 Mattila E, Anttila VJ, Broas M, *et al.* A randomized, double-blind study comparing Clostridium difficile immune whey and metronidazole for recurrent Clostridium difficile-associated diarrhoea: Efficacy and safety data of a prematurely interrupted trial. *Scand J Infect Dis* 2008; **40**: 702–8.

31 Mcfarland L V., Surawicz CM, Elmer GW, *et al.* A Randomized Placebo-Controlled Trial of Saccharomyces boulardii in Combination With Standard Antibiotics for Clostridium difficile Disease. *JAMA J Am Med Assoc* 1994; **271**: 1913–8.

32 Wiström J. Treatment of clostridium difficile associated diarrhea and colitis with an oral preparation of teicoplanin; a dose finding study. *Scand J Infect Dis* 1994; **26**: 309–16.

33 Wullt M, Hagslätt MLJ, Odenholt I. Lactobacillus plantarum 299v for the treatment of recurrent clostridium difficile-associated diarrhoea: A double-blind, placebo-controlled trial. *Scand J Infect Dis* 2003; **35**: 365–7.

34 Gao XW, Mubasher M, Fang CY, Reifer C, Miller LE. Dose-response efficacy of a proprietary probiotic formula of lactobacillus acidophilus CL1285 and lactobacillus casei LBC80R for antibiotic-associated diarrhea and clostridium difficile-associated diarrhea prophylaxis in adult patients. *Am J Gastroenterol* 2010; **105**: 1636–41.
